# Two Synthetic Tools to Deepen the Understanding of the Influence of Stereochemistry on the Properties of Iridium(III) Heteroleptic Emitters

**DOI:** 10.1021/acs.inorgchem.3c03133

**Published:** 2023-11-21

**Authors:** Juan C. Babón, Pierre-Luc T. Boudreault, Miguel A. Esteruelas, Miguel A. Gaona, Susana Izquierdo, Montserrat Oliván, Enrique Oñate, Jui-Yi Tsai, Andrea Vélez

**Affiliations:** † Departamento de Química Inorgánica - Instituto de Síntesis Química y Catálisis Homogénea (ISQCH) - Centro de Innovación en Química Avanzada (ORFEO−CINQA), Universidad de Zaragoza - CSIC, 50009 Zaragoza, Spain; ‡ Universal Display Corporation, Ewing, New Jersey 08618, United States

## Abstract

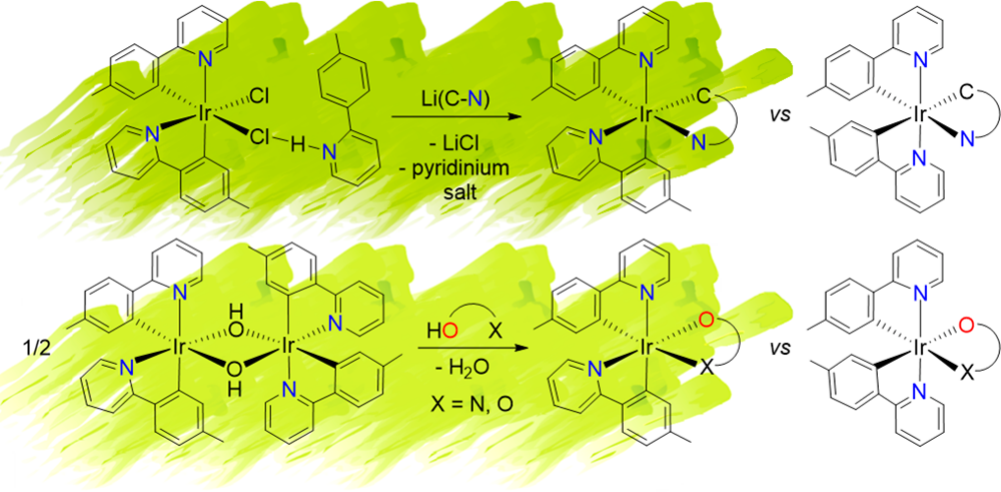

Two complementary procedures are presented to prepare *cis*-pyridyl-iridium(III) emitters of the class [**3b**+**3b**+**3b′**] with two orthometalated ligands of the 2-phenylpyridine type (**3b**) and a third ligand (**3b′**). They allowed to obtain four emitters of this class and to compare their properties with those of the *trans*-pyridyl isomers. The finding starts from IrH_5_(P^i^Pr_3_)_2_, which reacts with 2-(*p*-tolyl)pyridine to give *fac*-[Ir{κ^2^-*C,N*-[C_6_MeH_3_-py]}_3_] with an almost quantitative yield. Stirring the latter in the appropriate amount of a saturated solution of HCl in toluene results in the *cis*-pyridyl adduct IrCl{κ^2^-*C,N*-[C_6_MeH_3_-py]}_2_{κ^1^-*Cl*-[Cl–H-py-C_6_MeH_4_]} stabilized with *p*-tolylpyridinium chloride, which can also be transformed into dimer *cis*-[Ir(μ-OH){κ^2^-*C,N*-[C_6_MeH_3_-py]}_2_]_2_. Adduct IrCl{κ^2^-*C,N*-[C_6_MeH_3_-py]}_2_{κ^1^-*Cl*-[Cl–H-py-C_6_MeH_4_]} directly generates *cis*-[Ir{κ^2^-*C,N*-[C_6_MeH_3_-py]}_2_{κ^2^-*C,N*-[C_6_H_4_–Isoqui]}] and *cis*-[Ir{κ^2^-*C,N*-[C_6_MeH_3_-py]}_2_{κ^2^-*C,N*-[C_6_H_4_-py]}] by transmetalation from Li[2-(isoquinolin-1-yl)-C_6_H_4_] and Li[py-2-C_6_H_4_]. Dimer *cis*-[Ir(μ-OH){κ^2^-*C,N*-[C_6_MeH_3_-py]}_2_]_2_ is also a useful starting complex when the precursor molecule of **3b′** has a fairly acidic hydrogen atom, suitable for removal by hydroxide groups. Thus, its reactions with 2-picolinic acid and acetylacetone (Hacac) lead to *cis*-Ir{κ^2^-*C,N*-[C_6_MeH_3_-py]}_2_{κ^2^-*O,N*-[OC(O)-py]} and *cis*-Ir{κ^2^-*C,N*-[C_6_MeH_3_-py]}_2_{κ^2^-*O,O*-[acac]}. The stereochemistry of the emitter does not significantly influence the emission wavelengths. On the contrary, its efficiency is highly dependent on and associated with the stability of the isomer. The more stable isomer shows a higher quantum yield and color purity.

## Abstract 2

Two complementary procedures have been discovered to prepare *cis*-pyridyl-iridium(III) emitters of the class [**3b**+**3b**+**3b′**] with two orthometalated ligands of the 2-phenylpyridine type (**3b**) and a third ligand (**3b′**). These methods provide the entry to complexes with **3b′** being *C,N*-, *N,O-*, or *O,O*-ligands. The comparison of their photophysical properties with those of the usual *trans*-pyridyl counterparts is provided.

## Introduction

Organic light emitting diodes (OLEDs) are proposed as the near future for lighting and display applications; the devices based on phosphorescent emitters, PHOLEDs, are the most promising, since they improve the characteristics of those that use fluorescent dopants.^1^ The 5d metal containing PHOLEDs show rapid crossover between the S_1_–T_1_ states,^2^ allowing them to collect singlet and triplet excitons, thus achieving internal quantum efficiencies close to 100%.^3^ Vacuum thermal evaporation of the emissive dopant and subsequent vapor deposition are the predominant methods for the manufacture of PHOLEDs.^4^ As a consequence, charge-neutral 5d metal complexes of high stability are required for commercial fabrication. In this context, octahedral iridium(III) complexes are particularly suitable. Due to a high octahedral Δ_0_ splitting, the 5d^6^ electronic configuration of the metal center always has a low spin, which maximizes the stabilization energy of the ligand field. This hinders the M–L ruptures, reducing the emitter decomposition pathways.^5^

**Scheme 1:**
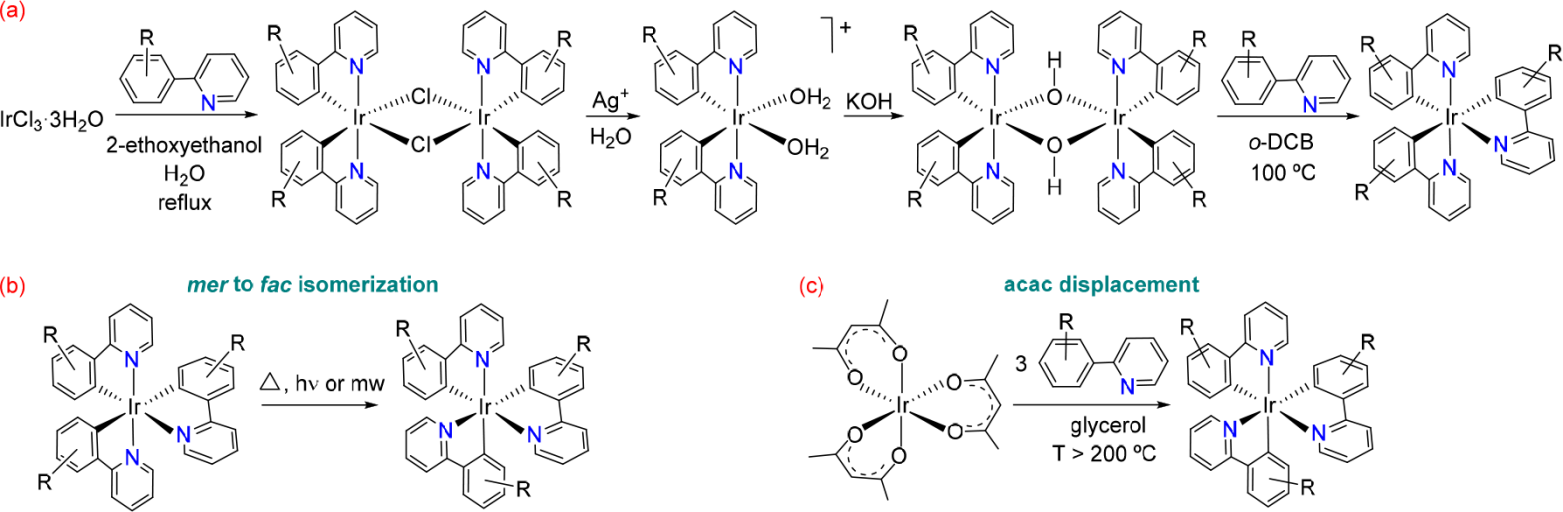
Synthesis Methods of Homoleptic-Ir(III) Complexes

The iridium(III) complexes that contain three orthometalated groups, belonging to the 2-phenylpyridine family, are archetypal emitters that should be especially highlighted. Due to their properties, these homoleptic compounds bearing three identical bidentate ligand donors of 3 electrons each (**3b**) are at the forefront of photophysics^6^ and photochemistry.^7^ They may have a meridional (*mer*) or facial (*fac*) configuration. The *mer*-isomers are kinetically favored; they can be obtained by performing the reactions at moderate temperatures (<150 °C), which inhibit the formation of the thermodynamic *fac*-isomers.^8^ An efficient approach to selective *mer*-isomer preparation starts from IrCl_3_·3H_2_O and involves four steps (Scheme 1a). The salt is initially transformed into a dichloro-bridged iridium(III) dimer, *trans*-[Ir(μ-Cl)(**3b**)_2_]_2_, by reaction with the corresponding 2-phenylpyridine-type molecule in 2-ethoxyethanol–water at reflux.^9^ Extraction of chloride ligands with AgBF_4_ in acetone followed by addition of water leads to the corresponding water solvate, a mononuclear cationic intermediate, which reacts with KOH to give a dihydroxide-bridged dimer *trans*-[Ir(μ-OH)(**3b**)_2_]_2_.^10^ Characteristics of this class of dimers include the *trans* arrangement of the heterocycles in the mononuclear moiety and the retention of this arrangement in their reactions. Thus, treatment of the dihydroxide-bridged dimer with the 2-phenylpyridine-type molecule in *ortho*-dichlorobenzene at 100 °C provides the *mer*-isomer.^11^ Rational preparation of the *fac*-isomers has been achieved mainly by two methods (Scheme 1b): isomerization of the *mer*-complexes and displacement of the acetylacetonate (acac) ligand from Ir(acac)_3_.^8^ Isomerization from *mer* to *fac* can be induced thermally, photochemically,^12^ or by microwave irradiation;^13^ in most cases, the *mer*-isomer is generated *in situ* from IrCl_3_·3H_2_O. The acac displacement is generally performed in glycerol at a temperature above 200 °C.^14^ The marked differences in the electrochemical and photophysical properties observed between both types of isomers is noteworthy.^15^ The *mer*-isomers are easier to oxidize. They show less color purity and red-shifted emissions relative to the *fac*-isomers. Furthermore, *fac*-isomers have longer lifetimes and higher quantum yields in solution.^8^

**Scheme 2:**
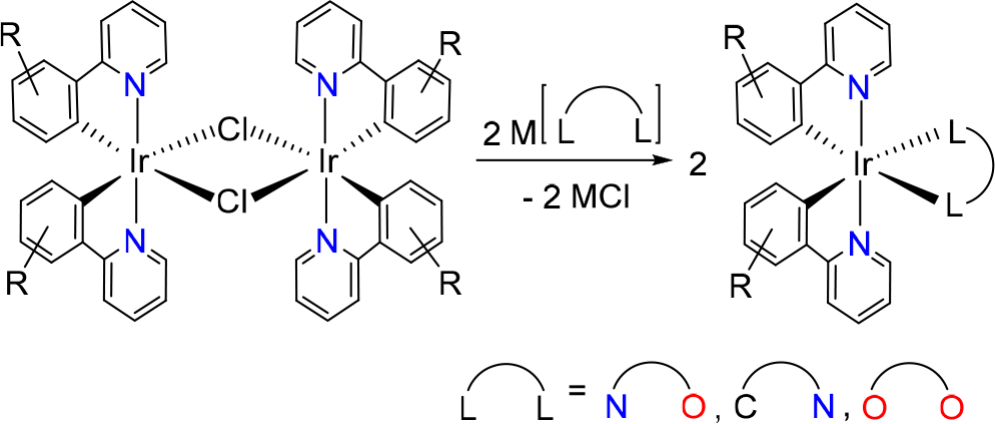
Preparation of Heteroleptic-Ir(III) Emitters

The presence of different ligands in the coordination sphere of the iridium(III) center facilitates a better tuning of the photophysical and photochemical characteristics of the emitters; those stabilized by two different bidentate groups, [**3b**+**3b**+**3b′**],^16^ are the most common because they present less problems associated with ligand distribution equilibria and have fewer stereoisomers than the emitters of the class [**3b**+**3b′**+**3b″**], formed by three different bidentate ligands.^17^ Emitters of the class [**3b**+**3b**+**3b′**] usually contain two orthometalated 2-phenylpyridine-type groups and a third **3b′***O,N*-, *C,N*-, or *O,O*-donor ligand. The preparation of these complexes involves the replacement of the chloride bridges of *trans*-[Ir(μ-Cl)(**3b**)_2_]_2_ dimers by the **3b′** ligand (Scheme 2).^18^ As a consequence of the use of these dimers, a common structural feature of the resulting emitters is the *trans* arrangement of the pyridyl groups.^16,18^ Thus, these species are heteroleptic counterparts of the homoleptic *mer*-isomers. On the other hand, there is no general procedure that allows the preparation of analogs that carry *cis*-pyridyl groups, the equivalents of the homoleptic *fac*-isomers. The isomerization of *trans*-pyridyl to *cis*-pyridyl is highly dependent on the **3b′** ligand. It has relative success by irradiation with visible or ultraviolet light, limited to particular cases,^19^ while it is generally partial by heat treatment including sublimation.^20^ In solution, the nature of the solvent seems to play a fundamental role.^19^

**Scheme 3:**
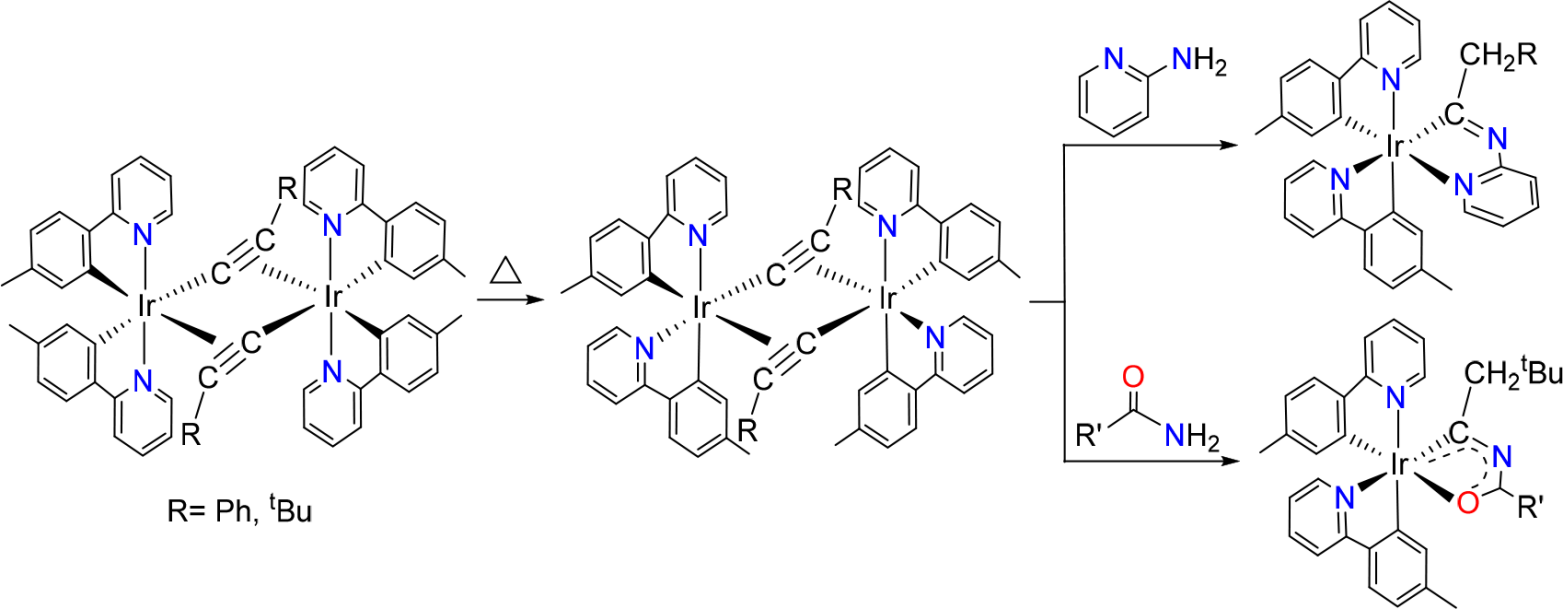
Synthesis of Iridaimidazo[1,2-*a*]pyridine and Iridaoxazole Complexes

Notable exceptions are the recently discovered iridaimidazo[1,2-*a*]pyridine^21^ and iridaoxazole^22^ complexes with two orthometalated 2-(*p*-tolyl)pyridine ligands. Despite their heteroleptic nature, they show a *fac* arrangement of *C*- and *N*-donor atoms as the homoleptic emitters shown in Scheme 1b. The procedure for their preparation involves substitution of the chloride bridges of the *trans*-[Ir(μ-Cl)(**3b**)_2_]_2_ dimers by acetylides and uses the ability of these groups to act as building blocks. Unlike the dichloro-bridged dimers, the diacetylide-bridged dimers, *trans*-[Ir(μ^2^-η^2^-C≡CR)(**3b**)_2_]_2_, exchange the relative positions of the donor atoms of one of the **3b** ligands to give *cis*-[Ir(μ^2^-η^2^-C≡CR)(**3b**)_2_]_2_, with *cis*-pyridyl groups, which are thermodynamically preferred. Subsequent reactions of the acetylide bridges with 2-aminopyridine and amides give the respective diheterometallacycles (Scheme 3).^21,22^

The recent discovery of these iridaimidazo[1,2-*a*]pyridine and iridaoxazole complexes and the thermodynamic preference of *cis*-[Ir(μ^2^-η^2^-C≡CR)(**3b**)_2_]_2_ over *trans*-[Ir(μ^2^-η^2^-C≡CR)(**3b**)_2_]_2_ led us to try to prepare a dichloro-bridged dimer, *cis*-[Ir(μ-Cl)(**3b**)_2_]_2_, with *cis*-pyridyl groups, with the aim of developing a synthetic procedure for preparing the heteroleptic equivalents of the homoleptic *fac*-isomers. This article describes a new procedure, more efficient than previously reported, to prepare homoleptic iridium(III) complexes containing three *fac*-arranged 2-phenylpyridine-type orthometalated groups. It shows that these thermodynamic isomers are a useful starting point to obtain dimers *cis*-[Ir(μ-Cl)(**3b**)_2_]_2_, points out pathways to prepare heteroleptic equivalents of the homoleptic *fac*-isomers, and compares the electrochemical and photophysical properties of the *trans*- and *cis*-pyridyl heteroleptic isomers.

## Results and Discussion

### Preparation of Tris(cyclometalated)-Iridium(III) *fac*-Homoleptic Emitters from a Polyhydride

Platinum group metal polyhydride complexes have a special ability to activate σ-bonds, particularly C–H.^23^ Associated with this capacity is their importance in homogeneous catalysis^24^ and recently also their use as starting compounds to develop novel procedures for the preparation of original osmium(II), osmium(IV), and iridium(III) phosphorescent emitters.^25^ Inspired by such a relevant ability of polyhydride complexes, useful in markedly different fields, we decided to use iridium(V) pentahydride IrH_5_(P^i^Pr_3_)_2_ (**1**) as starting material to obtain homoleptic *fac*-isomers with orthometalated 2-phenylpyridine-type ligands, as just happened. This pentahydride is easily prepared in high yield (>75%) from the iridium(III) compound IrHCl_2_(P^i^Pr_3_)_2_ by reaction with NaBH_4_, a significant improved procedure over those previously used.^26^ Its formation takes place via the tetrahydroborate intermediate IrH_2_{κ^2^-*H,H*-[BH_4_]}(P^i^Pr_3_)_2_.^27^ 1-Phenylethanol was employed as a solvent because it prevents carbonylation of the metal center, given its secondary nature, unlike primary alcohols such as 2-ethoxyethanol. Additionally, 1-phenylethanol also has a reasonable boiling point of 204 °C. Its utilization has been successful in coordinating tetradentate and hexadentate ligands from pro-ligands that need two and three C–H activations, including a C(sp^3^)–H bond.^28^

**Scheme 4:**
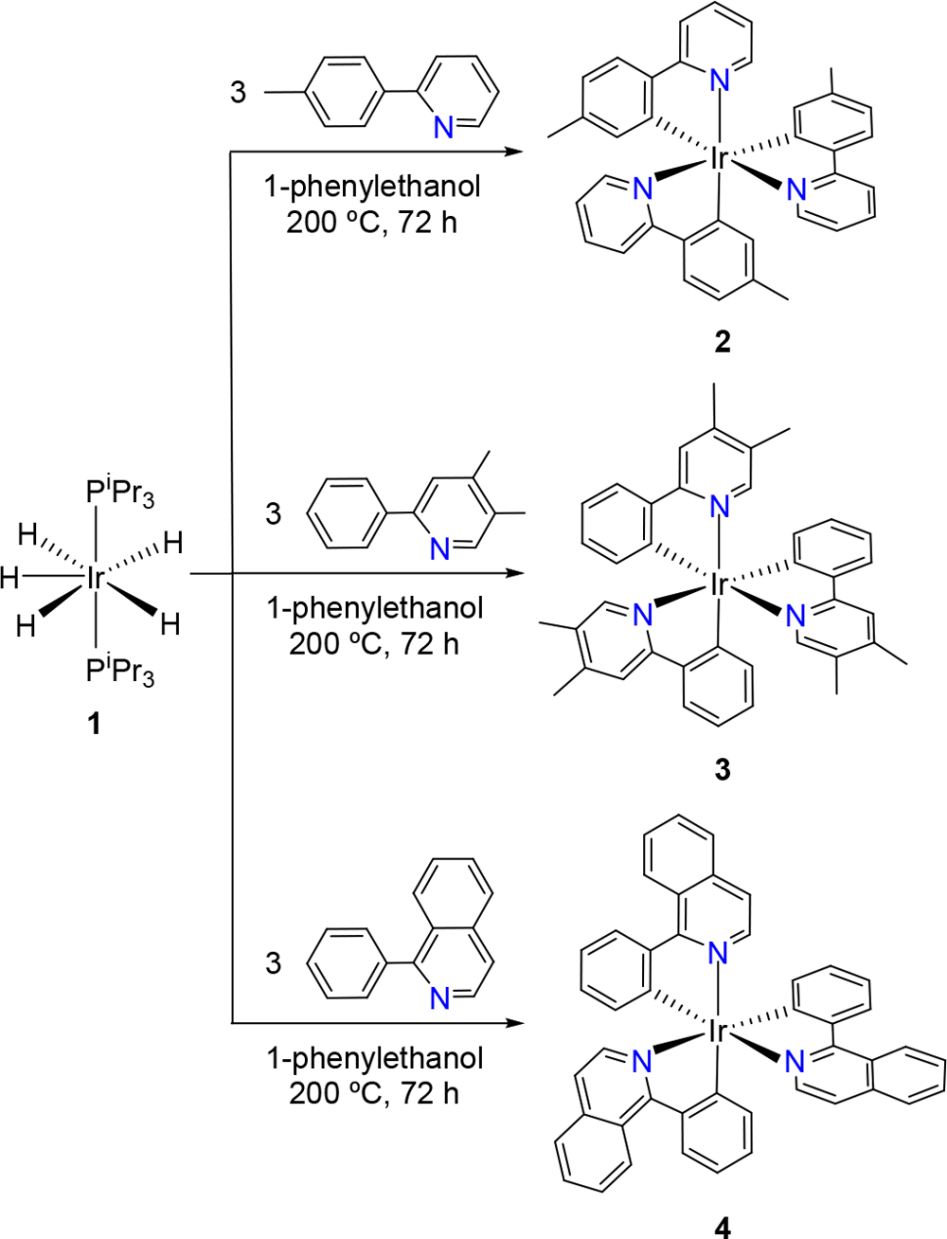
Reactions of IrH_5_(P^i^Pr_3_)_2_ with 2-(*p*-Tolyl)pyridine, 4,5-Dimethyl-2-phenylpyridine, and 1-Phenylisoquinoline

2-(*p*-Tolyl)pyridine, 4,5-dimethyl-2-phenylpyridine, and 1-phenylisoquinoline were selected as examples of 2-phenylpyridine-type molecules for validation of the starting-polyhydride method: the first as a representation of a substituted phenyl pro-ligand, the second as a substituted pyridyl case, and the third as a prototype of a fused aromatic ring heterocycle. Treatment of complex **1** with 5.0 equiv of these molecules, in refluxing 1-phenylethanol, for 72 h leads to the corresponding *fac*-[Ir(**3b**)_3_] derivatives (Scheme 4). The pyridyl derivatives *fac*-[Ir{κ^2^-*C,N*-[C_6_MeH_3_-py]}_3_] (**2**) and *fac*-[Ir{κ^2^-*C,N*-[C_6_H_4_-pyMe_2_]}_3_] (**3**) were isolated as analytically pure yellow solids in high yields (∼85%) after concentration of solutions in 1-phenylethanol and subsequent addition of diethyl ether, whereas the isoquinolyl counterpart *fac*-[Ir{κ^2^-*C,N*-[C_6_H_4_–Isoqui]}_3_] (**4**) was obtained as an analytically pure red solid, in almost quantitative yield, by the same procedure.

**Figure 1:**
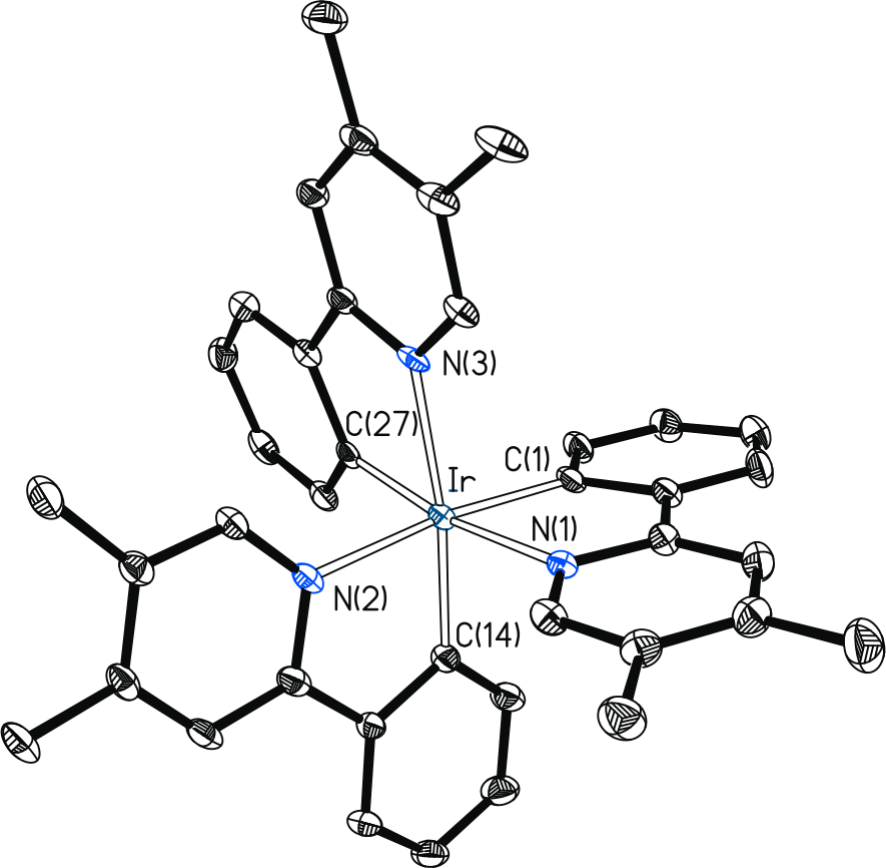
Molecular diagram of complex **3** (displacement ellipsoids shown at 50% probability). All hydrogen atoms are omitted for clarity. Selected bond distances (Å) and angles (deg): Ir–C(1) = 1.987(3), Ir–C(14) = 1.972(3), Ir–C(27) = 2.053(4), Ir–N(1) = 2.175(3), Ir–N(2) = 2.089(3), Ir–N(3) = 2.118(3); C(1)–Ir–N(1) = 83.13(13), C(14)–Ir–N(2) = 86.15(13), C(27)–Ir–N(3) = 81.62(13), C(1)–Ir–N(2) = 172.55(13), C(14)–Ir–N(3) = 171.88(13), C(27)–Ir–N(1) = 171.60(11), N(1)–Ir–N(2) = 92.07(11), N(1)–Ir–N(3) = 92.61(12), N(2)–Ir–N(3) = 88.12(11).

The X-ray diffraction analysis structure of the new emitter complex **3** (Figure 1) validates the method and further demonstrates the *fac* arrangement of the *C*- and *N*-donor atoms. The coordination polyhedron around the iridium center is the expected distorted octahedron with angles *trans*-C–Ir–N in the range 172.55(13)°–171.60(11)°. Consistent with the structure, the ^1^H and ^13^C{^1^H} NMR spectra of this compound, in dichloromethane-*d*_2_, at room temperature show resonances for equivalent ligands, the most notorious signals being two singlets corresponding to the methyl groups at 2.36 and 2.05 ppm in ^1^H and at 19.8 and 16.7 ppm in ^13^C{^1^H}. The ^1^H and ^13^C{^1^H} NMR spectra of **2**(8) and **4**(14d,14e) agree well with those previously reported for these compounds.

### Starting Complexes with a *cis*-Pyridyl Arrangement

Aoki and co-workers previously reported the degradation of *fac*-[Ir(**3b**)_3_] isomers to *trans*-[Ir(μ-X)(**3b**)_2_]_2_ dimers, which show a *trans* arrangement of pyridyl groups. Reactions were carried out using halogenated Brønsted and Lewis acids, including solutions of HCl in 1,4-dioxane, in 1,2-dichloroethane as solvent. They propose the formation of five-coordinate intermediates [IrX(**3b**)_2_] as key species for the rearrangement of **3b** ligands within mononuclear units.^17c^ Obviously, the coordination capacity of the solvent must play an essential role in the stabilization of said intermediates. Accordingly, we reasoned that one way to avoid the position exchange of the pyridyl groups in these mononuclear intermediates should be to shorten the existence of said unsaturated intermediates by using a solvent with lower coordination ability that facilitates the dimerization. With this aim, we decided to employ saturated solutions of HCl in toluene (∼0.20 M) as degradation agent and reaction solvent.

**Scheme 5:**
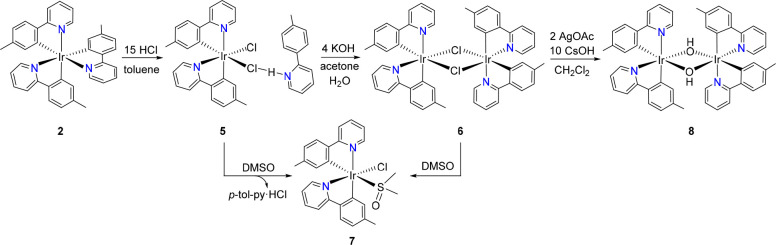
Preparation of Starting Materials to Generate Heteroleptic Emitters with *cis*-Pyridyl Arrangement

Stirring homoleptic complex **2** in said solution, for 12 h, at room temperature effectively produces the extraction of one of the chelates from the iridium coordination sphere and the formation of a five-coordinate intermediate [IrCl(**3b**)_2_], which is not stabilized by toluene. However, this unsaturated intermediate, which has not yet undergone rearrangement of the pyridyl groups, does not experience dimerization. The surprising reason is its stabilization by saturation of the coordination vacancy of the iridium center by donating electrons from the chlorine atom of Cl–H-py-C_6_MeH_4_. This adduct results from the addition of solvated HCl, which is present in excess in the starting solution, to the released pyridine. Thus, the stable complex IrCl{κ^2^-*C,N*-[C_6_MeH_3_-py]}_2_{κ^1^-*Cl*-[Cl–H-py-C_6_MeH_4_]} (**5**) with the pyridyl groups arranged in *cis* is formed (Scheme 5).

**Figure 2:**
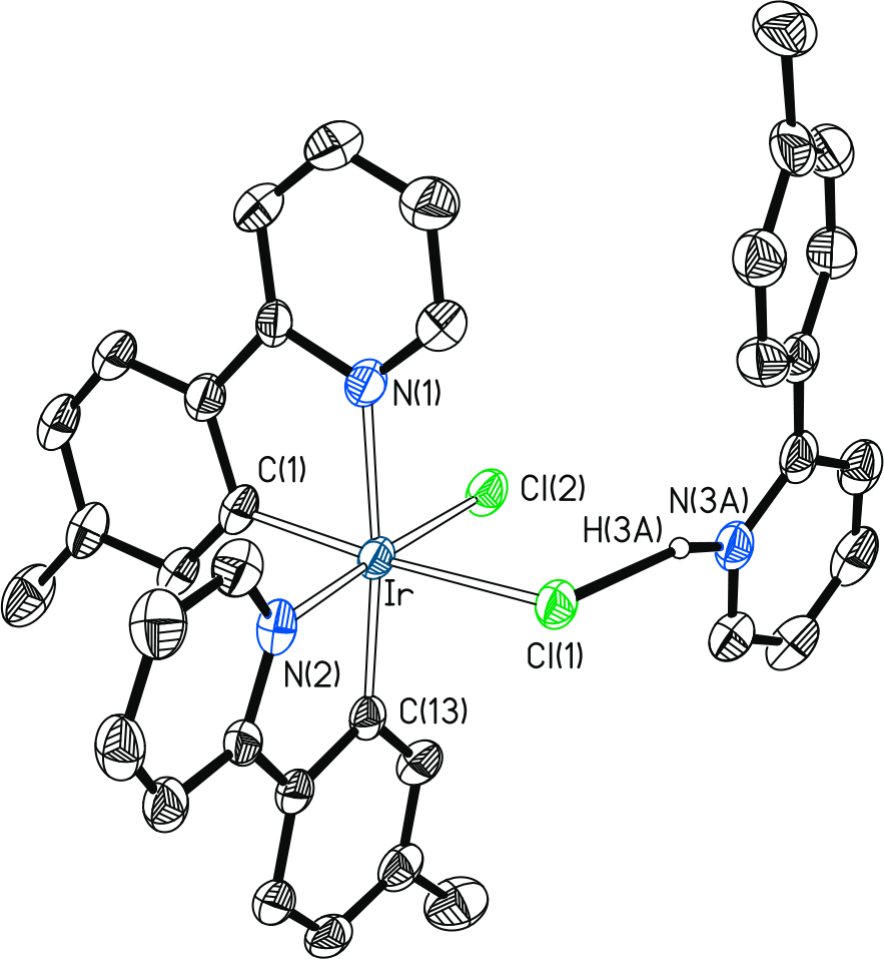
Molecular diagram of complex **5** (displacement ellipsoids shown at 50% probability). All hydrogen atoms (except that of pyridinium) are omitted for clarity. Selected bond distances (Å) and angles (deg): Ir–C(1) = 2.004(6), Ir–C(13) = 2.006(6), Ir–N(1) = 2.127(6), Ir–N(2) = 2.019(6), Ir–Cl(1) = 2.5151(16), Ir–Cl(2) = 2.3935(16), Cl(1)–H(3A) = 2.02(8), N(3A)–H(3A) = 1.13(8); Cl(1)–Ir–Cl(2) = 91.16(6), N(2)–Ir–Cl(2) = 177.04(16), Cl(1)–Ir–C(1) = 173.78(19), N(1)–Ir–N(2) = 96.0(2), C(1)–Ir–N(2) = 90.3(2), C(1)–Ir–N(1) = 80.0(2), C(13)–Ir–N(2) = 80.4(2), C(13)–Ir–N(1) = 173.8(2).

Complex **5** was isolated as a yellow solid with a yield of 86% and characterized by X-ray diffraction analysis. The structure (Figure 2) proves the *cis* arrangement of the heterocycles (N(1)–Ir–N(2) = 96.0(2)°) and the coordination of the Cl(1) atom of pyridinium chloride, which is found *cis* to the chloride ligand Cl(2) in an octahedral environment around the iridium center (Cl(1)–Ir–Cl(2) = 91.16(6)°). As expected, the iridium-chloride bond length (Ir–Cl(2) = 2.3935(16) Å) is significantly shorter (∼0.12 Å) than the iridium–pyridinium chloride distance (Ir–Cl(1) = 2.5151(16) Å). The heterocycle of one of the chelates is arranged in the *trans* position with respect to the phenyl group of the other chelate (N(1)–Ir–C(13) = 173.8(2)°), while the heterocycle of the latter is placed *trans* to the chloride ligand (N(2)–Ir–C(2) = 177.04(16)°). Thus, the pyridinium chloride is necessarily *trans* to the remaining phenyl group (Cl(1)–Ir–C(1) = 173.78(19)°). The iridium–phenyl distances of 2.004(6) (Ir–C(1)) and 2.006(6) (Ir–C(13)) Å are not sensitive to the group they have in *trans*. In contrast, the lengths of the iridium–heterocycle bonds are highly dependent on the *trans* influence of the group arranged in *trans*; the heterocycle located *trans* to the chloride ligand (Ir–N(2) = 2.019(6) Å) is further separated from iridium, around 0.11 Å, than the heterocycle positioned *trans* to the phenyl group of the other chelate (Ir–N(1) = 2.127(6) Å). The Cl(1)–H(3A) distance in the coordinated pyridinium chloride of 2.02(8) Å, which is about 1.0 Å shorter than the sum of the van der Waals radii of hydrogen and chloride (*r*_vdw_(H) = 1.20 Å, *r*_vdw_(Cl) = 1.75 Å), should be mentioned.^29^

**Figure 3:**
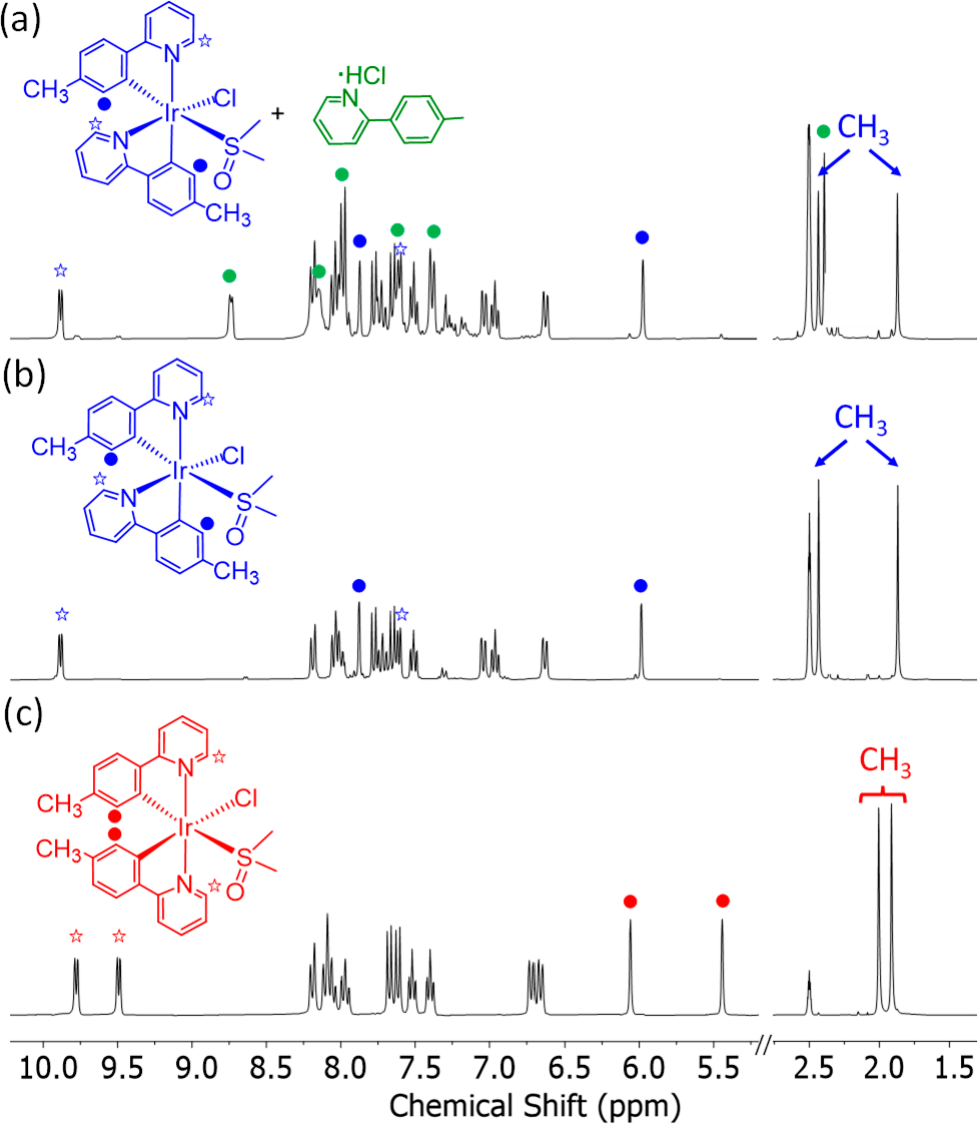
^1^H NMR spectra (300 MHz, DMSO-*d*_6_, 298 K) of *cis*-[IrCl{κ^2^-*C,N*-[C_6_MeH_3_-py]}_2_{κ^1^-*S*-[S(O)Me_2_]}] (**7**) generated from **5** (a) or **6** (b) and its comparison with its *trans* isomer (c).

Pyridinium chloride of **5** can be abstracted from the iridium coordination sphere, in acetone, with a KOH solution in water. The abstraction produces the immediate dimerization of the [IrCl(**3b**)_2_] moiety to afford the very insoluble *cis*-[Ir(μ-Cl){κ^2^-*C,N*-[C_6_MeH_3_-py]}_2_]_2_ (**6**) as a yellow solid in 89% yield. Its binuclear nature is strongly supported by the MALDI-TOF spectrum of the yellow solid in dichloromethane, which shows a peak for *m*/*z* of 1093.2, corresponding to a C_48_H_40_ClIr_2_N_4_ ([M_2_ – Cl]^+^) fragment. The extraction of the pyridinium chloride also takes place in dimethyl sulfoxide. In this solvent, complex **5** generates the solvate derivative *cis*-[IrCl{κ^2^-*C,N*-[C_6_MeH_3_-py]}_2_{κ^1^-*S*-[S(O)Me_2_]} (**7**). As is typical for [Ir(μ-Cl)(**3b**)_2_]_2_ dimers,^16k^ this species is also formed from the cleavage of the chloride bridges of **6**, which occurs as a result of its solubilization in this highly coordinating solvent. Figure 3 shows the overwhelming difference between the spectra of **7**, generated from **5** and **6**, and that of its *trans* isomer resulting from the dissolution of the usual *trans*-[Ir(μ-Cl){κ^2^-*C,N*-[C_6_H_3_Me-py]}_2_]_2_ dimer in dimethyl sulfoxide. The resonances corresponding to the methyl substituents of the phenyl groups are especially worth noting. The spectra of **7** (Figure 3a,b) show markedly separated signals, at 2.44 and 1.89 ppm, compared to the analogous singlets of the spectrum of the *tran*s-pyridyl isomer (Figure 3c). In the low field region, between 10.2 and 9.6 ppm, the difference is also noticeable; while the spectra of **7** contain one doublet, the spectrum of the *trans* isomer contains two.

The low solubility of **6** in the usual organic solvents makes it difficult to apply as a starting compound for the preparation of heteroleptic emitters of the class [**3b**+**3b**+**3b′**] with the pyridyl groups of the **3b** ligands arranged in *cis*. On the other hand, hydroxide complexes provide great versatility in organometallic synthesis due to the presence of the internal OH^–^ base, which facilitates σ-bond heterolytic activation reactions,^30,31^ as shown in Scheme 1. This versatility inspired us to transform complex **6** into a related hydroxide–dimer complex. We first tried a method similar to the one shown in Scheme 1. However, all attempts were unsuccessful, and complex mixtures of solvate species were formed. In view of such a situation, we decided to use a silver salt with a strongly coordinating anion, as a chloride extracting agent, and perform the transformation in one-pot to avoid the formation of five-coordinate intermediate species. Thus, we selected silver acetate as the chloride extraction agent and CsOH, which is more soluble than other alkali hydroxides, as the OH^–^ source. As expected, the stirring of **6** with 5.0 equiv of the base and 1.0 equiv of the salt in dichloromethane at room temperature for 60 h yields the desired *cis*-[Ir(μ-OH){κ^2^-*C,N*-[C_6_MeH_3_-py]}_2_]_2_ (**8**) dimer. This complex was isolated as an orange solid in 56% yield. Figure S93 compares the ^1^H NMR spectra in dichloromethane-*d*_2_ at room temperature of **8** and of *trans*-[Ir(μ-OH){κ^2^-*C,N*-[C_6_MeH_3_-py]}_2_]_2_ isomer containing pyridyl groups located in *trans* positions, which was prepared as shown in Scheme 1. The spectrum of **8** is more complex, since it reveals the presence of inequivalent chelates and equivalent hydroxide bridges, consistent with the isomer drawn in Scheme 5. The resonance chemical shift corresponding to the bridges, −0.45 ppm, which is shifted about 1 ppm downfield with respect to the resonance due to the hydroxide groups of *trans*-[Ir(μ-OH){κ^2^-*C,N*-[C_6_MeH_3_-py]}_2_]_2_ (−1.53 ppm) is worth mentioning. The dimeric nature of **8** is unquestionable, since the HRMS spectrum (electrospray ionization) in dichloromethane shows a peak for *m*/*z* of 1073.2501, corresponding to the fragment C_48_H_41_Ir_2_N_4_O ([M_2_ – OH]^+^). Complex *trans*-[Ir(μ-OH){κ^2^-*C,N*-[C_6_H_3_Me-py]}_2_]_2_ is more stable than **8**. Furthermore, the activation energy for the rearrangement of the pyridyl groups appears to be low; in toluene, at 100 °C, dimer **8** undergoes the transformation into *trans*-[Ir(μ-OH){κ^2^-*C,N*-[C_6_MeH_3_-py]}_2_]_2_. The isomerization is quantitative after 12 h. This means that complex **8** is only a useful starting compound to prepare [**3b**+**3b**+**3b′**] emitters with the pyridyl groups *cis* disposed in low activation energy procedures, that is, when they require low reaction temperatures.

### Procedures for the Preparation of [**3b**+**3b**+**3b′**] Emitters with a *cis*-Pyridyl Arrangement

Complexes **5** and **8** are suitable compounds when it is desired to maintain the *cis* arrangement of the pyridyl groups, although the use of **8** is limited to reactions that require temperatures lower than 100 °C.

**Scheme 6:**
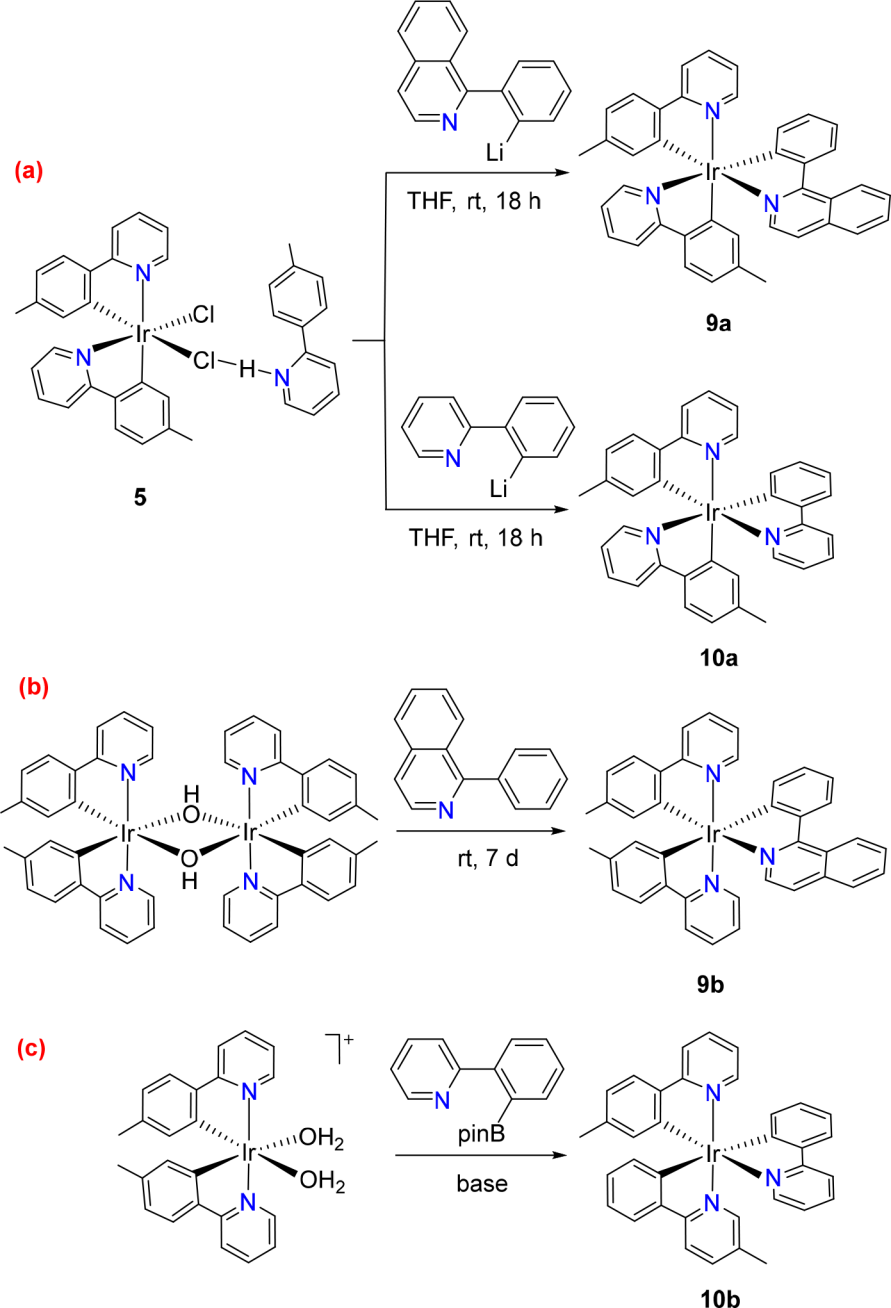
Synthesis of [**3b**+**3b**+**3b′**] Emitters

We chose the mononuclear complex **5** to obtain the desired emitters [**3b**+**3b**+**3b′**], which carry a **3b′** ligand of the orthometalated 2-phenylpyridine type. The selection was motivated by the usual need to employ moderate temperatures to promote the activation of the *ortho*-CH bond of the heterocycle substituent. Furthermore, the presence of the chloride ligand in the starting complex makes it advisable to use a transmetalation agent to introduce **3b′**. In this context, lithium is particularly suitable due to the accessibility of its reagents, which can be generated *in situ* by adding solutions of ^n^BuLi, in hexane, to the appropriate bromo precursor. To validate the method, we selected 1-(2-bromophenyl)isoquinoline and 2-(2-bromophenyl)pyridine as **3b′** ligand precursors. The addition of 1.5 equiv of ^n^BuLi in hexane to solutions of these halogenated derivatives in tetrahydrofuran at −78 °C and subsequent treatment of the resulting solutions with 0.5 equiv of **5** at room temperature selectively led to complexes *cis*-[Ir{κ^2^-*C,N*-[C_6_MeH_3_-py]}_2_{κ^2^-*C,N*-[C_6_H_4_–Isoqui]}] (**9a**) and *cis*-[Ir{κ^2^-*C,N*-[C_6_MeH_3_-py]}_2_{κ^2^-*C,N*-[C_6_H_4_-py]}] (**10a**), as desired (Scheme 6a).

**Figure 4:**
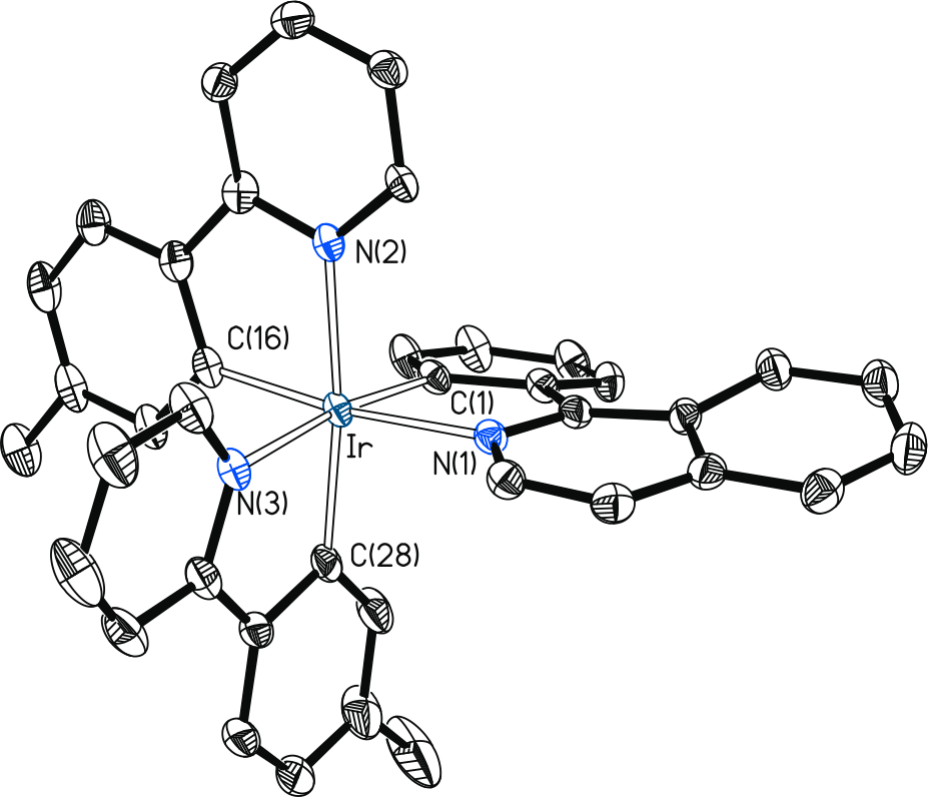
Molecular diagram of complex **9a** (displacement ellipsoids shown at 50% probability). All hydrogen atoms are omitted for clarity. Selected bond distances (Å) and angles (deg): Ir–C(1) = 2.001(3), Ir–C(16) = 2.017(3), Ir–C(28) = 2.007(3), Ir–N(1) = 2.110(3), Ir–N(2) = 2.127(3), Ir–N(3) = 2.135(3); C(1)–Ir–N(1) = 78.53(13), C(16)–Ir–N(2) = 79.55(13), C(28)–Ir–N(3) = 79.22(14), C(28)–Ir–N(2) = 172.81(12), C(16)–Ir–N(1) = 172.04(12), C(1)–Ir–N(3) = 172.42(13).

Isoquinolinyl complex **9a** was isolated as red crystals in 35% yield after purification of the reaction crude by silica column chromatography. Figure 4 gives a view of the molecule. The structure confirms the *fac* arrangement of carbon and nitrogen atoms in an octahedral environment.

**Figure 5:**
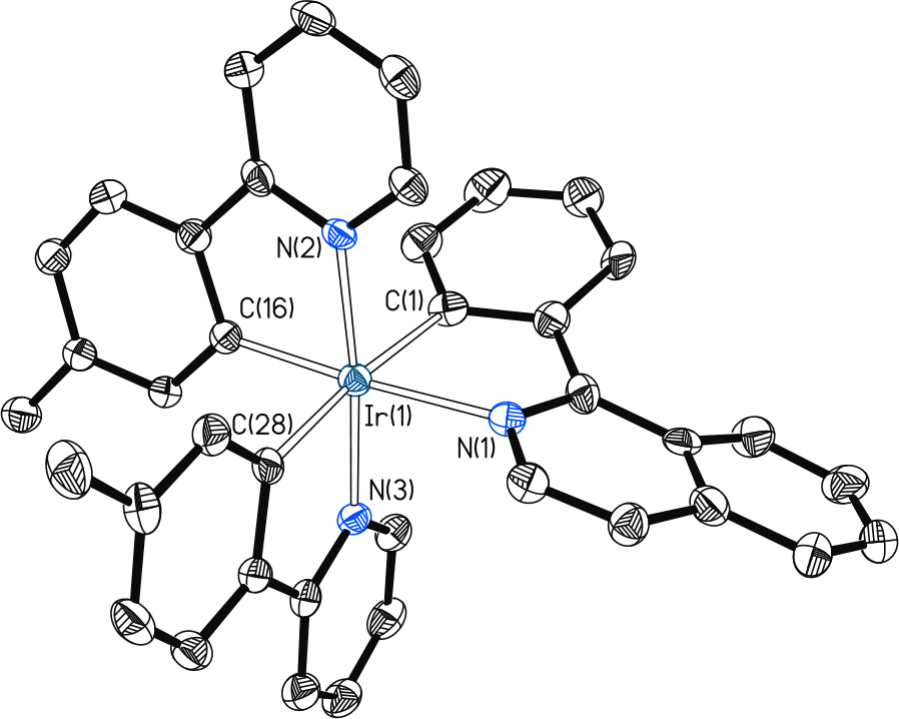
Molecular diagram of one of the two chemically equivalent but crystallographically independent molecules of complex **9b** (displacement ellipsoids shown at 50% probability). All hydrogen atoms are omitted for clarity. Selected bond distances (Å) and angles (deg): Ir(1)–C(1) = 2.086(7), 2.091(7), Ir(1)–C(16) = 1.996(7), 2.002(7), Ir(1)–C(28) = 2.065(7), 2.054(9), Ir(1)–N(1) = 2.150(6), 2.139(7), Ir(1)–N(2) = 2.035(6), 2.041(7), Ir(1)–N(3) = 2.043(6), 2.046(6); N(2)–Ir(1)–N(3) = 174.2(2), 172.4(3), C(28)–Ir(1)–C(1) = 174.0(3), 172.5(3), C(16)–Ir(1)–N(1) = 174.9(3), 171.2(3), C(1)–Ir(1)–N(1) = 77.3(3), 77.0(3), C(16)–Ir(1)–N(2) = 80.3(3), 80.7(3), N(3)–Ir(1)–C(28) = 79.7(3), 79.8(4).

DFT calculations (B3LYPG-D3//SDD(f)-6-31G**) reveal that complex **9a** is 8.1 kcal mol^–1^ more stable than its isomer **9b** with pyridyl groups arranged in the *trans* position (Figure S1). Isomer **9b** was prepared as a red solid in 22% yield by treating a suspension of the dimer *trans*-[Ir(μ-OH){κ^2^-*C,N*-[C_6_MeH_3_-py]}_2_]_2_ in dichloromethane with 1.0 equiv of 2-phenylisoquinoline at room temperature for 7 days (Scheme 6b). The reaction implies the *ortho*-CH bond heterolytic activation of the phenyl substituent assisted by the heterocycle and promoted by the hydroxide bridges that act as internal base. The *trans* arrangement of the pyridyl groups (N(2)–Ir(1)–N(3) = 174.2(2)° and 172.4(3)°) was confirmed by X-ray diffraction analysis. Figure 5 shows one of the two chemically equivalent but crystallographically independent molecules of the asymmetric unit.

Complex **10a** was isolated in 56% yield. It had been previously generated by photoisomerization of a *mer*-pyridyl isomer **10b**, which bears the pyridyl groups of orthometalated 2-*p*-tolylpyridine arranged in *trans*. DFT calculations reveal that the latter is 7.6 kcal mol^–1^ less stable than **10a** (Figure S2). Isomer **10b** was prepared for first time by reaction of the dimer *trans*-[Ir(μ-Cl){κ^2^-*C,N*-[C_6_MeH_3_-py]}_2_]_2_ with 2-phenylpyridyne in glycerol at 150 °C using K_2_CO_3_ as external base for promoting the *ortho*-CH bond heterolytic activation of the phenyl group.^19^ However, a more appropriate method to obtain it is by transmetalation involving the *cis*-bis(aquo)iridium(III) cation [Ir{κ^2^-*C,N*-[C_6_MeH_3_-py]}_2_(H_2_O)_2_]^+^ and the boronated aryl-proligand also in a base-assisted reaction (Scheme 6c).^32^

**Scheme 7:**
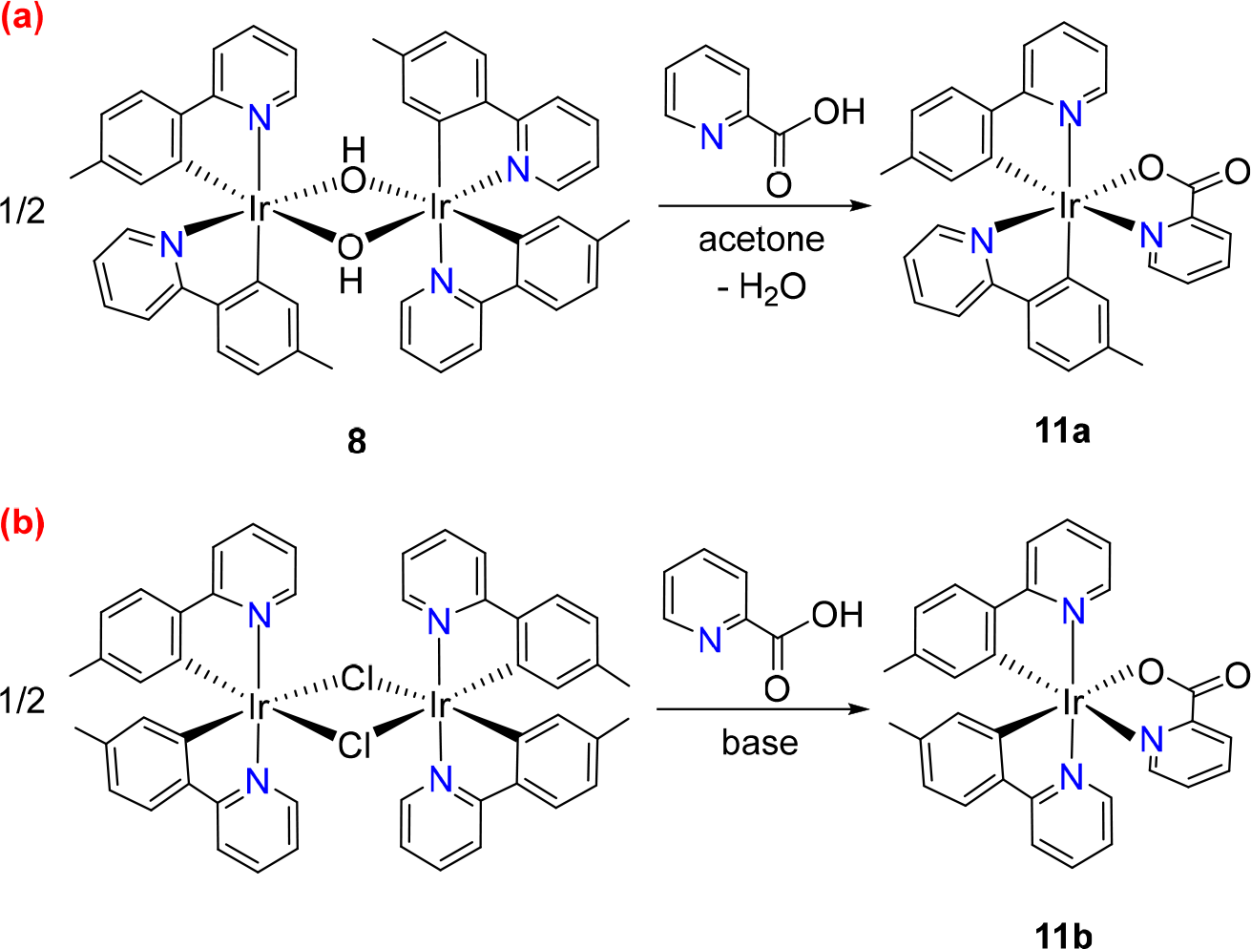
Preparation of [**3b**+**3b**+**3b′**]-Picolinate Isomers

The dimer of hydroxide bridges **8** is a useful starting complex for preparing [**3b**+**3b**+**3b′**] derivatives with pyridyl groups *cis* arranged when the precursor molecule of the **3b′** ligand has a fairly acidic hydrogen atom, suitable to be abstracted by hydroxide groups. An example of this type of molecule is 2-picolinic acid. Although its deprotonation generates an asymmetric anion, which can form *fac*- and *mer*-pyridyl isomers while maintaining the *cis* arrangement of the pyridyl groups of the starting dimer, the treatment of **8** with 1.0 equiv of the acid in acetone at room temperature for 14 h selectively leads to the *fac*-pyridyl isomer Ir{κ^2^-*C,N*-[C_6_MeH_3_-py]}_2_{κ^2^-*O,N*-[OC(O)-py]} (**11a**). This compound was isolated as an analytically pure yellow solid in 56% yield without further purification (Scheme 7a).

**Figure 6:**
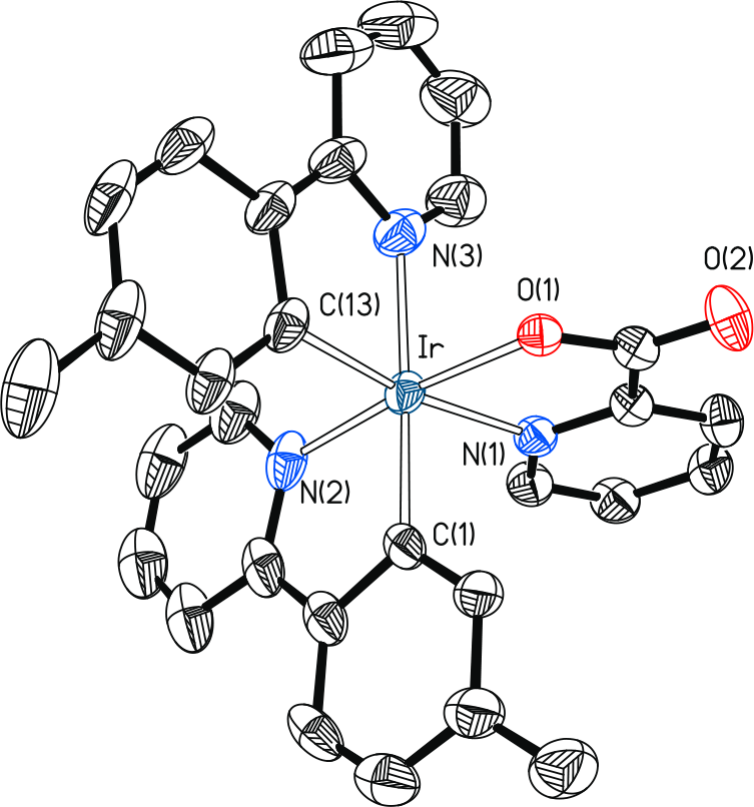
Molecular diagram of complex **11a** (displacement ellipsoids shown at 50% probability). All hydrogen atoms are omitted for clarity. Selected bond distances (Å) and angles (deg): Ir–C(1) = 2.008(4), Ir–C(13) = 2.010(4), Ir–N(1) = 2.124(3), Ir–N(2) = 2.028(4), Ir–N(3) = 2.137(4), Ir–O(1) = 2.068(3); C(1)–Ir–N(2) = 80.04(19), C(13)–Ir–N(3) = 79.6(2), O(1)–Ir–N(1) = 78.53(12), C(1)–Ir–N(3) = 176.10(16), C(13)–Ir–N(1) = 170.74(17), N(2)–Ir–O(1) = 174.13(15).

The *fac* arrangement of the pyridyl groups was confirmed by X-ray diffraction analysis. Figure 6 gives a view of the structure of the molecule. The isolation of **11a** with the pyridyl groups of the orthometalated 2-(*p*-tolyl)pyridine ligands situated mutually *cis* is noticeable, since DFT calculations revealed that is 4.3 kcal mol^–1^ less stable than the isomer **11b** containing such pyridyl groups in the *trans* position (Figure S3). Complex **11b** has been previously prepared by reacting dimer *trans*-[Ir(μ-Cl){κ^2^-*C,N*-[C_6_MeH_3_-py]}_2_]_2_ with 2-picolinic acid in the presence of base (Scheme 7b).^16a,16c^

**Scheme 8:**
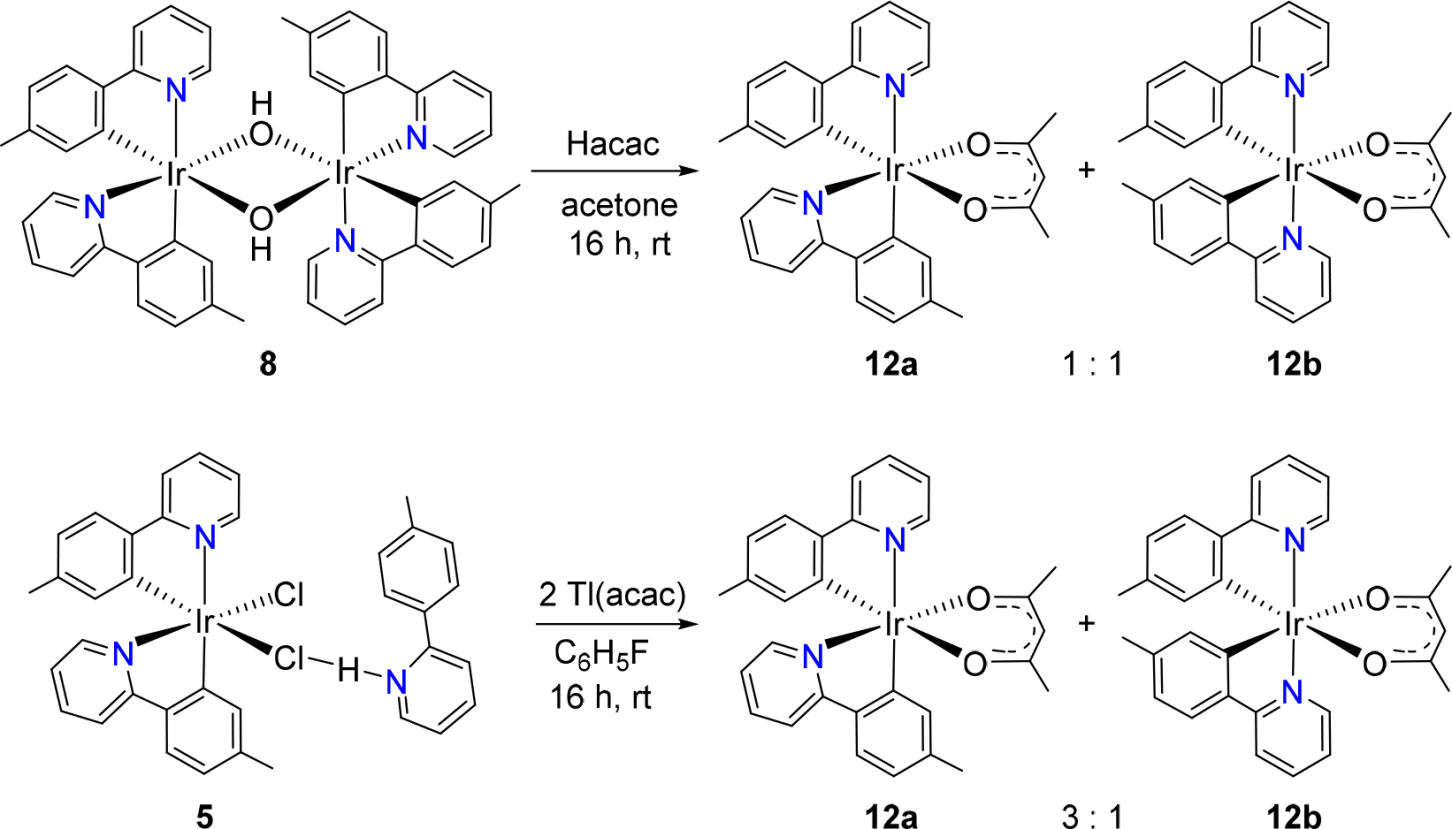
Preparation of [**3b**+**3b**+**3b′**]-acac Isomers

The CH_2_ group of acetylacetone (Hacac) has acidic properties like 2-picolinic acid. Thus, dimer **8** should also be suitable in principle to give the complex *cis*-Ir{κ^2^-*C,N*-[C_6_MeH_3_-py]}_2_{κ^2^-*O,O*-[acac]} (**12a**) with *cis*-pyridyl groups by a procedure similar to that employed to generate **11a** using Hacac instead of 2-picolinic acid. As for **11**, DFT calculations indicate that said isomer is 4.0 kcal mol^–1^ less stable than the isomer that carries the heterocycles in the *trans* position, **12b** (Figure S4). Unfortunately, the activation energy for the κ^2^-*O,O*-coordination of the acac ligand, generated from the protonation of hydroxide bridges, appears to be comparable to the activation energy for the rearrangement from *cis*-pyridyl to *trans*-pyridyl in mononuclear fragments. Consequently, the addition of 1.0 equiv of Hacac to solutions of **8** in acetone at room temperature provides a mixture of the desired complex **12a** and its isomer **12b** in a molar ratio of about 1:1 (Scheme 8). The low stereoselectivity of the reaction could tentatively be related to the ability of the acac ligand to act as κ^1^-*C*^3^-monodentate, allowing five-coordinate mononuclear intermediates capable of undergoing the *cis*-pyridyl to *trans*-pyridyl rearrangement.^33^ A more appropriate procedure to obtain **12a** is the treatment of solutions of **5** in fluorobenzene with 2.1 equiv of Tl(acac) at room temperature for 16 h. Under these conditions, the amount of **12a** increases significantly; the reaction crude shows a **12a**:**12b** molar ratio of 3:1. Isomer **12a** was separated from the mixture by column chromatography on basic alumina and isolated as a yellow solid in 40% yield.

### Photophysical and Electrochemical Properties: Comparison between Isomers **a** and **b**

In order to obtain information on the influence of stereochemistry on the absorption and emission characteristics of iridium(III) heteroleptic emitters of type [**3b**+**3b**+**3b′**] as well as on the ease of oxidizing them, we comparatively studied the absorption and emission and electrochemical properties of the four pairs of **a** and **b** isomers discussed above. In addition, the same properties of homoleptic emitter **3** were also obtained, since this compound has not been previously reported, and therefore, its properties are not known.

The UV–vis spectra of **3** and the **a** and **b** isomers of **9**–**12** in 2-methyltetrahydrofuran (2-MeTHF) at room temperature are as expected for six-coordinate iridium(III) species (Figures S5a–S13a). They show bands with intensities that depend on their position. Thus, the spectra can be divided into three regions: very strong absorptions lie below 320 nm (ε ≈ 50 000–20 000 M^–1^ cm^–1^), strong bands are observed between 350 and 450 nm (ε ≈ 15 000–500 M^–1^ cm^–1^), and weak absorptions appear at energies below 450 nm (ε < 4000 M^–1^ cm^–1^). The spectra were calculated in tetrahydrofuran by TD-DFT (B3LYP-D3//SDD(f)/6-31G**); Figures S5b–S13b show those obtained, whereas Tables S1–S9 collect the transitions that contribute to the bands of each one of them. In addition, Figures S14–S22 offer views of the most relevant orbitals and Tables S10–S18 summarize the fragments involved in these orbitals. Table 1 lists some selected experimental absorptions assigned on these bases.

**Table 1:** Selected Calculated (TD-DFT in THF) and Experimental UV–Vis Absorptions for **3** and **a** and **b** isomers of **9**–**12** (in 2-MeTHF) and Their Major Contributions

λ exp (nm)	ε (M^–1^ cm^–1^)	exc. energy (nm)	oscilator strength, *f*	transition	character of the transition
Complex **3**					
286	54100	282	0.5461	HOMO-5 → LUMO (67%)	(**3b** + **3b** + **3b** → **3b** + **3b** + **3b**)
379	13500	384	0.0641	HOMO-2 → LUMO (93%)	(Ir + **3b** + **3b** → **3b** + **3b** + **3b**)
402	8800	408 (S_1_)	0.0149	HOMO → LUMO (97%)	(Ir + **3b** + **3b** + **3b** → **3b** + **3b** + **3b**)
453	3000	454 (T_1_)	0	HOMO → LUMO (60%)	(Ir + **3b** + **3b** + **3b** → **3b** + **3b** + **3b**)
Complex **9a**					
285	42500	293	0.1866	HOMO-4 → LUMO+2 (79%)	(**3b** + **3b** → **3b** + **3b**)
382	10100	384	0.0474	HOMO-1 → LUMO+1 (77%)	(Ir + **3b** → **3b**)
434	7100	440	0.1434	HOMO-2 → LUMO (93%)	(Ir + **3b** + **3b′** → **3b′**)
481	3300	486 (S_1_)	0.0119	HOMO → LUMO (94%)	(Ir + **3b** + **3b** + **3b** → **3b′**)
567	500	565 (T_1_)	0	HOMO-3 → LUMO (17%)	(Ir + **3b** + **3b′** → **3b′**)
				HOMO → LUMO (59%)	(Ir + **3b** + **3b** + **3b** → **3b′**)
Complex **9b**					
280	43700	281	0.0406	HOMO-7 → LUMO+1 (40%)	(**3b** + **3b** → **3b**)
				HOMO-4 → LUMO+3 (33%)	(**3b** + **3b** → **3b** + **3b′**)
383	9400	387	0.0819	HOMO-3 → LUMO (67%)	(Ir + **3b** + **3b′** → **3b′**)
				HOMO-2 → LUMO (23%)	(Ir + **3b** + **3b** + **3b′** → **3b′**)
452	4000	444 (S_1_)	0.0625	HOMO-1 → LUMO (97%)	(Ir + **3b′** → **3b′**)
504	1200	506 (T_1_)	0	HOMO → LUMO (84%)	(Ir + **3b** + **3b** → **3b′**)
Complex **10a**					
284	27920	285	0.2592	HOMO-5 → LUMO (87%)	(**3b′** → **3b** + **3b** + **3b′**)
376	8200	387	0.0648	HOMO-2 → LUMO (52%)	(Ir + **3b** + **3b′** → **3b** + **3b** + **3b′**)
				HOMO-1 → LUMO (39%)	(Ir + **3b** + **3b** → **3b** + **3b** + **3b′**)
408	5400	413 (S_1_)	0.0136	HOMO → LUMO (95%)	(Ir + **3b** + **3b** + **3b′** → **3b** + **3b** + **3b′**)
449	2140	458 (T_1_)	0	HOMO-2→ LUMO+1 (11%)	(Ir + **3b** + **3b′** → **3b** + **3b** + **3b′**)
				HOMO → LUMO (60%)	(Ir + **3b** + **3b** + **3b′** → **3b** + **3b** + **3b′**)
Complex **10b**					
276	62200	274	0.0466	HOMO-7 → LUMO+1 (26%)	(**3b** + **3b** → **3b** + **3b′**)
				HOMO-7 → LUMO+2 (48%)	(**3b** + **3b** → **3b**)
384	9200	384	0.0225	HOMO-1 → LUMO+1 (96%)	(Ir + **3b** + **3b′** → **3b** + **3b′**)
422	5900	430 (S_1_)	0.0427	HOMO → LUMO (81%)	(Ir + **3b** + **3b** → **3b** + **3b′**)
				HOMO → LUMO+1 (16%)	(Ir + **3b** + **3b** → **3b** + **3b′**)
463	3800	460 (T_1_)	0	HOMO → LUMO+1 (24%)	(Ir + **3b** + **3b** → **3b** + **3b′**)
				HOMO → LUMO+2 (47%)	(Ir + **3b** + **3b** → **3b**)
Complex **11a**					
283	60400	287	0.0879	HOMO-4 → LUMO+2 (73%)	(**3b** + **3b** → **3b**)
350	10300	350	0.0553	HOMO-2 → LUMO+2 (15%)	(Ir + **3b′** → **3b**)
				HOMO → LUMO+3 (73%)	(Ir + **3b** + **3b** → **3b** + **3b** + **3b′**)
406	4800	412 (S_1_)	0.0195	HOMO → LUMO (95%)	(Ir + **3b** + **3b** → **3b′**)
466	700	465 (T_1_)	0	HOMO → LUMO (14%)	(Ir + **3b** + **3b** → **3b′**)
				HOMO → LUMO+1 (57%)	(Ir + **3b** + **3b** → **3b** + **3b** + **3b′**)
Complex **11b**					
269	23860	264	0.017	HOMO-6 → LUMO+3 (15%)	(**3b** + **3b** + **3b′** → **3b** + **3b** + **3b′**)
				HOMO-5 → LUMO+3 (52%)	(**3b** + **3b** + **3b′** → **3b** + **3b** + **3b′**)
353	4620	359	0.0266	HOMO-1 → LUMO (93%)	(Ir + **3b** + **3b′** → **3b** + **3b** + **3b′**)
419	2440	421 (S_1_)	0.0413	HOMO → LUMO+1 (97%)	(Ir + **3b** + **3b** → **3b** + **3b** + **3b′**)
480	600	470 (T_1_)	0	HOMO → LUMO (18%)	(Ir + **3b** + **3b** → **3b** + **3b** + **3b′**)
				HOMO → LUMO+1 (58%)	(Ir + **3b** + **3b** → **3b** + **3b** + **3b′**)
Complex **12a**					
298	32000	299	0.1281	HOMO-3 → LUMO+1 (81%)	(**3b** + **3b** → **3b** + **3b**)
376	7200	379	0.0482	HOMO-1 → LUMO (43%)	(Ir + **3b** + **3b** + **3b′** → **3b** + **3b**)
				HOMO-1 → LUMO+1 (43%)	(Ir + **3b** + **3b** + **3b′** → **3b** + **3b**)
404	5300	419 (S_1_)	0.0173	HOMO → LUMO (90%)	(Ir + **3b** + **3b** → **3b** + **3b**)
471	1200	474 (T_1_)	0	HOMO → LUMO (67%)	(Ir + **3b** + **3b** → **3b** + **3b**)
				HOMO → LUMO+1 (12%)	(Ir + **3b** + **3b** → **3b** + **3b**)
Complex **12b**					
275	45600	276	0.032	HOMO-7 → LUMO+2 (27%)	(**3b** + **3b** + **3b′** → **3b′**)
				HOMO-3 → LUMO+2 (44%)	(**3b** + **3b** → **3b′**)
368	7000	374	0.042	HOMO-1 → LUMO (11%)	(Ir + **3b′** → **3b** + **3b**)
				HOMO-1 → LUMO+1 (83%)	(Ir + **3b′** → **3b** + **3b**)
423	3600	430 (S_1_)	0.0527	HOMO → LUMO (91%)	(Ir + **3b** + **3b** → **3b** + **3b**)
461	3200	475 (T_1_)	0	HOMO-3 → LUMO+1 (10%)	(**3b** + **3b** → **3b** + **3b**)
				HOMO → LUMO (51%)	(Ir + **3b** + **3b** → **3b** + **3b**)
				HOMO → LUMO+1 (28%)	(Ir + **3b** + **3b** → **3b** + **3b**)

The higher energy bands are due to inter- and intraligand ^1^π–π* transitions. The absorptions in the region of 350–450 nm correspond mainly to spin-allowed charge transfers from the iridium center to the heterocycles combined with transitions from the orthometalated phenyl groups to the heterocycles. The tails after 450 nm imply formal spin-forbidden HOMO–LUMO transitions, which result from the large spin–orbit coupling provided by iridium. The HOMO of the homoleptic complex **3** is delocalized in the metal center (53%) and the orthometalated aryl groups that contribute with similar percentages (15–17% each), while the LUMO is distributed among the three heterocycles also in a homogeneous way (32–34%). The HOMO of the isoquinolyl derivatives **9a** and **9b** is not significantly different from that of **3**; it is delocalized on the iridium atom (49% and 41%, respectively) and the orthometalated aryl groups. However, LUMO is located almost exclusively on the isoquinolyl group, 94% in both cases. The tris(pyridyl) derivatives **10a** and **10b** present a situation similar to **3**, although the metal contribution to the HOMO is higher for the *fac*-pyridyl isomer than for the *mer*-pyridyl isomer (52% versus 42%) and the distribution of this orbital among the orthometalated groups is also more homogeneous for the first of them (14–18% versus 7–32%). Picolinate compounds **11a** and **11b** resemble isoquinolyl emitters **9**; HOMO is delocalized in the metal center (≈44%) and homogeneously between the orthometalated *p*-tolyl groups (25–27% each), while LUMO is mostly centered on the pyridyl group of the picolinate ligand (≈80%). Unlike picolinate, the acac ligand has minimal participation in the frontier orbitals of complexes **12a** and **12b**; the HOMO is like that of the picolinate counterparts, while the LUMO is distributed among the pyridyl groups.

The calculated HOMO–LUMO gaps for **9a** and **9b** are consistent with the overwhelming participation of the isoquinolyl group in the LUMO. This group significantly stabilizes the LUMO of both isomers, compared to the LUMO of the other compounds, which gives rise to smaller values. In all cases, the HOMO–LUMO gap for the *cis*-pyridyl **a** derivative is between 0.15 and 0.10 eV larger than the HOMO–LUMO gap for the *trans*-pyridyl **b** isomer. This is, however, a consequence of the greater stability of the HOMO of **a** complexes with respect to the HOMO of the respective **b** isomers, since the LUMO of both isomers has practically identical energy. For the nine complexes, the energy calculated for the HOMO and that obtained experimentally from the value of the corresponding redox anodic potential, versus Fc/Fc^+^, in dichloromethane (Figure S23) are in excellent agreement (Table 2). So, the calculated and experimental values reveal that the *trans*-pyridyl **b** isomers are easier to oxidize than the *cis*-pyridyl **a** isomers, consistent with the previously observed greater ease to oxidize the *mer*-homoleptic tris(pyridyl) derivatives with respect to their *fac*-isomers.^8^

**Table 2:** Electrochemical and DFT Molecular Orbital Energy Data for **3** and **a** and **b** Isomers of **9**–**12**

		obs (eV)	calcd (eV)		
complex	*E*_1/2_^ox^ vs Fc/Fc^+^ (V)^a^	HOMO^b^	HOMO	LUMO	HLG^c^
**3**	0.05	–4.85	–4.91	–1.12	3.79
**9a**	0.19	–4.99	–4.97	–1.72	3.25
**9b**	0.11	–4.91	–4.86	–1.74	3.12
**10a**	0.20	–5.00	–4.99	–1.23	3.76
**10b**	0.09	–4.89	–4.86	–1.25	3.61
**11a**	0.58	–5.38	–5.19	–1.45	3.74
**11b**	0.47	–5.27	–5.05	–1.41	3.64
**12a**	0.40	–5.20	–5.01	–1.25	3.76
**12b**	0.38	–5.18	–4.93	–1.27	3.66

a Measured under argon in dichloromethane/[Bu_4_N]PF_6_ (0.1 M) vs Fc/Fc^+^.

b HOMO = −[*E*_1/2_^ox^ vs Fc/Fc + 4.8] eV.

c HGL = LUMO – HOMO.

Emission measurements were performed upon photoexcitation, under three different conditions: on a 5 wt % poly(methyl methacrylate) (PMMA) doped film at room temperature, on 2-MeTHF at room temperature, and on 2-MeTHF at 77 K (Figures S24–S50). Table 3 lists the most notable features. Homoleptic complex **3** is a very efficient blue-green emitter (488–526 nm), reaching 100% quantum yield on both PMMA film and 2-MeTHF at room temperature and lifetimes in the range of 1.3–3.0 μs. Complexes **9a** and **9b** emit in the orange region between 574 and 622 nm, while the **a** and **b** isomers of **10**–**12** are green emitters (483–544 nm) in agreement with larger HOMO–LUMO gaps.

**Table 3:** Photophysical Data for **3** and **a** and **b** isomers of **9**–**12**

calcd λ_em_ (nm)	media (*T*/K)	λ_em_ (nm)	τ (μs)^a^	fwhm (nm)^b^	Φ_L_	*k*_r_ (s^–1^)^c^	*k*_nr_ (s^–1^)^d^	*k*_r_/*k*_nr_
Complex **3**								
497	PMMA (298)	502	1.3	70	∼1	7.7 × 10^5^		
	2-MeTHF (298)	500	1.5	65	∼1	6.7 × 10^5^		
	2-MeTHF (77)	488, 526	3.0					
Complex **9a**								
640	PMMA (298)	628	1.2	109	0.60	5.0 × 10^5^	3.3 × 10^5^	1.51
	2-MeTHF (298)	632	1.3	106	0.51	3.9 × 10^5^	3.8 × 10^5^	1.02
	2-MeTHF (77)	602, 654	2.9					
Complex **9b**								
624	PMMA (298)	622	1.5 (1.95, 54.4%; 0.87, 45.6%)	119	0.50	3.3 × 10^5^	3.3 × 10^5^	1.00
	2-MeTHF (298)	644	0.3	133	0.12	4.0 × 10^5^	2.9 × 10^6^	0.14
	2-MeTHF (77)	574, 624	3.3					
Complex **10a**								
504	PMMA (298)	512	1.28 (1.33, 94.4%; 0.44, 5.6%)	74	∼1	7.8 × 10^5^		
	2-MeTHF (298)	512	1.6	72	0.98	6.1 × 10^5^	1.3 × 10^4^	46.92
	2-MeTHF (77)	492, 530	4.0 (5.1, 37.9%; 3.3, 62.1%)					
Complex **10b**								
543	PMMA (298)	544	1.0 (1.1, 75.5%; 0.5, 24.5%)	113	0.70	7.0 × 10^5^	3.0 × 10^5^	2.33
	2-MeTHF (298)	542	0.8 (1.6, 44.8%; 0.1, 55.2%)	119	0.09	1.1 × 10^5^	1.1 × 10^6^	0.10
	2-MeTHF (77)	532	5.3 (6.1, 64.5%; 3.8, 35.5%)					
Complex **11a**								
510	PMMA (298)	510	1.1 (1.5, 50%; 0.6, 50%)	88	0.28	2.2 × 10^5^	5.5 × 10^5^	0.40
	2-MeTHF (298)	518	1.0	84	0.28	2.8 × 10^5^	7.2 × 10^5^	0.39
	2-MeTHF (77)	492, 534	4.4					
Complex **11b**								
515	PMMA (298)	502	1.5 (1.6, 86.6%; 0.7, 13.4%)	66	0.84	5.6 × 10^5^	1.1 × 10^5^	5.09
	2-MeTHF (298)	504	1.7	67	0.85	2.0 × 10^5^	0.9 × 10^5^	2.22
	2-MeTHF (77)	483, 522	3.8					
Complex **12a**								
506	PMMA (298)	523	0.94 (0.61, 76.3%; 2.01, 23.7%)	74	0.30	3.2 × 10^5^	7.4 × 10^5^	0.43
	2-MeTHF (298)	517	0.83 (0.05, 50.0%; 1.62, 50.0%)	65	0.30	3.6 × 10^5^	8.4 × 10^5^	0.43
	2-MeTHF (77)	504, 541	4.7					
Complex **12b**								
520	PMMA (298)	519	1.3 (1.41, 80.7%; 0.72, 19.2%)	65	∼1	7.7 × 10^5^		
	2-MeTHF (298)	517	1.6	62	0.79	4.6 × 10^5^	1.2 × 10^5^	3.83
	2-MeTHF (77)	501, 539	4.59 (6.52, 24.4%; 3.96, 75.6%)					

a Relative amplitudes (%) are given in parentheses for biexponential decays.

b Full width at half-maximum.

c Radiative rate constants calculated according to *k*_r_ = Φ_L_/τ.

d Radiationless deactivation rate constants calculated according to *k*_nr_ = (1 – Φ_L_)/τ. For biexponential decays, the amplitude-weighted average lifetimes were used.

**Figure 7:**
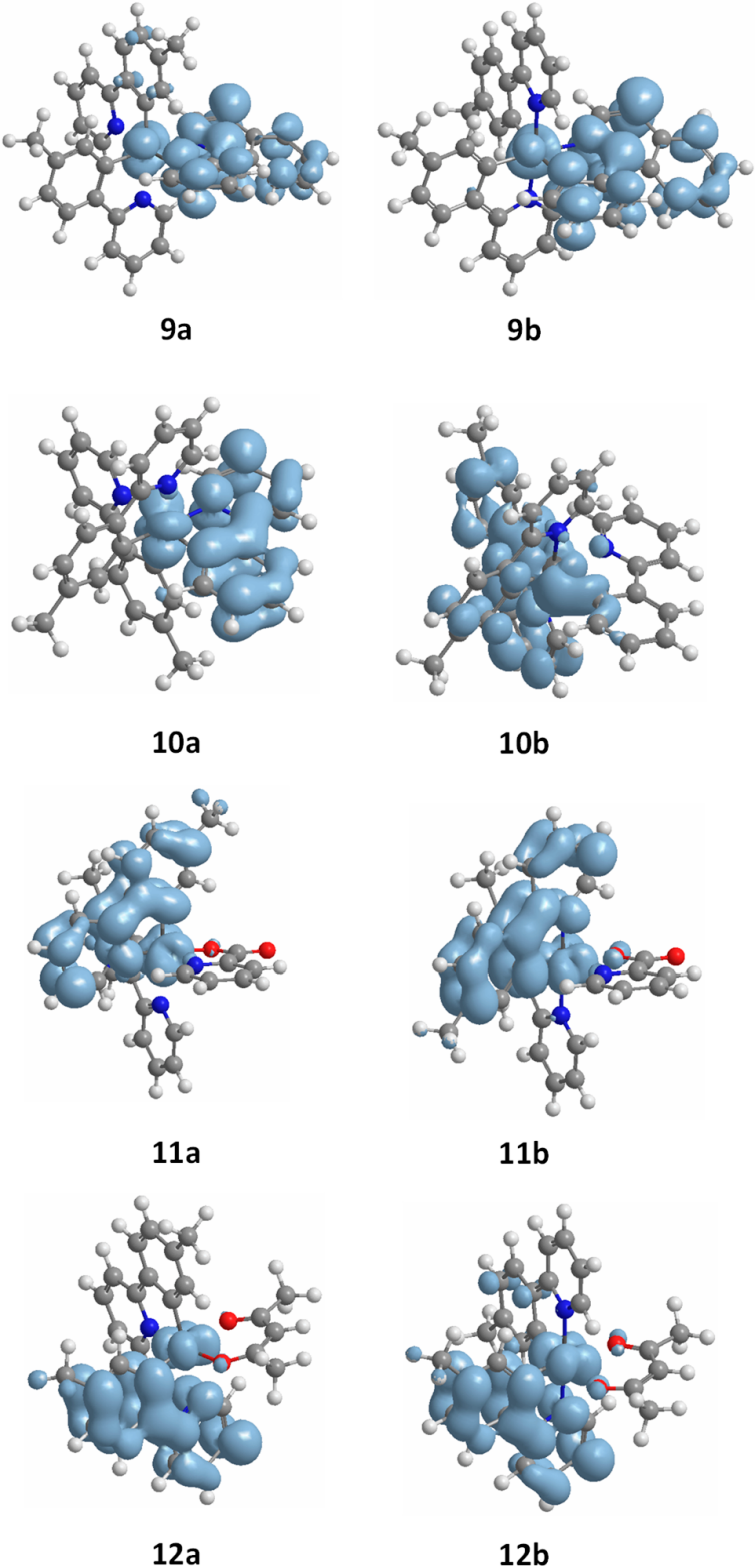
DFT calculated spin density for the T_1_ states of isomers **a** and **b** of complexes **9**–**12** at 0.002 au contour level.

The emissions start from the respective excited state T_1_, as confirmed by the excellent agreement between the wavelengths of the maxima, in 2-MeTHF at room temperature and the values calculated for the energy differences between the optimized state T_1_ and the ground state singlet S_0_, considering tetrahydrofuran as solvent. Figure 7 shows the calculated spin density distribution for the T_1_ states at the minimum energy geometry of the **a** and **b** isomers of **9**–**12**. There is no significant difference in their distribution between the isomers of **9**, **11**, and **12**; it is on the same fragments of both isomers, the metal and the 2-phenylisoquinoline group at **9** and the metal and 2-*p*-tolylpyridine at **11** and **12**. However, for **10**, the situation is different; while it sits on the metal and 2-phenypyridine group of the **a** isomer, it sits on the metal and a *p*-tolylpyridine ligand on the **b** isomer.

The isomer stereochemistry does not show a significant influence on the experimental emission wavelengths, although pairwise comparison of the calculated values suggests trends: a very slight redshift for **9a** compared to **9b** and very slight blueshifts for **10a**–**12a** with respect to **10b**–**12b**. On the contrary, the difference in quantum yield between isomers is dramatic in some cases and seems to be associated with the stability of the stereochemistry. The more stable isomer shows a higher quantum yield, **9a** > **9b**, **10a** > **10b**, **11b** > **11a**, and **12b** > **12a**. The more stable isomer also presents a higher color purity, lower fwhm value, although in this case the differences are very slight and are in the range of 3–47 nm. The lifetimes are short for the nine emitters, in the range of 1 to 5 μs. The decrease in quantum yields is joined with the rise of the nonradiative rate constants (*k*_nr_) along with a slight mitigation of the radiative ones (*k*_r_).

## Concluding Remarks

This study reveals the discovery of two complementary methods to prepare phosphorescent iridium(III) heteroleptic emitters of the class [**3b**+**3b**+**3b′**] with two orthometalated **3b** ligands of the 2-phenylpyridine type with pyridyl groups arranged in *cis* and a third ligand (**3b′**) *C,N*-, *O,N*-, or *O,O*-donor.

The ability of the pentahydride IrH_5_(P^i^Pr_3_)_2_ to activate C–H bonds has certainly facilitated the discovery. This pentahydride activates an *ortho*-CH bond of 2-phenylpyridine-type molecules to produce the well-known *fac*-[Ir(**3b**)_3_] emitters in almost quantitative yield. Stirring these emitters in the appropriate amount of a saturated solution of HCl in toluene results in the release of one of the **3b** ligands and the protonation of the resulting 2-phenylpyridine-like molecule to the corresponding pyridinium chloride. Departure of ligand **3b** by addition of HCl to *fac*-[Ir(**3b**)_3_] generates an IrCl(**3b**)_2_ fragment, which maintains the *cis* arrangement of the pyridyl groups. This five-coordinate mononuclear fragment is then trapped by the pyridinium chloride, before the *cis*-to-*trans* rearrangement of the pyridyl groups takes place. The adduct stabilized with the pyridinium chloride directly generates *cis*-pyridyl emitters of the class [**3b**+**3b**+**3b′**] with **3b′** being a *C,N*-ligand by transmetalation reactions. In addition, it can be transformed into a *cis*-[Ir(μ-OH)(**3b**)_2_]_2_ dimer, which is a useful starting complex for preparing [**3b**+**3b**+**3b′**] emitters with **3b′** being a *O,N*- or *O,O*-ligand, when the precursor molecule of **3b′** has a fairly acidic hydrogen atom, suitable for removal by hydroxide groups.

The procedures discovered have made it possible to prepare four emitters of class [**3b**+**3b**+**3b′**] with *cis*-pyridyl and to compare their emissive properties with those of their usual *trans*-pyridyl isomers: two with a **3b′***C,N*-ligand, one with a **3b′***O,N*-ligand, and one with a **3b′***O,O*-ligand. DFT calculations indicate that the former are more stable than the respective *trans*-pyridyl isomers, while the second and third are less stable. The stereochemistry does not significantly influence the emission wavelengths of the emitter. On the contrary, its efficiency is highly dependent on and associated with the stability of the isomer. The more stable isomer shows a higher quantum yield and color purity.

*cis*-Pyridyl iridium(III) emitters of the class [**3b**+**3b**+**3b′**] with two orthometalated **3b** ligands of the 2-phenylpyridine type and a third **3b′***X,Y*- or *X,X*-ligand are now easily accessible through two complementary procedures, regardless of the relative stability of this stereochemistry with respect to others of the same stoichiometry. Therefore, we can say that two new synthetic tools have been discovered that will help in the effort to understand more deeply the influence of stereochemistry on the properties of this class of emitters.

## Experimental Section

### General Information

All reactions were carried out with the exclusion of air using Schlenk-tube techniques or in a drybox. Instrumental methods and X-ray details are given in the Supporting Information. In the NMR spectra (Figures S78–S104), the chemical shifts (in ppm) are referenced to residual solvent peaks (^1^H, ^13^C{^1^H}) or external 85% H_3_PO_4_ (^31^P{^1^H}), while coupling constants are given in hertz. IrHCl_2_(P^i^Pr_3_)_2_,^34^*trans*-[Ir(μ-Cl){κ^2^-*C,N*-[C_6_MeH_3_-py]}_2_]_2_,^16c^**10b**,^32^**11b**,^16c^ and **12b**(16a) were prepared according to the reported procedures.

### Preparation of IrH_5_(P^i^Pr_3_)_2_ (**1**)

Methanol (drop by drop and very slowly) was added to a suspension of IrHCl_2_(P^i^Pr_3_)_2_ (1.0 g) and NaBH_4_ (400.0 mg) in toluene (20 mL). During the methanol addition, the evolution of hydrogen was observed, and the color of the solution turned from dark purple to colorless. Then, the solvent was removed under vacuum, and toluene (20 mL) was added. The resulting suspension was filtered to remove the sodium salts, and the solution thus obtained was evaporated to dryness to afford a white residue. Addition of cold methanol afforded a white solid that was washed with methanol (2 × 5 mL) and dried under vacuum. Yield: 646 mg (73%). ^1^H NMR (300 MHz, C_6_D_6_, 298 K): δ 1.71 (m, 6H, PC*H*(CH_3_)_2_), 1.13 (dvt, *J*_H–H_ = 6.9, *N* = 13.7, 36H, PCH(C*H*_3_)_2_), −10.85 (t, *J*_H–P_ = 12.2, 5H, Ir–H). ^31^P{^1^H} NMR (121 MHz, C_6_D_6_, 298 K): δ 45.7 (s, sextet under off-resonance conditions). These NMR data are in agreement with those reported for this compound.^26c^

### Reaction of **1** with 2-(*p*-Tolyl)pyridine: Preparation of *fac*-[Ir{κ^2^-*C,N*-[C_6_MeH_3_-py]}_3_] (**2**)

A mixture of **1** (300 mg, 0.58 mmol) and 2-(*p*-tolyl)pyridine (496 μL, 2.90 mmol) in 1-phenylethanol (4 mL) was heated under reflux for 72 h. After this, it was concentrated to approximately 0.1 mL. Addition of diethyl ether (5 mL) afforded a yellow solid, which was washed with further portions of ether (5 × 5 mL) and dried in vacuo. Yield: 350 mg (86%). Anal. Calcd for C_36_H_30_IrN_3_ (%): C, 62.05; H, 4.34; N, 6.03. Found: C, 62.00; H, 4.50; N, 6.01. HRMS (electrospray, *m*/*z*) calcd for C_36_H_30_IrN_3_ [M]^+^: 697.2065; found 697.2069. ^1^H NMR (300 MHz, DMSO-*d*_6_, 298 K): δ 8.06 (d, *J*_H–H_ = 8.3, 3H, py), 7.74 (m, 3H, py), 7.63 (d, *J*_H–H_ = 8.0, 3H, *p*-tolyl), 7.37 (d, *J*_H–H_ = 5.5, 3H, py), 7.05 (m, 3H, py), 6.62 (d, *J*_H–H_ = 8.0, 3H, *p*-tolyl), 6.62 (s, 3H, *p*-tolyl), 2.00 (s, 9H, CH_3_). These ^1^H NMR data agree with those previously reported for this compound.^8^

### Reaction of **1** with 4,5-Dimethyl-2-phenylpyridine: Preparation of *fac*-[Ir{κ^2^-*C,N*-[C_6_H_4_-pyMe_2_]}_3_] (**3**)

A mixture of **1** (300 mg, 0.58 mmol) and 4,5-dimethyl-2-phenylpyridine (523 μL, 2.9 mmol) in 1-phenylethanol (1 mL) was heated under reflux for 72 h. After this, it was concentrated to approximately 0.1 mL. Addition of diethyl ether (5 mL) afforded a yellow solid, which upon removal of the supernatant solution was washed with diethyl ether (5 × 5 mL), and finally, it was dried in vacuo. Yield: 364 mg (85%). Anal. Calcd for C_39_H_36_IrN_3_ (%): C, 63.39; H, 4.91; N, 5.69. Found: C, 62.99; H, 4.90; N, 5.71. HRMS (electrospray, *m*/*z*) calcd for C_39_H_36_IrN_3_Na [M + Na]^+^: 762.2431; found 762.2409. ^1^H NMR (300 MHz, CD_2_Cl_2_, 298 K): δ 7.69 (s, 3H, py), 7.61 (d, *J*_H–H_ = 7.6, 3H, Ph), 7.31 (s, 3H, py), 6.84 (m, 3H, Ph), 6.73 (m, 3H, Ph), 6.67 (m, 3H, Ph), 2.36 (s, 9H, CH_3_), 2.05 (s, 9H, CH_3_). ^13^C{^1^H}-apt NMR (75.45 MHz, CD_2_Cl_2_, 298 K): δ 164.3 (s, C py), 161.4 (s, C Ph), 147.0 (s, CH py), 146.9 (s, C py), 144.8 (s, C Ph), 137.0 (s, CH Ph), 131.5 (s, C py), 129.3 (s, CH Ph), 123.8 (s, CH Ph), 119.9 (s, CH py), 119.8 (s, CH Ph), 19.8 (s, CH_3_), 16.7 (s, CH_3_).

### Reaction of **1** with 1-Phenylisoquinoline: Preparation of *fac*-[Ir{κ^2^-*C,N*-[C_6_H_4_–Isoqui]}_3_] (**4**)

A mixture of **1** (300 mg, 0.58 mmol) and 1-phenylisoquinoline (595 mg, 2.9 mmol) in 1-phenylethanol (1 mL) was heated under reflux for 72 h. After this, it was concentrated to approximately 0.1 mL. Addition of diethyl ether (5 mL) afforded a red solid, which upon removal of the supernatant solution was washed with diethyl ether (5 × 5 mL), and finally, it was dried in vacuo. Yield: 434 mg (93%). Anal. Calcd for C_45_H_30_IrN_3_ (%): C, 67.14; H, 3.76; N, 5.22. Found: C, 67.00; H, 3.72; N, 5.22. HRMS (electrospray, *m*/*z*) calcd. for C_45_H_29_IrN_3_ [M – H]^+^: 804.1988; found 804.1966. ^1^H NMR (300 MHz, CD_2_Cl_2_, 298 K): δ 8.97 (m, 3H, Ar), 8.21 (d, *J*_H–H_ = 8.0, 3H, Ar), 7.78 (m, 3H, Ar), 7.68 (m, 6H, Ar), 7.40 (d, *J*_H–H_ = 6.2, 3H, Ar), 7.19 (d, *J*_H–H_ = 6.2, 3H, Ar), 6.89 (m, 9H, Ar). ^13^C{^1^H}-apt NMR (75.45 MHz, CD_2_Cl_2_, 298 K): δ 167.7 (s, C), 165.0 (s, C), 145.8 (s, C), 140.2 (s, CH), 137.5 (s, CH), 137.1 (s, C), 130.7, 130.6, 130.0, 128.1, 127.9, 127.5 (all s, CH), 126.9 (s, C), 120.9 (s, CH), 119.9 (s, CH). These NMR data agree with those previously reported for this compound.^14d,14e^

### Reaction of **2** with Hydrogen Chloride: Preparation of IrCl{κ^2^-*C,N*-[C_6_MeH_3_-py]}_2_{κ^1^-*Cl*-[Cl–H-py-C_6_MeH_4_]} (**5**)

Complex **2** (200 mg, 0.29 mmol) was added to a 22 mL HCl toluene solution (0.20 M). The resulting suspension was stirred at room temperature for 12 h. The solvent was removed under vacuo to afford an orange residue. Addition of diethyl ether (3 mL) afforded an orange solid that was washed with diethyl ether (2 × 3 mL) and dried in vacuo. Yield: 190 mg (86%). Anal. Calcd for C_36_H_32_Cl_2_IrN_3_ (%): C, 56.17; H, 4.19; N, 5.46. Found: C, 55.96; H, 4.19; N, 5.46. HRMS (electrospray, *m*/*z*) calcd for C_24_H_20_IrN_2_ [M – Cl]^−^: 529.1206; found 529.1229. Upon dissolving in dimethyl sulfoxide-*d*_6_, 2-(*p*-tolyl)pyridinium chloride is released and complex *cis*-[IrCl{κ^2^-*C,N*-[C_6_MeH_3_-py]}_2_{κ^1^-*S*-[S(O)Me_2_]}] (**7**) is formed. ^1^H NMR (300 MHz, DMSO-*d*_6_, 298 K): δ 9.91–9.86 (m, 1H, CH Ar), 8.78–8.69 (m, 1H, CH Ar 2-(*p*-tolyl)-py·HCl), 8.22–8.11 (3H, 1H CH Ar + 2H CH Ar 2-(*p*-tolyl)-py·HCl), 8.08–7.95 (4H, 2H CH Ar + 2H CH Ar 2-(*p*-tolyl)-py·HCl), 7.87 (s, 1H, CH Ar), 7.80–7.57 (5H, 4H CH Ar + 1H CH Ar 2-(*p*-tolyl)-py·HCl), 7.54–7.48 (m, 1H, CH Ar), 7.41–7.34 (m, 2H, CH Ar 2-(*p*-tolyl)-py·HCl), 7.07–7.00 (m, 1H, CH Ar), 6.99–6.91(m, 1H, CH Ar), 6.66–6.58 (m, 1H, CH Ar), 5.97 (s, 1H, CH Ar), 2.44 (s, 3H, CH_3_) 2.39 (s, 3H, CH_3,_ 2-(*p*-tolyl)py·HCl), 1.89 (s, 3H, CH_3_). ^13^C{^1^H}-apt NMR (75.48 MHz, DMSO-*d*_6_, 298 K): δ 190.4, 186.0, 168.5, 165.3, 152.0, 151.7 (all C_q_ Ar), 151.5, 149.9 (both CH, Ar), 142.5, 142.0, 139.9 (all C_q_ Ar), 138.9, 138.5 (both CH, Ar), 138.0, 137.2 (both C_q_ Ar), 136.7, 135.0, 133.6, 130.1, 129.4, 129.1, 128.7, 128.4, 127.6, 127.3, 125.1, 124.2, 124.0, 123.7, 123.3, 122.8, 119.8, 119.8 (all CH, Ar), 22.1, 21.9, 21.3 (all CH_3_).

### Preparation of *cis*-[Ir(μ-Cl){κ^2^-*C,N*-[C_6_MeH_3_-py]}_2_]_2_ (**6**)

A solution of potassium hydroxide (85% weight, 66 mg, 1.00 mmol) in water (1 mL) was added over a solution of complex **5** (200 mg, 0.26 mmol) in acetone (4 mL). The mixture was kept stirring at room temperature for 18 h, upon which an orange yellowish precipitate is formed. Then, the supernatant solution was removed, and the solid was washed with diethyl ether (3 × 4 mL) and dried under vacuo. Yield: 130 mg (89%). Anal. Calcd for C_48_H_40_Cl_2_Ir_2_N_4_ (%): C, 51.10; H, 3.57; N, 4.97. Found: C, 51.28; H, 3.86; N, 4.81. HRMS (MALDI, *m*/*z*) calcd. for C_48_H_40_ClIr_2_N_4_ [M – Cl]^+^: 1093.219; found 1093.514. Upon dissolving in dimethyl sulfoxide-*d*_6_, complex *cis*-[IrCl{κ^2^-*C,N*-[C_6_MeH_3_-py]}_2_{κ^1^-*S*-[S(O)Me_2_]}] (**7**) is formed. ^1^H NMR (300 MHz, DMSO-*d*_6_, 298 K): δ 9.91–9.86 (m, 1H, CH Ar), 8.22–8.11 (m, 1H, CH Ar), 8.08–7.95 (2H, CH Ar), 7.87 (s, 1H, CH Ar), 7.80–7.57 (4H, CH Ar), 7.54–7.48 (m, 1H, CH Ar), 7.07–7.00 (m, 1H, CH Ar), 6.99–6.91(m, 1H, CH Ar), 6.66–6.58 (m, 1H, CH Ar), 5.97 (s, 1H, CH Ar), 2.44 (s, 3H, CH_3_), 1.89 (s, 3H, CH_3_).

### Preparation of *cis*-[Ir(μ-OH){κ^2^-*C,N*-[C_6_MeH_3_-py]}_2_]_2_ (**8**)

A mixture of **6** (335 mg, 0.297 mmol), CsOH·H_2_O (498.6 mg, 2.97 mmol), and silver acetate (99.1 mg, 0.594 mmol) in dichloromethane (40 mL) was stirred protected from the light at room temperature for 60 h. After this, the resulting suspension was filtered through Celite, and the red liquors were evaporated to dryness. Addition of pentane (4 mL) afforded an orange solid that was further washed with pentane (2 × 2 mL) and dried in vacuo. Yield: 180 mg (56%). Anal. Calcd for C_48_H_42_Ir_2_N_4_O_2_ (%): C, 52.83; H, 3.88; N, 5.13. Found: C, 52.48; H, 3.61; N, 4.92. HRMS (electrospray, *m*/*z*) calcd for C_48_H_41_Ir_2_N_4_O [M – OH]^+^: 1073.2512; found 1073.2501. ^1^H NMR (300 MHz, CD_2_Cl_2_, 298 K): δ 8.65 (m, 1H, CH py), 7.95 (d, *J*_H–H_ = 8.1, 1H, py), 7.68–7.62 (m, 1H, 1H py), 7.59 (d, *J*_H–H_ = 6.0, 1H *p*-tol), 7.55 (d, *J*_H–H_ = 8.0, 1H py), 7.50 (d, *J*_H–H_ = 7.8, 1H *p*-tol), 7.23–7.18 (m, 1H, CH py), 6.93 (d, *J*_H–H_ = 5.4, 1H py), 6.89 (d, *J*_H–H_ = 7.9, 1H, *p*-tol), 6.78 (s, 1H, *p*-tol), 6.76–6.71 (m, 1H, py), 6.50–6.47 (m, 1H, *p*-tol), 6.39–6.34 (m, 1H, py), 6.16 (s, 1H, *p*-tol), 2.14, 1.91 (both s, 3H each, CH_3_), −0.45 (s, 1H, OH). ^13^C{^1^H}-apt NMR (75.45 MHz, CD_2_Cl_2_, 298 K): δ 171.8, 166.3, 159.7 (all s, C Ar), 149.0, 147.7 (both s, CH Ar), 143.2, 142.4 (both s, C Ar), 139.3 (s, CH Ar), 138.9, 138.2 (both s, C Ar), 136.9, 134.8, 134.7 123.9, 123.4, 122.0, 121.6, 121.5, 120.2, 118.5, 117.2 (all s, CH Ar), 22.3, 21.4 (both s, CH_3_).

### Preparation of *trans*-[Ir(μ-OH){κ^2^-*C,N*-[MeC_6_H_3_-py]}_2_]_2_

A mixture of *trans*-[Ir(μ-Cl){κ^2^-*C,N*-(C_6_MeH_3_-py)}_2_]_2_ (2.5 g, 2.216 mmol) and AgBF_4_ (863.5 mg, 4.432 mmol) in acetone (30 mL) was stirred at room temperature protected from the light for 2.5 h. After this, the resulting suspension was filtered through Celite under argon to remove the silver salts that were washed with acetone until the extracted solutions were colorless. Then, the combined brown yellowish solution was concentrated to ca. 10 mL. The addition of a 0.87 M solution of KOH formed an orange suspension that was stirred for 30 min. The suspension was filtered, and the orange solid was washed with distilled water (5 × 15 mL) and dried in vacuo. Yield: 2.17 g (90%). Anal. Calcd for C_48_H_42_Ir_2_N_4_O_2_ (%): C, 52.83; H, 3.88; N, 5.13. Found: C, 52.53; H, 3.77; N, 5.01. HRMS (electrospray, *m*/*z*) calcd for C_48_H_41_Ir_2_N_4_O [M–OH]^+^: 1075.2539; found 1075.2515. ^1^H NMR (300 MHz, CD_2_Cl_2_, 298 K): δ 8.63 (m, 2H, CH py), 7.95 (d, *J*_H–H_ = 8.1, 1H, py), 7.59 (td, *J*_H–H_ = 7.8, 1.6, 2H py), 7.44 (d, *J*_H–H_ = 7.9, 2H, *p*-tol), 6.67–6.50 (2H py +2H *p*-tol), 5.73 (s, 2H, *p*-tol), 2.14, 1.93 (s, 6H, CH_3_), −1.53 (s, 1H, OH). ^13^C{^1^H}-apt NMR (75.45 MHz, CD_2_Cl_2_, 298 K): δ 169.4, 150.9 (both s, C Ar), 148.7 (s, CH Ar), 142.6, 139.0 (both s, C Ar), 135.8, 132.6, 123.9, 121.3, 121.0, 118.1 (all s, CH Ar), 21.6 (s, CH_3_).

### Preparation of *cis*-[Ir{κ^2^-*C,N*-[C_6_MeH_3_-py]}_2_{κ^2^-*C,N*-[C_6_H_4_–Isoqui]}] (**9a**)

A solution of 1-(2-bromophenyl)isoquinoline (369 mg, 1.3 mmol) in THF (50 mL) was cooled to −78 °C, and ^n^BuLi (1.22 mL, 1.6 M in hexanes, 1.96 mmol) was added dropwise. After stirring at this temperature for 2 h, **5** (500 mg, 0.65 mmol) was added into the lithiation flask, and the mixture was allowed to slowly warm to room temperature over 18 h. Then, it was concentrated to ca. 10 mL, and the crude was purified by column chromatography (deactivated silica gel) using dichloromethane/pentane (gradient elution from 1:3 to 3:1). The pure fractions were combined and concentrated to give a red solid which was washed with further portions of pentane (3 × 5 mL), and it was dried in vacuo. Yield: 167 mg (35%). Anal. Calcd for C_39_H_30_IrN_3_ (%): C, 63.91; H, 4.13; N, 5.73. Found: C, 63.90; H, 4.05; N, 5.79. HRMS (electrospray, *m*/*z*) calcd for C_39_H_31_IrN_3_ [M + H]^+^: 734.2144; found 734.2146. ^1^H NMR (300 MHz, CD_2_Cl_2_, 298 K): δ 8.97 (m, 1H, CH Ar), 8.21 (d, *J*_H–H_ = 7.9, 1H, CH Ar), 7.87 (t, *J*_H–H_ = 8.3, 2H, CH Ar), 7.80 (m, 1H, CH Ar), 7.648–7.5 (m, 6H, CH Ar), 7.50 (d, *J*_H–H_ = 7.5, 1H, CH Ar), 7.45 (d, *J*_H–H_ = 6.2, 1H, CH Ar), 7.35 (d, *J*_H–H_ = 9.7, 1H, CH Ar), 7.23 (d, *J*_H–H_ = 6.1, 1H, CH Ar), 6.99 (m, 2H, CH Ar), 6.90–6.60 (m, 5H, CH Ar), 6.62 (s, 1H, CH Ar), 6.50 (s, 1H, CH Ar), 2.11, 2.05 (both s, 3H each, CH_3_). ^13^C{^1^H}-apt NMR (75.45 MHz, CD_2_Cl_2_, 298 K): δ 166.9, 165.2, 162.2, 161.3 (all s, C), 147.7, 147.2 (both s, CH Ar), 145.6, 141.7, 141.6 (all s, C Ar), 139.9, (s, CH Ar), 139.9, 139.8 (both s, C Ar), 137.9, 137.7, 137.3 (all s, CH Ar), 137.0 (s, C Ar), 136.4 (s, CH Ar), 133.1 (s, C Ar), 130.6, 129.9, 127.9, 127.4 (all s, CH Ar), 126.9, 126.8 (both s, C Ar), 124.2, 121.9. 121.6, 121.4, 120.7, 119.6, 118.9 (all s, CH Ar), 21.9, 21.8 (both s, CH_3_).

### Preparation of *trans*-[Ir{κ^2^-*C,N*-[C_6_MeH_3_-py]}_2_{κ^2^-*C,N*-[C_6_H_4_–Isoqui]}] (**9b**)

*trans*-[Ir(μ-OH){κ^2^-*C,N*-(MeC_6_H_3_-py)}_2_]_2_ (100 mg, 0.092 mmol) and 1-phenylisoquinoline (38 mg, 0.183 mmol) were suspended in dichloromethane (3 mL) and stirred at room temperature for 7 days. After this, it was evaporated to dryness, and the crude was washed with a mixture of dichloromethane:diethyl ether 2:1 (3 mL) and pentane (2 mL × 2). The red-orangish solid obtained was dried in vacuum. Yield: 29 mg (22%). Anal. Calcd for C_39_H_30_IrN_3_ (%): C, 63.91; H, 4.13; N, 5.73. Found: C, 63.69; H, 4.05; N, 5.79. HRMS (electrospray, *m*/*z*) calcd for C_39_H_31_IrN_3_ [M + H]^+^: 734.2144; found 734.2134. ^1^H NMR (300 MHz, CD_2_Cl_2_, 298 K): δ 8.98–8.93 (m, 1H, CH Ar), 8.21 (d, *J*_H–H_ = 7.8, 1H, CH Ar), 8.06 (d, *J*_H–H_ = 6.0, 1H, CH Ar), 7.92 (d, *J*_H–H_ = 6.0, 1H, CH Ar), 7.82–7.73 (3H, CH Ar), 7.69–7.56 (3H CH Ar + 2H CH *p*-tol), 7.49 (2H, CH Ar), 7.23 (d, *J*_H–H_ = 6.2, 1H, CH Ar), 7.08–6.89 (3H, CH Ar), 6.82 (dd, *J*_H–H_ = 8.0, 1.9, 1H, *p*-tol), 6.74 (dd, *J*_H–H_ = 8.0, 1.9, 1H, *p*-tol), 6.70–6.62 (2H, CH Ar), 6.40 (s, 1H, *p*-tol), 6.22 (s, 1H, *p*-tol), 2.16, 2.14 (both s, 3H each, CH_3_). ^13^C{^1^H}-apt NMR (75.45 MHz, CD_2_Cl_2_, 298 K): δ 180.5, 176.1, 170.9, 170.1, 168.1, 160.2, (all s, C), 153.5, 148.6 (both s, CH Ar), 147.4 (s, C Ar), 143.6 (s, CH Ar), 142.6, 140.3, 140.2, 139.8 (all s, C Ar), 137.9 (s, CH Ar), 137.4 (s, C Ar), 135.9, 134.5, 133.9, 131.6, 131.2, 130.9, 129.9, 128.4, 127.8, 127.4 (s, CH Ar), 126.9 (s, C Ar), 124.6, 124.3, 122.5, 122.0, 121.3, 121.0, 120.7, 120.5, 118.7, 118.5 (all s, CH Ar), 22.0 (s, CH_3_).

### Preparation of *cis*-[Ir{κ^2^-*C,N*-[C_6_MeH_3_-py]}_2_{κ^2^-*C,N*-[C_6_H_4_-py]}] (**10a**)

A solution of 2-(2-bromophenyl)pyridine (223 μL, 1.3 mmol) in THF (50 mL) was cooled to −78 °C, and ^n^BuLi (1.22 mL, 1.6 M in hexanes, 1.96 mmol) was added dropwise. After stirring at this temperature for 2 h, **5** (500 mg, 0.65 mmol) was added into the lithiation flask, and the mixture was allowed to slowly warm to room temperature over 18 h. Then, it was concentrated to ca. 10 mL and purified by column chromatography (basic aluminum oxide) using dichloromethane/pentane (gradient elution from 1:3 to 3:1). The combined pure fractions were concentrated to dryness to give a yellow solid, which was washed with further portions of pentane (3 × 5 mL) and dried in vacuo. Yield: 248 mg (56%). Anal. Calcd for C_35_H_28_IrN_3_ (%): C, 61.56; H, 4.13; N, 6.15. Found: C, 61.52; H, 4.02; N, 6.18. HRMS (electrospray, *m*/*z*) calcd for C_35_H_28_IrN_3_Na [M + Na]^+^: 706.1804; found 706.1824. ^1^H NMR (300 MHz, DMSO-*d*_6_, 298 K): δ 8.13 (d, *J*_H–H_ = 8.2, 1H, CH Ar), 8.06 (m, 2H, CH Ar), 7.75 (m, 4H, CH Ar), 7.64 (dd, *J*_H–H_ = 8.0, *J*_H–H_ = 2.2, 2H, CH Ar), 7.41 (m, 3H, CH Ar), 7.09 (m, 3H, CH Ar), 6.81 (m, 1H, CH Ar), 6.69 (m, 2H, CH Ar), 6.62 (d, *J*_H–H_ = 9.5, 2H, CH Ar), 6.52 (s, 1H, CH Ar), 6.48 (s, 1H, CH Ar), 2.00, 1.99 (both s, 3H each, CH_3_). ^13^C{^1^H}-apt NMR (75.45 MHz, DMSO-*d*_6_, 298 K): δ 165.6, 165.6, 165.6, 161.0, 161.0, 161.0 (all s, C), 146.7, 146.6, 146.5 (all s, CH Ar), 143.7, 141.2,141.2, 137.8, 137.8 (all s, C Ar), 136.9, 136.9, 136.8, 136.7. 136.3 (all s, CH Ar), 129.1, 128.9, 128.2, 125.3, 124.1, 124.1, 122.7, 122.2, 120.8. 119.5, 119.0, 118.7. 118.6 (all s, CH Ar), 21.5, 21.5 (both s, CH_3_). The NMR data are in agreement with those reported for this compound.^19^

### Preparation of *cis*-Ir{κ^2^-*C,N*-[C_6_MeH_3_-py]}_2_{κ^2^-*O,N*-[OC(O)-py]} (**11a**)

Complex **8** (100 mg, 0.0916 mmol) and picolinic acid (22.6 mg, 0.1832 mmol) were dissolved in acetone (3 mL), and the resulting orange solution was stirred at room temperature for 14 h. After this, the yellow suspension obtained was decanted. The orange supernatant solution was poured off, and the remaining yellow solid was washed with acetone (0.5 mL) and dried under vacuo. Yield: 67 mg (56%). Anal. Calcd for C_30_H_24_IrN_3_O_2_ (%): C, 55.37, H, 3.72, N, 6.46. Found: C, 55.02; H, 3.49; N, 6.34. HRMS (electrospray, *m*/*z*) calcd for C_30_H_25_IrN_3_O_2_ [M + H]: 652.1572; found 652.1555. ^1^H NMR (300 MHz, CD_2_Cl_2_, 298 K): δ 8.14 (d, *J*_H–H_ = 7.8, 1H, CH Ar), 7.98 (d, *J*_H–H_ = 8.4, 1H, CH Ar), 7.90 (dt, *J*_H–H_ = 7.8, *J*_H–H_ = 1.5, 1H, CH Ar), 7.81–7.74 (2H, CH Ar), 7.61–7.49 (6H, CH Ar), 7.34 (m, 1H, CH Ar), 7.28 (s, 1H, *p*-tol), 7.07 (m, 1H, CH Ar), 6.90 (d, *J*_H–H_ = 8.1, 1H, CH Ar), 6.72–6.70 (m, 2H, CH Ar), 6.37 (s, 1H, *p*-tol), 2.35, 2.04 (both s, 3H each, CH_3_). ^13^C{^1^H}-apt NMR (75.45 MHz, CD_2_Cl_2_, 298 K): δ 174.5, 171.0, 166.7, 157.0, 151.0 (all s, C Ar), 150.7 (CH Ar), 150.0 (s, C Ar), 147.2, 146.3 (both s, CH Ar), 142.8 (s, C Ar), 141.3, 140.9, 139.6 (all s, C Ar), 139.4, 138.3, 137.7, 136.3, 132.7, 128.6, 128.3, 124.4, 124.3, 123.5, 122.9, 122.7, 121.8, 119.6, 118.8 (all s, CH Ar), 22.0, 21.7 (both s, CH_3_).

### Reaction of IrCl{κ^2^-*C,N*-[C_6_MeH_3_-py]}_2_{κ^1^-*Cl*-[Cl–H-py-C_6_MeH_4_]} (**5**) with Thallium Acetylacetonate

An orange suspension of **5** (250 mg, 0.32 mmol) in fluorobenzene (8 mL) was treated with Tl(acac) (207 mg, 0.68 mmol). The reaction was vigorously stirred in darkness for 16 h. After this, the resulting bright yellow suspension was filtered through Celite to remove the thallium salts. The yellow solution obtained was concentrated to approximately 0.5 mL. Addition of diethyl ether (1 mL) afforded a yellow solid, which was further washed with diethyl ether (2 × 1 mL) and dried in vacuo. The ^1^H NMR spectrum of this crude solid shows a 3:1 mixture of *cis*-Ir{κ^2^-*C,N*-[C_6_MeH_3_-py]}_2_{κ^2^-*O,O*-[acac]} (**12a**) and *trans*-Ir{κ^2^-*C,N*-[C_6_MeH_3_-py]}_2_{κ^2^-*O,O*-[acac]} (**12b**). Complex **12a** was separated from the minor isomer by column chromatography (basic alumina) using *n*-hexane/dichloromethane (gradient elution from 100% to 0% *n*-hexane). Yield: 80 mg (39%).

#### Data for **12a**

Anal. Calcd for C_29_H_27_IrN_2_O_2_ (%): C, 55.49; H, 4.34; N, 4.46. Found C, 55.12; H, 4.44; N, 4.47. HRMS (electrospray, *m*/*z*) calcd for C_29_H_27_IrN_2_O_2_ [M]^+^: 628.1672; found 628.1696. ^1^H NMR (300 MHz, CD_2_Cl_2_, 298 K): δ 8.46 (m, 1H, CH py), 7.96 (d, *J*_H–H_ = 8.2, 1H, CH py), 7.81 (m, 1H, CH py), 7.67 (m, 1H, CH py), 7.57 (d, *J*_H–H_ = 7.9, 1H, CH tolyl), 7.52 (d, *J*_H–H_ = 7.9, 1H, CH tolyl), 7.48 (m, 1H, CH py), 7.41 (m, 1H, CH py), 7.29 (m, 1H, CH py), 7.23 (m, 1H, CH tolyl), 6.94 (m, 1H, CH tolyl), 6.60 (m, 2H, CH py + CH tolyl), 6.34 (m, 1H, CH tolyl), 5.29 (s, 1H, CH acac), 2.45, 2.02 (both s, 3H each, CH_3_ tolyl), 1.78, 1.73 (both s, 3H each, CH_3_ acac). ^13^C{^1^H}-apt NMR (75.45 MHz, CD_2_Cl_2_, 298 K): δ 183.9, 183.2 (both s, C acac), 171.5 (s, C Ar), 166.1 (s, C Ar), 159.1 (s, C Ar), 151.3 (s, CH Ar), 147.0 (s, CH Ar), 146.9, 143.3, 142.2, 140.2 (all s, C Ar), 139.8(s, CH Ar), 138.8 (s, C Ar), 138.0, 136.2, 133.4, 124.2, 124.1, 122.7, 122.4, 122.0, 120.9, 119.1, 118.0 (all s, CH Ar), 101.5 (s, CH acac), 28.6, 27.9 (both s, CH_3_ tolyl), 22.1, 21.6 (both s, CH_3_ acac).

## Supporting Information Available

General information for the experimental section, structural analysis of complexes **3**, **5**, **9a**, **9b**, and **11a**, computational details, energies of optimized structures, observed and calculated UV–vis spectra of **3** and **a** and **b** isomers of **9**–**12**, analysis of computed UV–vis data, theoretical analysis of molecular orbitals, cyclic voltammograms of **3** and **a** and **b** isomers of **9**–**12**, photophysical studies, and NMR spectra (PDF)
Atomic coordinates of optimized complexes (XYZ)

## Supplementary Material

ic3c03133_si_001.pdf

ic3c03133_si_002.xyz
