# Supplementary material for: Two Synthetic Tools to Deepen the Understanding of the Influence of Stereochemistry on the Properties of Iridium(III) Heteroleptic Emitters
Source: Inorg Chem. 2023 Nov 21;62(49):19821–37. doi: 10.1021/acs.inorgchem.3c03133 (PMC10880056; doi:10.1021/acs.inorgchem.3c03133)
Supplement: Supplementary file 1 — ic3c03133_si_001.pdf [file ic3c03133_si_001.pdf]

SUPPORTING INFORMATION

Two Synthetic Tools to Deepen the Understanding of the Influence of Stereochemistry on the Properties of Iridium(III) Heteroleptic Emitters

Juan C. Babón,[†] Pierre-Luc T. Boudreault,[‡] Miguel A. Esteruelas,[†,*] Miguel A. Gaona,[†] Susana Izquierdo,[†] Montserrat Oliván,[†] Enrique Oñate,[†] Jui-Yi Tsai,[‡] and Andrea Vélez[†]

[†] Departamento de Química Inorgánica - Instituto de Síntesis Química y Catálisis Homogénea (ISQCH) - Centro de Innovación en Química Avanzada (ORFEO-CINQA), Universidad de Zaragoza - CSIC, 50009 Zaragoza, Spain
[‡] Universal Display Corporation, Ewing, New Jersey 08618, United States

* e-mail: maester@unizar.es

Contents:

- Experimental Section: General Information.	S2
- Structural Analysis of Complexes **3**, **5**, **9a**, **9b**, and **11a**	S3
- Computational Details and Energies of Calculated Complexes	S5
- UV-vis Spectra of **3** and Isomers **a** and **b** of **9-12** (Observed and Calculated)	S11
- Analysis of Computed UV-vis Data	S14
- Theoretical Analysis of Molecular Orbitals of Complex **3** and Isomers **a** and **b** of **9-12**	S23
- Cyclic Voltammograms	S41
- Photophysical Studies	S42
- NMR spectra	S65
- References	S91

• Experimental Section: General Information

All reactions were carried out with exclusion of air using Schlenk-tube techniques or in a drybox. Diethyl ether, dichloromethane, pentane and toluene were obtained oxygen- and water-free from an MBraun solvent purification apparatus, while acetone and tetrahydrofuran were dried and distilled under argon prior to use. 1-phenylethanol was deoxygenated prior to use. ^{1}H, $^{13}C\{^{1}H\}$ and $^{31}P\{^{1}H\}$ NMR spectra were recorded on Bruker 300 ARX, Bruker Avance 300 MHz, Bruker Avance 400 MHz, or Bruker Avance 500 MHz instruments. Chemical shifts (expressed in ppm) are referenced to residual solvent peaks. Coupling constants J and N ($N = J_{P\text{-}H} + J_{P'\text{-}H}$) are given in hertz. Attenuated total reflection infrared spectra (ATR-IR) of solid samples were run on a PerkinElmer Spectrum 100 FT-IR spectrometer. C, H, and N analyses were carried out in a PerkinElmer 2400 CHNS/O analyzer. High-resolution electrospray mass spectra were acquired using a MicroTOF-Q hybrid quadrupole time-of-flight spectrometer (Bruker Daltonics, Bremen, Germany). UV-visible spectra were recorded on an Evolution 600 spectrophotomer. Steady-state photoluminescence spectra and lifetime measurements were recorded with either a Jobin-Yvon Horiba Fluorolog FL-3-11 Tau 3 spectrometer or with a PicoQuant FluoTime 300 spectrometer (PMMA films and 2-methyl tetrahydrofuran solutions). Data were fitted to either monoexponential or biexponential functions. Relative amplitudes are given in parentheses for biexponential decays. Quantum yields were measured using the Hamamatsu Absolute PL Quantum Yield Measurement System C11347-11 (an uncertainty of ±5% is estimated). PMMA films at 5 wt % were prepared in a glove box dissolving 1 mg of compound and 19 mg of PMMA (average Mw 97,000, average Mn 46,000) in CH_2Cl_2 (1 mL). The solutions were filtered through a PTFE syringe filter (0.22 micron pore size, 17 mm diameter) and then drop-coated onto the quartz plates and dried. The 2-methyl tetrahydrofuran solutions were prepared in a glove box filled with argon. Cyclic voltammetry measurements were performed using a Voltalab PST050 potentiostat with Pt wire as working electrode, Pt wire as counter electrode, and saturated calomel (SCE) as reference electrode. The experiments were carried out under argon in dichloromethane solutions (10^{-3} M), with $[Bu_4N]PF_6$ as supporting electrolyte (0.1 M). Scan rate was 100 mV s^{-1}. The potentials were referenced to the ferrocene/ferrocenium (Fc/Fc^{+}) couple. $IrHCl_2(P^iPr_3)_2$ was prepared according to the reported procedure.[1]

• **Structural Analysis of Complexes 3, 5, 9a, 9b, and 11a**.

X-ray data were collected on a APEX CCD (**5**), and D8 Venture Bruker diffractometers (Mo radiation, λ = 0.71073 Å). The crystals were mounted under oil in a MiTeGen mount and cooled to 100(2) or 120(2) K with an open-flow nitrogen gas (Oxford Cryosystems). Data were collected using φ and/or ω narrow scans. Diffracted intensities were integrated and corrected for absorption effects using SAINT[2] and SADABS[3] programs, included in APEX4 package. The structures were solved by direct methods and refined by full-matrix least squares on F^2 with SHELXL2019,[4] including isotropic and subsequently anisotropic displacement parameters. The hydrogen atoms were observed in the last Fourier Maps or calculated, and refined freely or using a restricted riding model.
The disordered solvent molecules were refined with different moieties, restrained geometry and isotropic thermal parameters.

Crystal data for **3** (CCDC 2284937): $C_{39}H_{36}IrN_3$, CH_2Cl_2, M_W 823.83, yellow, irregular block, (0.280 x 0.055 x 0.010 mm^3), orthorhombic, space group Pbca, *a*: 16.106(7) Å, *b*: 17.317(6) Å, *c*: 24.729(11) Å, V = 6897(5) Å^3, Z = 8, Z' = 1, D_{calc}: 1.587 g cm^{-3}, F(000): 3280, T = 100(2) K, μ 4.059 mm^{-1}. 103485 measured reflections (2θ: 3-57°, ω and φ scans 0.5°), 10549 unique (R_{int} = 0.0445); min./max. transm. factors 0.771/0.862. Final agreement factors were R^1 = 0.0356 (7562 observed reflections, I > 2σ(I)) and wR^2 = 0.0898; data/restraints/parameters 10549/0/421; GoF = 1.009. Largest peak and hole 1.954 (close to Ir atoms) and -1.815 e/ Å^3.

Crystal data for **5** (CCDC 2284934): $C_{24}H_{20}Cl_2IrN_2$, $C_{12}H_{12}N$, 13/8(CH_2Cl_2), M_W 907.75, yellow, irregular block (0.166 x 0.158 x 0.157 mm^3), monoclinic, space group C2/c, *a*: 27.659(3) Å, *b*: 20.687(2) Å, *c*: 15.2308(15) Å, β: 113.5760(10)°, V = 7987.2(14) Å^3, Z = 8, Z' = 1, D_{calc}: 1.510 g cm^{-3}, F(000): 3586, T = 120(2) K, μ 3.723 mm^{-1}. 41432 measured reflections (2θ: 3-57°, ω scans 0.3°), 11575 unique (R_{int} = 0.0490); min./max. transm. factors 0.714/0.862. Final agreement factors were R^1 = 0.0524 (7891 observed reflections, I > 2σ(I)) and wR^2 = 0.1552; data/restraints/parameters 11575/18/441; GoF = 1.070. Largest peak and hole 2.450 (close to Ir atoms) and -1.642 e/ Å^3.

Crystal data for **9a** (CCDC 2284936): $C_{39}H_{30}IrN_3$, M_W 732.86, red, irregular block, (0.198 x 0.030 x 0.010 mm^3), orthorhombic, space group Pbca, *a*: 8.9452(2) Å, *b*: 18.6327(5) Å, *c*: 34.8486(9) Å, V = 5808.3(3) $Å^3$, Z = 8, Z' = 1, D_{calc}: 1.676 g cm^{-3}, F(000): 2896, T = 100(2) K, μ 4.631 mm^{-1}. 69243 measured reflections (2θ: 3-57°, ω and φ scans 0.5°), 7206 unique (R_{int} = 0.0383); min./max. transm. factors 0.700/0.862. Final agreement factors were R^1 = 0.0296 (6357 observed reflections, I > 2σ(I)) and wR^2 = 0.0774; data/restraints/parameters 7206/0/391; GoF = 1.069. Largest peak and hole 2.203 (close to Ir atoms) and -0.594 e/ $Å^3$.

Crystal data for **9b** (CCDC 2284938): $C_{39}H_{30}IrN_3$, 1/4($C_4H_{10}O$),1/4(CH_2Cl_2), M_W 772.62, orange, irregular block, (0.560 x 0.260 x 0.182 mm^3), triclinic, space group P-1, *a*: 14.1775(8) Å, *b*: 14.8030(8) Å, *c*: 17.4202(10) Å, *α*: 95.514(2)°, *β*: 97.443(2)°, *γ*: 112.637(2)°, V = 3303.1(3) $Å^3$, Z = 4, Z' = 2, D_{calc}: 1.554 g cm^{-3}, F(000): 1532, T = 100(2) K, μ 4.116 mm^{-1}. 195198 measured reflections (2θ: 3-57°, ω and φ scans 0.5°), 12285 unique (R_{int} = 0.0421); min./max. transm. factors 0.634/0.746. Final agreement factors were R^1 = 0.0534 (11670 observed reflections, I > 2σ(I)) and wR^2 = 0.1253; data/restraints/parameters 12285/4/828; GoF = 1.094. Largest peak and hole 6.798 (close to Ir atoms) and -3.957 e/ $Å^3$.

Crystal data for **11a** (CCDC 2284935): $C_{30}H_{24}IrN_3O_2$, 5/4(CH_2Cl_2), M_W 756.88, yellow, irregular block (0.185 x 0.169 x 0.118 mm^3), monoclinic, space group $P2_1/c$, *a*: 10.2360(3) Å, *b*: 12.2725(6) Å, *c*: 23.4660(11) Å, *β*: 102.281(2)°, V = 2880.4(2) $Å^3$, Z = 4, Z' = 1, D_{calc}: 1.745 g cm^{-3}, F(000): 1482, T = 100(2) K, μ 4.901 mm^{-1}. 70124 measured reflections (2θ: 3-57°, ω and φ scans 0.5°), 7161 unique (R_{int} = 0.0362); min./max. transm. factors 0.593/0.746. Final agreement factors were R^1 = 0.0347 (6647 observed reflections, I > 2σ(I)) and wR^2 = 0.1023; data/restraints/parameters 7161/8/363; GoF = 0.1023. Largest peak and hole 2.288 (close to Ir atoms) and -2.071 e/ $Å^3$.

• Computational Details and Energies of Calculated Complexes

Computational Details. All calculations were performed at the DFT level using the B3LYP functional[5] supplemented with the Grimme's dispersion correction D3[6] as implemented in Gaussian09.[7] Ir atoms were described by means of an effective core potential SDD for the inner electron[8] and its associated double-ζ basis set for the outer ones, complemented with a set of f-polarization functions for iridium.[9] The 6-31G** basis set was used for the H, C, N, and O atoms.[10] All minima were verified to have no negative frequencies. The geometries were fully optimized in THF (ε = 7.4257) solvent using the continuum SMD model.[11] We performed TD-DFT calculations at the same level of theory in THF calculating the lowest 50 singlet-singlet excitations at the ground state S_0. It should be noted that the singlet-triplet excitations are set to zero due to the neglect of spin-orbit coupling in the TDDFT calculations as implemented in G09. The UV/vis absorption spectra were obtained by using the GaussSum 3 software.[12] The phosphorescence emission compares well with the 0-0 transition calculated taking into account the zero point energies (zpe) of the geometries of both the optimized T_1 and S_0 states in THF.

Energies of Calculated Complexes

3-S_0

Zero-point correction= 0.646667 (Hartree/Particle)
Thermal correction to Energy= 0.685426
Thermal correction to Enthalpy= 0.686371
Thermal correction to Gibbs Free Energy= 0.575895
Sum of electronic and zero-point Energies= -1776.206107
Sum of electronic and thermal Energies= -1776.167348
Sum of electronic and thermal Enthalpies= -1776.166404
Sum of electronic and thermal Free Energies= -1776.276879

3-T_1

Zero-point correction= 0.643101 (Hartree/Particle)
Thermal correction to Energy= 0.682300
Thermal correction to Enthalpy= 0.683245
Thermal correction to Gibbs Free Energy= 0.571026
Sum of electronic and zero-point Energies= -1776.114468
Sum of electronic and thermal Energies= -1776.075268
Sum of electronic and thermal Enthalpies= -1776.074324
Sum of electronic and thermal Free Energies= -1776.186542

9a-S_0

Zero-point correction= 0.582657 (Hartree/Particle)

Thermal correction to Energy= 0.617784
Thermal correction to Enthalpy= 0.618728
Thermal correction to Gibbs Free Energy= 0.514587
Sum of electronic and zero-point Energies= -1772.613496
Sum of electronic and thermal Energies= -1772.578369
Sum of electronic and thermal Enthalpies= -1772.577425
Sum of electronic and thermal Free Energies= -1772.681566

9a-T$_1$
Zero-point correction= 0.579181 (Hartree/Particle)
Thermal correction to Energy= 0.614742
Thermal correction to Enthalpy= 0.615686
Thermal correction to Gibbs Free Energy= 0.509766
Sum of electronic and zero-point Energies= -1772.542309
Sum of electronic and thermal Energies= -1772.506747
Sum of electronic and thermal Enthalpies= -1772.505803
Sum of electronic and thermal Free Energies= -1772.611723

9b-S$_0$
Zero-point correction= 0.582794 (Hartree/Particle)
Thermal correction to Energy= 0.617810
Thermal correction to Enthalpy= 0.618754
Thermal correction to Gibbs Free Energy= 0.515052
Sum of electronic and zero-point Energies= -1772.600839
Sum of electronic and thermal Energies= -1772.565823
Sum of electronic and thermal Enthalpies= -1772.564879
Sum of electronic and thermal Free Energies= -1772.668580

9b-T$_1$
Zero-point correction= 0.579032 (Hartree/Particle)
Thermal correction to Energy= 0.614350
Thermal correction to Enthalpy= 0.615294
Thermal correction to Gibbs Free Energy= 0.510705
Sum of electronic and zero-point Energies= -1772.527790
Sum of electronic and thermal Energies= -1772.492472
Sum of electronic and thermal Enthalpies= -1772.491528
Sum of electronic and thermal Free Energies= -1772.596117

10a-S$_0$
Zero-point correction= 0.535621 (Hartree/Particle)
Thermal correction to Energy= 0.568116
Thermal correction to Enthalpy= 0.569060
Thermal correction to Gibbs Free Energy= 0.470599
Sum of electronic and zero-point Energies= -1619.011842
Sum of electronic and thermal Energies= -1618.979347
Sum of electronic and thermal Enthalpies= -1618.978403
Sum of electronic and thermal Free Energies= -1619.076864

10a-T$_1$
Zero-point correction= 0.531719 (Hartree/Particle)

Thermal correction to Energy= 0.564850
Thermal correction to Enthalpy= 0.565794
Thermal correction to Gibbs Free Energy= 0.465210
Sum of electronic and zero-point Energies= -1618.921505
Sum of electronic and thermal Energies= -1618.888374
Sum of electronic and thermal Enthalpies= -1618.887429
Sum of electronic and thermal Free Energies= -1618.988014

10b-S$_0$
Zero-point correction= 0.535442 (Hartree/Particle)
Thermal correction to Energy= 0.567854
Thermal correction to Enthalpy= 0.568798
Thermal correction to Gibbs Free Energy= 0.470972
Sum of electronic and zero-point Energies= -1619.000219
Sum of electronic and thermal Energies= -1618.967807
Sum of electronic and thermal Enthalpies= -1618.966863
Sum of electronic and thermal Free Energies= -1619.064689

10b-T$_1$
Zero-point correction= 0.532053 (Hartree/Particle)
Thermal correction to Energy= 0.565165
Thermal correction to Enthalpy= 0.566109
Thermal correction to Gibbs Free Energy= 0.465602
Sum of electronic and zero-point Energies= -1618.916244
Sum of electronic and thermal Energies= -1618.883132
Sum of electronic and thermal Enthalpies= -1618.882188
Sum of electronic and thermal Free Energies= -1618.982695

11a-S$_0$
Zero-point correction= 0.469924 (Hartree/Particle)
Thermal correction to Energy= 0.500399
Thermal correction to Enthalpy= 0.501343
Thermal correction to Gibbs Free Energy= 0.407311
Sum of electronic and zero-point Energies= -1576.593096
Sum of electronic and thermal Energies= -1576.562621
Sum of electronic and thermal Enthalpies= -1576.561677
Sum of electronic and thermal Free Energies= -1576.655709

11a-T$_1$
Zero-point correction= 0.465506 (Hartree/Particle)
Thermal correction to Energy= 0.496685
Thermal correction to Enthalpy= 0.497629
Thermal correction to Gibbs Free Energy= 0.401020
Sum of electronic and zero-point Energies= -1576.503876
Sum of electronic and thermal Energies= -1576.472697
Sum of electronic and thermal Enthalpies= -1576.471753
Sum of electronic and thermal Free Energies= -1576.568362

11b-S$_0$
Zero-point correction= 0.470064 (Hartree/Particle)

Thermal correction to Energy= 0.500505
Thermal correction to Enthalpy= 0.501449
Thermal correction to Gibbs Free Energy= 0.407660
Sum of electronic and zero-point Energies= -1576.600163
Sum of electronic and thermal Energies= -1576.569722
Sum of electronic and thermal Enthalpies= -1576.568778
Sum of electronic and thermal Free Energies= -1576.662567

11b-T$_1$
Zero-point correction= 0.466240 (Hartree/Particle)
Thermal correction to Energy= 0.497356
Thermal correction to Enthalpy= 0.498300
Thermal correction to Gibbs Free Energy= 0.401815
Sum of electronic and zero-point Energies= -1576.511758
Sum of electronic and thermal Energies= -1576.480643
Sum of electronic and thermal Enthalpies= -1576.479698
Sum of electronic and thermal Free Energies= -1576.576183

12a-S$_0$
Zero-point correction= 0.489288 (Hartree/Particle)
Thermal correction to Energy= 0.520725
Thermal correction to Enthalpy= 0.521669
Thermal correction to Gibbs Free Energy= 0.425883
Sum of electronic and zero-point Energies= -1485.501045
Sum of electronic and thermal Energies= -1485.469609
Sum of electronic and thermal Enthalpies= -1485.468665
Sum of electronic and thermal Free Energies= -1485.564450

12a-T$_1$
Zero-point correction= 0.485178 (Hartree/Particle)
Thermal correction to Energy= 0.517284
Thermal correction to Enthalpy= 0.518228
Thermal correction to Gibbs Free Energy= 0.419473
Sum of electronic and zero-point Energies= -1485.409366
Sum of electronic and thermal Energies= -1485.377260
Sum of electronic and thermal Enthalpies= -1485.376316
Sum of electronic and thermal Free Energies= -1485.475071

12b-S$_0$
Zero-point correction= 0.488542 (Hartree/Particle)
Thermal correction to Energy= 0.519442
Thermal correction to Enthalpy= 0.520387
Thermal correction to Gibbs Free Energy= 0.425611
Sum of electronic and zero-point Energies= -1485.507835
Sum of electronic and thermal Energies= -1485.476934
Sum of electronic and thermal Enthalpies= -1485.475990
Sum of electronic and thermal Free Energies= -1485.570766

12b-T$_1$
Zero-point correction= 0.484779 (Hartree/Particle)

Thermal correction to Energy= 0.517148
Thermal correction to Enthalpy= 0.518093
Thermal correction to Gibbs Free Energy= 0.418624
Sum of electronic and zero-point Energies= -1485.420270
Sum of electronic and thermal Energies= -1485.387901
Sum of electronic and thermal Enthalpies= -1485.386957
Sum of electronic and thermal Free Energies= -1485.486426

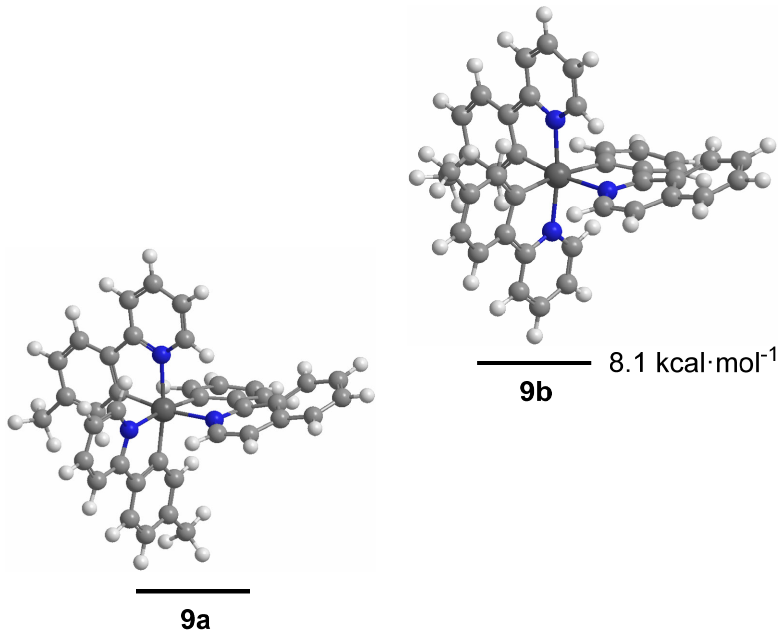

Figure S1. Relative stabilities of **9a** and **9b**.

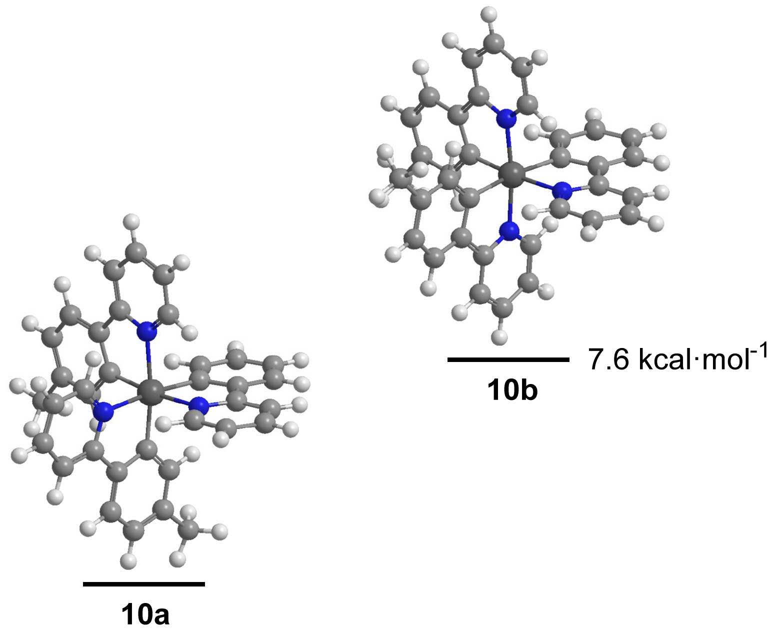

Figure S2. Relative stabilities of **10a** and **10b**.

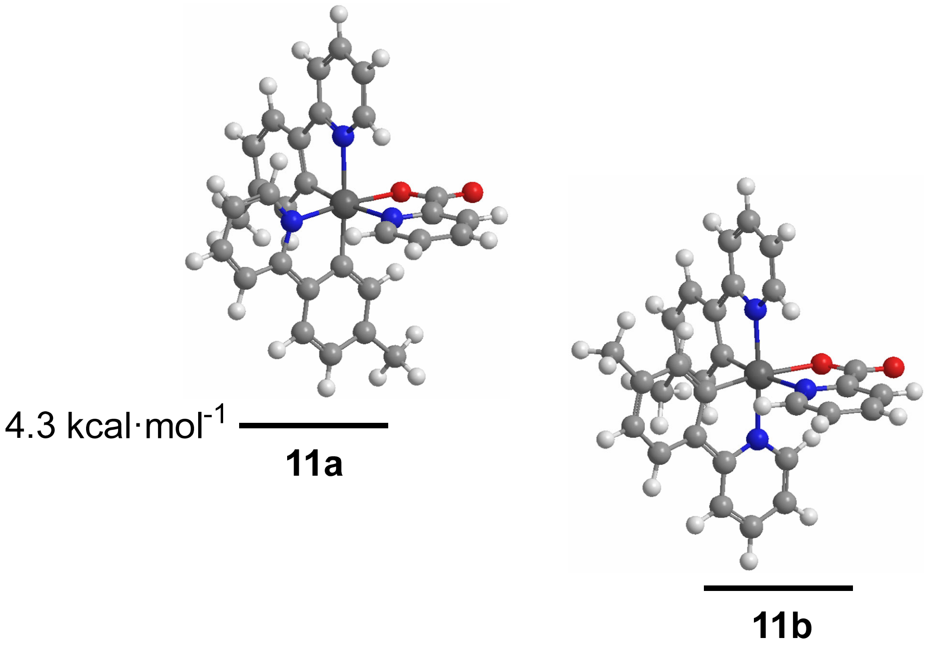

Figure S3. Relative stabilities of **11a** and **11b**.

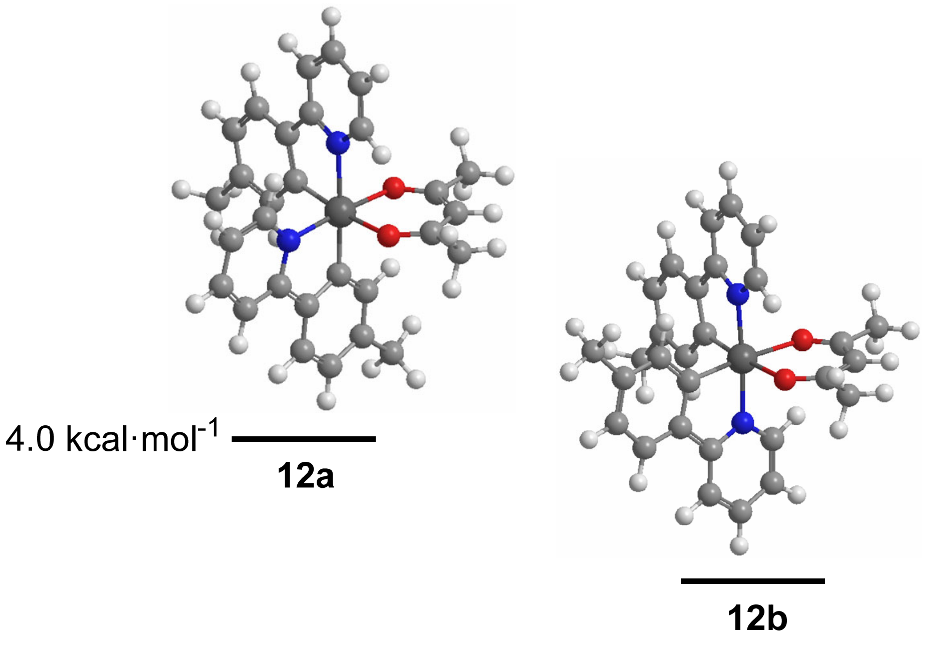

Figure S4. Relative stabilities of **12a** and **12b.**

• UV-vis Spectra of Complex 3 and Isomers a and b of 9-12 (Observed and Calculated)

(a) (b)

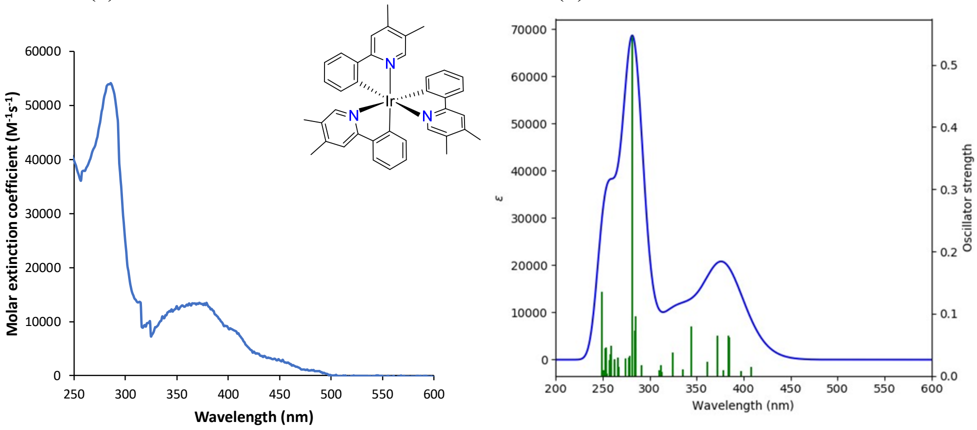

Figure S5. (a) Observed UV-vis spectrum of complex **3** in 2-MeTHF (1.0 x 10^{-5} M) and (b) calculated (B3LYP(GD3)//SDD(f)/6-31G**) in THF.

(a) (b)

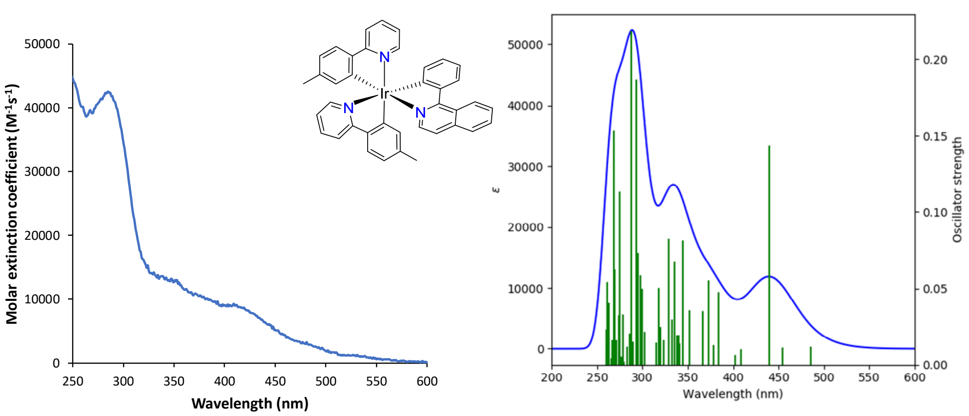

Figure S6. (a) Observed UV-vis spectrum of complex **9a** in 2-MeTHF (1.0 x 10^{-5} M) and (b) calculated (B3LYP(GD3)//SDD(f)/6-31G**) in THF.

(a) (b)

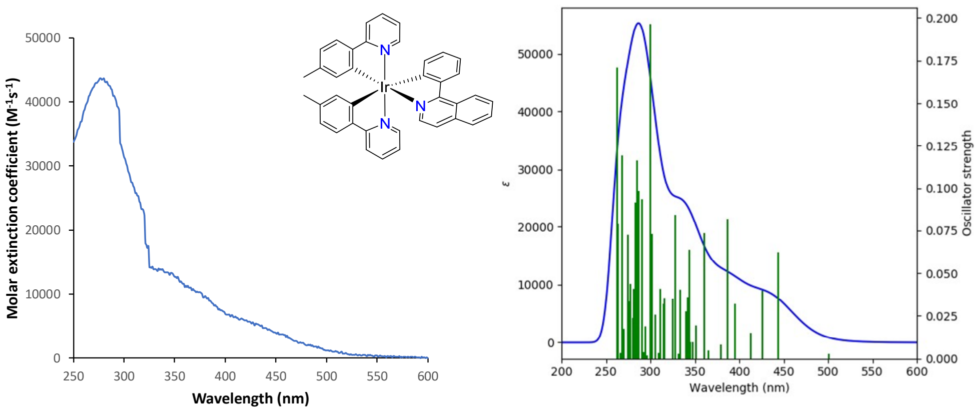

Figure S7. (a) Observed UV-vis spectrum of complex **9b** in 2-MeTHF (1.0 x 10^{-5} M) and (b) calculated (B3LYP(GD3)//SDD(f)/6-31G**) in THF.

(a) (b)

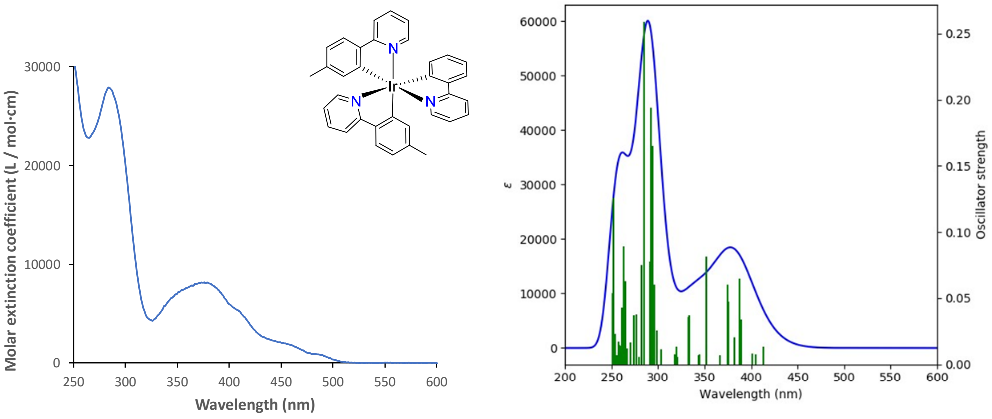

Figure S8. (a) Observed UV-vis spectrum of complex **10a** in 2-MeTHF (5.0 x 10^{-5} M) and (b) calculated (B3LYP(GD3)//SDD(f)/6-31G**) in THF.

(a) (b)

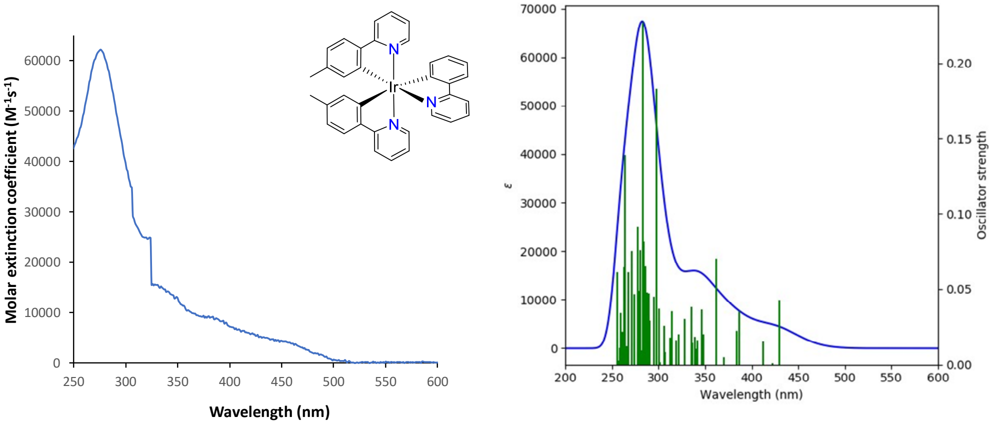

Figure S9. (a) Observed UV-vis spectrum of complex **10b** in 2-MeTHF (1.0 x 10^{-5} M) and (b) calculated (B3LYP(GD3)//SDD(f)/6-31G**) in THF.

(a) (b)

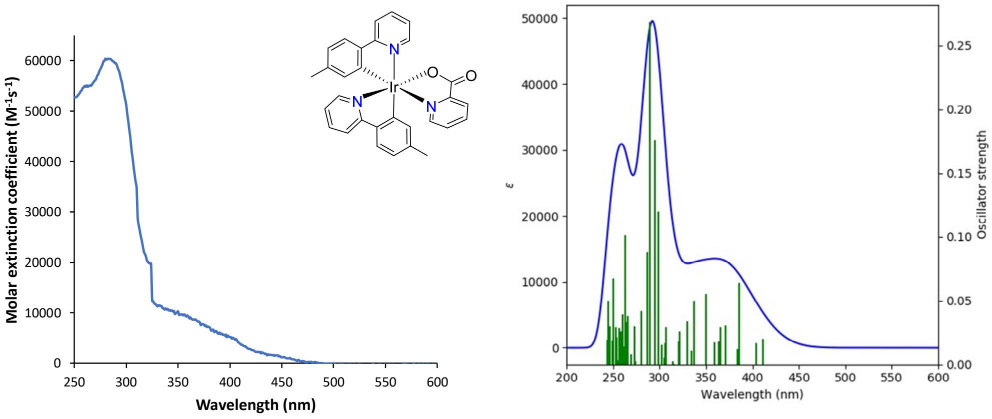

Figure S10. (a) Observed UV-vis spectrum of complex **11a** in 2-MeTHF (1.0 x 10^{-5} M) and (b) calculated (B3LYP(GD3)//SDD(f)/6-31G**) in THF.

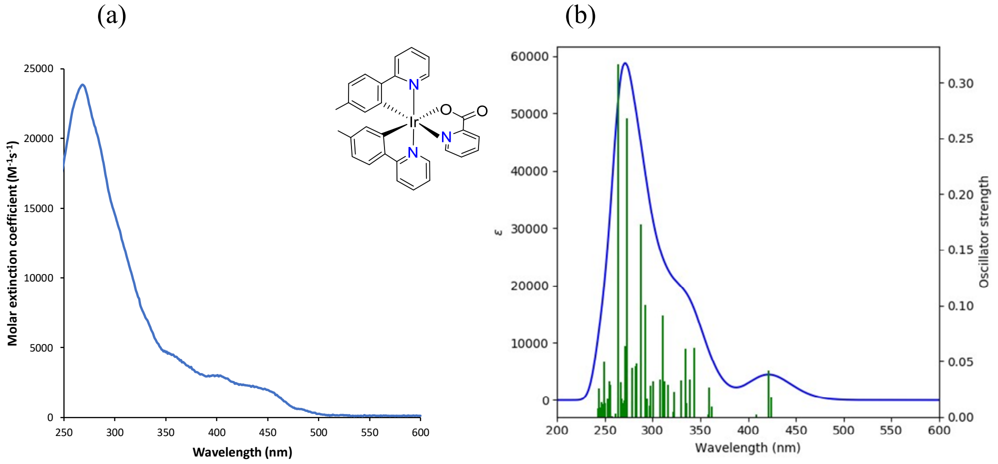

Figure S11. (a) Observed UV-vis spectrum of complex **11b** in 2-MeTHF (5.0 x 10^{-5} M) and (b) calculated (B3LYP(GD3)//SDD(f)/6-31G**) in THF.

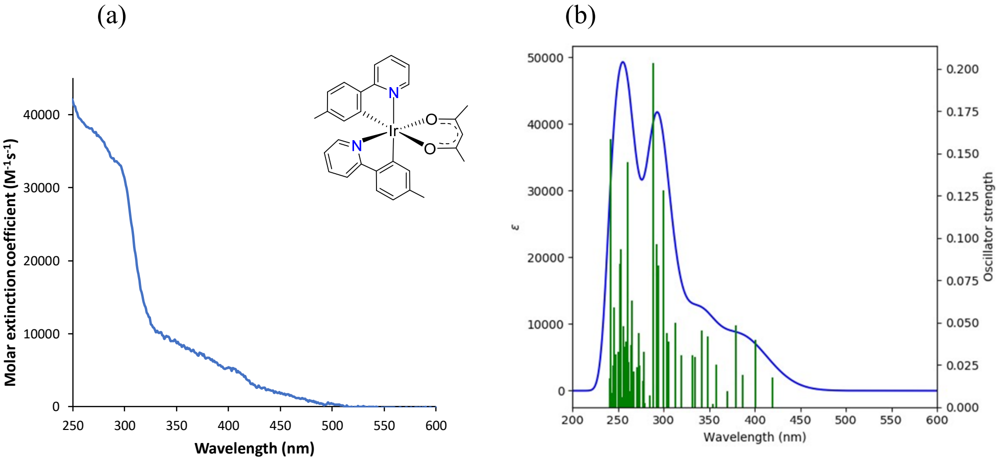

Figure S12. (a) Observed UV-vis spectrum of complex **12a** in 2-MeTHF (1.0 x 10^{-5} M) and (b) calculated (B3LYP(GD3)//SDD(f)/6-31G**) in THF.

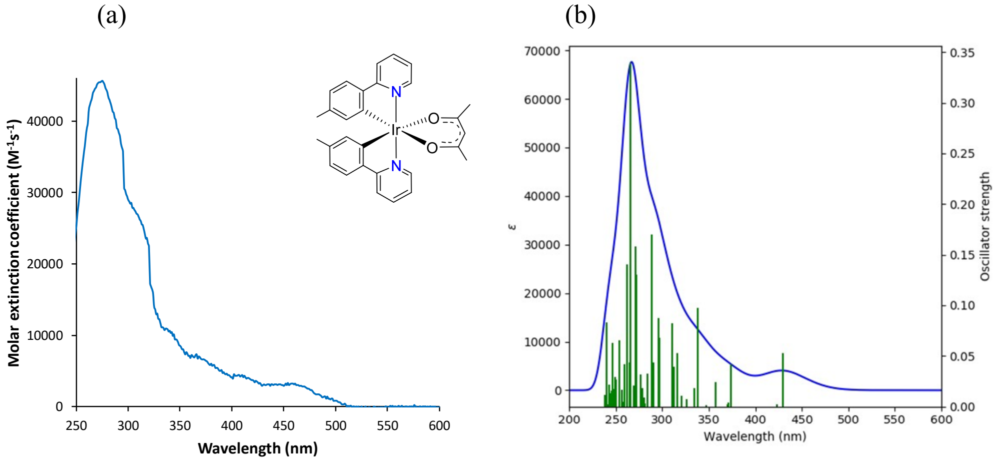

Figure S13. (a) Observed UV-vis spectrum of complex **12b** in 2-MeTHF (1.0 x 10^{-5} M) and (b) calculated (B3LYP(GD3)//SDD(f)/6-31G**) in THF.

• Analysis of Computed UV/Vis Data

Table S1. Selected transitions for the calculated UV spectrum of complex 3 in THF

λ (nm)	Osc. Strength	Symmetry	Major contributions	Minor contributions
454	0	Triplet-A	HOMO → LUMO (60%)	HOMO-5 → LUMO (3%)<br>HOMO-4 → LUMO+1 (4%)<br>HOMO-3 → LUMO+2 (5%)<br>HOMO-2 → LUMO+1 (3%)<br>HOMO-2 → LUMO+2 (6%)<br>HOMO-1 → LUMO+1 (5%)<br>HOMO-1 → LUMO+2 (4%)
408	0.0149	Singlet-A	HOMO → LUMO (97%)	-
385	0.0614	Singlet-A	HOMO-1 → LUMO (93%)	HOMO → LUMO+1 (2%)
384	0.0641	Singlet-A	HOMO-2 → LUMO (93 %)	HOMO → LUMO+2 (2%)
344	0.0795	Singlet-A	HOMO-2 → LUMO+2 (10%)<br>HOMO → LUMO+3 (76%)	HOMO-1 → LUMO+1 (9%)
286	0.0437	Singlet-A	HOMO-3 → LUMO+1 (85%)	HOMO-4 → LUMO+1 (9%)
285	0.0962	Singlet-A	HOMO-4 → LUMO+1 (43%)<br>HOMO-3 → LUMO +2 (48%)	-
282	0.5461	Singlet-A	HOMO-5 → LUMO (67%)	HOMO-4 → LUMO+1 (8%)<br>HOMO-3 → LUMO+2 (8%)

Table S2. Selected transitions for the calculated UV spectrum of complex 9a in THF

λ (nm)	Osc. Strength	Symmetry	Major contributions	Minor contributions
565	0	Triplet-A	HOMO-3 → LUMO (17%)<br>HOMO → LUMO (59%)	HOMO-5 → LUMO (3%)<br>HOMO-5 → LUMO (4%),<br>HOMO-4 → LUMO (2%)<br>HOMO-2 → LUMO (5%)<br>HOMO-1 → LUMO (7%)
486	0.0119	Singlet-A	HOMO → LUMO (94%)	HOMO-2 → LUMO (4%)
440	0.1434	Singlet-A	HOMO-2 → LUMO (93%)	HOMO → LUMO (4%)
408	0.0102	Singlet-A	HOMO → L+1 (95%)	HOMO-1 → LUMO+1 (2%)
384	0.0474	Singlet-A	HOMO-1 → LUMO+1 (77%)	HOMO-2 → LUMO+1 (4%)<br>HOMO-2 → LUMO+2 (8%)<br>HOMO-1 → LUMO+2 (5%)<br>HOMO → LUMO+1 (3%)
352	0.0355	Singlet-A	HOMO-1 → LUMO+2 (13%)<br>HOMO → LUMO+3 (72%)	HOMO-5 → LUMO (2%)<br>HOMO-4 → LUMO (2%)<br>HOMO-2 → LUMO+1 (5%)
297	0.0585	Singlet-A	HOMO-4 → LUMO+1 (82%)	HOMO-4 → LUMO+2 (7%)<br>HOMO-3 → LUMO+1 (2%)
295	0.0732	Singlet-A	HOMO-3 → LUMO+2 (86%)	HOMO-4 → LUMO+2 (4%)
293	0.1866	Singlet-A	HOMO-4 → LUMO+2 (79%)	HOMO-7 → LUMO (3%)<br>HOMO-4 → LUMO+1 (7%)

Table S3. Selected transitions for the calculated UV spectrum of complex 9b in THF

λ (nm)	Osc. Strength	Symmetry	Major contributions	Minor contributions
506	0	Triplet-A	HOMO → LUMO (84%)	HOMO-1 →LUMO (5%)
444	0.0625	Singlet-A	HOMO-1 → LUMO (97%)	-
387	0.0819	Singlet-A	HOMO-3 → LUMO (67%)<br>HOMO-2 → LUMO (23%)	HOMO-5 →LUMO (4%)
328	0.0841	Singlet-A	HOMO-6 →LUMO (62%)<br>HOMO-1 → LUMO+3 (18%)	HOMO-5 →LUMO (2%)<br>HOMO-3 → LUMO+2 (7%)
316	0.0356	Singlet-A	HOMO-4 → LUMO+1 (12%)<br>HOMO-1 → LUMO+5 (73%)	HOMO-4 → LUMO+2 (6%)
315	0.0321	Singlet-A	HOMO-1 → LUMO+4 (89%)	-
285	0.1164	Singlet-A	HOMO-7 → LUMO+1 (11%)<br>HOMO-6 → LUMO+1 (19%)<br>HOMO-3 → LUMO+5 (42%)	HOMO-3 → LUMO+4 (5%)<br>HOMO-2 → LUMO+4 (8%)<br>HOMO-2 → LUMO+5 (2%)
281	0.0406	Singlet-A	HOMO-7 → LUMO+1 (40%)<br>HOMO-4 → LUMO+3 (33%)	HOMO-5 → LUMO+3 (3%)<br>HOMO-4 → LUMO+4 (4%)<br>HOMO-2 → LUMO+4 (3%)<br>HOMO-2 → LUMO+5 (3%)

Table S4. Selected transitions for the calculated UV spectrum of complex 10a in THF

λ (nm)	Osc. Strength	Symmetry	Major contributions	Minor contributions
458	0	Triplet-A	HOMO-2 → LUMO+1 (11%)<br>HOMO → LUMO (60%)	HOMO-5 → LUMO (3%)<br>HOMO-4 → LUMO+1 (4%)<br>HOMO-3 → LUMO+2 (4%)<br>HOMO-2 → LUMO (2%)<br>HOMO-1 → LUMO+2 (6%)
413	0.0136	Singlet-A	HOMO → LUMO (95%)	-
389	0.0337	Singlet-A	HOMO-2 → LUMO (37%)<br>HOMO-1 → LUMO (49%)	HOMO-2 → LUMO+1 (5%)<br>HOMO-1 → LUMO+1 (5%)
387	0.0648	Singlet-A	HOMO-2 → LUMO (52%)<br>HOMO-1 → LUMO (39%)	HOMO-1 → LUMO+1 (2%)
375	0.0605	Singlet-A	HOMO-2 → LUMO+1 (14%)<br>HOMO-2 → LUMO+2 (29%)<br>HOMO-1 → LUMO+1 (20%)<br>HOMO-1 → LUMO+2 (31%)	HOMO-2 → LUMO (2%)
291	0.0779	Singlet-A	HOMO-4 → LUMO+2 (88%)	HOMO-3 → LUMO+2 (2%)<br>HOMO-3 → LUMO+3 (4%)
285	0.2592	Singlet-A	HOMO-5 → LUMO (87%)	-

Table S5. Selected transitions for the calculated UV spectrum of complex 10b in THF

λ (nm)	Osc. Strength	Symmetry	Major contributions	Minor contributions
460	0	Triplet-A	HOMO → LUMO+1 (24%)<br>HOMO → LUMO+2 (47%)	HOMO-4 → LUMO+2 (4%)<br>HOMO-2 → LUMO (8%)<br>HOMO-2 → LUMO+1 (4%)
430	0.0427	Singlet-A	HOMO → LUMO (81 %)<br>HOMO → LUMO+1 (16%)	-
412	0.0158	Singlet-A	HOMO → LUMO+2 (94%)	HOMO-1 → LUMO+2 (3%)
384	0.0225	Singlet-A	HOMO-1 → LUMO+1 (96%)	-
362	0.0700	Singlet-A	HOMO-1 → LUMO+2 (87%)	HOMO → LUMO+2 (3%)<br>HOMO → LUMO+3 (2%)
328	0.0304	Singlet-A	HOMO-3 → LUMO+1 (54%)<br>HOMO-2 → LUMO+1 (17%)	HOMO-3 → LUMO (7%)<br>HOMO-3 → LUMO+2 (6%)<br>HOMO-1 → LUMO+3 (4%)
281	0.0760	Singlet-A	HOMO-7 → LUMO (12%)<br>HOMO-6 → LUMO+2 (10%)<br>HOMO-3 → LUMO+4 (26%)<br>HOMO-3 → LUMO+5 (14%)<br>HOMO-2 → LUMO+5 (21%)	HOMO-7 → LUMO+1 (3%)
277	0.0174	Singlet-A	HOMO-7 → LUMO (15%)<br>HOMO-7 → LUMO+1 (42%)<br>HOMO-7 → LUMO+2 (18%)	HOMO-5 → LUMO+3 (2%)<br>HOMO-4 → LUMO+3 (5%)<br>HOMO → LUMO+6 (7%)
274	0.0466	Singlet-A	HOMO-7 → LUMO+1 (26%)<br>HOMO-7 → LUMO+2 (48%)	HOMO-7 → LUMO (4%)<br>HOMO-4 → LUMO+3 (3%)<br>HOMO-3 → LUMO+5 (6%)

Table S6. Selected transitions for the calculated UV spectrum of complex 11a in THF

λ (nm)	Osc. Strength	Symmetry	Major contributions	Minor contributions
465	0	Triplet-A	HOMO → LUMO (14%)<br>HOMO → LUMO+1 (57%)	HOMO-4 → LUMO+1 (5%)<br>HOMO-3 → LUMO+1 (3%)<br>HOMO-1 → LUMO+1 (2%)<br>HOMO → LUMO+2 (4%)
412	0.0195	Singlet-A	HOMO → LUMO (95%)	-
404	0.0168	Singlet-A	HOMO → LUMO+1 (93%)	HOMO-1 → LUMO (2%)
359	0.0172	Singlet-A	HOMO-2 → LUMO+1 (23%)<br>HOMO-1 → LUMO+2 (62%)	HOMO-1 → LUMO+1 (4%)<br>HOMO → LUMO+3 (6%)
350	0.0553	Singlet-A	HOMO-2 → LUMO+2 (15%)<br>HOMO → LUMO+3 (73%)	HOMO-1 → LUMO+1 (6%)
330	0.0339	Singlet-A	HOMO → LUMO+4 (93%)	-
287	0.0879	Singlet-A	HOMO-4 → LUMO+2 (73%)	HOMO-4 → LUMO+1 (5%)<br>HOMO-3 → LUMO+3 (6%)<br>HOMO-2 → LUMO+5 (8%)
280	0.0421	Singlet-A	HOMO-3 → LUMO+3 (83%)	HOMO-4 → LUMO+2 (8%)

Table S7. Selected transitions for the calculated UV spectrum of complex 11b in THF

λ (nm)	Osc. Strength	Symmetry	Major contributions	Minor contributions
470	0	Triplet-A	HOMO → LUMO (18%)<br>HOMO → LUMO+1 (58%)	HOMO-2 → LUMO+1 (3%)<br>HOMO-2 → LUMO+2 (6%)<br>HOMO-1 → LUMO+2 (4%)
421	0.0413	Singlet-A	HOMO → LUMO+1 (97%)	-
359	0.0266	Singlet-A	HOMO-1 → LUMO (93%)	HOMO-2 → LUMO (5%)
344	0.0618	Singlet-A	HOMO-1 → LUMO+2 (59%)<br>HOMO → LUMO+4 (24%)	HOMO-3 → LUMO (2%)<br>HOMO-2 → LUMO (2%)<br>HOMO-1 → LUMO+1 (7%)
334	0.0611	Singlet-A	HOMO-3 → LUMO (27%)<br>HOMO-2 → LUMO (23%)<br>HOMO → LUMO+5 (19%)	HOMO-3 → LUMO+1 (8%)<br>HOMO-3 → LUMO+2 (6%)<br>HOMO-2 → LUMO+1 (7%)<br>HOMO-1 → LUMO+2 (3%)
322	0.0225	Singlet-A	HOMO-2 → LUMO+1 (49%)<br>HOMO-2 → LUMO+2 (12%)	HOMO-6 → LUMO (2%)<br>HOMO-3 → LUMO+1 (4%)<br>HOMO-3 → LUMO+2 (6%)<br>HOMO-2 → LUMO (8%)<br>HOMO-1 → LUMO+1 (5%)<br>HOMO-1 → LUMO+3 (2%)
264	0.0170	Singlet-A	HOMO-6 → LUMO+3 (15%)<br>HOMO-5 → LUMO+3 (52%)	HOMO-6 → LUMO+2 (9%)<br>HOMO-6 → LUMO+5 (4%)<br>HOMO-3 → LUMO+5 (3%)<br>HOMO-2 → LUMO+5 (5%)<br>HOMO → LUMO+7 (3%)

Table S8. Selected transitions for the calculated UV spectrum of complex 12a in THF

λ (nm)	Osc. Strength	Symmetry	Major contributions	Minor contributions
474	0	Triplet-A	HOMO → LUMO (67%)<br>HOMO → LUMO+1 (12%)	HOMO-4 → LUMO (2%)<br>HOMO-3 → LUMO (5%)<br>HOMO-2 → LUMO (3%)
419	0.0173	Singlet-A	HOMO → LUMO (90%)	HOMO → LUMO+1 (8%)
401	0.0396	Singlet-A	HOMO → LUMO+1 (87%)	HOMO-1 → LUMO (3%)<br>HOMO → LUMO (7%)
379	0.0482	Singlet-A	HOMO-1 → LUMO (43%)<br>HOMO-1 → LUMO+1 (43%)	HOMO-2 → LUMO (4%)<br>HOMO-2 → LUMO+1 (4%)
358	0.0249	Singlet-A	HOMO-2 → LUMO (46%)<br>HOMO-1 → LUMO+2 (35%)	HOMO-2 → LUMO+1 (4%)<br>HOMO-1 → LUMO (2%)<br>HOMO → LUMO+3 (4%)
342	0.0454	Singlet-A	HOMO-2 → LUMO+1 (70%)<br>HOMO-1 → LUMO+3 (12%)	HOMO-2 → LUMO (4%)<br>HOMO → LUMO+3 (5%)
332	0.0305	Singlet-A	HOMO-1 → LUMO+3 (26%)<br>HOMO → LUMO+4 (60%)	HOMO-2 → LUMO+1 (2%)<br>HOMO-2 → LUMO+2 (2%)<br>HOMO-1 → LUMO+2 (3%)
304	0.0439	Singlet-A	HOMO-3 → LUMO (44%)<br>HOMO-2 → LUMO+3 (45%)	HOMO-3 → LUMO+1 (3%)
299	0.1281	Singlet-A	HOMO-3 → LUMO+1 (81%)	HOMO-2 → LUMO+1 (2%)<br>HOMO-2 → LUMO+4 (9%)

Table S9. Selected transitions for the calculated UV spectrum of complex 12b in THF

λ (nm)	Osc. Strength	Symmetry	Major contributions	Minor contributions
475	0	Triplet-A	HOMO-3 → LUMO+1 (10%)<br>HOMO → LUMO (51%)<br>HOMO → LUMO+1 (28%)	-
430	0.0527	Singlet-A	HOMO → LUMO (91%)	HOMO → L+1 (6%)
374	0.0420	Singlet-A	HOMO-1 → LUMO (11%)<br>HOMO-1 → LUMO+1 (83%)	-
357	0.0242	Singlet-A	HOMO → LUMO+3 (96%)	-
338	0.0974	Singlet-A	HOMO-2 → LUMO (82%)	HOMO-4 → LUMO (3%)<br>HOMO-2 → LUMO+1 (5%)<br>HOMO-1 → LUMO+1 (2%)<br>HOMO-1 → LUMO+2 (4%)
334	0.0180	Singlet-A	HOMO-2 → LUMO+1 (78%)	HOMO-4 → LUMO+1 (4%)<br>HOMO-2 → LUMO (5%)<br>HOMO-1 → LUMO (3%)<br>HOMO-1 → LUMO+3 (3%)<br>HOMO → LUMO+4 (3%)
276	0.0320	Singlet-A	HOMO-7 → LUMO+2 (27%)<br>HOMO-3 → LUMO+2 (44%)	HOMO-6 → LUMO+1 (8%)<br>HOMO-5 → LUMO+2 (7%)<br>HOMO-3 → LUMO+4 (4%)<br>HOMO → LUMO+6 (2%)

• Theoretical Analysis of Molecular Orbitals of complex 3 and isomers a and b of complexes 9-12

Energies and population analysis (%) of molecular orbitals are given in Tables S10-S18 whereas Figures S14–S22 collect the molecular orbitals.

Table S10. Composition (%) of molecular orbitals of 3

MO	eV	Ir	Ph-pyMe$_2$ (1)	Ph-pyMe$_2$ (2)	Ph-pyMe$_2$ (3)
L+9	0.72	6	59	6	29
L+8	0.72	6	4	57	33
L+7	0.55	6	30	31	32
L+6	0.18	96	1	1	2
L+5	-0.39	4	58	3	36
L+4	-0.40	4	7	62	28
L+3	-0.66	4	32	32	33
L+2	-0.99	4	62	10	24
L+1	-0.99	4	1	55	39
LUMO	-1.12	2	34	32	33
HOMO	-4.91	53	17	16	15
H-1	-5.03	46	6	21	28
H-2	-5.03	46	30	16	9
H-3	-5.88	6	61	18	15
H-4	-5.89	6	2	45	48
H-5	-6.02	8	30	30	32
H-6	-6.50	17	27	26	30
H-7	-6.60	18	44	28	10
H-8	-6.60	18	12	30	40
H-9	-6.72	16	27	27	30

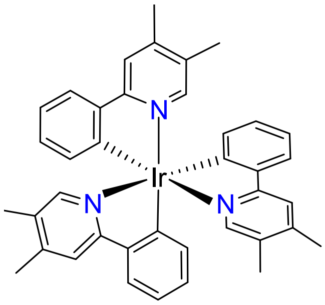

3

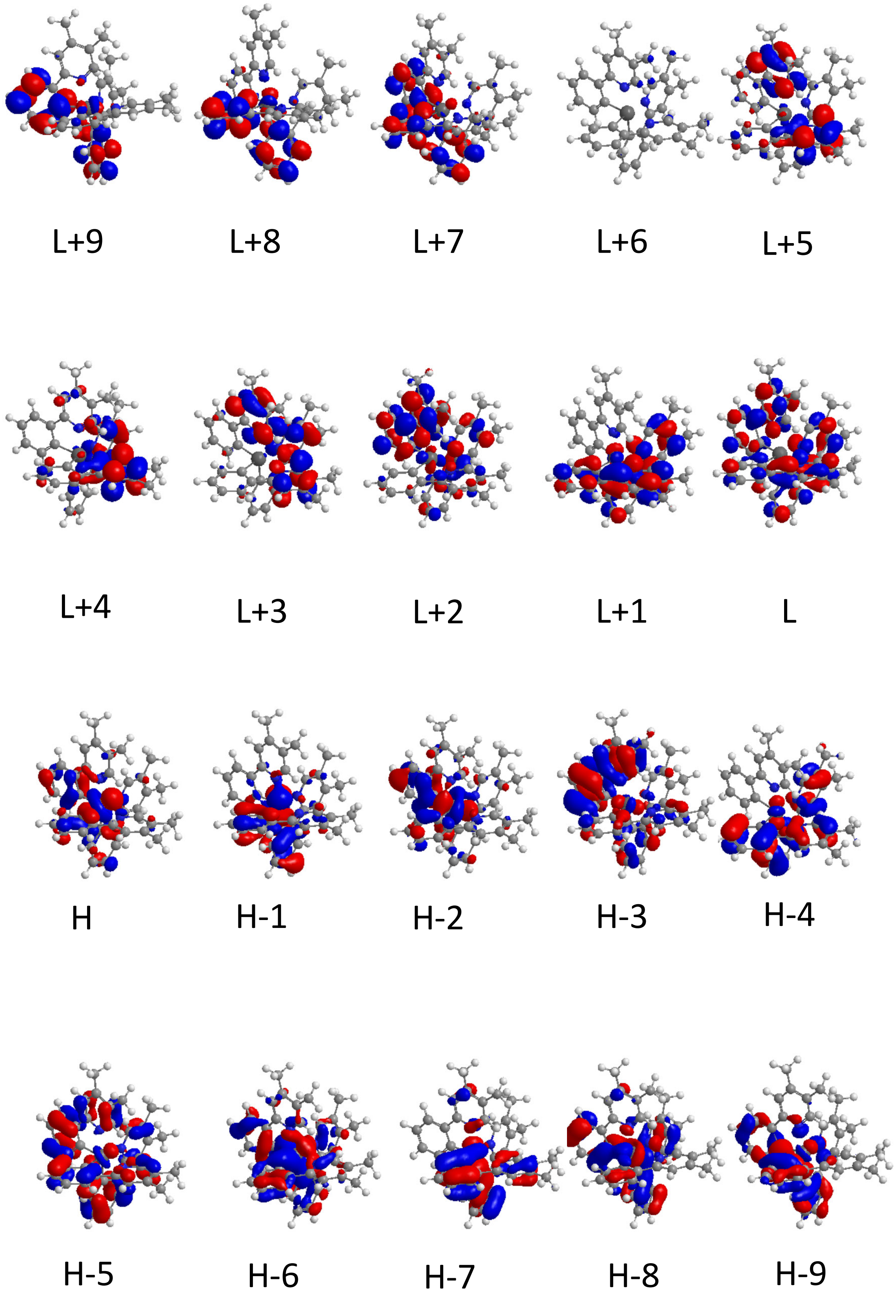

Figure S14. Selected molecular orbitals of complex **3** (isovalue 0.03 au).

Table S11. Composition (%) of molecular orbitals of 9a

MO	eV	Ir	Ph-isoq	*p*-tol-py (1)	*p*-tol-py (2)
L+9	0.61	8	36	21	34
L+8	0.46	8	53	24	15
L+7	0.19	94	4	1	1
L+6	-0.08	3	96	1	0
L+5	-0.56	3	3	38	56
L+4	-0.65	3	50	36	12
L+3	-0.82	3	46	23	28
L+2	-1.09	5	1	37	58
L+1	-1.16	3	2	58	37
LUMO	-1.72	3	94	2	1
HOMO	-4.97	49	15	10	25
H-1	-5.10	43	7	39	10
H-2	-5.15	44	33	5	17
H-3	-5.82	6	54	2	38
H-4	-5.87	7	10	58	25
H-5	-6.00	9	26	32	33
H-6	-6.53	18	29	29	24
H-7	-6.61	14	61	6	19
H-8	-6.73	21	20	41	18
H-9	-6.79	15	33	19	33

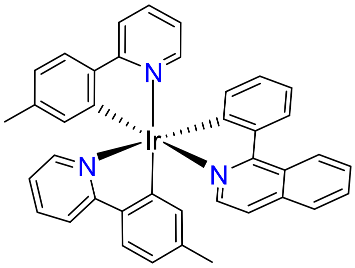

9a

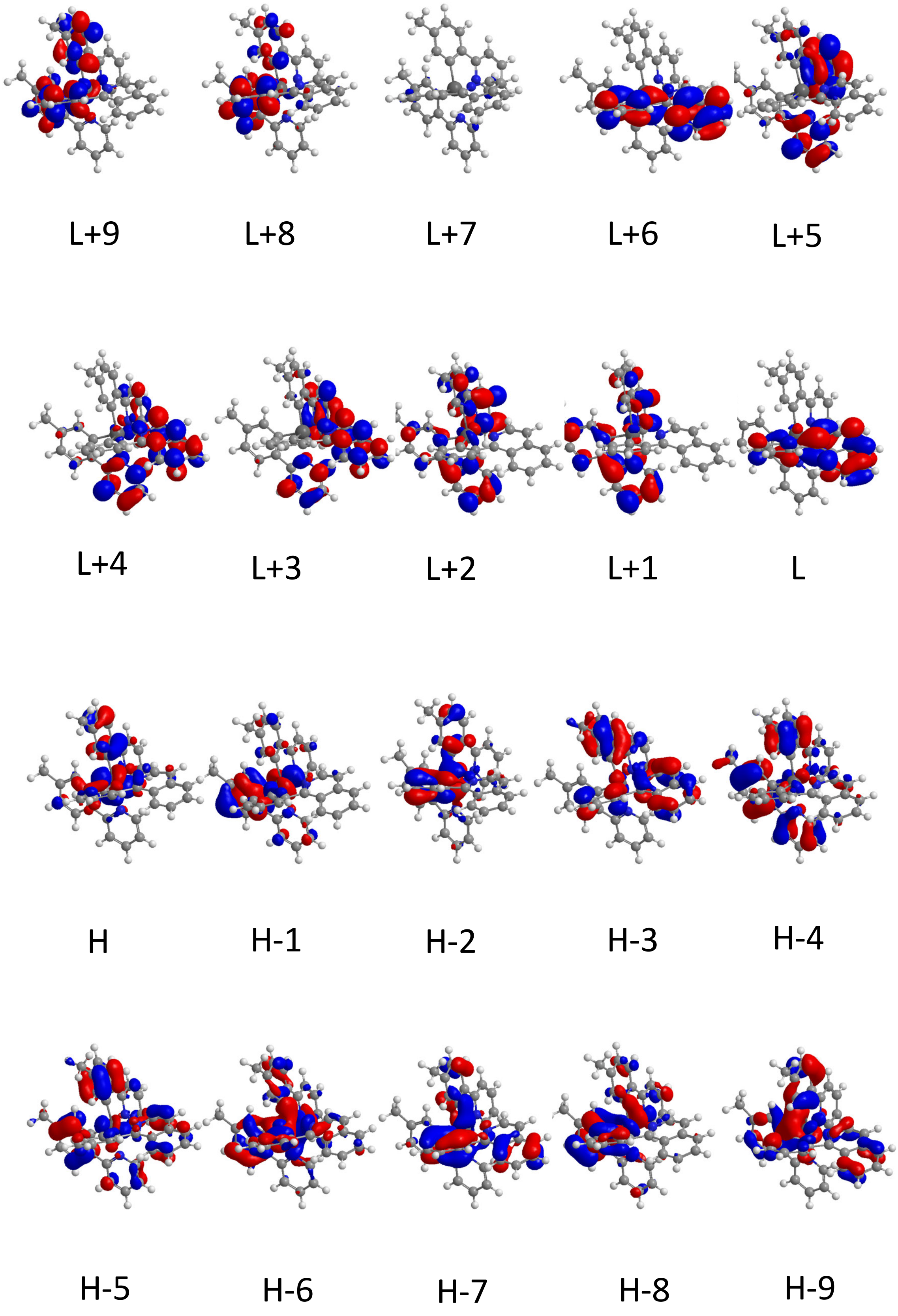

Figure S15. Selected molecular orbitals of complex **9a** (isovalue 0.03 au).

Table S12. Composition (%) of molecular orbitals of 9b

MO	eV	Ir	Ph-isoq	*p*-tol-py (1)	*p*-tol-py (2)
L+9	0.57	4	4	25	67
L+8	0.48	9	78	6	8
L+7	0.19	94	4	1	1
L+6	-0.11	4	94	1	1
L+5	-0.59	3	0	61	36
L+4	-0.61	3	44	21	32
L+3	-0.82	2	55	14	29
L+2	-1.10	4	2	84	11
L+1	-1.20	4	3	9	83
LUMO	-1.74	2	94	2	2
HOMO	-4.86	41	6	33	19
H-1	-5.22	45	41	9	5
H-2	-5.52	32	16	12	40
H-3	-5.61	21	40	7	31
H-4	-5.80	16	14	48	23
H-5	-5.88	10	40	9	42
H-6	-6.03	20	33	23	24
H-7	-6.25	4	6	46	44
H-8	-6.65	14	77	6	4
H-9	-6.83	9	83	5	3

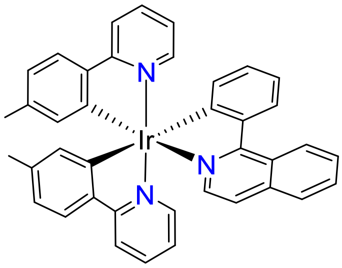

9b

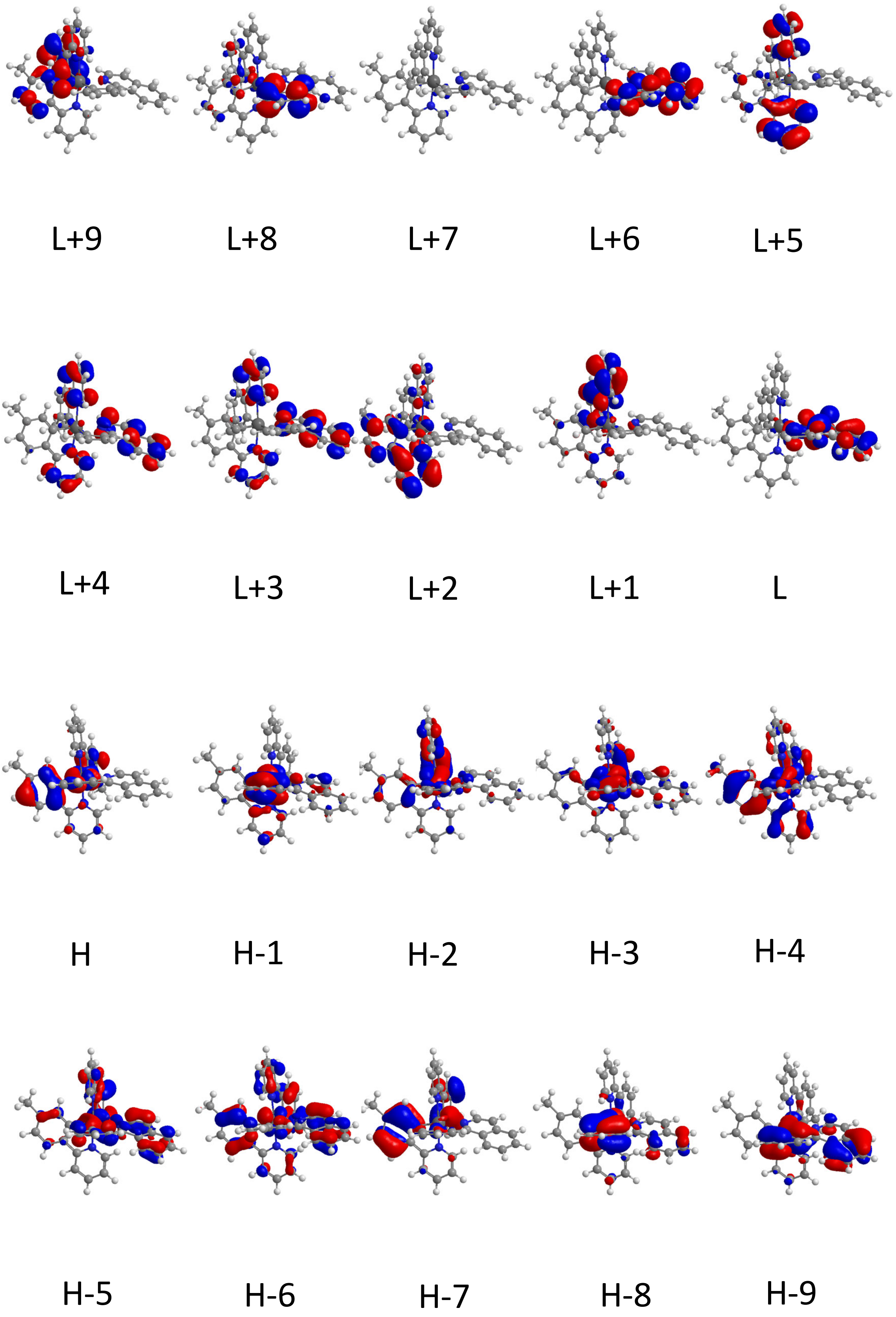

Figure S16. Selected molecular orbitals of complex **9b** (isovalue 0.03 au).

Table S13. Composition (%) of molecular orbitals of 10a

MO	eV	Ir	Ph-py	*p*-tol-py (1)	*p*-tol-py (2)
L+9	0.69	7	1	43	49
L+8	0.66	6	60	20	13
L+7	0.49	8	33	29	31
L+6	0.17	97	1	1	1
L+5	-0.57	3	1	42	54
L+4	-0.58	3	61	24	12
L+3	-0.84	3	35	31	31
L+2	-1.10	5	0	49	46
L+1	-1.13	5	51	21	24
LUMO	-1.23	2	45	26	27
HOMO	-4.99	52	14	18	16
H-1	-5.12	44	5	22	29
H-2	-5.13	45	32	15	8
H-3	-5.84	6	2	48	45
H-4	-5.90	6	11	40	43
H-5	-6.09	6	82	6	6
H-6	-6.58	18	24	28	29
H-7	-6.71	24	6	44	26
H-8	-6.71	24	43	10	23
H-9	-6.79	18	30	25	28

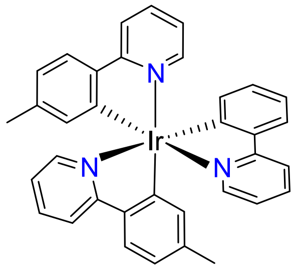

10a

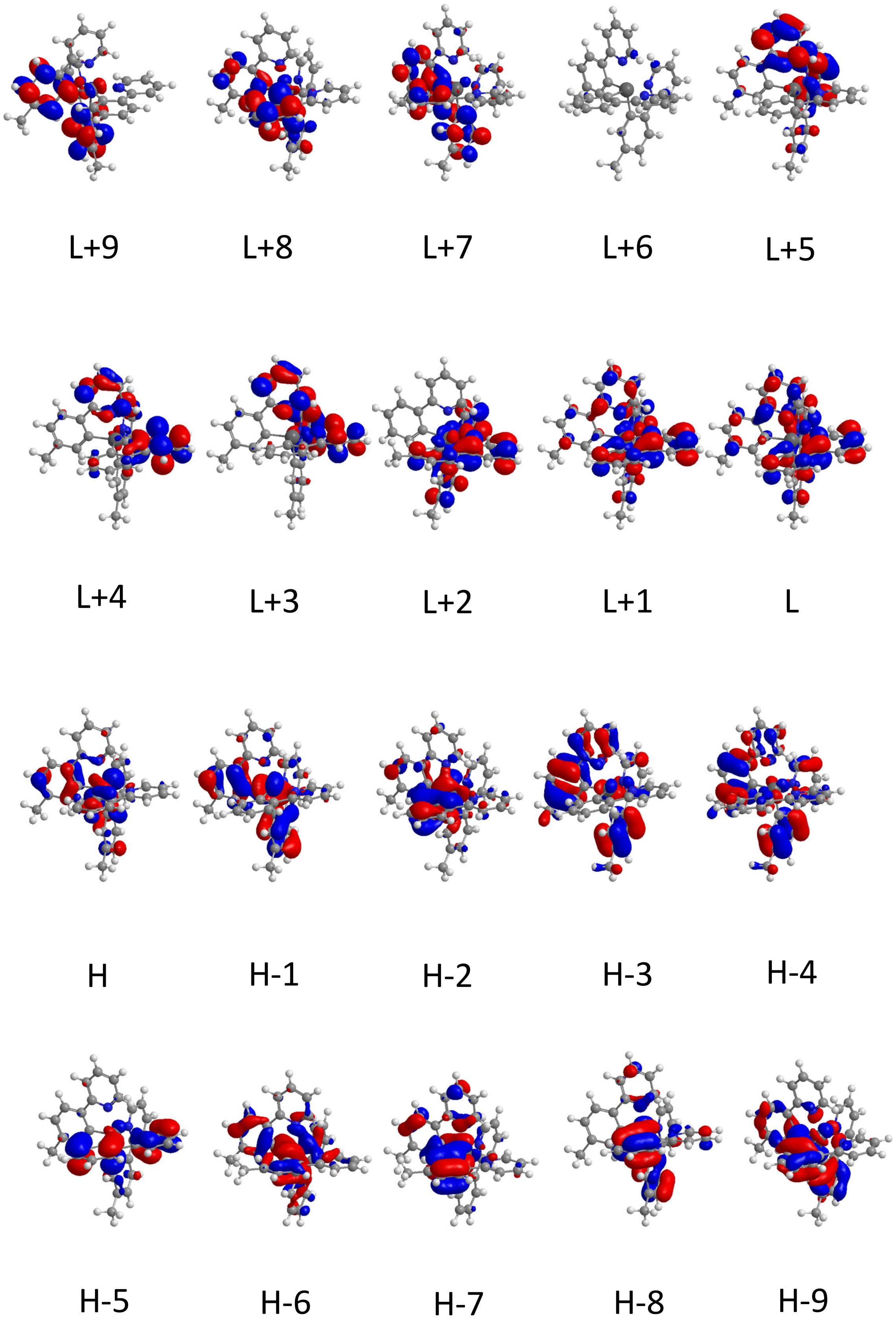

Figure S17. Selected molecular orbitals of complex **10a** (isovalue 0.03 au).

Table S14. Composition (%) of molecular orbitals of 10b

MO	eV	Ir	Ph-py	*p*-tol-py (1)	*p*-tol-py (2)
L+9	0,69	6	14	74	5
L+8	0,58	5	25	16	55
L+7	0,56	7	59	2	32
L+6	0,17	98	0	1	1
L+5	-0,55	3	51	31	15
L+4	-0,6	4	2	44	50
L+3	-0,84	2	45	21	32
L+2	-1,1	4	10	80	6
L+1	-1,19	6	50	1	44
LUMO	-1,25	2	38	14	46
HOMO	-4,86	42	7	32	19
H-1	-5,2	45	37	11	7
H-2	-5,55	27	10	12	50
H-3	-5,68	35	31	6	29
H-4	-5,81	14	11	62	12
H-5	-5,96	12	15	11	62
H-6	-6,11	11	76	9	5
H-7	-6,24	3	6	45	45
H-8	-6,64	24	62	10	4
H-9	-7,08	14	29	53	4

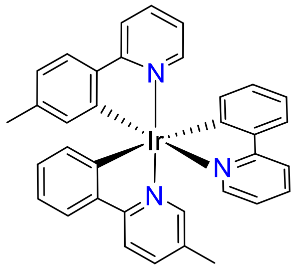

10b

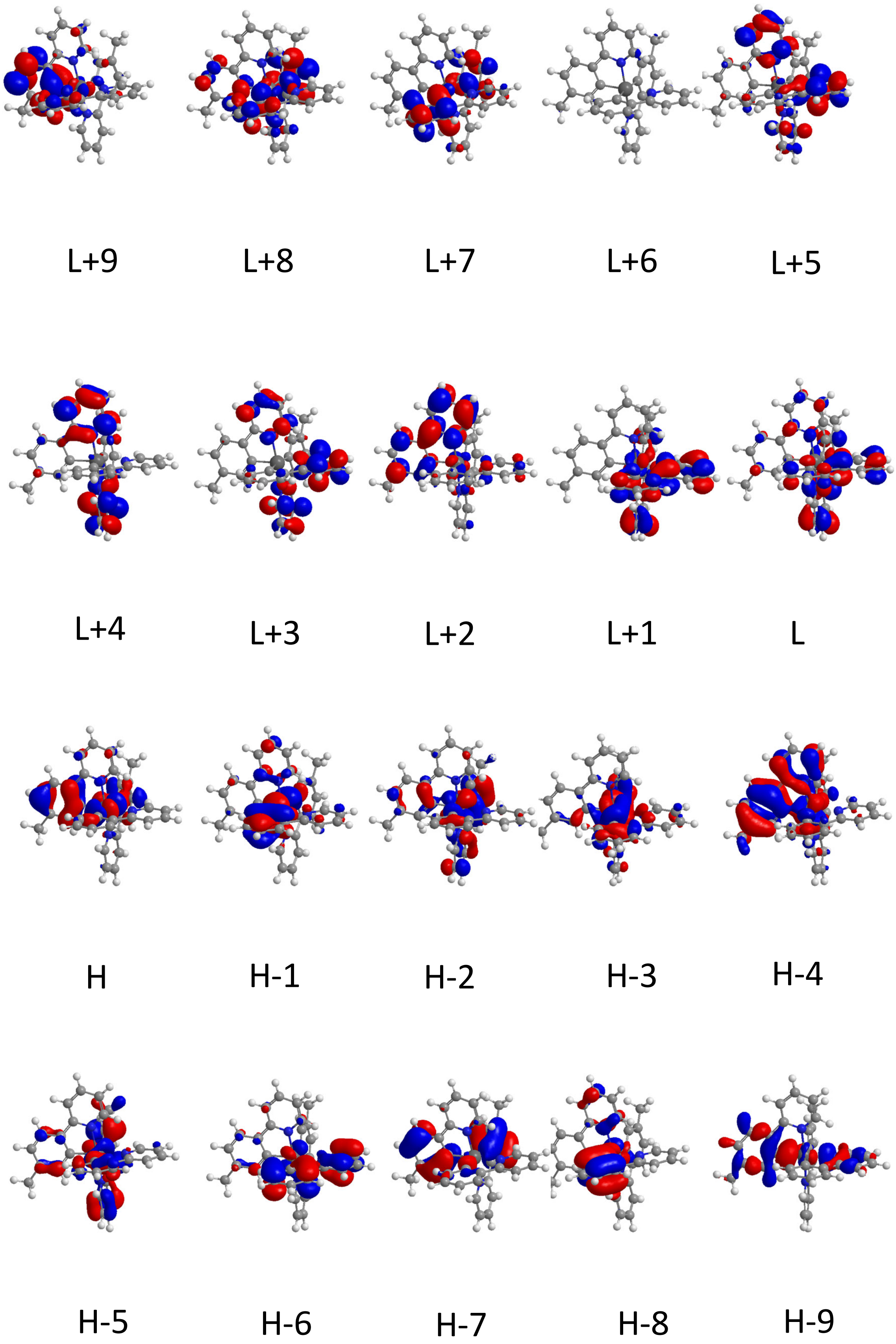

Figure S18. Selected molecular orbitals of complex **10b** (isovalue 0.03 au).

Table S15. Composition (%) of molecular orbitals of 11a

MO	eV	Ir	Picolinate	*p*-tol-py (1)	*p*-tol-py (2)
L+9	0.92	46	8	22	25
L+8	0.58	5	0	24	71
L+7	0.45	10	2	67	22
L+6	0.12	97	1	2	0
L+5	-0.66	3	2	24	72
L+4	-0.75	2	46	44	8
L+3	-0.98	3	50	29	18
L+2	-1.21	4	0	10	86
L+1	-1.32	5	12	74	9
LUMO	-1.45	2	84	10	3
HOMO	-5.19	45	4	27	25
H-1	-5.37	40	5	33	23
H-2	-5.59	65	19	6	9
H-3	-5.93	8	3	18	72
H-4	-6.07	9	1	71	19
H-5	-6.53	6	63	6	25
H-6	-6.81	19	24	28	29
H-7	-6.90	21	27	33	19
H-8	-7.01	16	13	40	31
H-9	-7.29	9	71	2	18

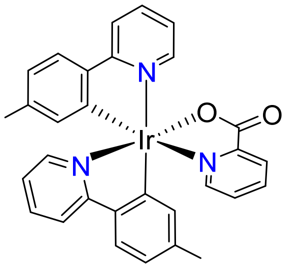

11a

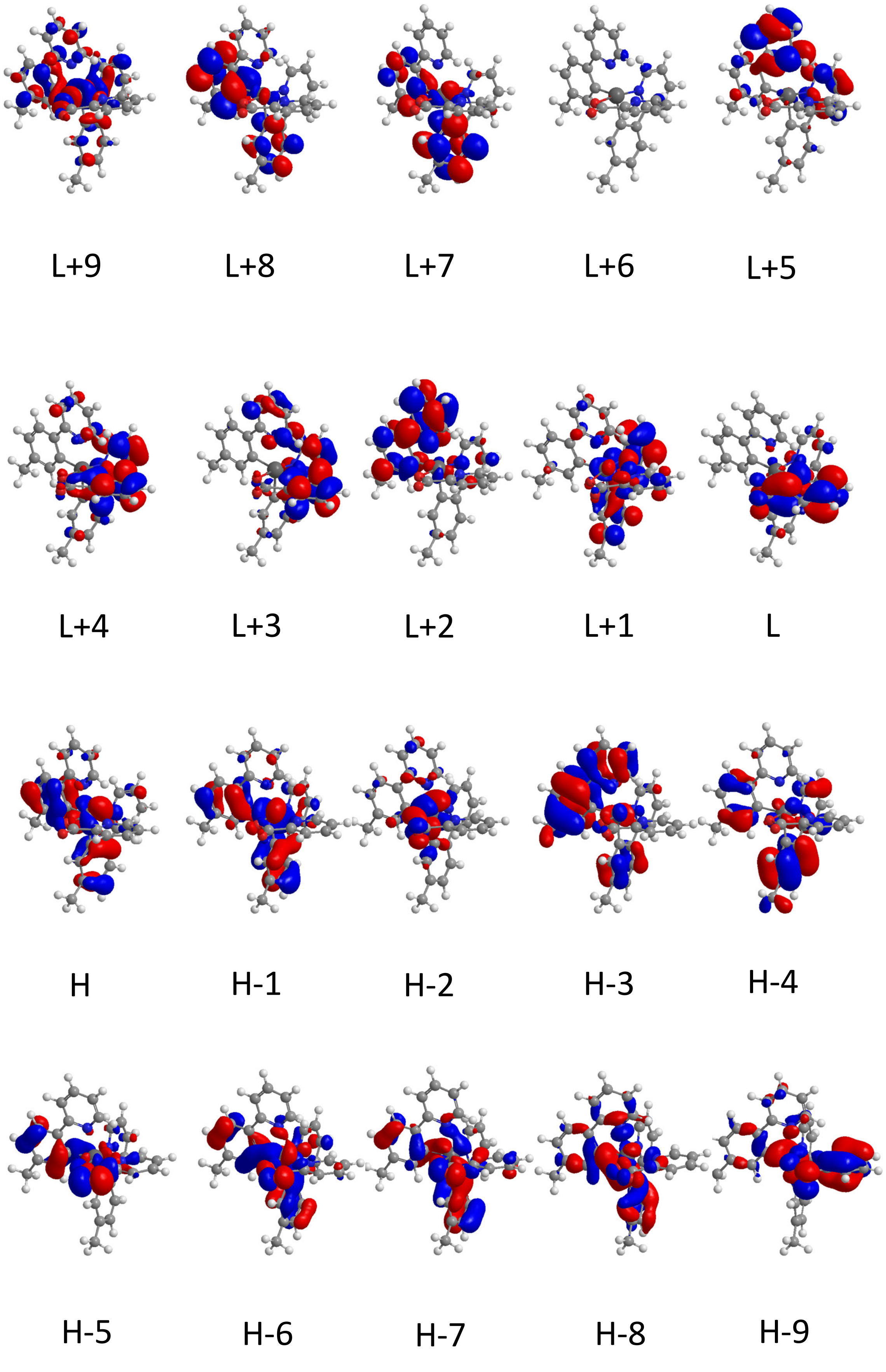

Figure S19. Selected molecular orbitals of complex **11a** (isovalue 0.03 au).

Table S16. Composition (%) of molecular orbitals of 11b

MO	eV	Ir	Picolinate	*p*-tol-py (1)	*p*-tol-py (2)
L+9	0.95	7	1	22	70
L+8	0.53	9	1	66	24
L+7	0.36	6	2	23	68
L+6	0.14	98	0	1	1
L+5	-0.67	3	45	31	22
L+4	-0.71	3	1	48	48
L+3	-0.95	2	52	18	28
L+2	-1.27	5	2	71	22
L+1	-1.33	6	19	16	60
LUMO	-1.41	2	78	8	12
HOMO	-5.05	43	4	27	26
H-1	-5.63	57	15	20	9
H-2	-5.81	20	4	34	42
H-3	-5.91	48	8	26	19
H-4	-6.09	16	3	45	36
H-5	-6.29	5	23	27	45
H-6	-6.50	2	68	12	19
H-7	-7.00	7	59	15	20
H-8	-7.18	9	78	8	5
H-9	-7.30	15	26	54	5

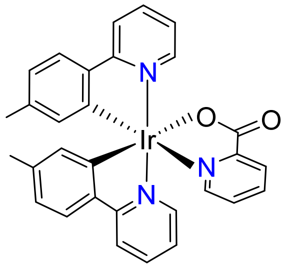

11b

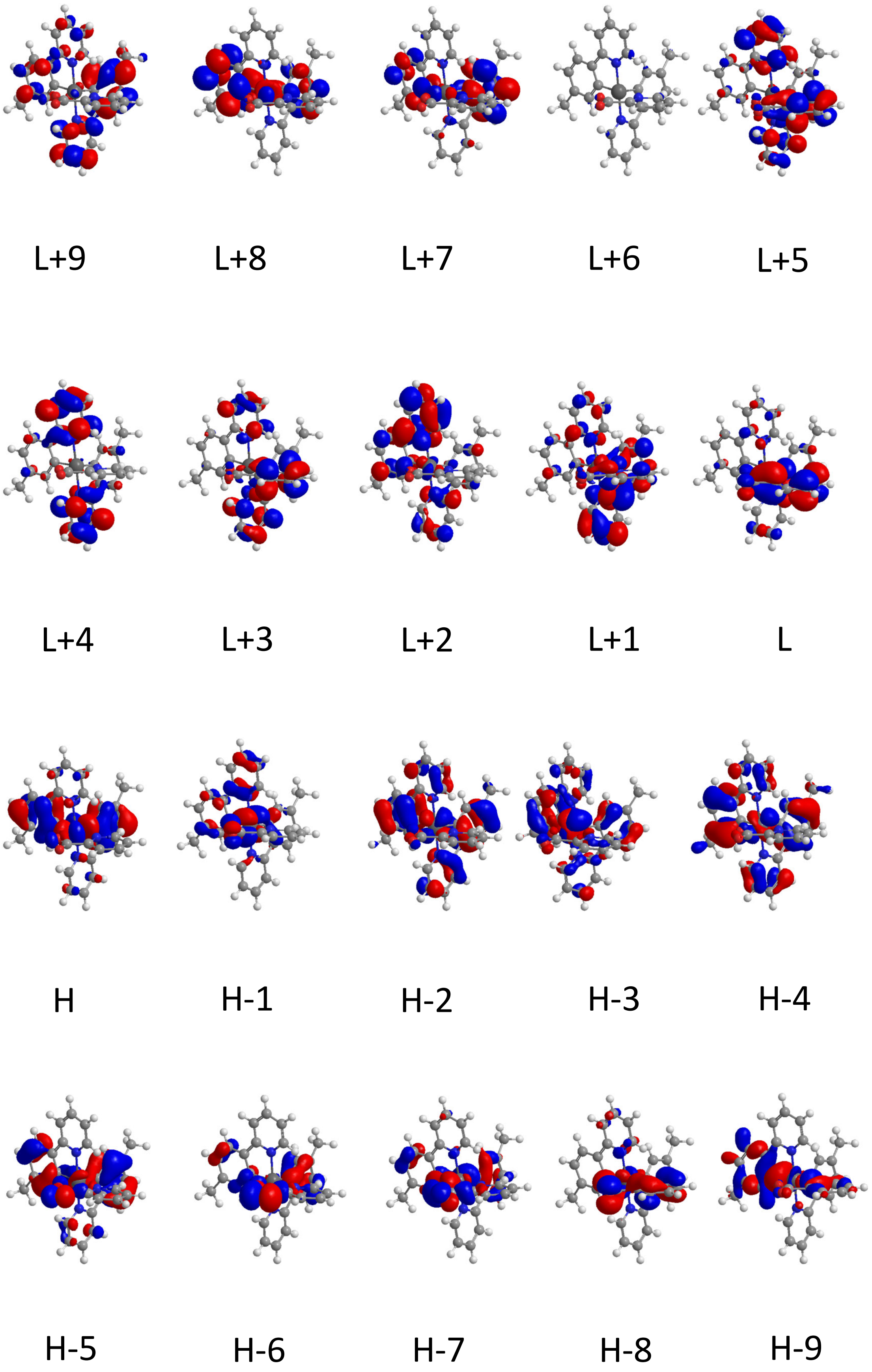

Figure S20. Selected molecular orbitals of complex **11b** (isovalue 0.03 au).

Table S17. Composition (%) of molecular orbitals of 12a

MO	eV	Ir	acac	*p*-tol-py (1)	*p*-tol-py (2)
L+9	1.08	36	4	22	37
L+8	0.98	36	4	39	21
L+7	0.56	5	0	72	22
L+6	0.52	9	1	22	68
L+5	0.09	96	2	0	1
L+4	-0.60	3	0	52	45
L+3	-0.76	3	14	40	44
L+2	-0.85	3	81	8	8
L+1	-1.18	4	2	76	18
LUMO	-1.25	4	1	19	76
HOMO	-5.01	46	7	16	30
H-1	-5.17	46	13	26	15
H-2	-5.53	46	28	8	17
H-3	-5.87	9	4	48	39
H-4	-6.02	11	2	44	43
H-5	-6.29	24	50	8	19
H-6	-6.64	12	20	48	20
H-7	-6.76	11	38	42	9
H-8	-6.97	32	17	8	43
H-9	-7.09	13	10	28	49

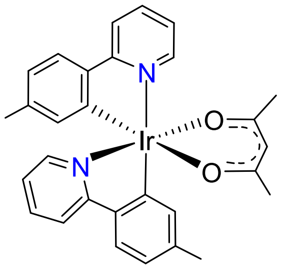

12a

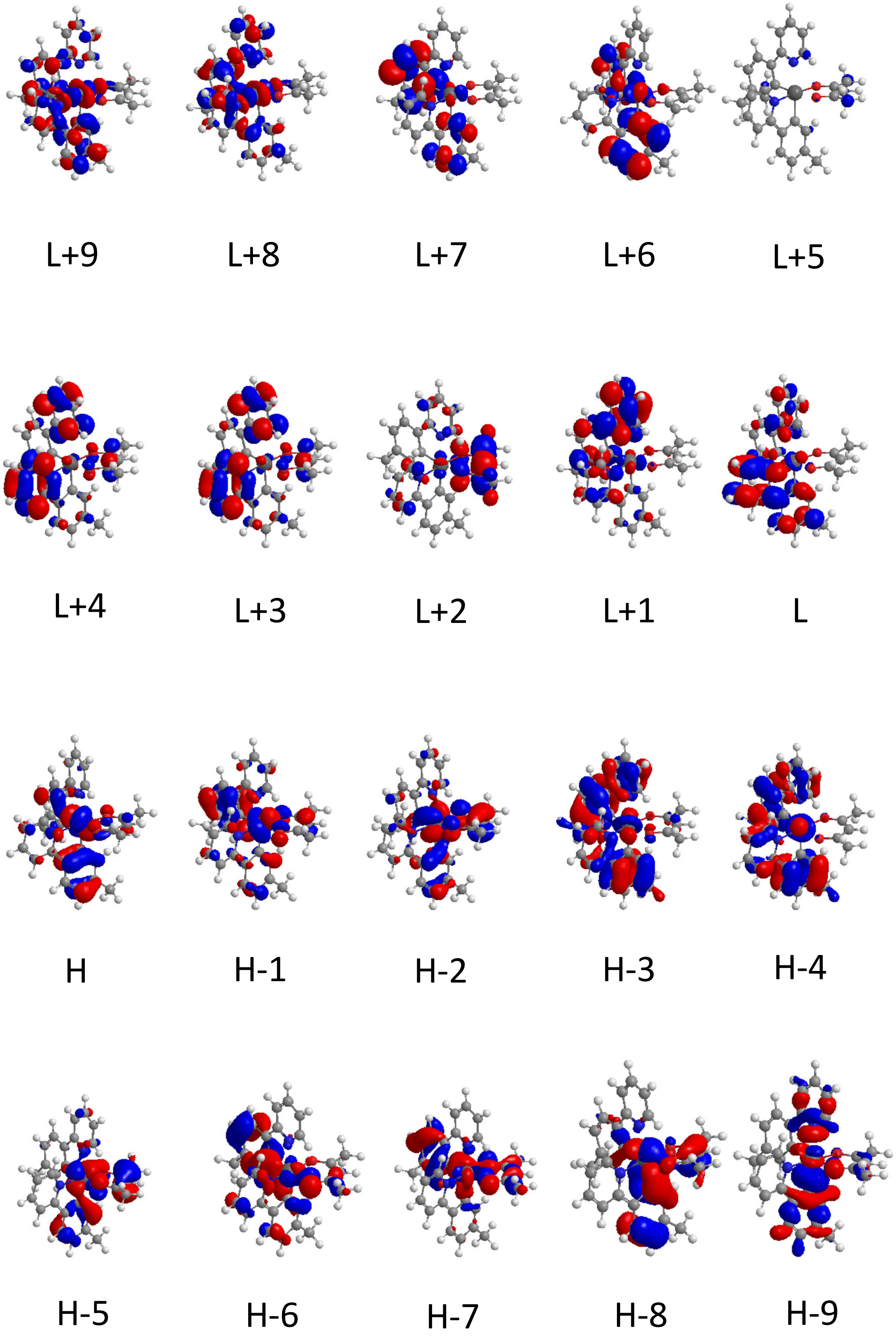

Figure S21. Selected molecular orbitals of complex **12a** (isovalue 0.03 au).

Table S18. Composition (%) of molecular orbitals of 12b

MO	eV	Ir	acac	*p*-tol-py (1)	*p*-tol-py (2)
L+9	1.08	34	2	37	26
L+8	1.01	9	0	41	50
L+7	0.59	9	1	39	50
L+6	0.45	6	1	53	41
L+5	0.09	96	2	1	1
L+4	-0.66	4	3	54	39
L+3	-0.76	3	1	42	54
L+2	-0.78	3	91	2	5
L+1	-1.25	5	2	72	21
LUMO	-1.27	5	1	22	73
HOMO	-4.93	46	4	26	24
H-1	-5.36	45	43	6	6
H-2	-5.75	64	5	15	16
H-3	-5.78	1	3	47	49
H-4	-6.03	16	1	37	46
H-5	-6.18	8	31	34	27
H-6	-6.37	21	29	26	24
H-7	-6.58	9	67	12	12
H-8	-7.12	11	38	25	26
H-9	-7.38	28	8	32	32

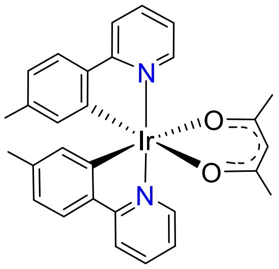

12b

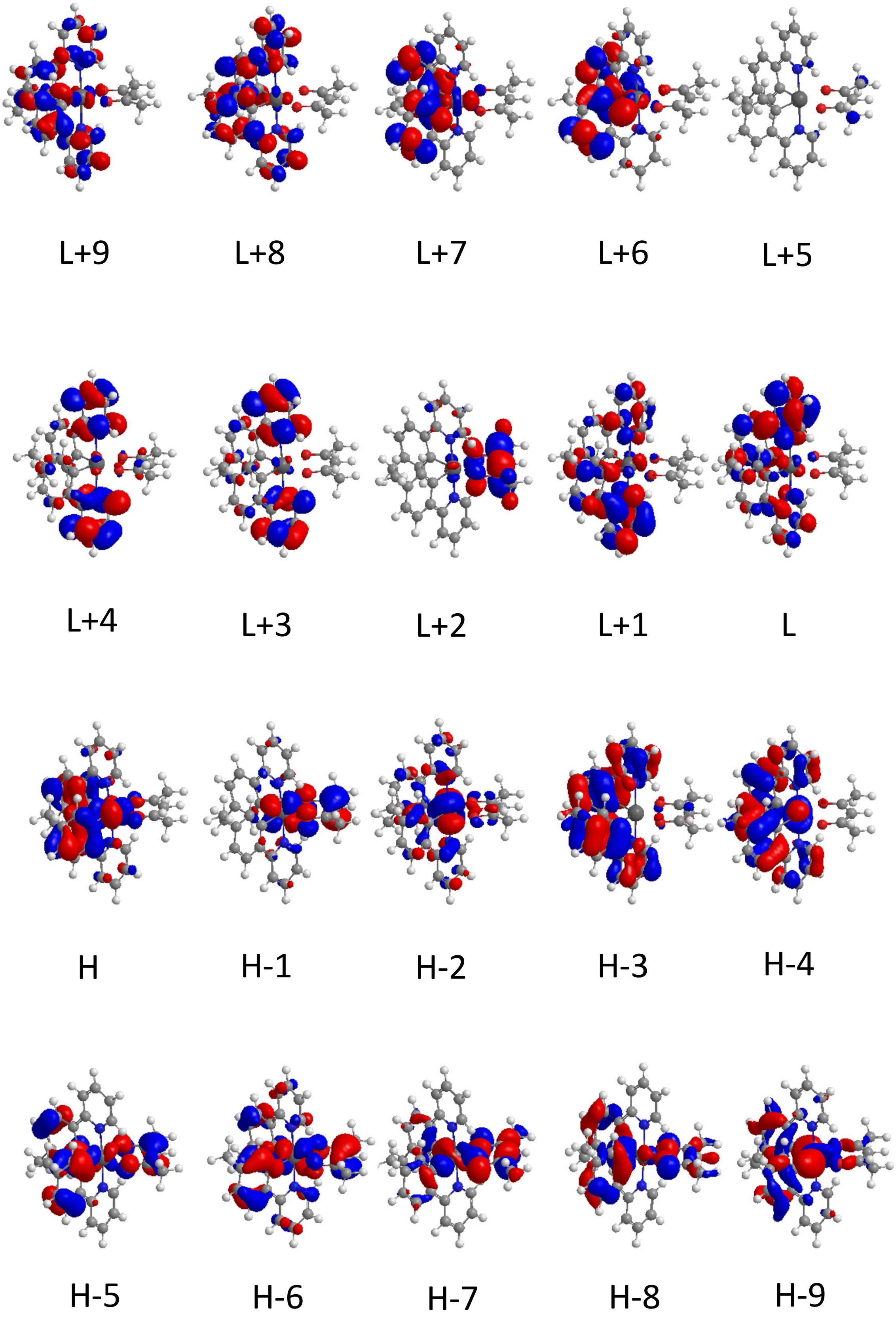

Figure S22. Selected molecular orbitals of complex **12b** (isovalue 0.03 au).

• Cyclic Voltammograms

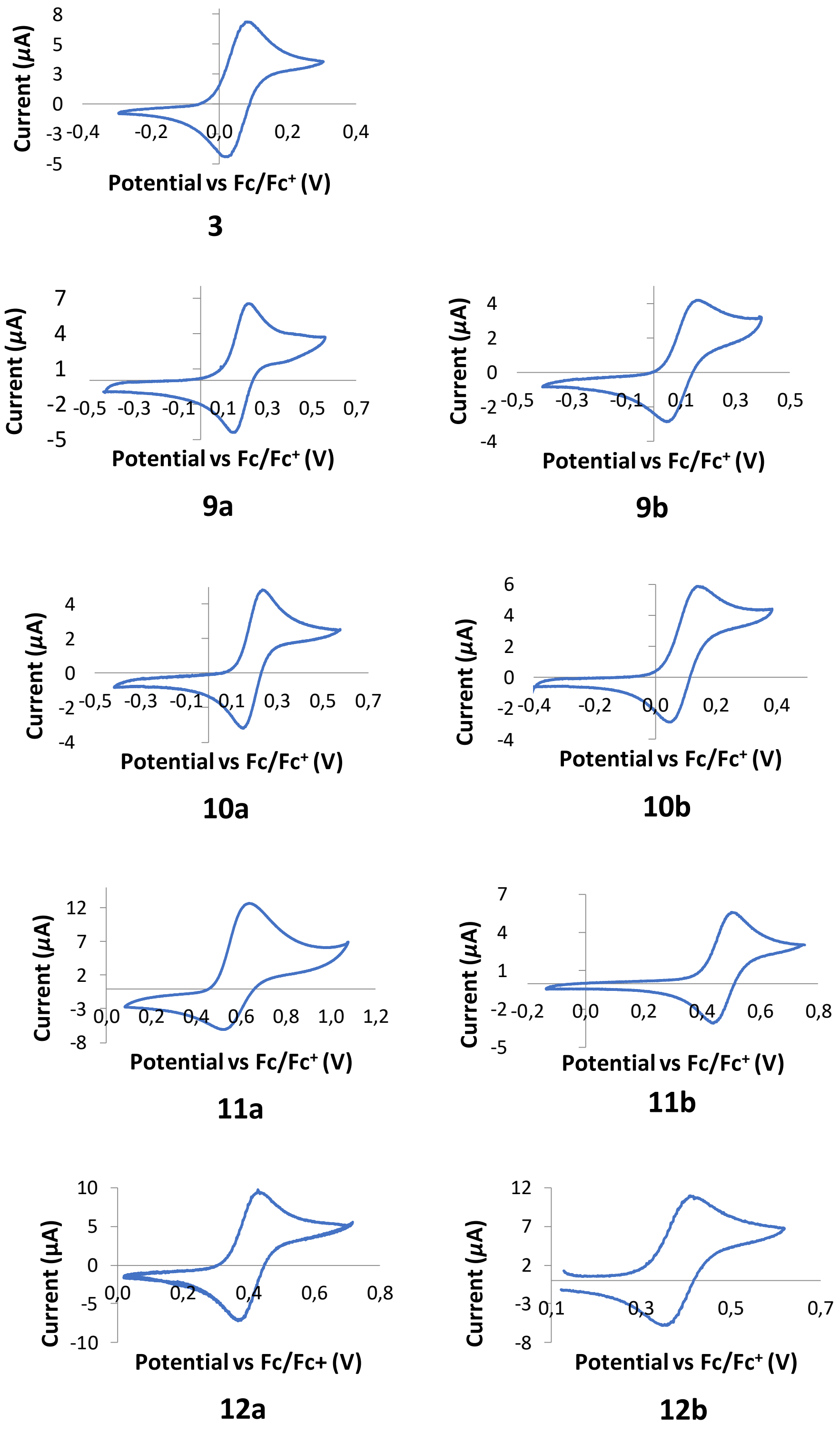

Figure S23. Cyclic voltammograms of complexes **3** and **9-12** in 10^{-3} M solutions with Bu_4NPF_6 as supporting electrolyte (0.1 M) at a scan rate of 100 mV s^{-1}. The potentials were referenced to the ferrocene/ferrocenium (Fc/Fc^+) couple.

• **Photophysical Studies**

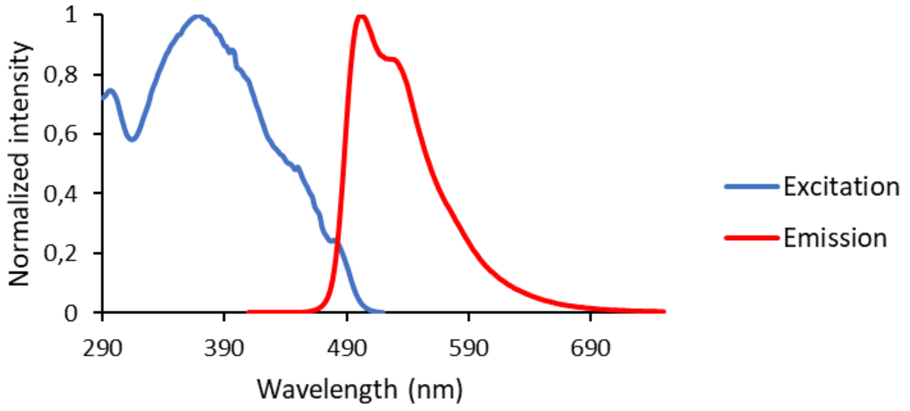

Figure S24. Normalized emission and excitation spectrum of complex **3** in PMMA film (5 wt %) at 298 K.

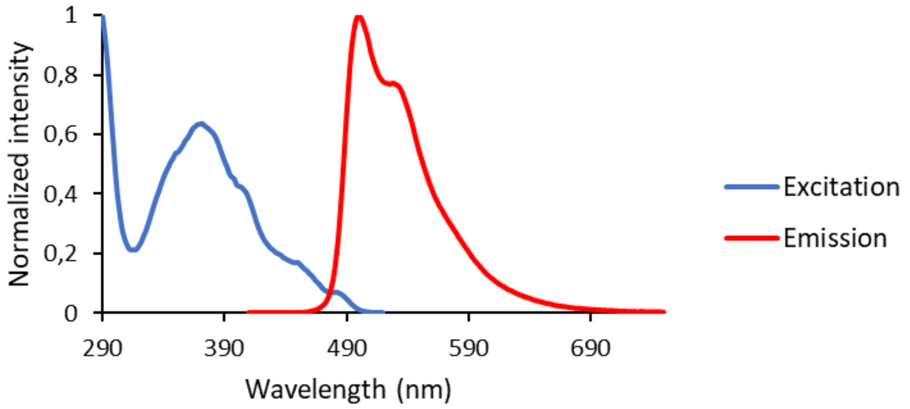

Figure S25. Normalized emission and excitation spectrum for a 1 x 10^{-5} M solution of complex **3** in 2-methyl tetrahydrofuran at 298 K.

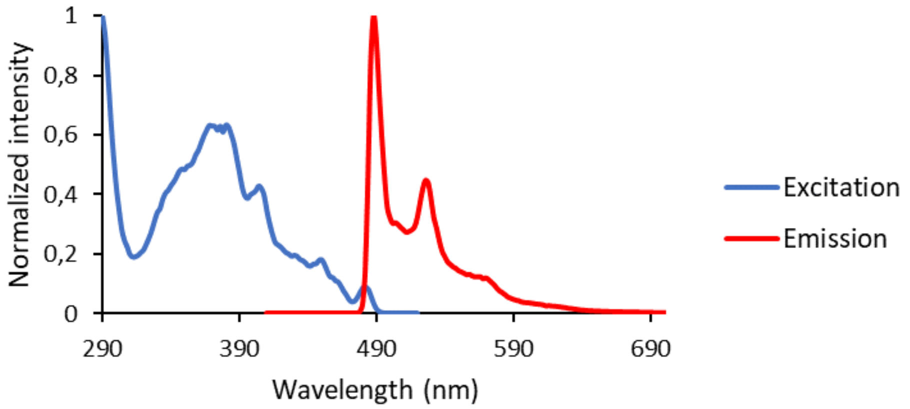

Figure S26. Normalized emission and excitation spectrum for a 1 x 10^{-5} M solution of complex **3** in 2-methyl tetrahydrofuran at 77 K.

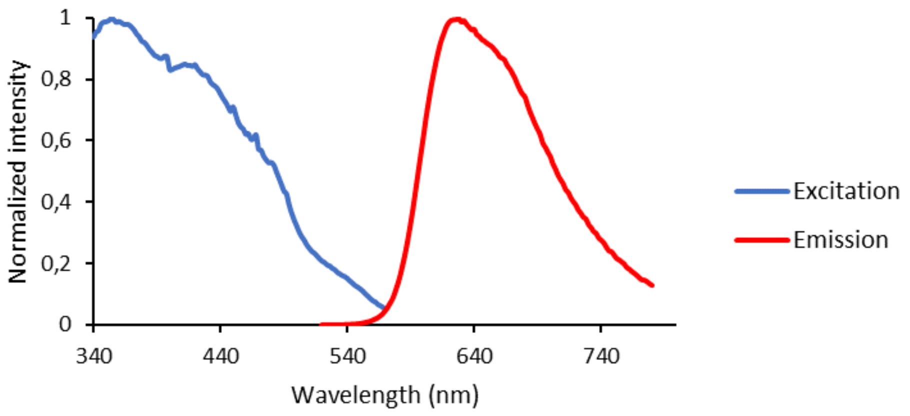

Figure S27. Normalized emission and excitation spectrum of complex **9a** in PMMA film (5 wt %) at 298 K.

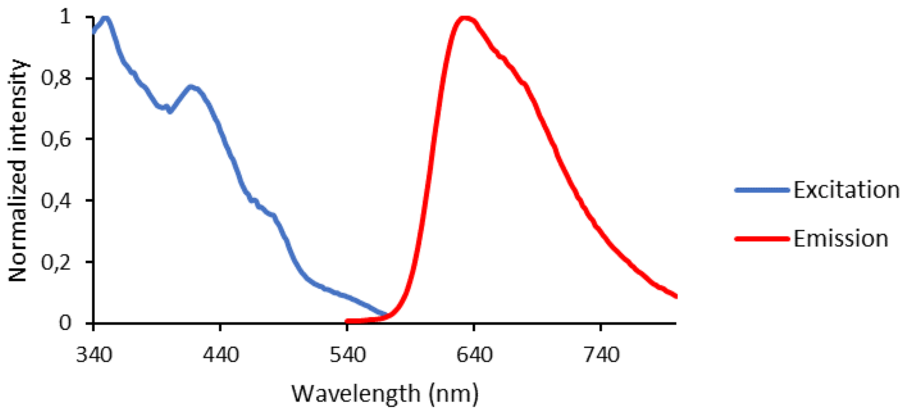

Figure S28. Normalized emission and excitation spectrum for a 1 x 10^{-5} M solution of complex **9a** in 2-methyl tetrahydrofuran at 298 K.

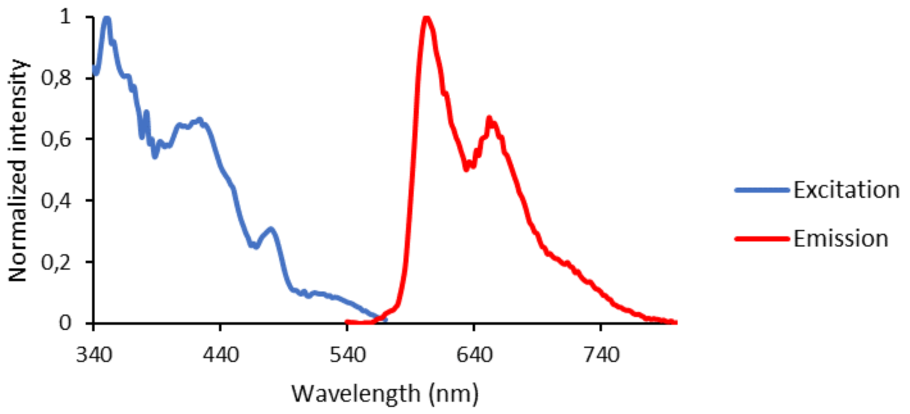

Figure S29. Normalized emission and excitation spectrum for a 1 x 10^{-5} M solution of complex **9a** in 2-methyl tetrahydrofuran at 77 K.

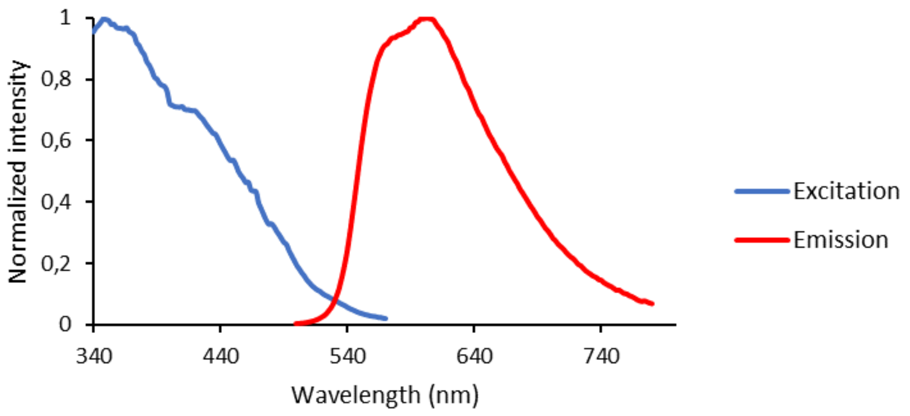

Figure S30. Normalized emission and excitation spectrum of complex **9b** in PMMA film (5 wt %) at 298 K.

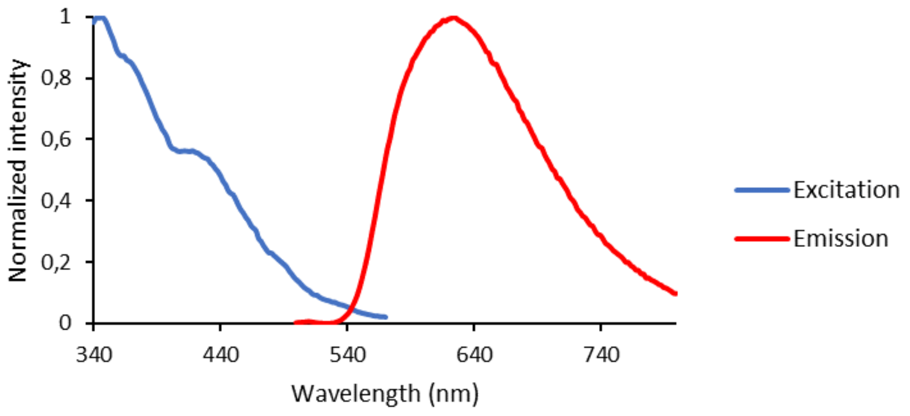

Figure S31. Normalized emission and excitation spectrum for a 1 x 10^{-5} M solution of complex **9b** in 2-methyl tetrahydrofuran at 298 K.

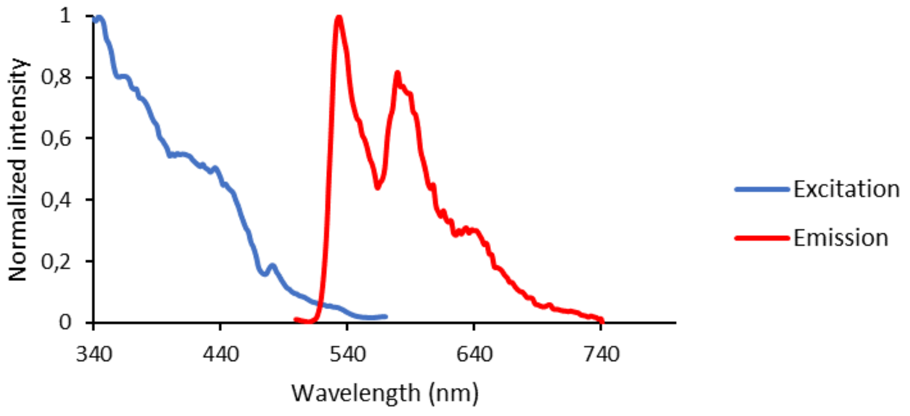

Figure S32. Normalized emission and excitation spectrum for a 1 x 10^{-5} M solution of complex **9b** in 2-methyl tetrahydrofuran at 77 K.

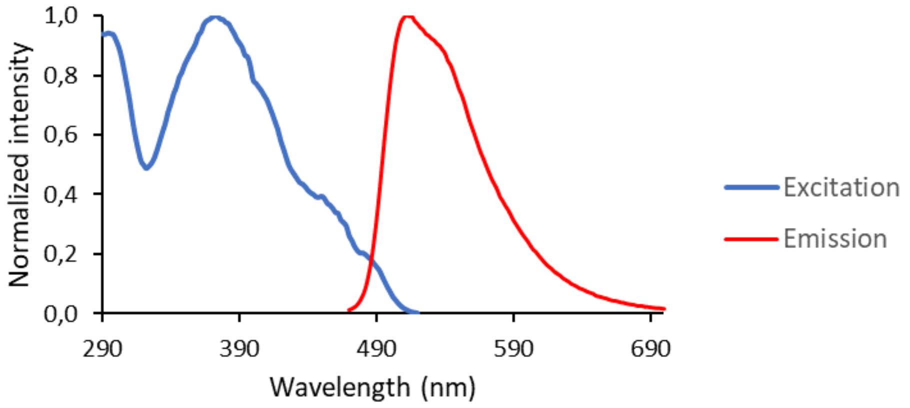

Figure S33. Normalized emission and excitation spectrum of complex **10a** in PMMA film (5 wt %) at 298 K.

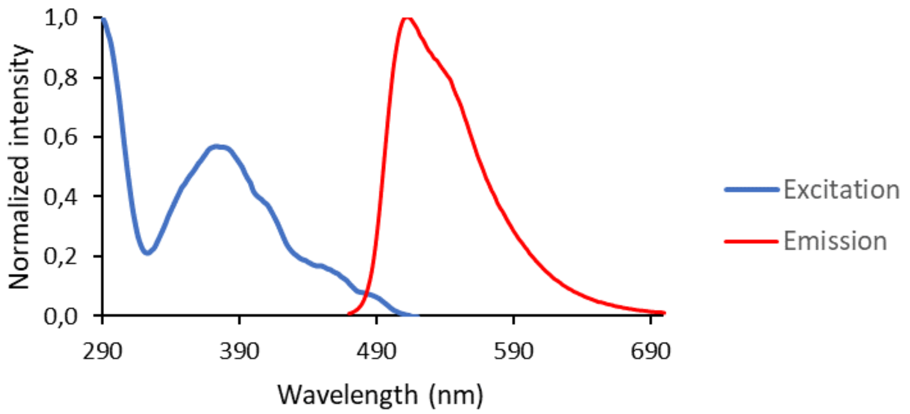

Figure S34. Normalized emission and excitation spectrum for a 1 x 10^{-5} M solution of complex **10a** in 2-methyl tetrahydrofuran at 298 K.

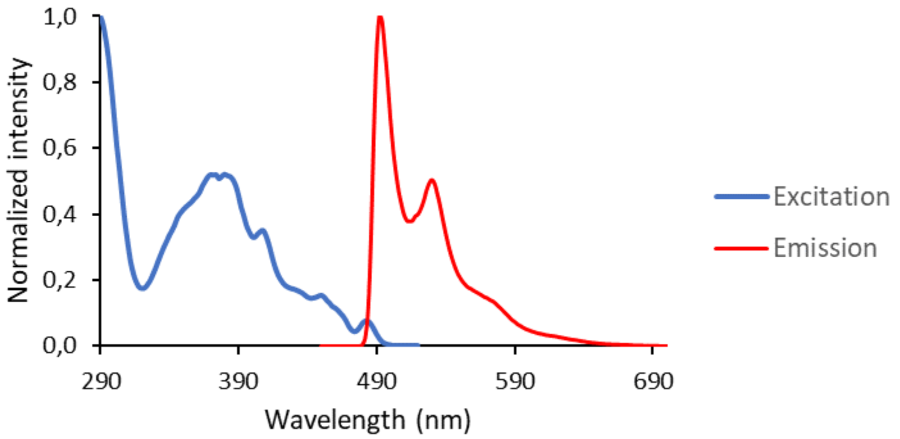

Figure S35. Normalized emission and excitation spectrum for a 1 x 10^{-5} M solution of complex **10a** in 2-methyl tetrahydrofuran at 77 K.

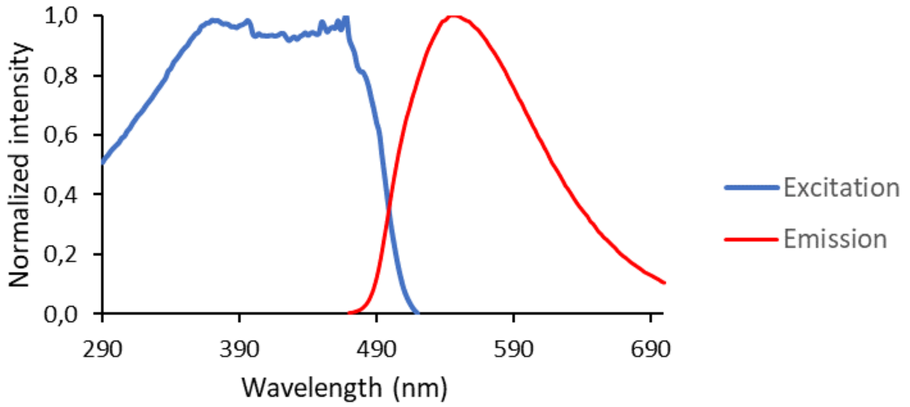

Figure S36. Normalized emission and excitation spectrum of complex **10b** in PMMA film (5 wt %) at 298 K.

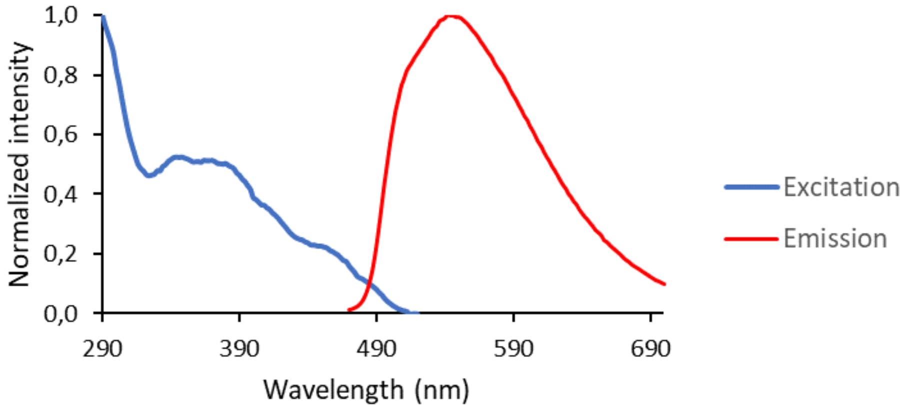

Figure S37. Normalized emission and excitation spectrum for a 1 x 10^{-5} M solution of complex **10b** in 2-methyl tetrahydrofuran at 298 K.

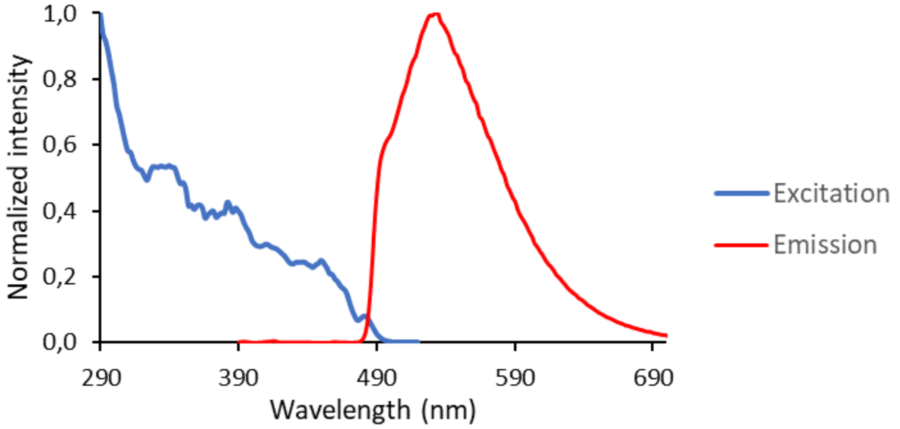

Figure S38. Normalized emission and excitation spectrum for a 1 x 10^{-5} M solution of complex **10b** in 2-methyl tetrahydrofuran at 77 K.

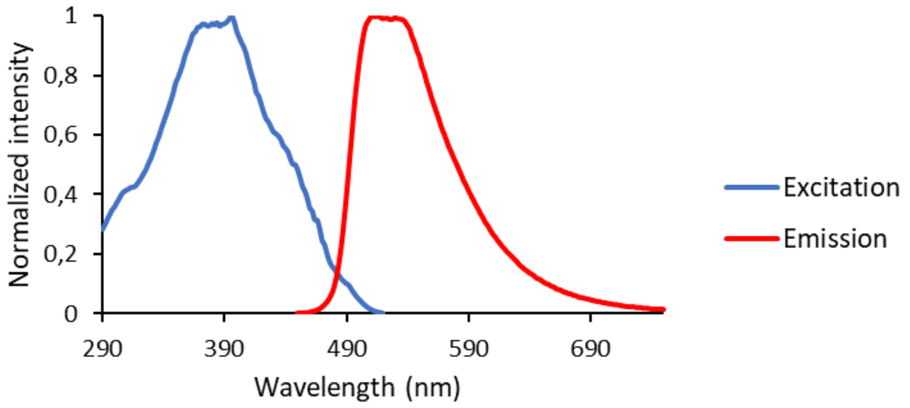

Figure S39. Normalized emission and excitation spectrum of complex **11a** in PMMA film (5 wt %) at 298 K.

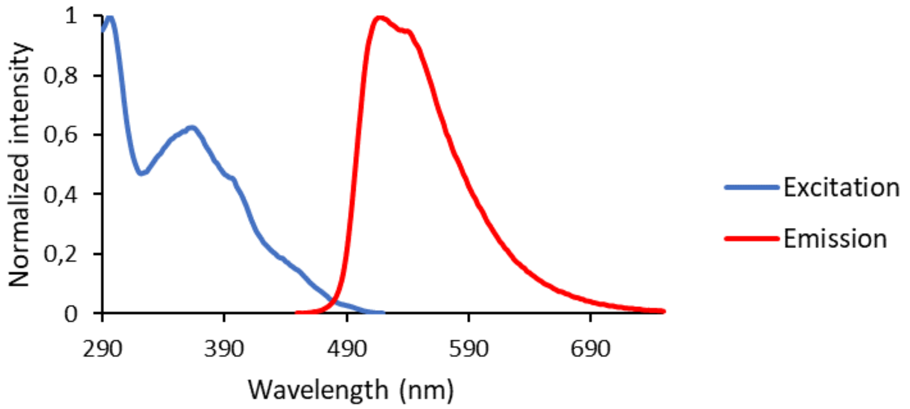

Figure S40. Normalized emission and excitation spectrum for a 1 x 10^{-5} M solution of complex **11a** in 2-methyl tetrahydrofuran at 298 K.

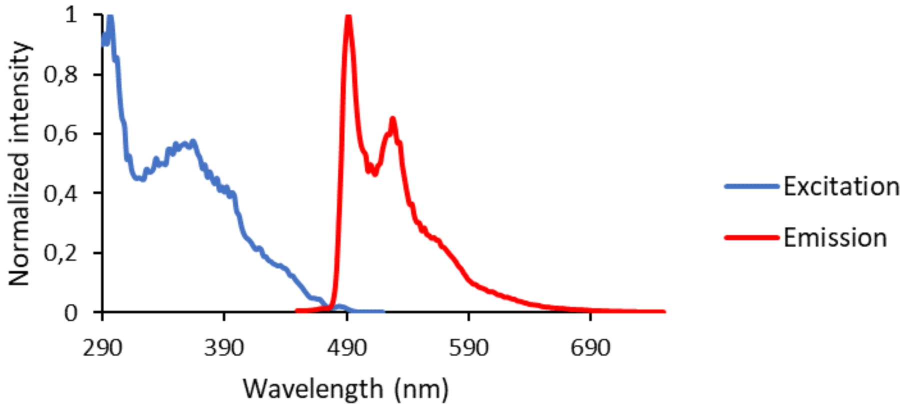

Figure S41. Normalized emission and excitation spectrum for a 1 x 10^{-5} M solution of complex **11a** in 2-methyl tetrahydrofuran at 77 K.

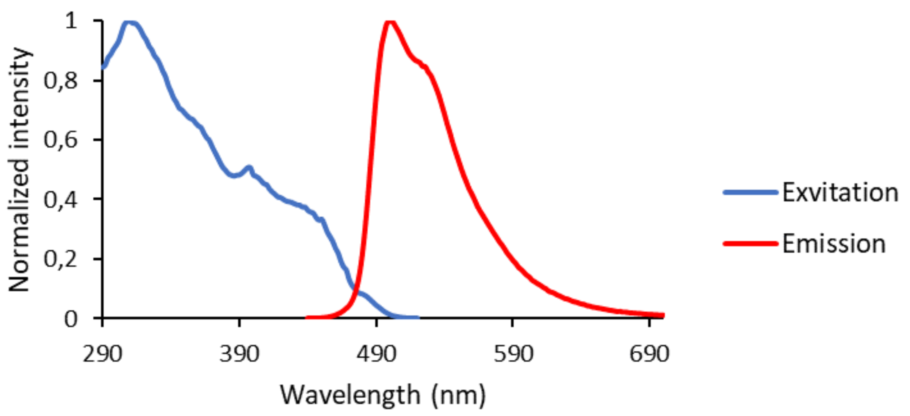

Figure S42. Normalized emission and excitation spectrum of complex **11b** in PMMA film (5 wt %) at 298 K.

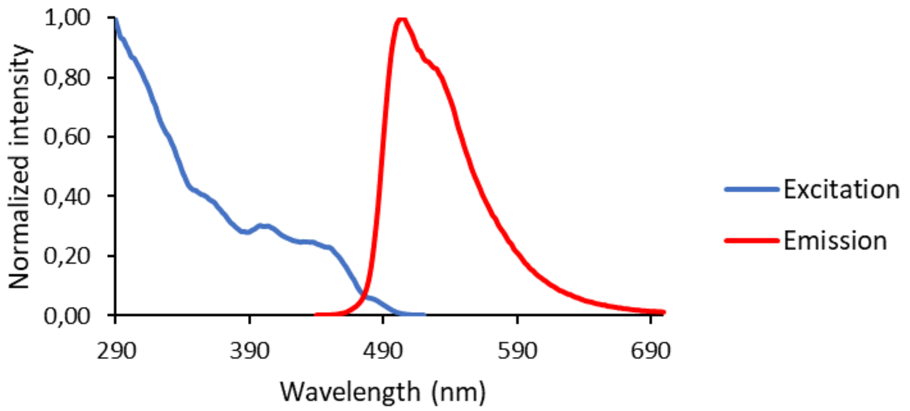

Figure S43. Normalized emission and excitation spectrum for a 1 x 10^{-5} M solution of complex **11b** in 2-methyl tetrahydrofuran at 298 K.

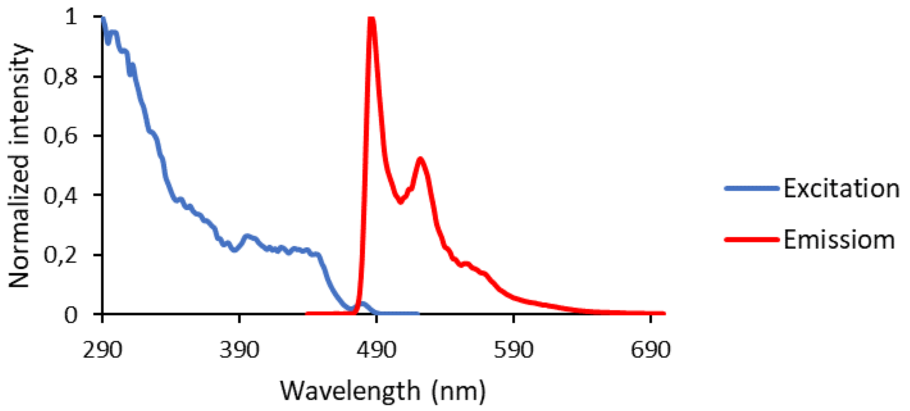

Figure S44. Normalized emission and excitation spectrum for a 1 x 10^{-5} M solution of complex **11b** in 2-methyl tetrahydrofuran at 77 K.

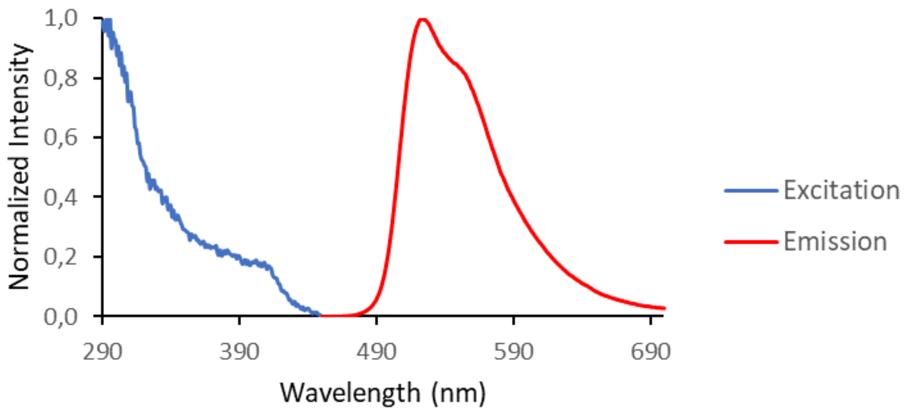

Figure S45. Normalized emission and excitation spectrum of complex **12a** in PMMA film (5 wt %) at 298 K.

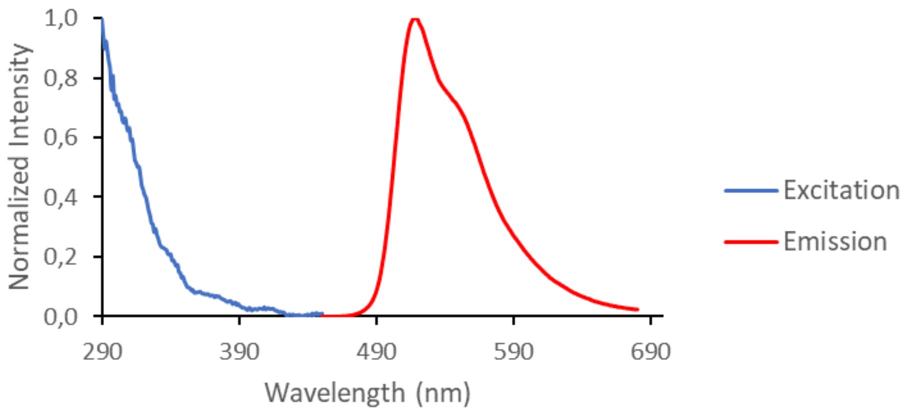

Figure S46. Normalized emission and excitation spectrum for a 1 x 10^{-5} M solution of complex **12a** in 2-methyl tetrahydrofuran at 298 K.

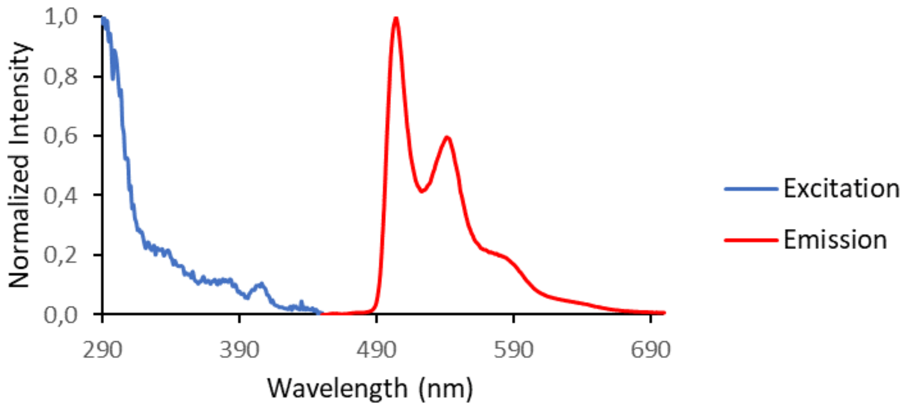

Figure S47. Normalized emission and excitation spectrum for a 1 x 10^{-5} M solution of complex **12a** in 2-methyl tetrahydrofuran at 77 K.

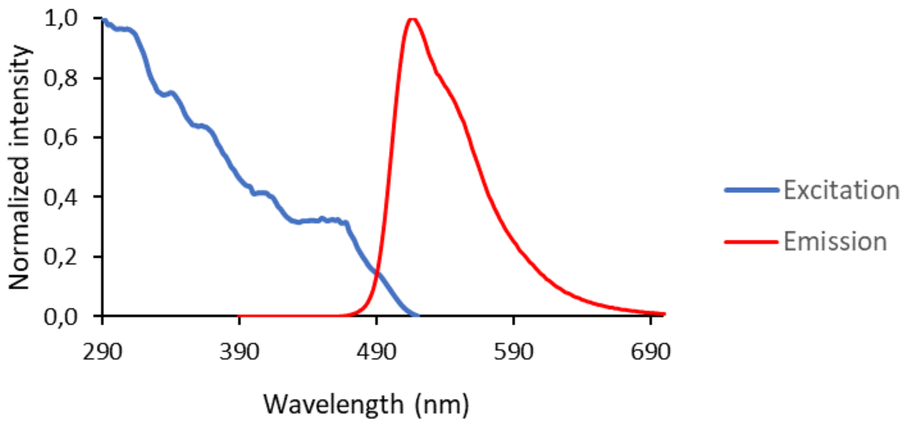

Figure S48. Normalized emission and excitation spectrum of complex **12b** in PMMA film (5 wt %) at 298 K.

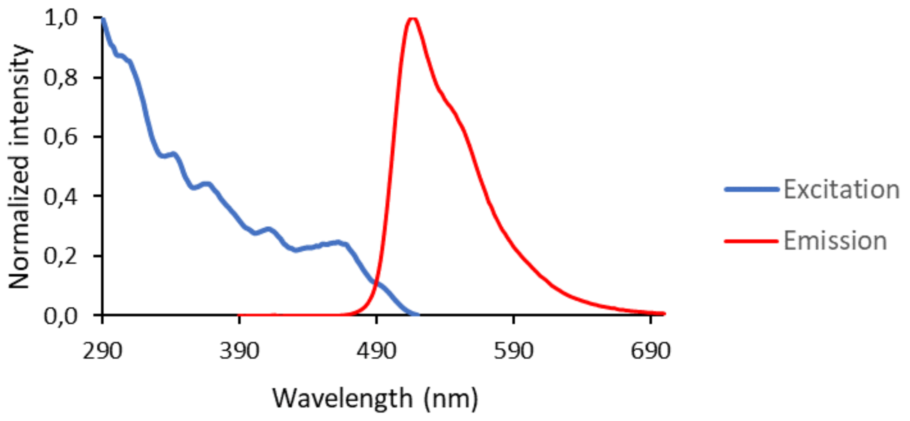

Figure S49. Normalized emission and excitation spectrum for a 1 x 10^{-5} M solution of complex **12b** in 2-methyl tetrahydrofuran at 298 K.

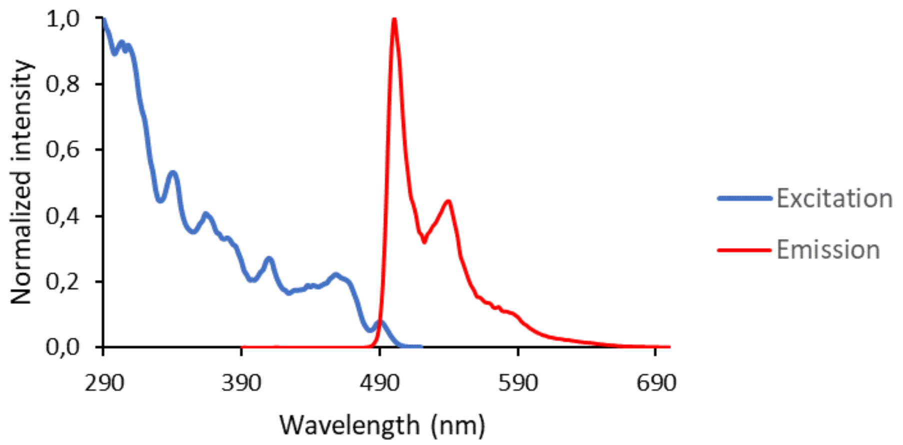

Figure S50. Normalized emission and excitation spectrum for a 1 x 10^{-5} M solution of complex **12b** in 2-methyl tetrahydrofuran at 77 K.

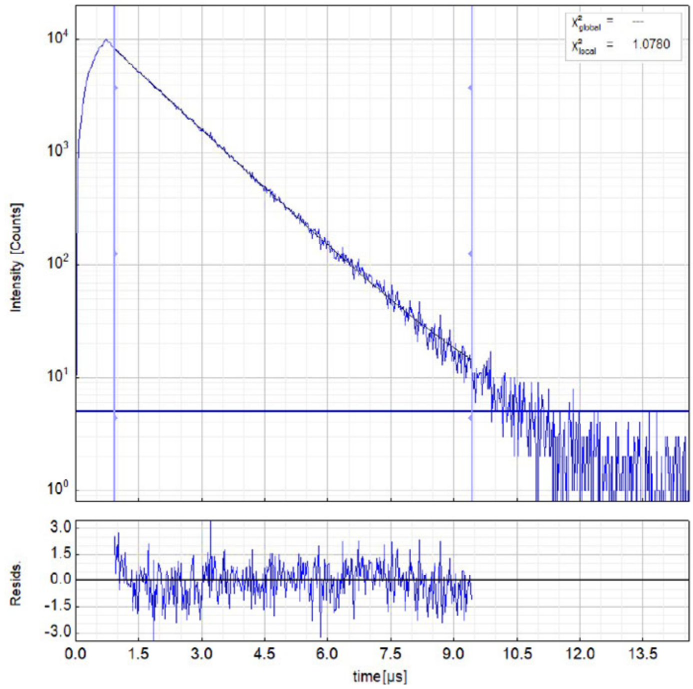

Parameter	Value	Δ	δ
A_1[kCnts/Chnl]	8.279	±0.019	0.2%
τ_1[ns]	1 258.8	±2.7	0.2%
I_1[kCnts]	521.03	±0.71	0.1%
$Bkgr_{Dec}$[kCnts]	0.0050	±0.0008	14%
τ_{AvInt}[ns]	1 258.8	±2.7	0.2%

Figure S51. Left: Raw (experimental) time-resolved photoluminescence decay of **3** in PMMA film (5 wt%) at 298 K (λ_{exc} = 370 nm, λ_{em} = 499 nm). Right: Fitting parameters and confidence limits.

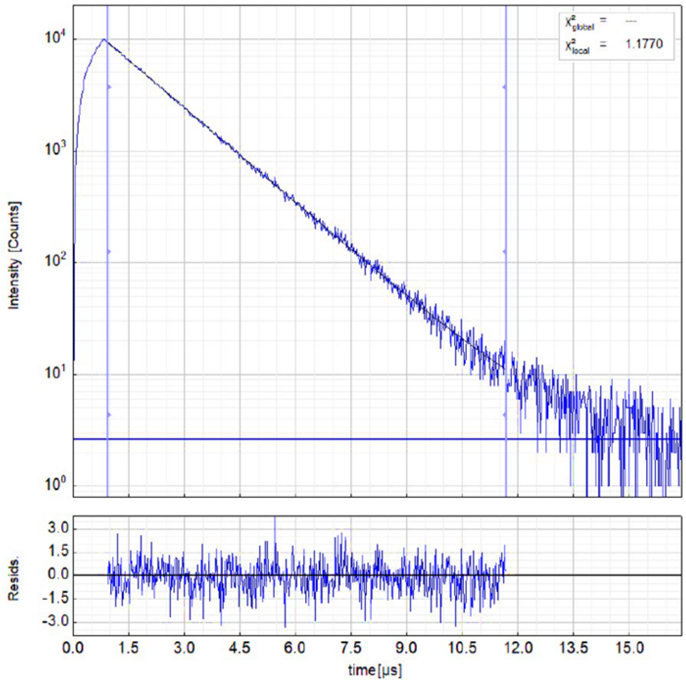

Parameter	Value	Δ	δ
A_1[kCnts/Chnl]	9.323	±0.013	0.1%
τ_1[ns]	1 533.8	±2.4	0.2%
I_1[kCnts]	714.95	±0.58	0.1%
$Bkgr_{Dec}$[kCnts]	0.0027	±0.0003	11%
τ_{AvInt}[ns]	1 533.8	±2.4	0.2%

Figure S52. Left: Raw (experimental) time-resolved photoluminescence decay of **3** (1 x 10^{-5} M) in 2-methyl tetrahydrofuran at 298 K (λ_{exc} = 370 nm, λ_{em} = 499 nm). Right: Fitting parameters and confidence limits.

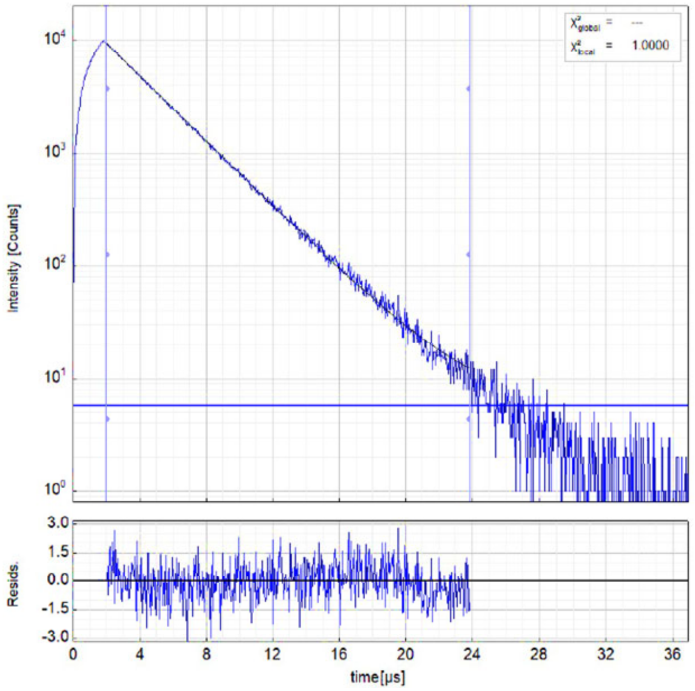

Parameter	Value	Δ	δ
A_1[kCnts/Chnl]	9.263	±0.019	0.2%
τ_1[ns]	3 008.7	±3.0	0.1%
I_1[kCnts]	696.69	±0.60	0.1%
$Bkgr_{Dec}$[kCnts]	0.0057	±0.0005	7.5%
τ_{AvInt}[ns]	3 008.7	±3.0	0.1%

Figure S53. Left: Raw (experimental) time-resolved photoluminescence decay of **3** (1×10^{-5} M) in 2-methyl tetrahydrofuran at 77 K (λ_{exc} = 370 nm, λ_{em} = 489 nm). Right: Fitting parameters and confidence limits.

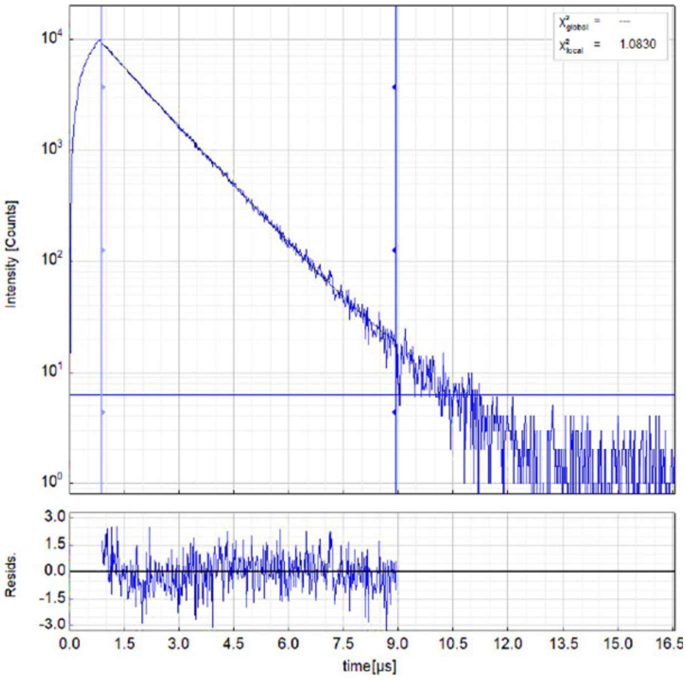

Parameter	Value	Δ	δ
A_1[kCnts/Chnl]	9.063	±0.016	0.2%
τ_1[ns]	1 224.4	±2.6	0.2%
I_1[kCnts]	554.79	±0.61	0.1%
$Bkgr_{Dec}$[kCnts]	0.0062	±0.0006	9.6%
τ_{AvInt}[ns]	1 224.4	±2.6	0.2%

Figure S54. Left: Raw (experimental) time-resolved photoluminescence decay of **9a** in PMMA film (5 wt%) at 298 K (λ_{exc} = 400 nm, λ_{em} = 615 nm). Right: Fitting parameters and confidence limits.

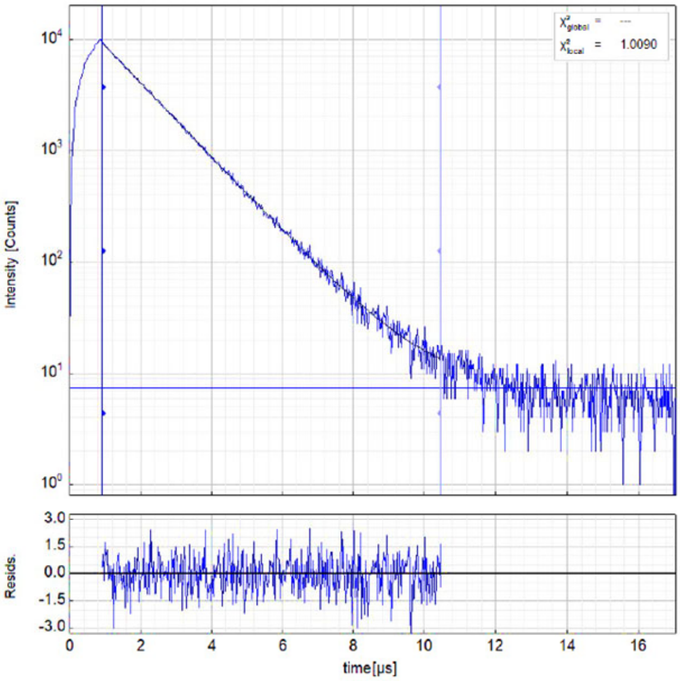

Parameter	Value	Δ	δ
A_1[kCnts/Chnl]	9.320	±0.011	0.1%
τ_1[ns]	1 299.5	±1.9	0.1%
I_1[kCnts]	605.56	±0.51	0.1%
$Bkgr_{Dec}$[kCnts]	0.0074	±0.0005	5.7%
τ_{AvInt}[ns]	1 299.5	±1.9	0.1%

Figure S55. Left: Raw (experimental) time-resolved photoluminescence decay of **9a** (1 x 10^{-5} M) in 2-methyl tetrahydrofuran at 298 K (λ_{exc} = 400 nm, λ_{em} = 625 nm). Right: Fitting parameters and confidence limits.

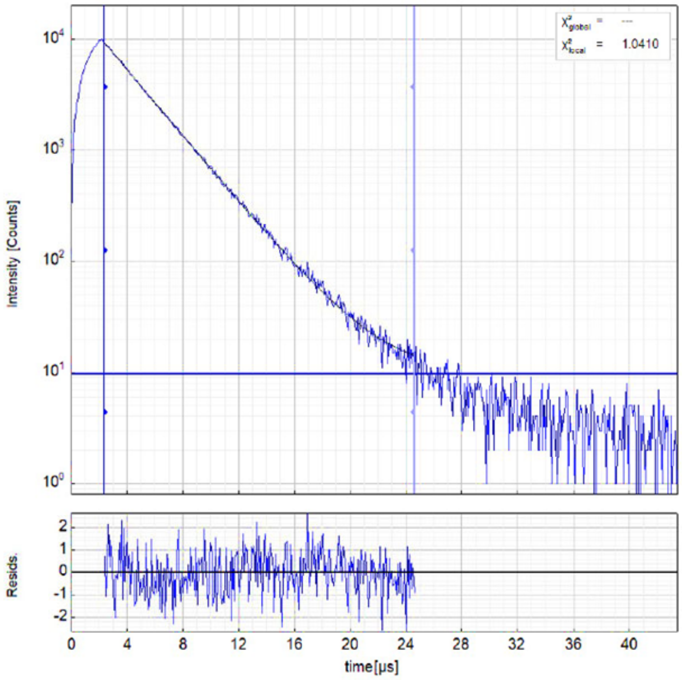

Parameter	Value	Δ	δ
A_1[kCnts/Chnl]	9.171	±0.021	0.2%
τ_1[ns]	2 916.3	±6.7	0.2%
I_1[kCnts]	334.31	±0.26	0.1%
$Bkgr_{Dec}$[kCnts]	0.0097	±0.0007	7.0%
τ_{AvInt}[ns]	2 916.3	±6.7	0.2%

Figure S56. Left: Raw (experimental) time-resolved photoluminescence decay of **9a** (1 x 10^{-5} M) in 2-methyl tetrahydrofuran at 77 K (λ_{exc} = 400 nm, λ_{em} = 605 nm). Right: Fitting parameters and confidence limits.

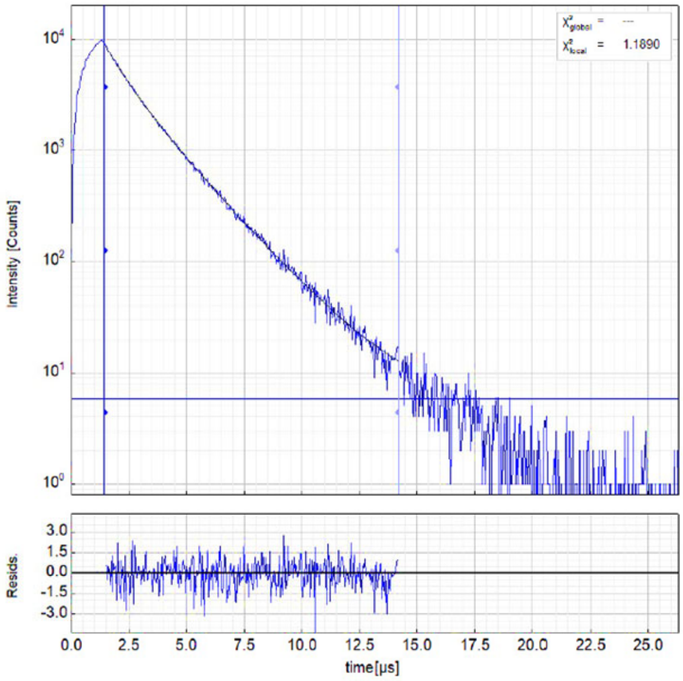

Parameter	Value	Δ	δ
A_1[kCnts/Chnl]	4.71	±0.23	4.8%
$τ_1$[ns]	1 952	±33	1.7%
I_1[kCnts]	229.6	±7.1	3.1%
A_2[kCnts/Chnl]	3.95	±0.24	6.0%
$τ_2$[ns]	874	±28	3.2%
I_2[kCnts]	86.3	±7.8	9.0%
$Bkgr_{Dec}$[kCnts]	0.0059	±0.0014	23%
$τ_{AvInt}$[ns]	1 657.4	±9.9	0.6%

Figure S57. Left: Raw (experimental) time-resolved photoluminescence decay of **9b** in PMMA film (5 wt%) at 298 K ($λ_{exc}$ = 400 nm, $λ_{em}$ = 595 nm). Right: Fitting parameters and confidence limits.

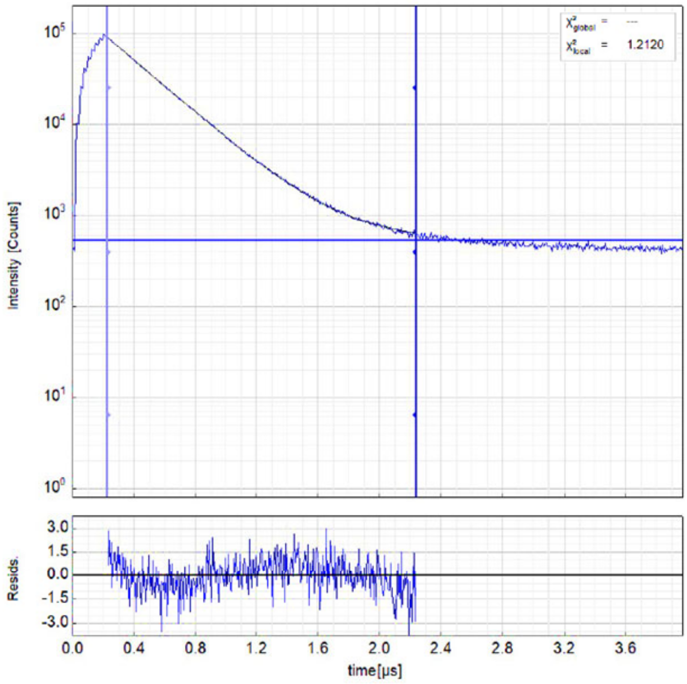

Parameter	Value	Δ	δ
A_1[kCnts/Chnl]	88.91	±0.11	0.1%
$τ_1$[ns]	298.42	±0.31	0.1%
I_1[kCnts]	5 306.3	±3.5	0.1%
$Bkgr_{Dec}$[kCnts]	0.5316	±0.0032	0.6%
$τ_{AvInt}$[ns]	298.42	±0.31	0.1%

Figure S58. Left: Raw (experimental) time-resolved photoluminescence decay of **9b** (1 x 10^{-5} M) in 2-methyl tetrahydrofuran at 298 K ($λ_{exc}$ = 400 nm, $λ_{em}$ = 644 nm). Right: Fitting parameters and confidence limits.

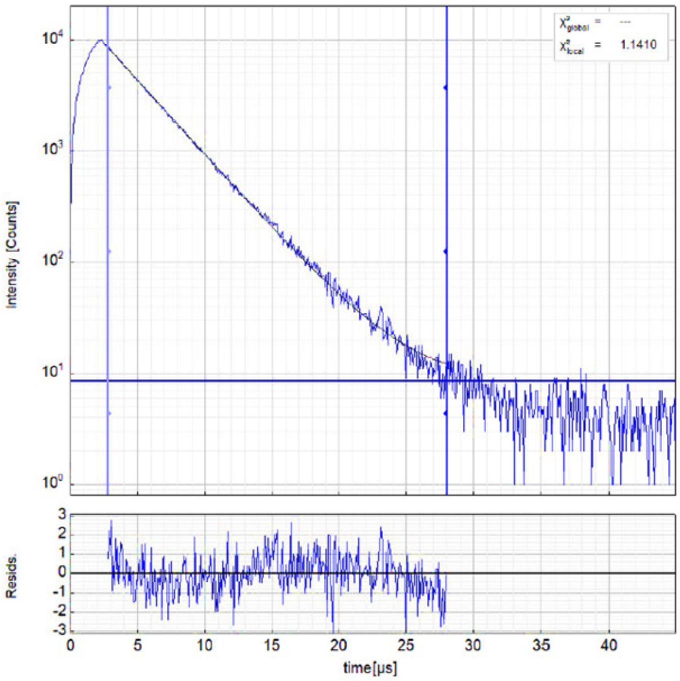

Parameter	Value	Δ	δ
A_1[kCnts/Chnl]	8.428	±0.023	0.3%
τ_1[ns]	3 257.8	±8.2	0.2%
I_1[kCnts]	343.20	±0.78	0.2%
$Bkgr_{Dec}$[kCnts]	0.0085	±0.0008	9.0%
τ_{AvInt}[ns]	3 257.8	±8.2	0.2%

Figure S59. Left: Raw (experimental) time-resolved photoluminescence decay of **9b** (1 x 10^{-5} M) in 2-methyl tetrahydrofuran at 77 K (λ_{exc} = 400 nm, λ_{em} = 575 nm). Right: Fitting parameters and confidence limits.

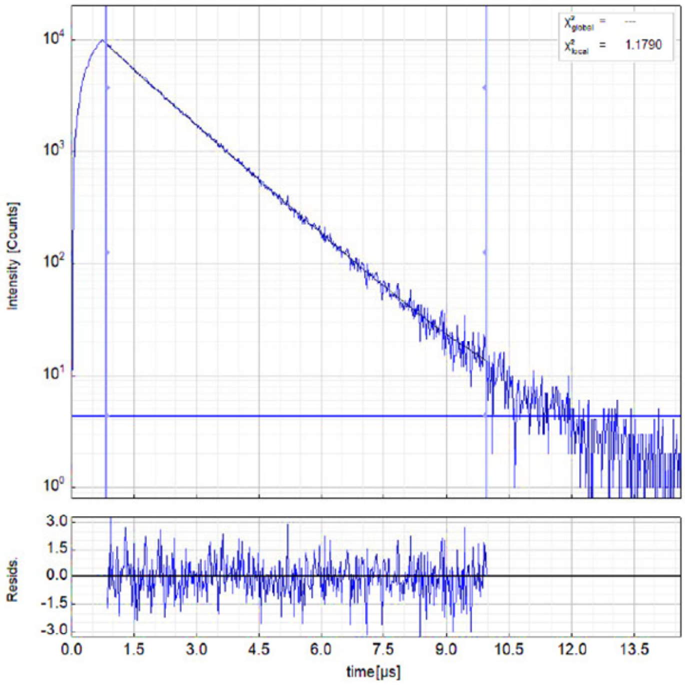

Parameter	Value	Δ	δ
A_1[kCnts/Chnl]	8.64	±0.12	1.4%
τ_1[ns]	1 334.3	±7.5	0.6%
I_1[kCnts]	576.1	±4.8	0.8%
A_2[kCnts/Chnl]	0.512	±0.076	15%
τ_2[ns]	440	±140	30%
I_2[kCnts]	11.3	±4.6	41%
$Bkgr_{Dec}$[kCnts]	0.0044	±0.0009	19%
τ_{AvInt}[ns]	1 317.2	±3.5	0.3%

Figure S60. Left: Raw (experimental) time-resolved photoluminescence decay of **10a** in PMMA film (5 wt%) at 298 K (λ_{exc} = 370 nm, λ_{em} = 514 nm). Right: Fitting parameters and confidence limits.

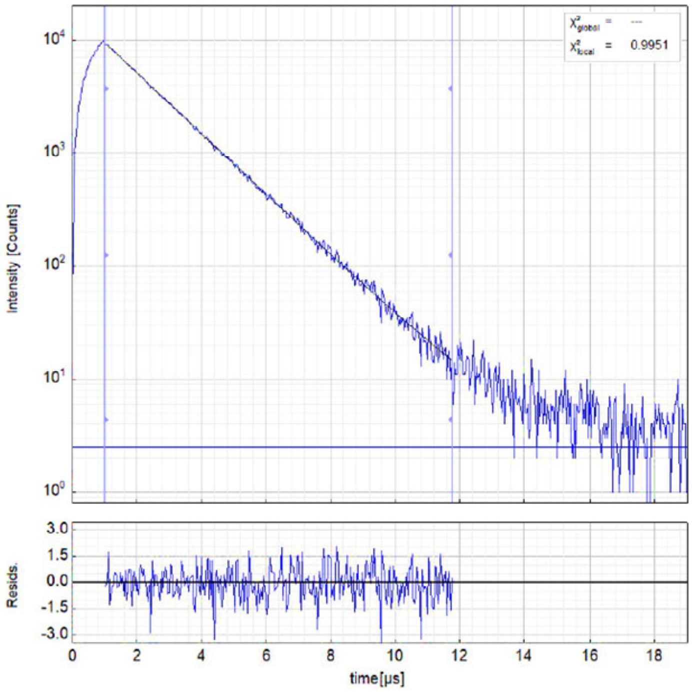

Parameter	Value	Δ	δ
A_1[kCnts/Chnl]	9.246	±0.013	0.1%
τ_1[ns]	1 617.2	±1.8	0.1%
I_1[kCnts]	373.81	±0.61	0.2%
$Bkgr_{Dec}$[kCnts]	0.0025	±0.0009	33%
τ_{AvInt}[ns]	1 617.2	±1.8	0.1%

Figure S61. Left: Raw (experimental) time-resolved photoluminescence decay of **10a** (1 x 10^{-5} M) in 2-methyl tetrahydrofuran at 298 K (λ_{exc} = 370 nm, λ_{em} = 514 nm). Right: Fitting parameters and confidence limits.

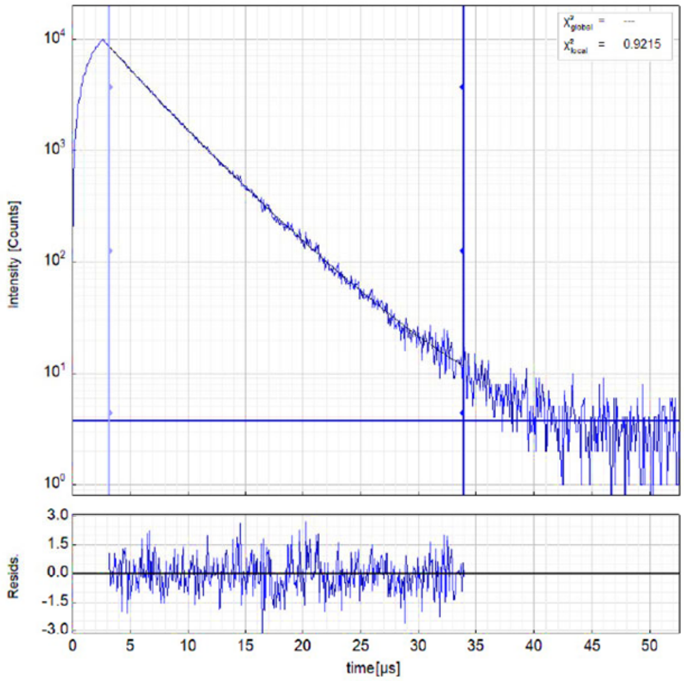

Parameter	Value	Δ	δ
A_1[kCnts/Chnl]	3.3	±1.7	52%
τ_1[ns]	5 060	±570	11%
I_1[kCnts]	203	±90	44%
A_2[kCnts/Chnl]	5.4	±1.7	31%
τ_2[ns]	3 340	±280	8.2%
I_2[kCnts]	222	±92	41%
$Bkgr_{Dec}$[kCnts]	0.0037	±0.0019	49%
τ_{AvInt}[ns]	4 157	±35	0.8%

Figure S62. Left: Raw (experimental) time-resolved photoluminescence decay of **10a** (1 x 10^{-5} M) in 2-methyl tetrahydrofuran at 77 K (λ_{exc} = 370 nm, λ_{em} = 494 nm). Right: Fitting parameters and confidence limits.

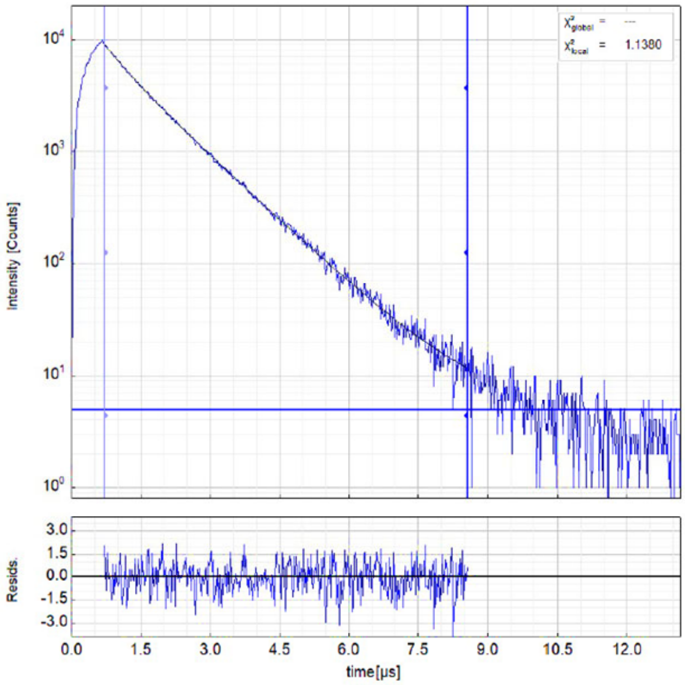

Parameter	Value	Δ	δ
A_1[kCnts/Chnl]	6.74	±0.21	3.1%
τ_1[ns]	1 136	±12	1.0%
I_1[kCnts]	382.5	±8.1	2.1%
A_2[kCnts/Chnl]	2.19	±0.20	8.7%
τ_2[ns]	487	±29	5.9%
I_2[kCnts]	53.1	±8.3	15%
$Bkgr_{Dec}$[kCnts]	0.0050	±0.0008	15%
τ_{AvInt}[ns]	1 056.5	±2.0	0.2%

Figure S63. Left: Raw (experimental) time-resolved photoluminescence decay of **10b** in PMMA film (5 wt%) at 298 K (λ_{exc} = 370 nm, λ_{em} = 544 nm). Right: Fitting parameters and confidence limits.

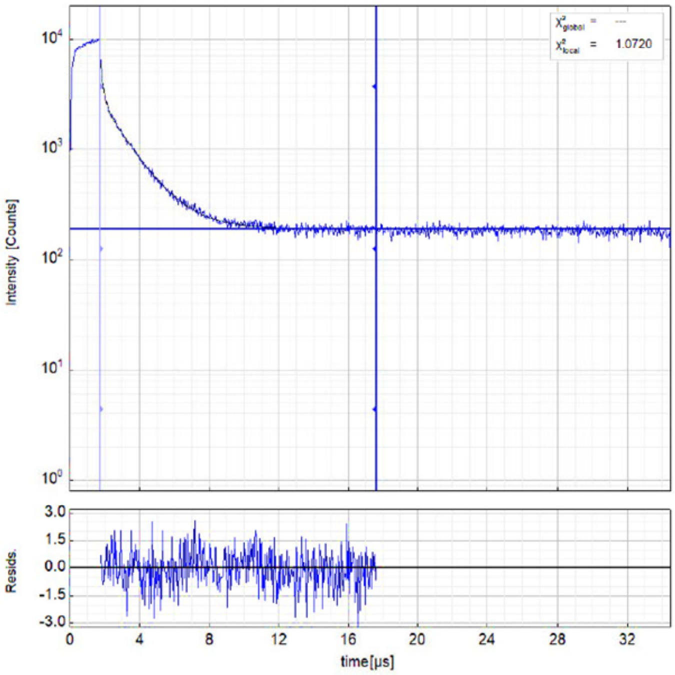

Parameter	Value	Δ	δ
A_1[kCnts/Chnl]	2.560	±0.029	1.1%
τ_1[ns]	1 605	±16	1.0%
I_1[kCnts]	102.69	±0.25	0.2%
A_2[kCnts/Chnl]	3.158	±0.061	1.9%
τ_2[ns]	128.3	±4.1	3.1%
I_2[kCnts]	10.13	±0.30	2.9%
$Bkgr_{Dec}$[kCnts]	0.1891	±0.0013	0.7%
τ_{AvInt}[ns]	1 472	±11	0.7%

Figure S64. Left: Raw (experimental) time-resolved photoluminescence decay of **10b** (1 x 10^{-5} M) in 2-methyl tetrahydrofuran at 298 K (λ_{exc} = 370 nm, λ_{em} = 546 nm). Right: Fitting parameters and confidence limits.

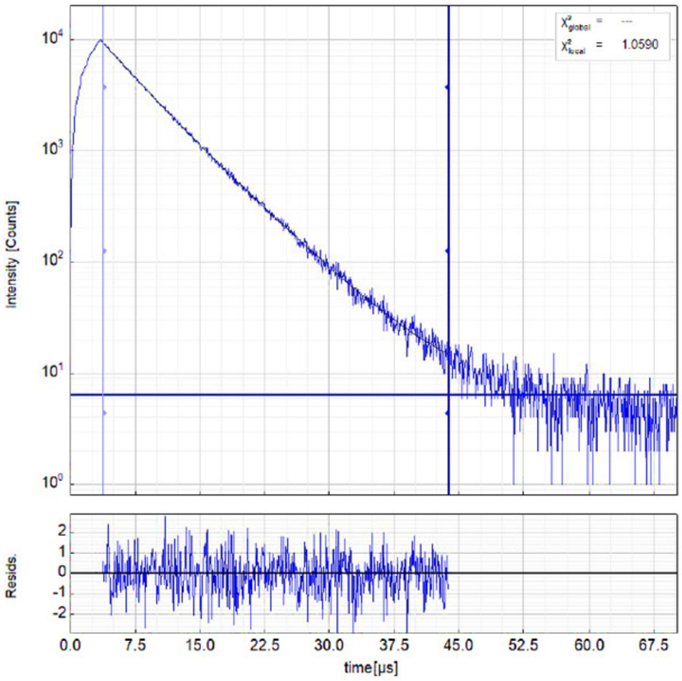

Parameter	Value	Δ	δ
A_1[kCnts/Chnl]	6.0	±2.3	38%
$τ_1$[ns]	6 080	±570	9.2%
I_1[kCnts]	460	±150	32%
A_2[kCnts/Chnl]	3.3	±2.3	67%
$τ_2$[ns]	3 790	±600	16%
I_2[kCnts]	160	±150	94%
$Bkgr_{Dec}$[kCnts]	0.0065	±0.0017	25%
$τ_{AvInt}$[ns]	5 489	±32	0.6%

Figure S65. Left: Raw (experimental) time-resolved photoluminescence decay of **10b** (1 x 10^{-5} M) in 2-methyl tetrahydrofuran at 77 K ($λ_{exc}$ = 370 nm, $λ_{em}$ = 560 nm). Right: Fitting parameters and confidence limits.

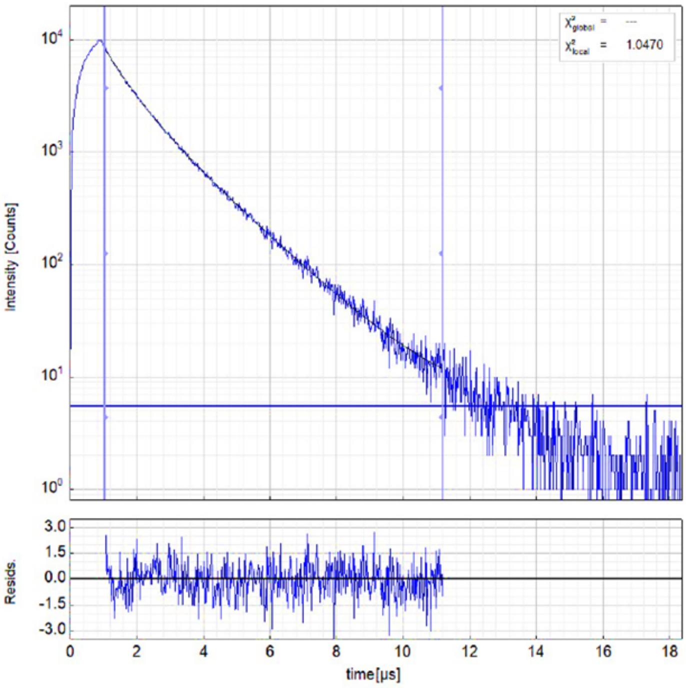

Parameter	Value	Δ	δ
A_1[kCnts/Chnl]	4.08	±0.31	7.4%
$τ_1$[ns]	1 570	±42	2.6%
I_1[kCnts]	320	±16	4.7%
A_2[kCnts/Chnl]	4.13	±0.21	5.1%
$τ_2$[ns]	631	±40	6.2%
I_2[kCnts]	131	±16	12%
$Bkgr_{Dec}$[kCnts]	0.0055	±0.0013	23%
$τ_{AvInt}$[ns]	1 297.8	±8.6	0.7%

Figure S66. Left: Raw (experimental) time-resolved photoluminescence decay of **11a** in PMMA film (5 wt%) at 298 K ($λ_{exc}$ = 400 nm, $λ_{em}$ = 520 nm). Right: Fitting parameters and confidence limits.

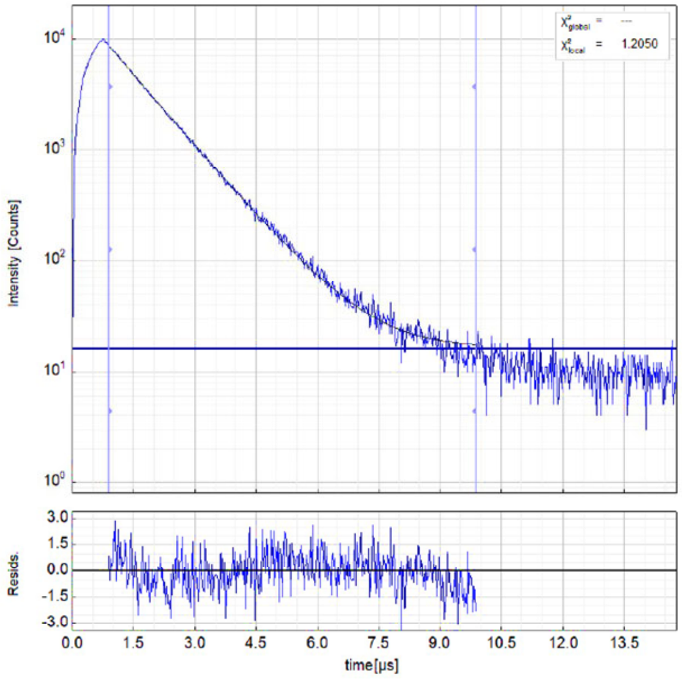

Parameter	Value	Δ	δ
A_1[kCnts/Chnl]	8.550	±0.020	0.2%
τ_1[ns]	1 020.2	±2.5	0.2%
I_1[kCnts]	436.1	±1.2	0.3%
$Bkgr_{Dec}$[kCnts]	0.0161	±0.0005	2.5%
τ_{AvInt}[ns]	1 020.2	±2.5	0.2%

Figure S67. Left: Raw (experimental) time-resolved photoluminescence decay of **11a** (1 x 10^{-5} M) in 2-methyl tetrahydrofuran at 298 K (λ_{exc} = 370 nm, λ_{em} = 519 nm). Right: Fitting parameters and confidence limits.

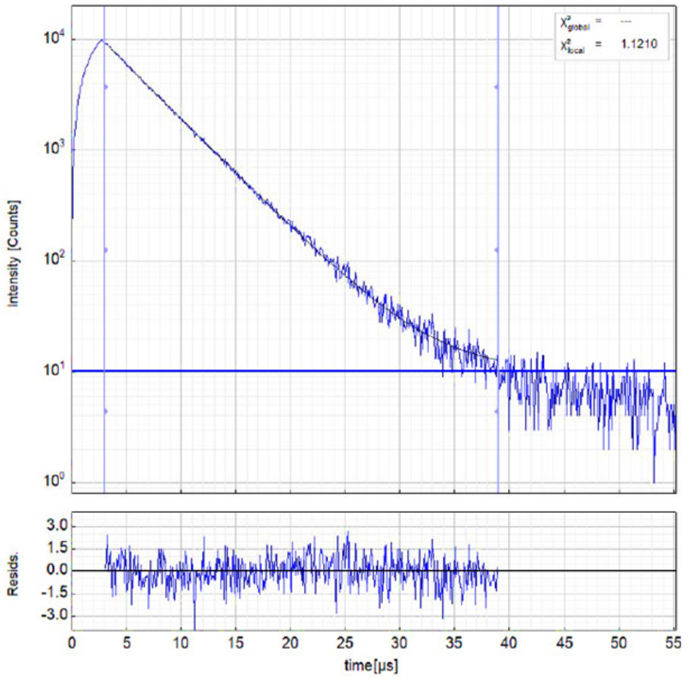

Parameter	Value	Δ	δ
A_1[kCnts/Chnl]	9.230	±0.024	0.3%
τ_1[ns]	4 405.8	±6.0	0.1%
I_1[kCnts]	508.26	±0.76	0.1%
$Bkgr_{Dec}$[kCnts]	0.0102	±0.0003	2.4%
τ_{AvInt}[ns]	4 405.8	±6.0	0.1%

Figure S68. Left: Raw (experimental) time-resolved photoluminescence decay of **11a** (1 x 10^{-5} M) in 2-methyl tetrahydrofuran at 77 K (λ_{exc} = 370 nm, λ_{em} = 494 nm). Right: Fitting parameters and confidence limits.

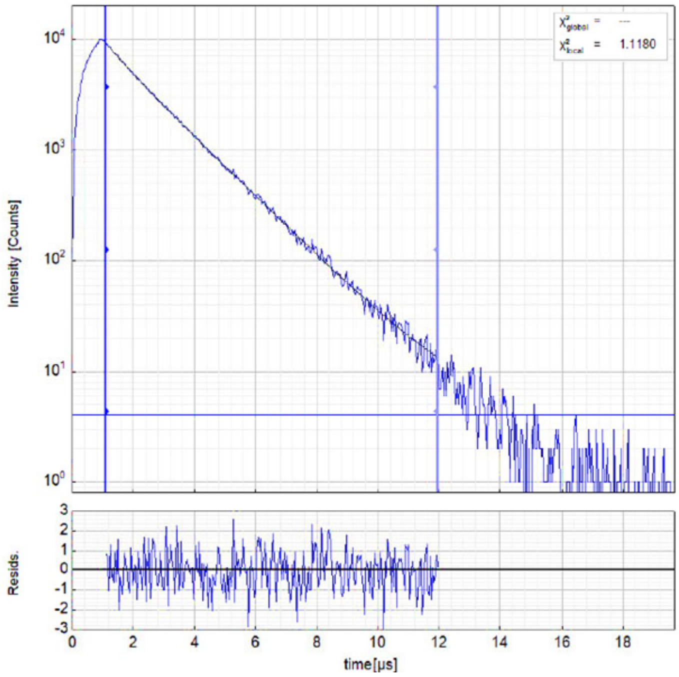

Parameter	Value	Δ	δ
A_1[kCnts/Chnl]	7.89	±0.47	5.9%
τ_1[ns]	1 611	±33	2.0%
I_1[kCnts]	318	±12	3.8%
A_2[kCnts/Chnl]	1.22	±0.41	34%
τ_2[ns]	650	±260	40%
I_2[kCnts]	20	±12	60%
$Bkgr_{Dec}$[kCnts]	0.0041	±0.0013	30%
τ_{AvInt}[ns]	1 554.4	±5.5	0.3%

Figure S69. Left: Raw (experimental) time-resolved photoluminescence decay of **11b** in PMMA film (5 wt%) at 298 K (λ_{exc} = 400 nm, λ_{em} = 500 nm). Right: Fitting parameters and confidence limits.

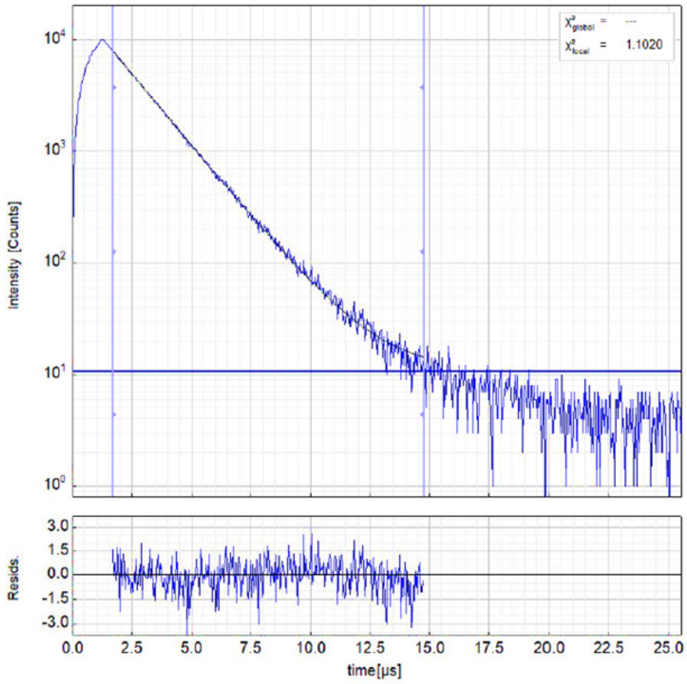

Parameter	Value	Δ	δ
A_1[kCnts/Chnl]	7.870	±0.030	0.4%
τ_1[ns]	1 698.1	±4.8	0.3%
I_1[kCnts]	334.09	±0.64	0.2%
$Bkgr_{Dec}$[kCnts]	0.0107	±0.0004	3.1%
τ_{AvInt}[ns]	1 698.1	±4.8	0.3%

Figure S70. Left: Raw (experimental) time-resolved photoluminescence decay of **11b** (1 x 10^{-5} M) in 2-methyl tetrahydrofuran at 298 K (λ_{exc} = 400 nm, λ_{em} = 504 nm). Right: Fitting parameters and confidence limits.

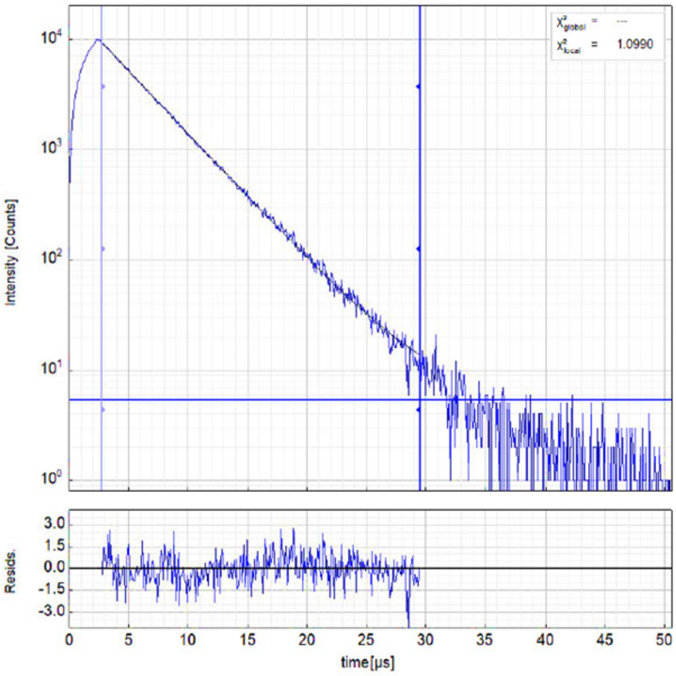

Parameter	Value	Δ	δ
A_1[kCnts/Chnl]	9.173	±0.020	0.2%
τ_1[ns]	3 810.2	±8.5	0.2%
I_1[kCnts]	436.86	±0.54	0.1%
$Bkgr_{Dec}$[kCnts]	0.0054	±0.0006	11%
τ_{AvInt}[ns]	3 810.2	±8.5	0.2%

Figure S71. Left: Raw (experimental) time-resolved photoluminescence decay of **11b** (1 x 10^{-5} M) in 2-methyl tetrahydrofuran at 77 K (λ_{exc} = 400 nm, λ_{em} = 486 nm). Right: Fitting parameters and confidence limits.

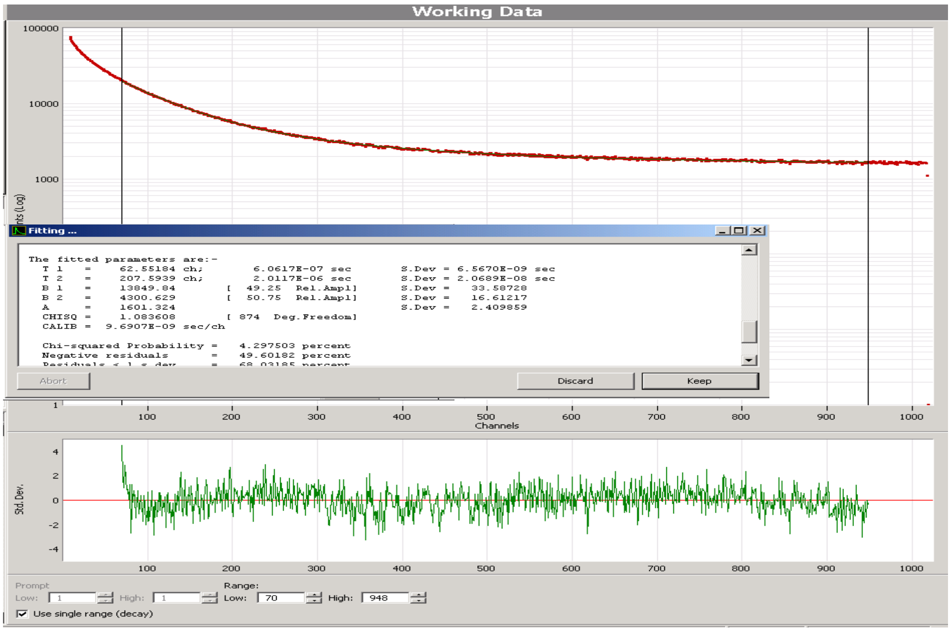

Figure S72. Raw (experimental) time-resolved photoluminescence decay of **12a** in PMMA film (5 wt%) at 298 K, fitting parameters, and confidence limits.

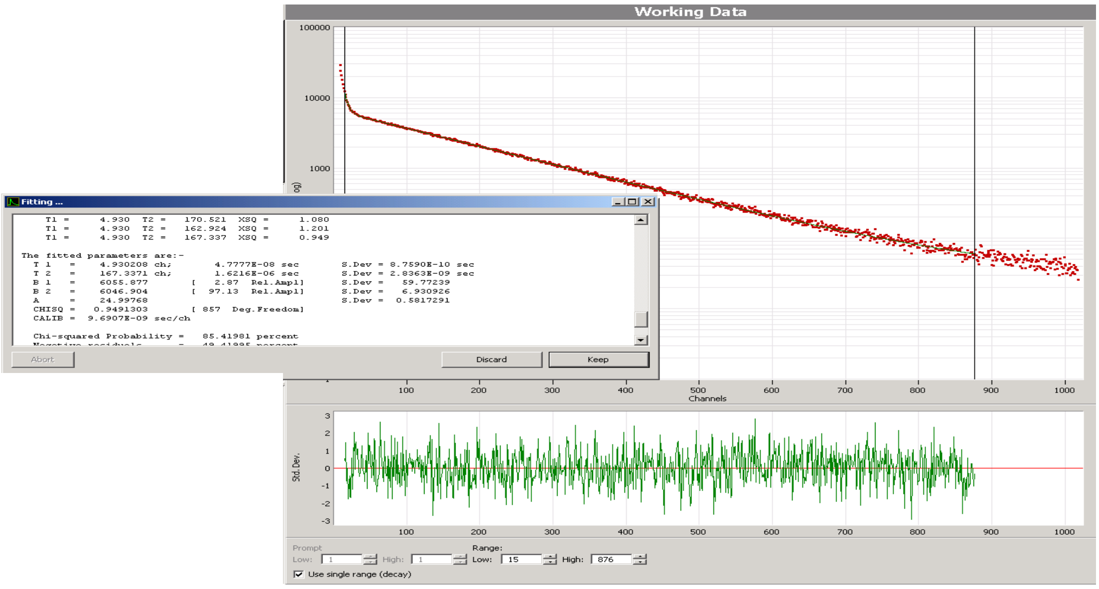

Figure S73. Raw (experimental) time-resolved photoluminescence decay of **12a** (1 x 10^{-5} M) in 2-methyl tetrahydrofuran at 298 K, fitting parameters, and confidence limits.

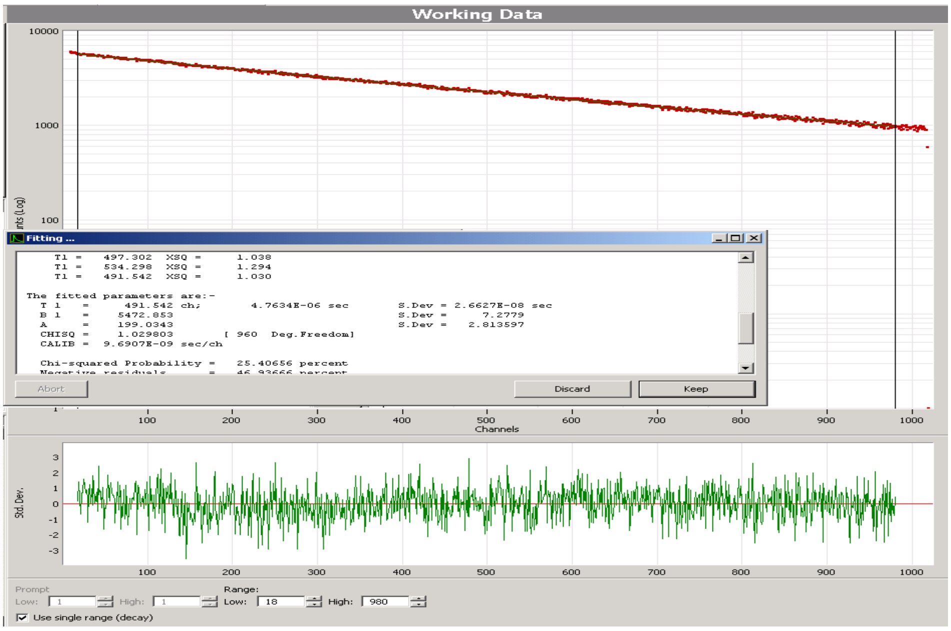

Figure S74. Raw (experimental) time-resolved photoluminescence decay of **12a** (1 x 10^{-5} M) in 2-methyl tetrahydrofuran at 77 K, fitting parameters, and confidence limits.

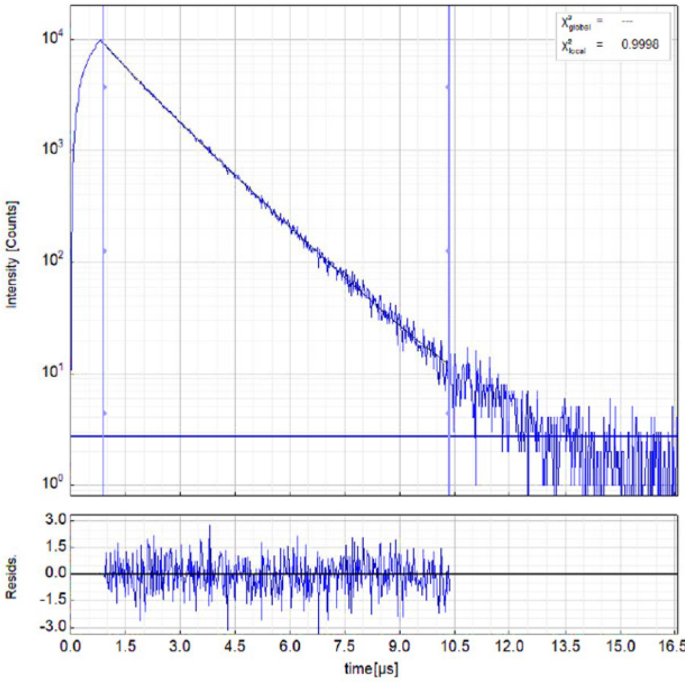

Parameter	Value	Δ	δ
A_1[kCnts/Chnl]	7.34	±0.40	5.4%
τ_1[ns]	1 414	±22	1.5%
I_1[kCnts]	519	±21	4.0%
A_2[kCnts/Chnl]	1.75	±0.40	23%
τ_2[ns]	715	±68	9.4%
I_2[kCnts]	63	±21	33%
$Bkgr_{Dec}$[kCnts]	0.0027	±0.0010	35%
τ_{AvInt}[ns]	1 338.8	±3.2	0.2%

Figure S75. Left: Raw (experimental) time-resolved photoluminescence decay of **12b** in PMMA film (5 wt%) at 298 K (λ_{exc} = 370 nm, λ_{em} = 514 nm). Right: Fitting parameters and confidence limits.

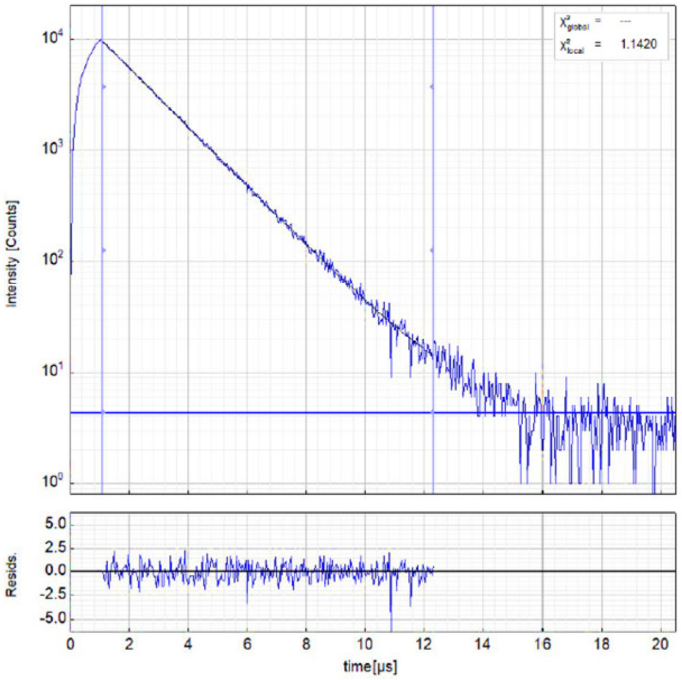

Parameter	Value	Δ	δ
A_1[kCnts/Chnl]	9.392	±0.020	0.2%
τ_1[ns]	1 630.4	±3.3	0.2%
I_1[kCnts]	382.77	±0.46	0.1%
$Bkgr_{Dec}$[kCnts]	0.0043	±0.0005	9.6%
τ_{AvInt}[ns]	1 630.4	±3.3	0.2%

Figure S76. Left: Raw (experimental) time-resolved photoluminescence decay of **12b** (1 x 10^{-5} M) in 2-methyl tetrahydrofuran at 298 K (λ_{exc} = 370 nm, λ_{em} = 519 nm). Right: Fitting parameters and confidence limits.

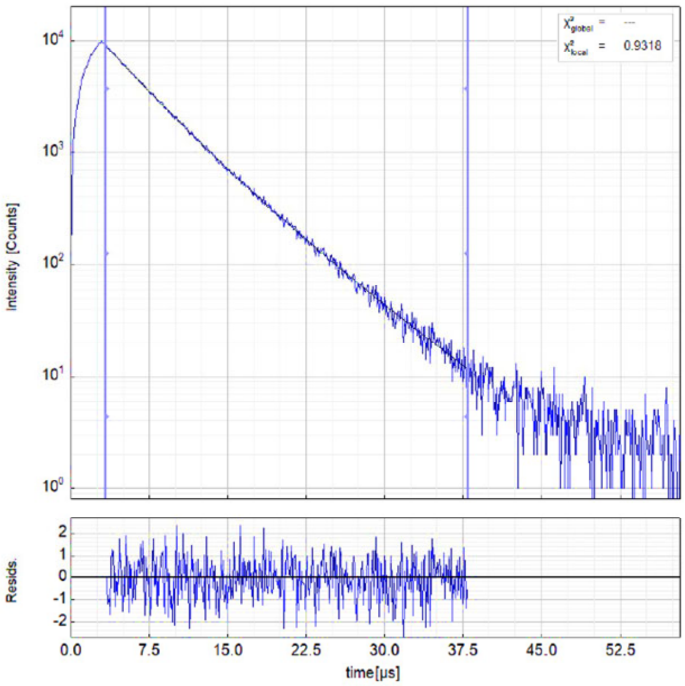

Parameter	Value	Δ	δ
A_1[kCnts/Chnl]	2.2	±1.1	47%
τ_1[ns]	6 520	±620	9.5%
I_1[kCnts]	174	±64	37%
A_2[kCnts/Chnl]	6.81	±0.97	14%
τ_2[ns]	3 960	±210	5.1%
I_2[kCnts]	337	±63	19%
$Bkgr_{Dec}$[kCnts]	0.0001	±0.0015	1859%
τ_{AvInt}[ns]	4 826	±29	0.6%

Figure S77. Left: Raw (experimental) time-resolved photoluminescence decay of **12b** (1 x 10^{-5} M) in 2-methyl tetrahydrofuran at 77 K (λ_{exc} = 370 nm, λ_{em} = 504 nm). Right: Fitting parameters and confidence limits.

• **NMR spectra**

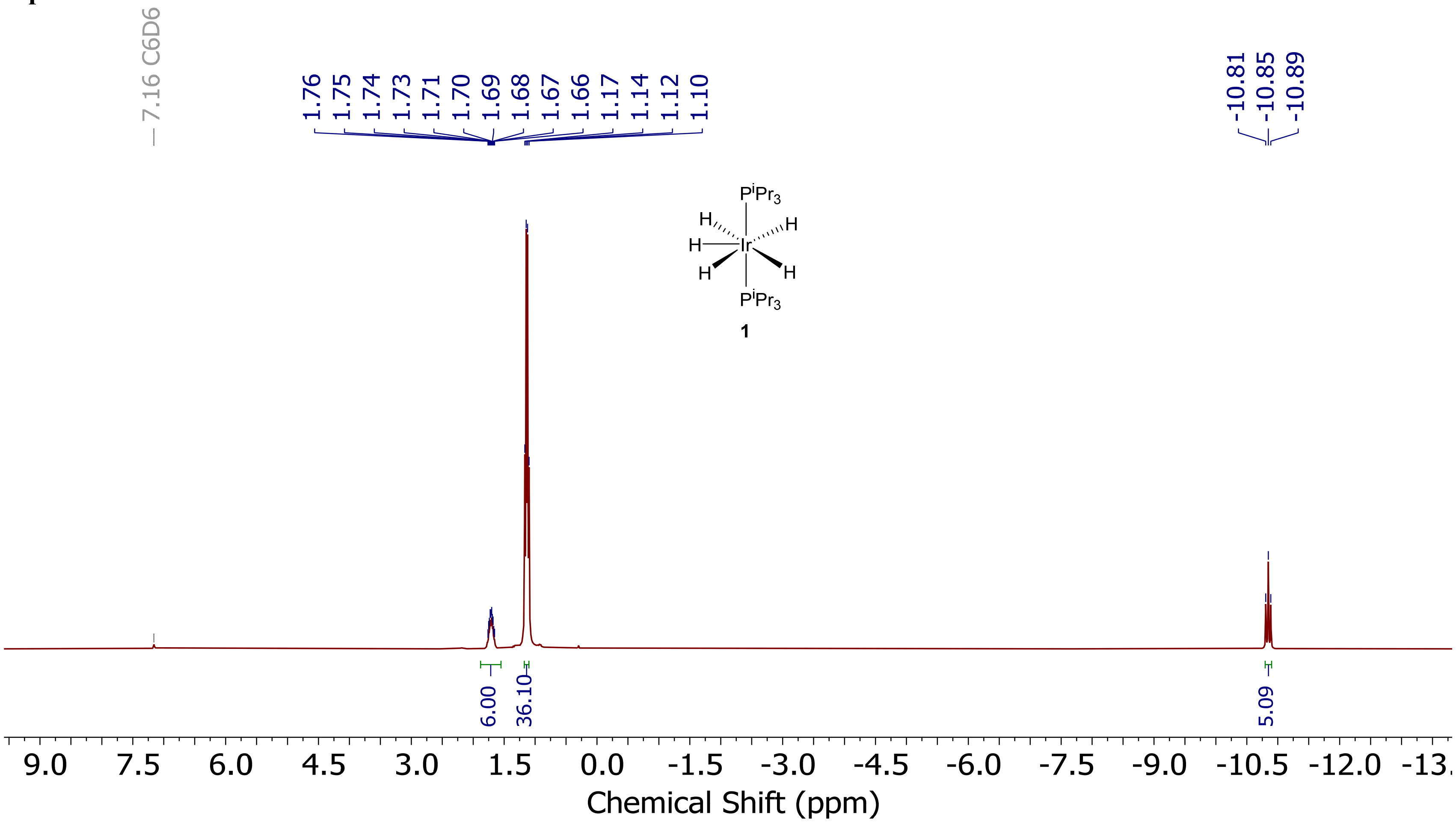

Figure S78. ^{1}H NMR spectrum (300 MHz, benzene-d_6, 298 K) of $IrH_5(P^iPr_3)_2$ (**1**).

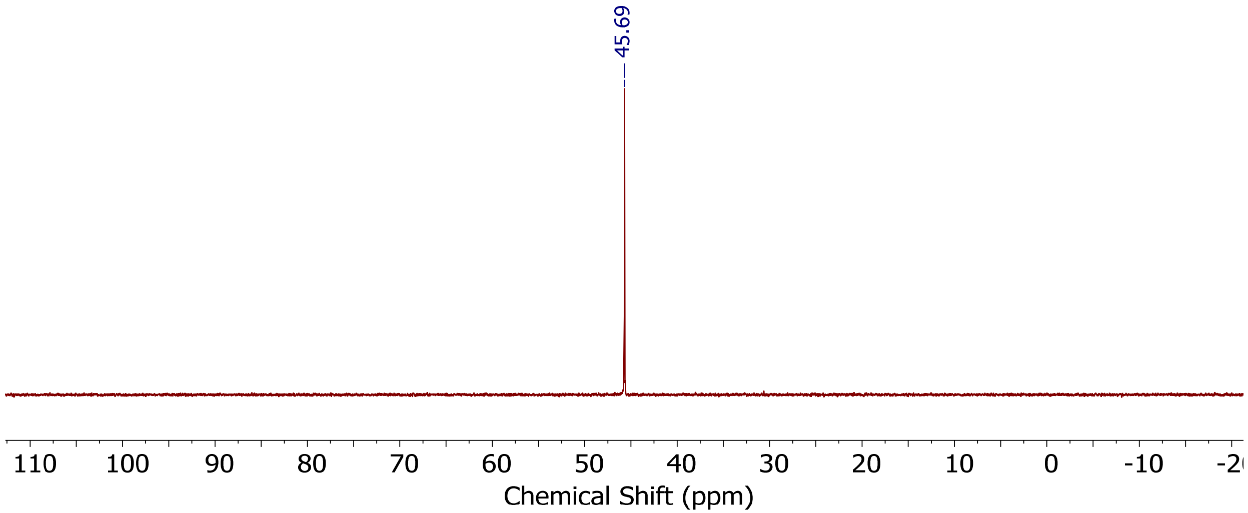

Figure S79. $^{31}P\{^{1}H\}$ NMR spectrum (121.5 MHz, benzene-d_6, 298 K) of $IrH_5(P^iPr_3)_2$ (**1**).

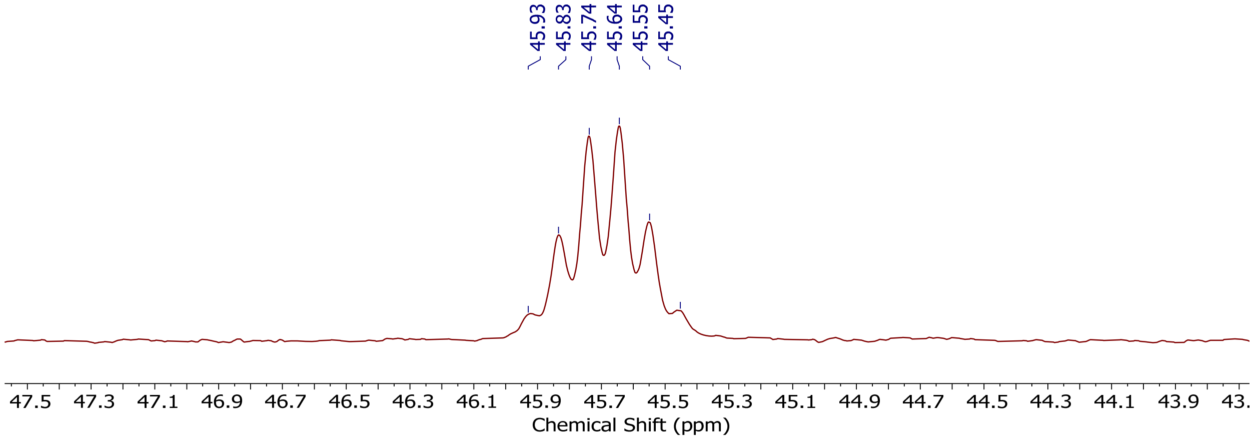

Figure S80. Off-resonance ^{31}P NMR spectrum (121.5 MHz, benzene-d_6, 298 K) of complex $IrH_5(P^iPr_3)_2$ (**1**).

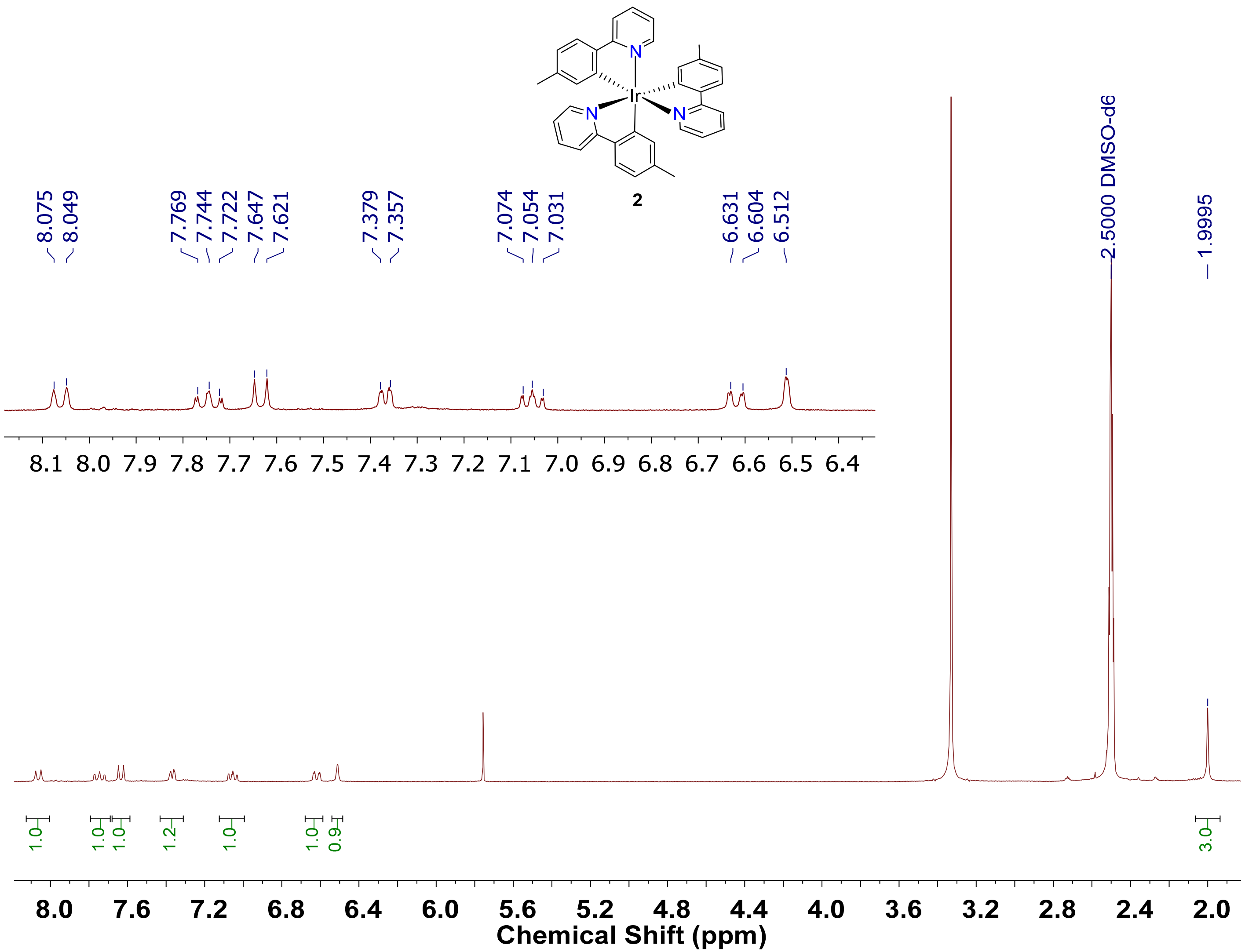

Figure S81. ^{1}H NMR spectrum (300 MHz, DMSO-d_6, 298 K) of *fac*-[Ir{κ[2]-*C*,*N*-[C_6MeH_3-py]}$_3$] (**2**).

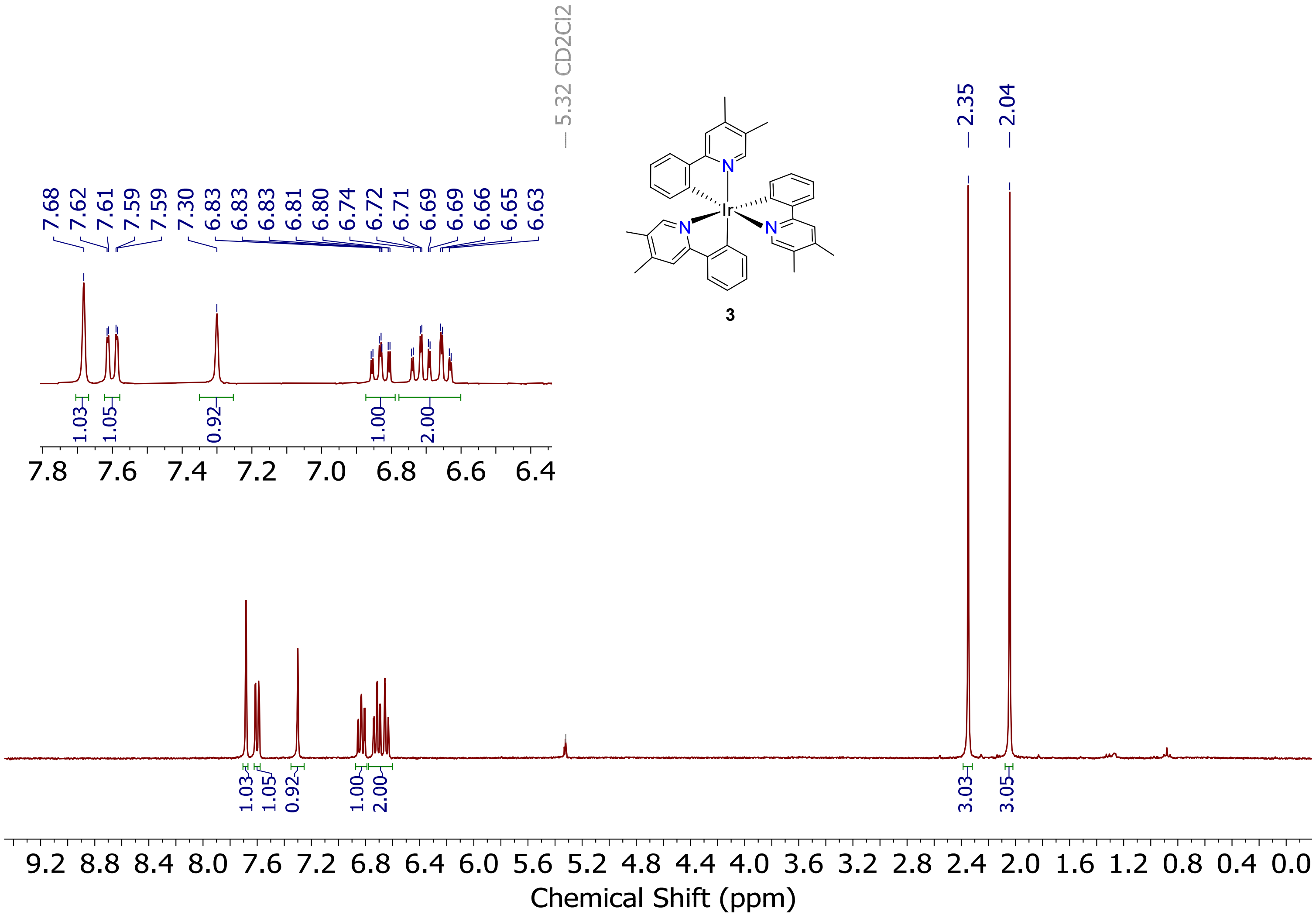

Figure S82. ^{1}H NMR spectrum (300 MHz, CD_2Cl_2, 298 K) of *fac*-[Ir{κ^2-*C,N*-[C_6H_4-pyMe$_2$]}$_3$] (**3**).

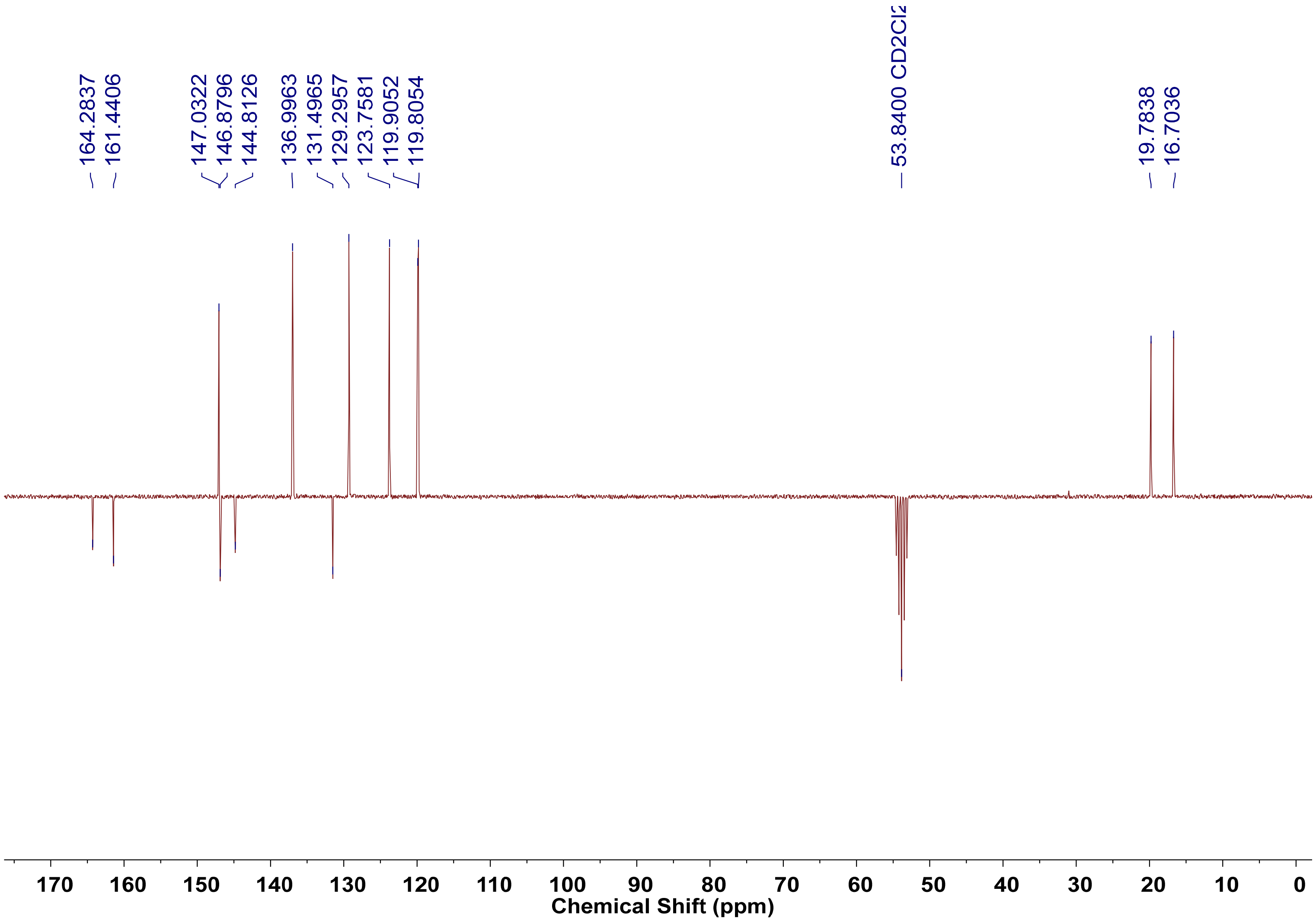

Figure S83. $^{13}C\{^1H\}$-apt NMR spectrum (75.45 MHz, CD_2Cl_2, 298 K) of *fac*-[Ir{κ^2-*C,N*-[C_6H_4-pyMe$_2$]}$_3$] (**3**).

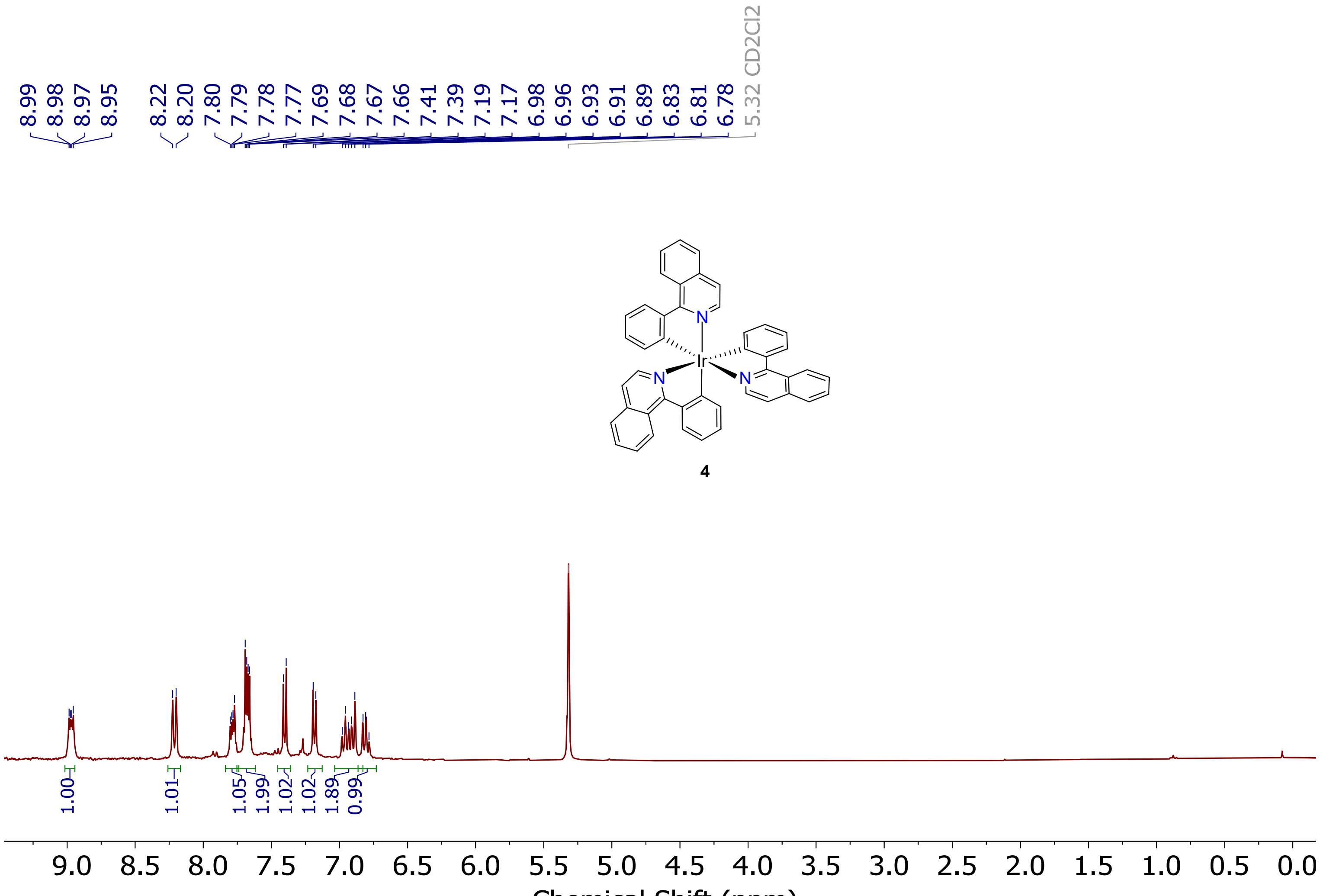

Figure S84. ^{1}H NMR spectrum (300 MHz, CD_2Cl_2, 298 K) of *fac*-[Ir{κ^2-*C,N*-[C_6H_4-Isoqui]}$_3$] (**4**).

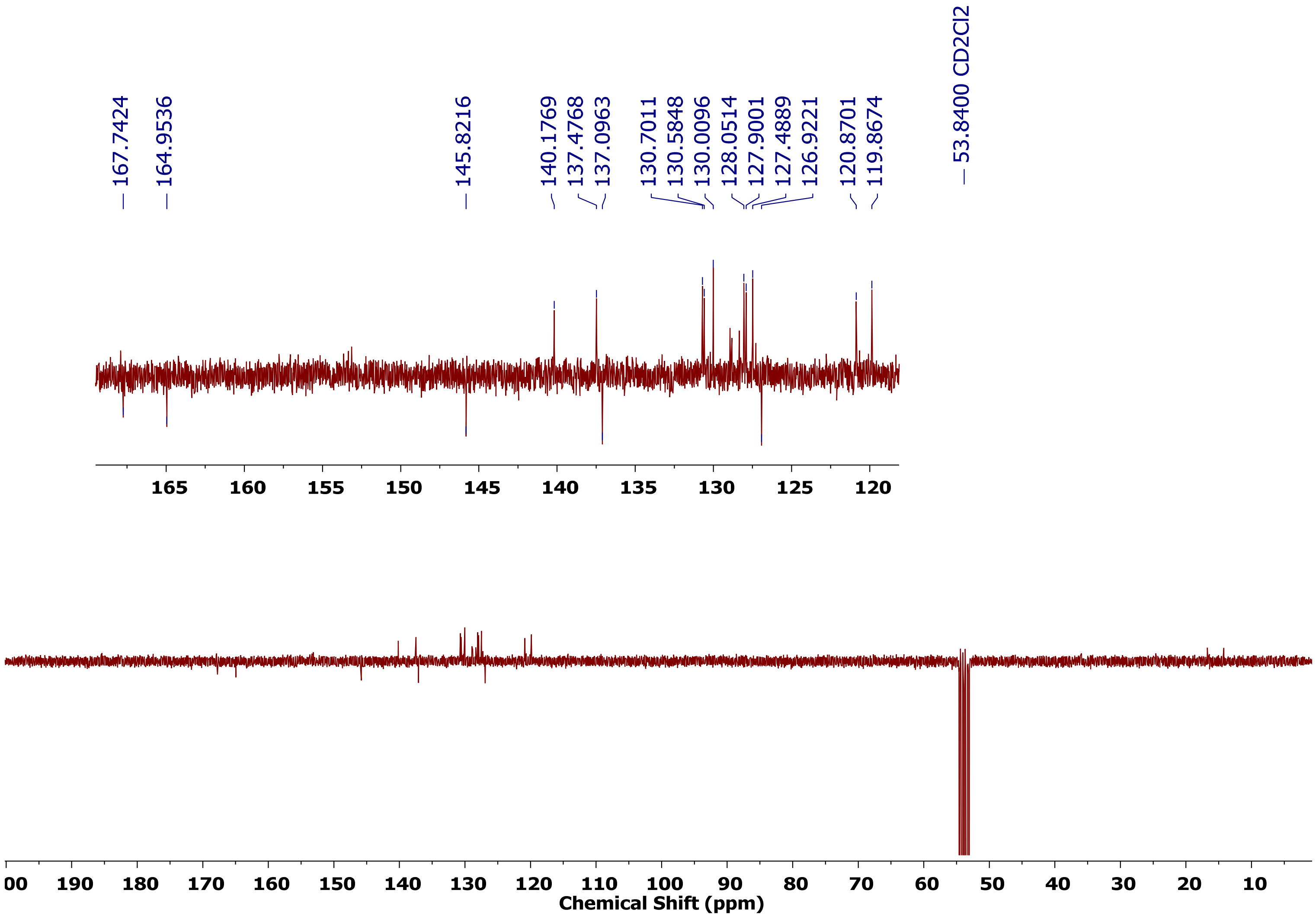

Figure S85. $^{13}C\{^{1}H\}$-apt NMR spectrum (75.45 MHz, CD_2Cl_2, 298 K) of *fac*-[Ir{κ^2-*C,N*-[C_6H_4-Isoqui]}$_3$] (**4**).

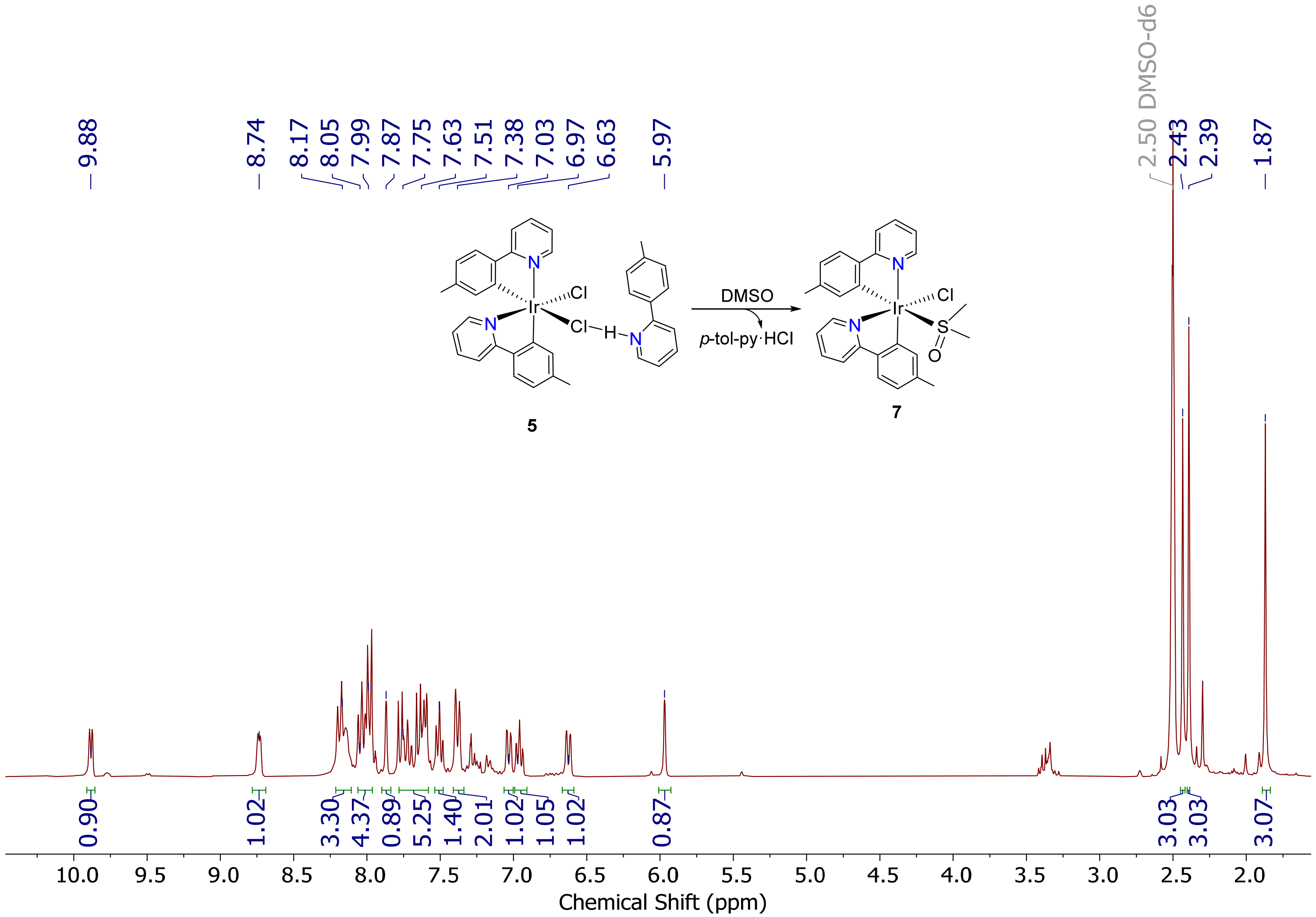

Figure S86. ^{1}H NMR spectrum (300 MHz, dmso-*d*$_6$, 298 K) of IrCl{κ^2-*C,N*-[C$_6$MeH$_3$-py]}$_2${κ^1-*Cl*-[Cl-H-py-C$_6$MeH$_4$]} (**5**). In these conditions, 2-(*p*-tolyl)pyridinium chloride is released and complex *cis*-[IrCl{κ^2-*C,N*-[C$_6$MeH$_3$-py]}$_2${κ^1-*S*-[S(O)Me$_2$]} (**7**) is formed.

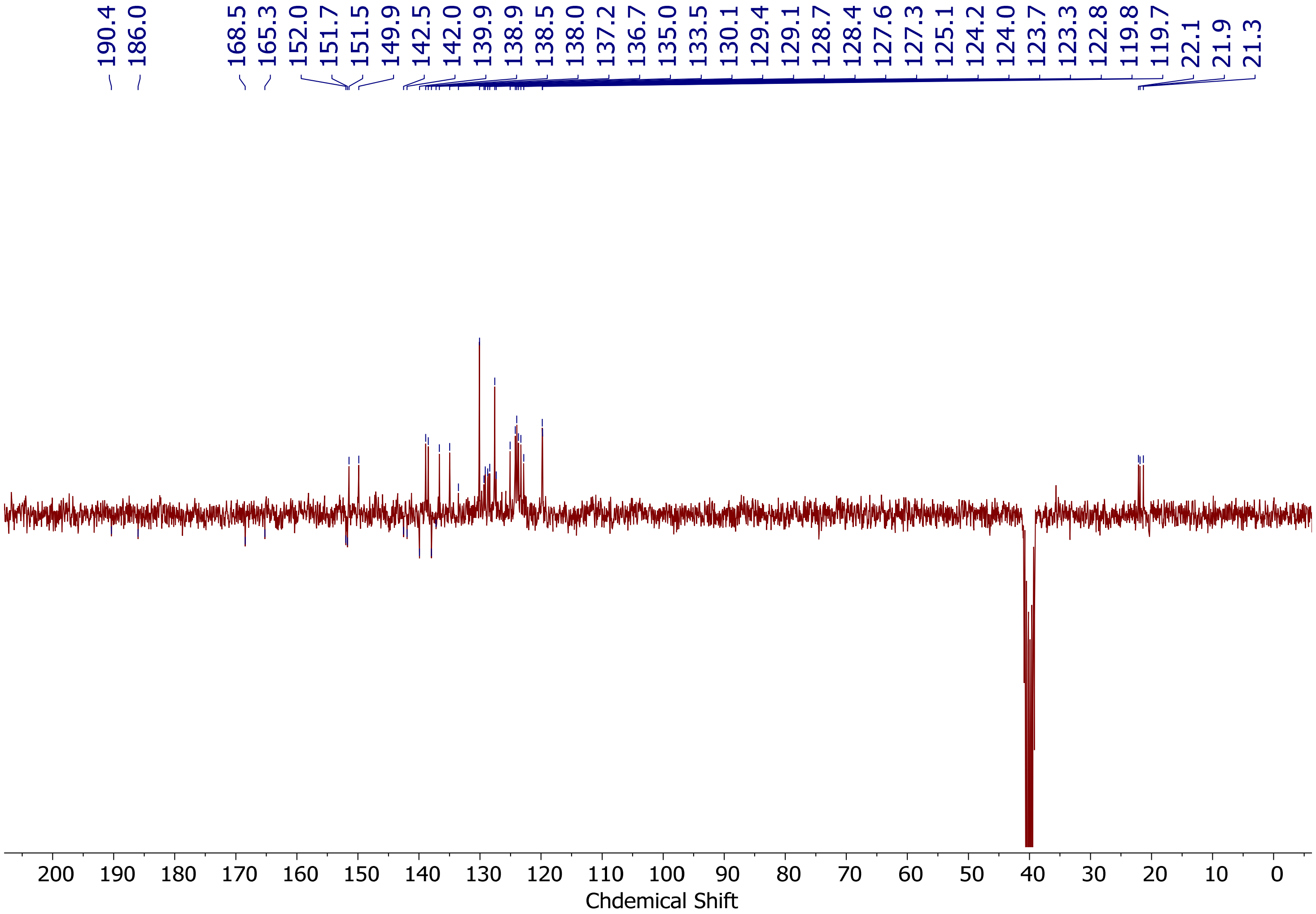

Figure S87. $^{13}C\{^{1}H\}$-apt NMR spectrum (75.45 MHz, dmso-d_6, 298 K) of compound of compound IrCl{κ²-*C,N*-[C_6MeH_3-py]}$_2${κ¹-*Cl*-[Cl-H-py-C_6MeH_4]} (**5**). In these conditions, 2-(*p*-tolyl)pyridinium chloride is released and complex *cis*-[IrCl{κ²-*C,N*-[C_6MeH_3-py]}$_2${κ¹-*S*-[S(O)Me_2]} (**7**) is formed.

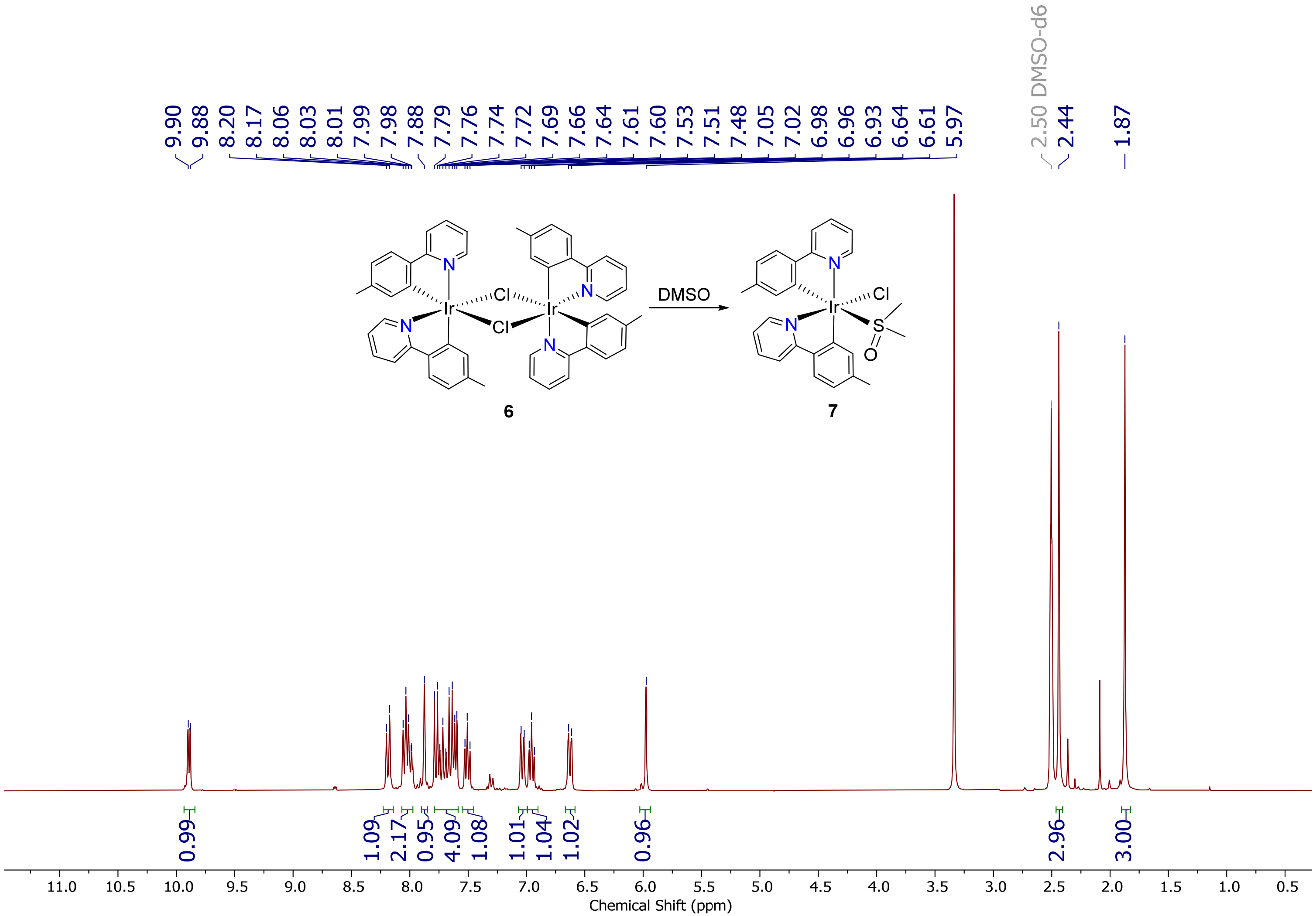

Figure S88. ^{1}H NMR spectrum (300 MHz, dmso-d_6, 298 K) of compound *cis*-[Ir(μ-Cl){κ[2]-*C,N*-[C_6MeH_3-py]}$_2$]$_2$ (**6**). In these conditions complex *cis*-[IrCl{κ[2]-*C,N*-[C_6MeH_3-py]}$_2${κ[1]-*S*-[S(O)Me_2]} (**7**) is formed.

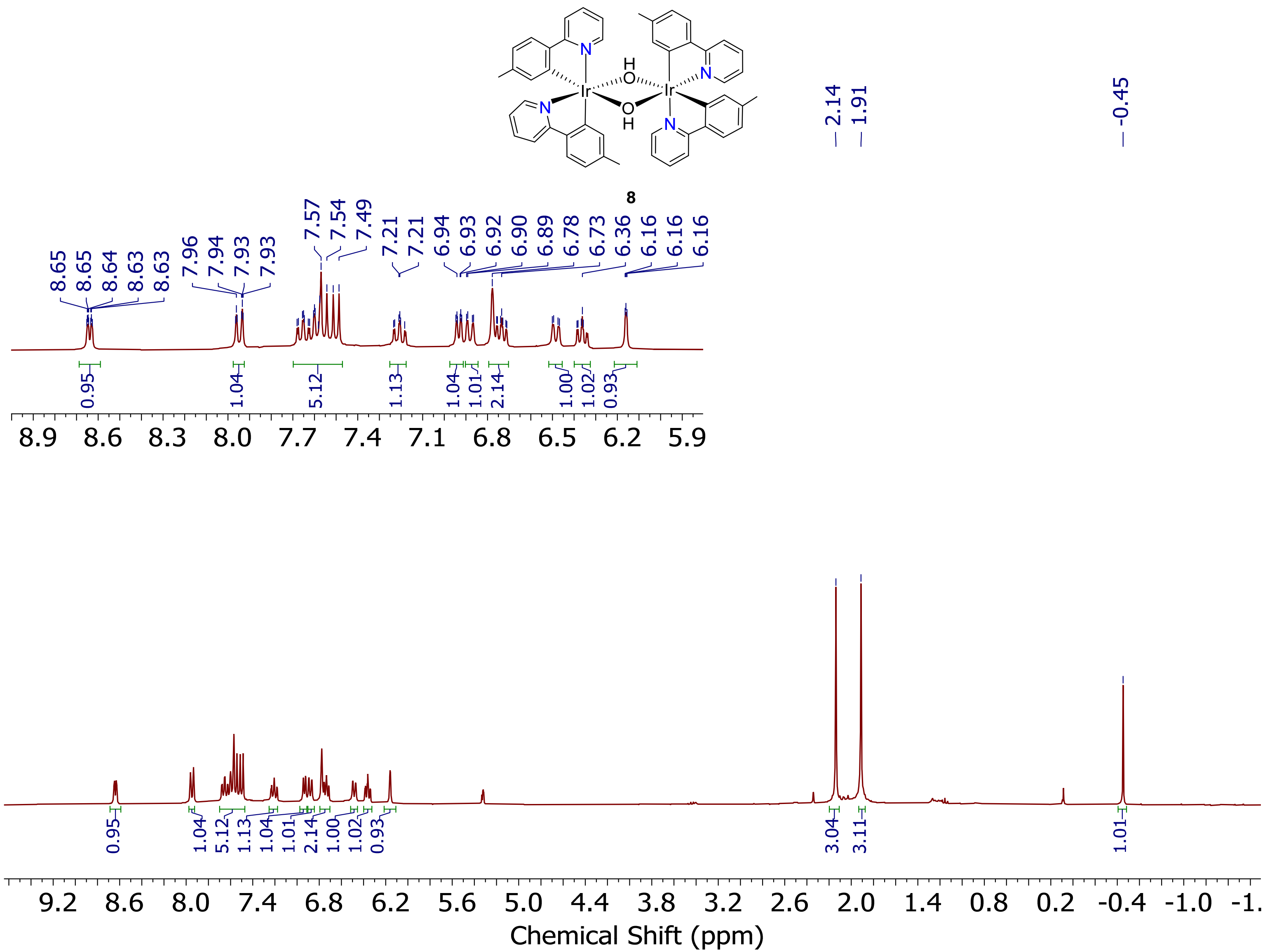

Figure S89. ^{1}H NMR spectrum (300 MHz, CD_2Cl_2, 298 K) of *cis*-[Ir(μ-OH){κ[2]-*C*,*N*-[C_6MeH_3-py]}$_2$]$_2$ (**8**).

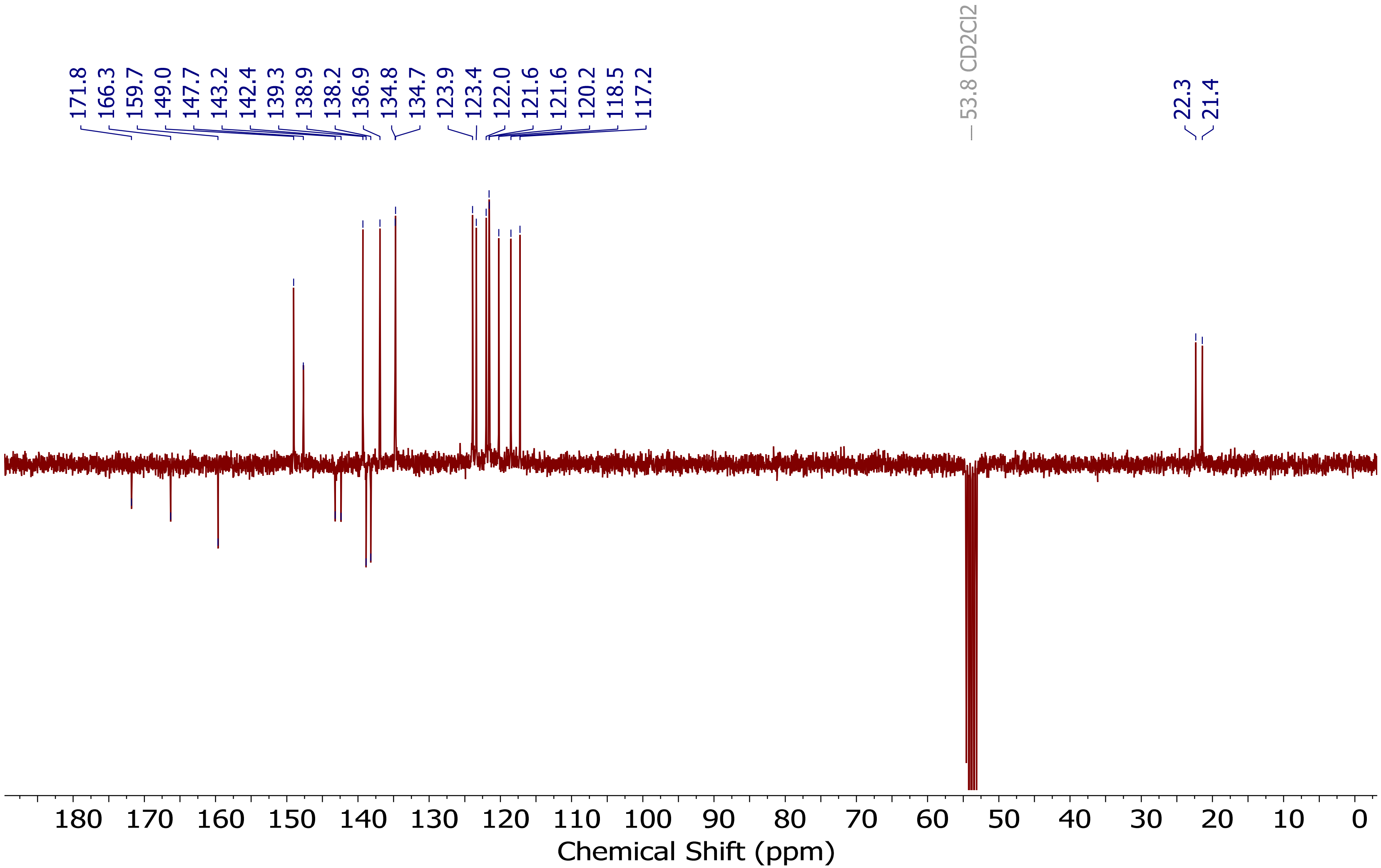

Figure S90. $^{13}C\{^1H\}$-apt NMR spectrum (75.45 MHz, CD_2Cl_2, 298 K) of *cis*-[Ir(μ-OH){κ^2-*C,N*-[C_6MeH_3-py]}$_2$]$_2$ (**8**).

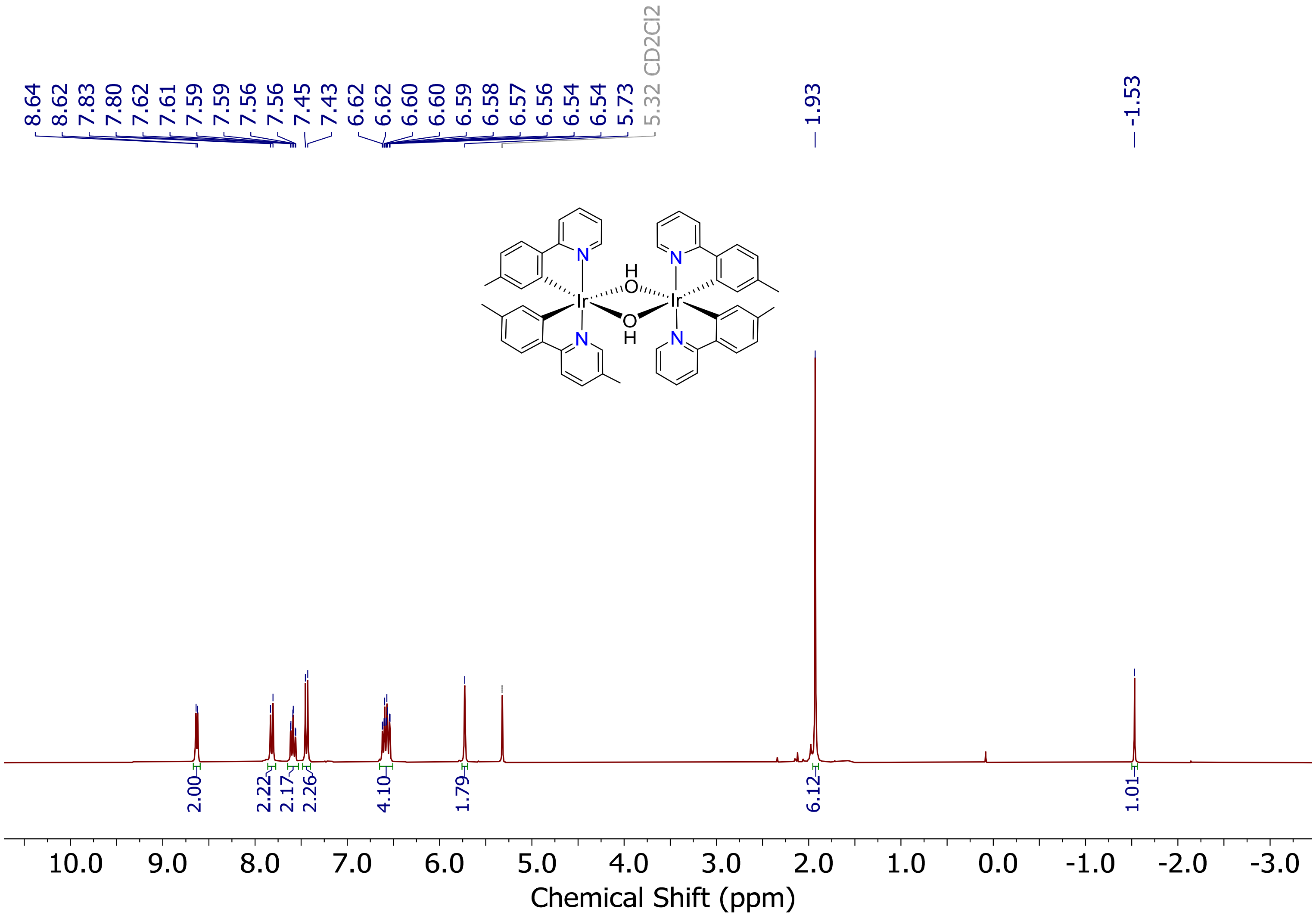

Figure S91. ^{1}H NMR spectrum (300 MHz, CD_2Cl_2, 298 K) of *trans*-[Ir(μ-OH){κ[2]-*C,N*-[C_6MeH_3-py]}$_2$]$_2$.

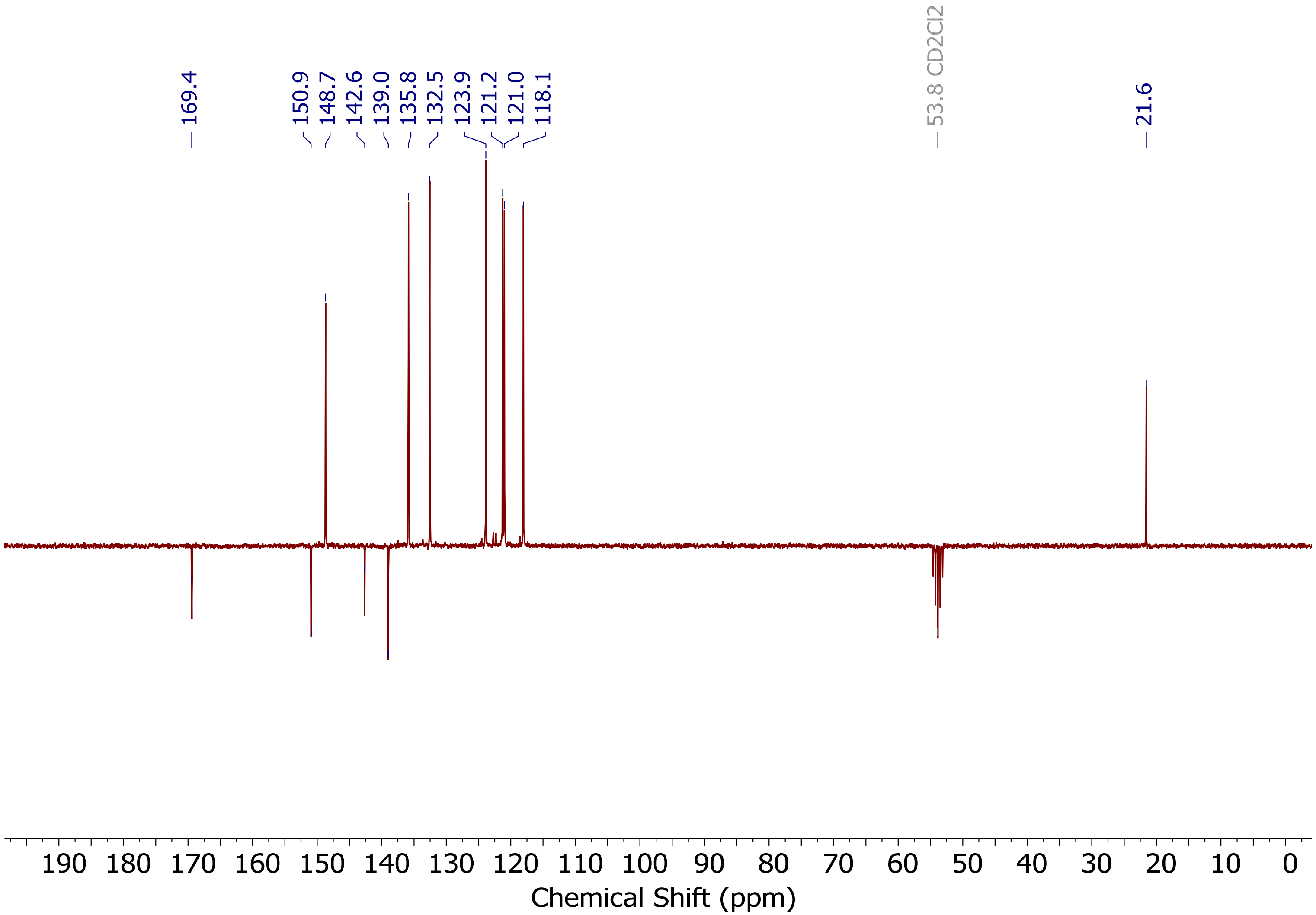

Figure S92. $^{13}C\{^1H\}$-apt NMR spectrum (75.45 MHz, CD_2Cl_2, 298 K) of *trans*-[Ir(μ-OH){κ²-*C,N*-[C_6MeH_3-py]}₂]₂.

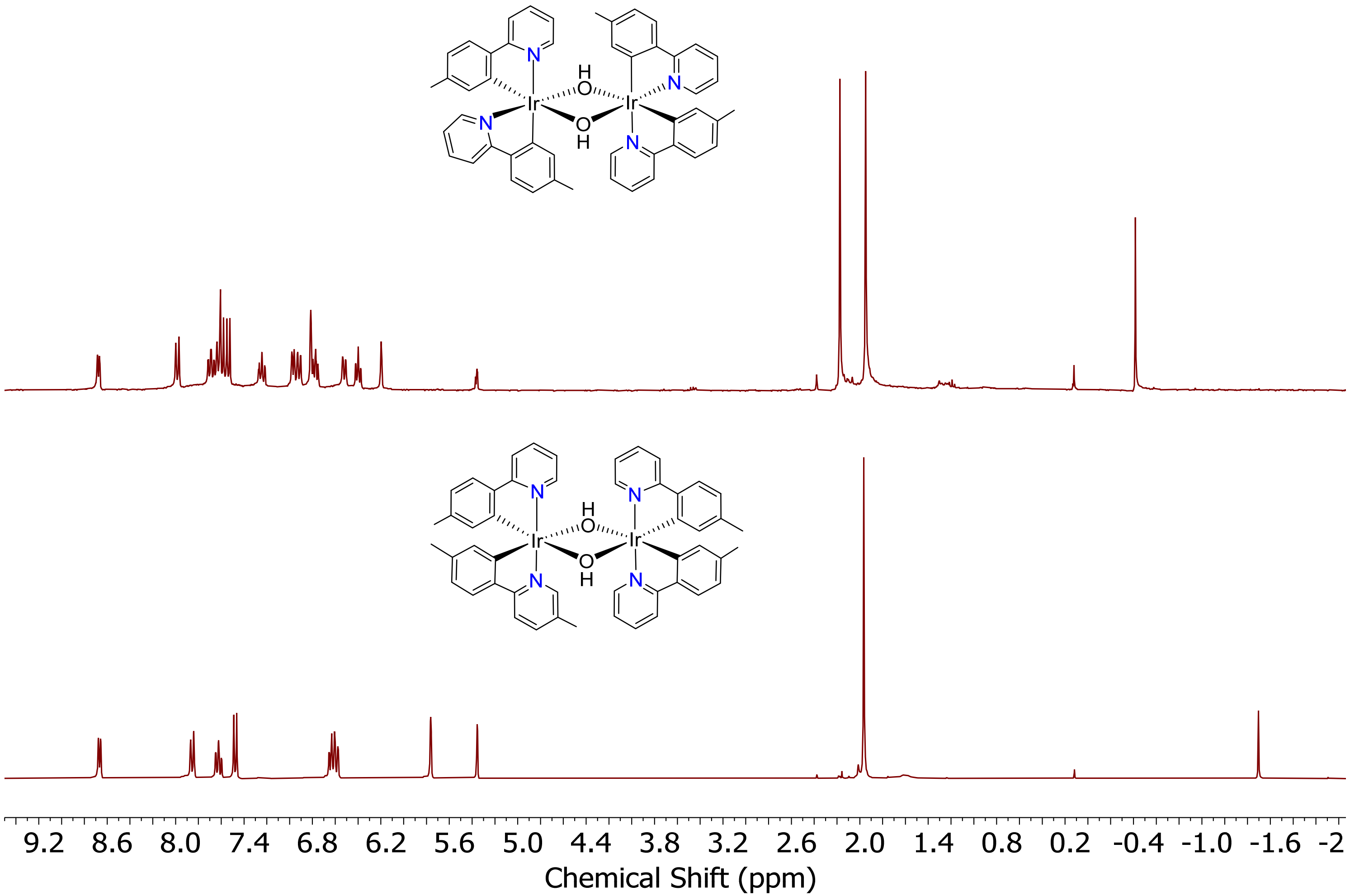

Figure S93. (top) ^{1}H NMR spectrum (300 MHz, CD_2Cl_2, 298 K) of *cis*-[Ir(μ-OH){κ[2]-*C,N*-[C_6MeH_3-py]}$_2$]$_2$ (**8**). (bottom) ^{1}H NMR spectrum (300 MHz, CD_2Cl_2, 298 K) of *trans*-[Ir(μ-OH){κ[2]-*C,N*-[C_6MeH_3-py]}$_2$]$_2$.

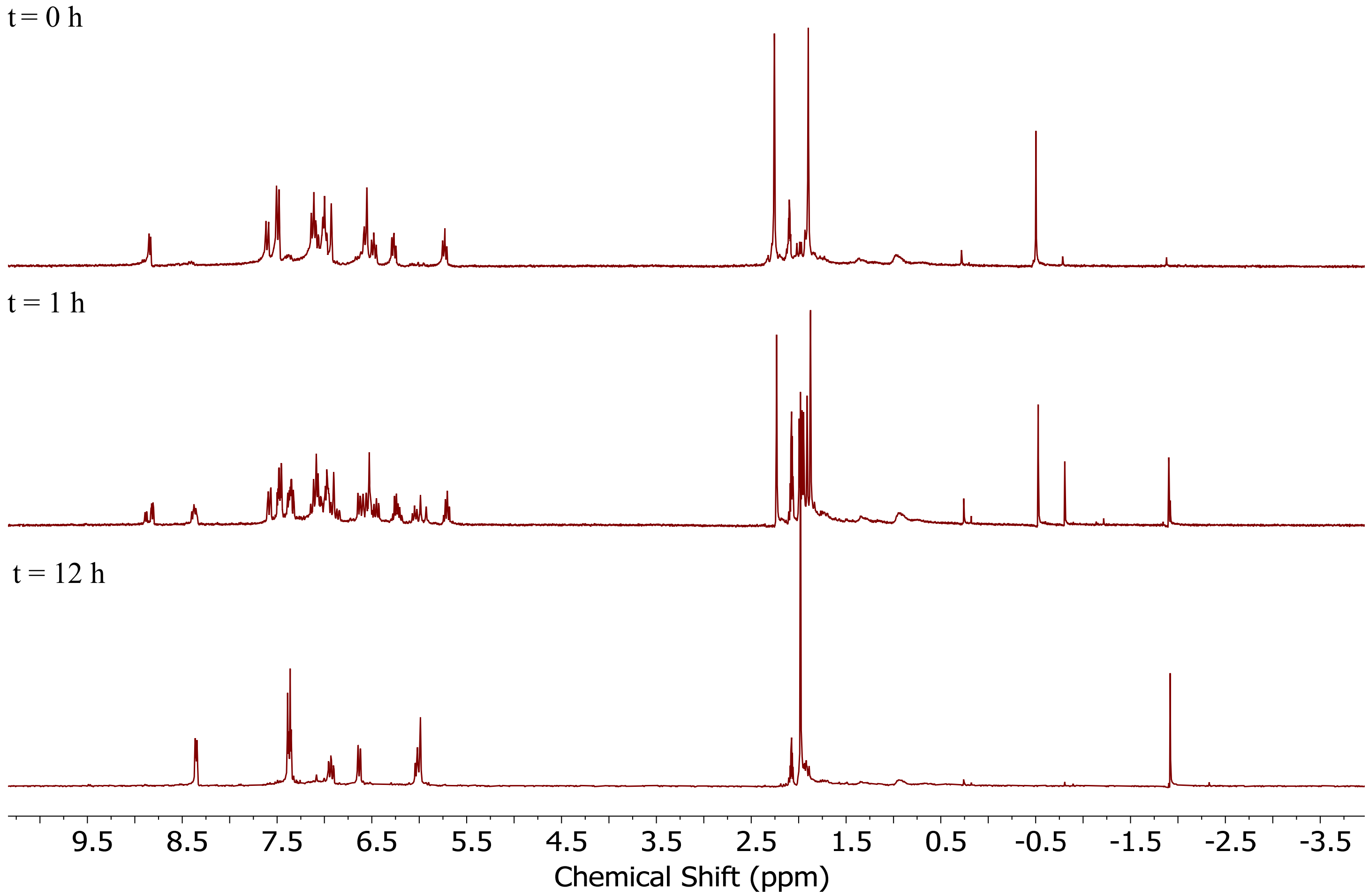

Figure S94. ^{1}H NMR spectra (300 MHz, toluene-d_8, 298 K) showing the isomerization of complex *cis*-[Ir(μ-OH){κ[2]-*C,N*-[C_6MeH_3-py]}$_2$]$_2$ (**8**) (top) into *trans*-[Ir(μ-OH){κ[2]-*C,N*-[C_6MeH_3-py]}$_2$]$_2$ (bottom). The transformation is complete after 12 h at 100 ºC. (middle) ^{1}H NMR spectrum registered after 1 h.

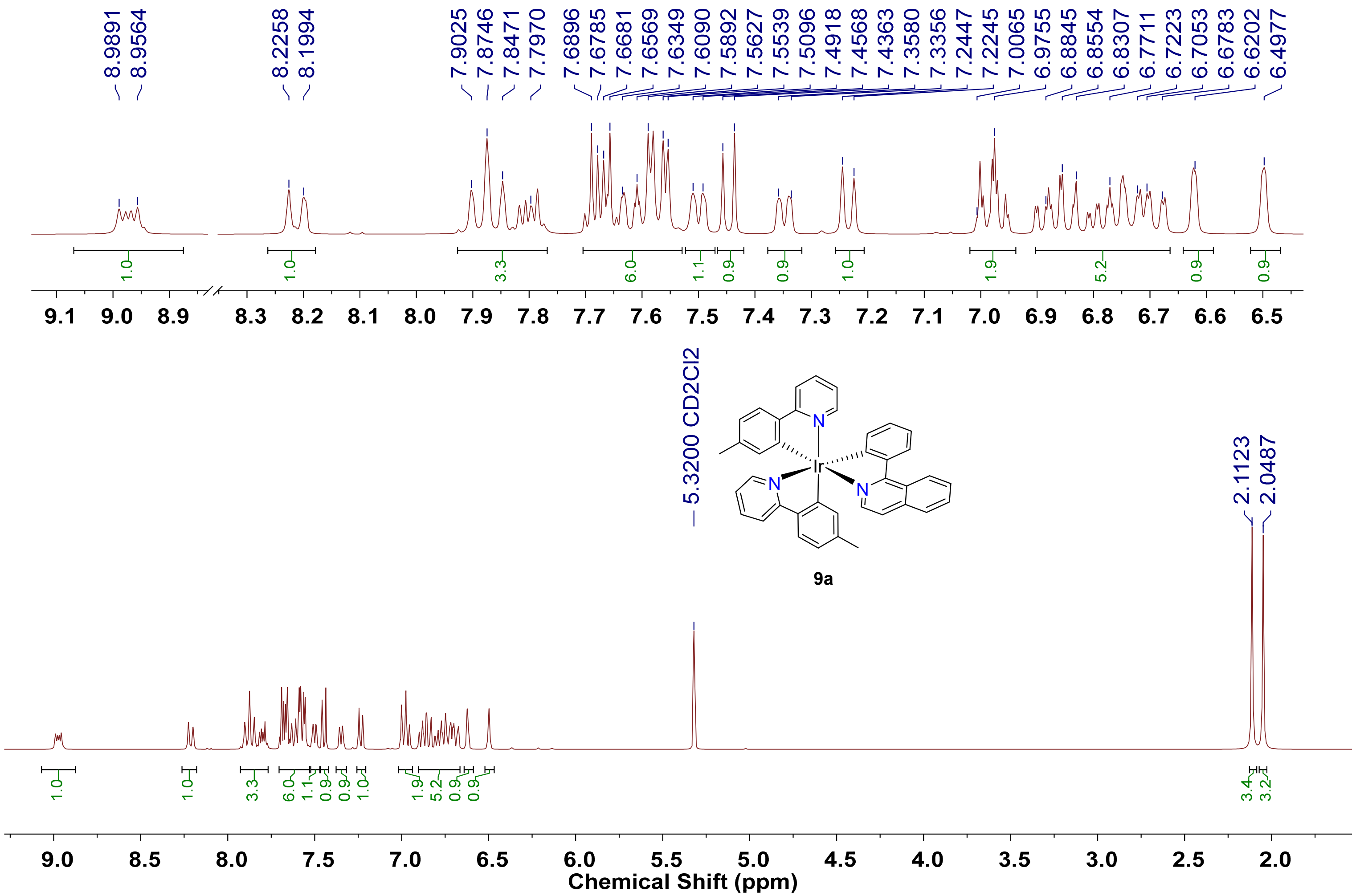

Figure S95. ^{1}H NMR spectrum (300 MHz, CD_2Cl_2, 298 K) of *cis*-[Ir{κ[2]-*C,N*-[C_6MeH_3-py]}$_2${κ[2]-*C,N*-[C_6H_4-Isoqui]} (**9a**).

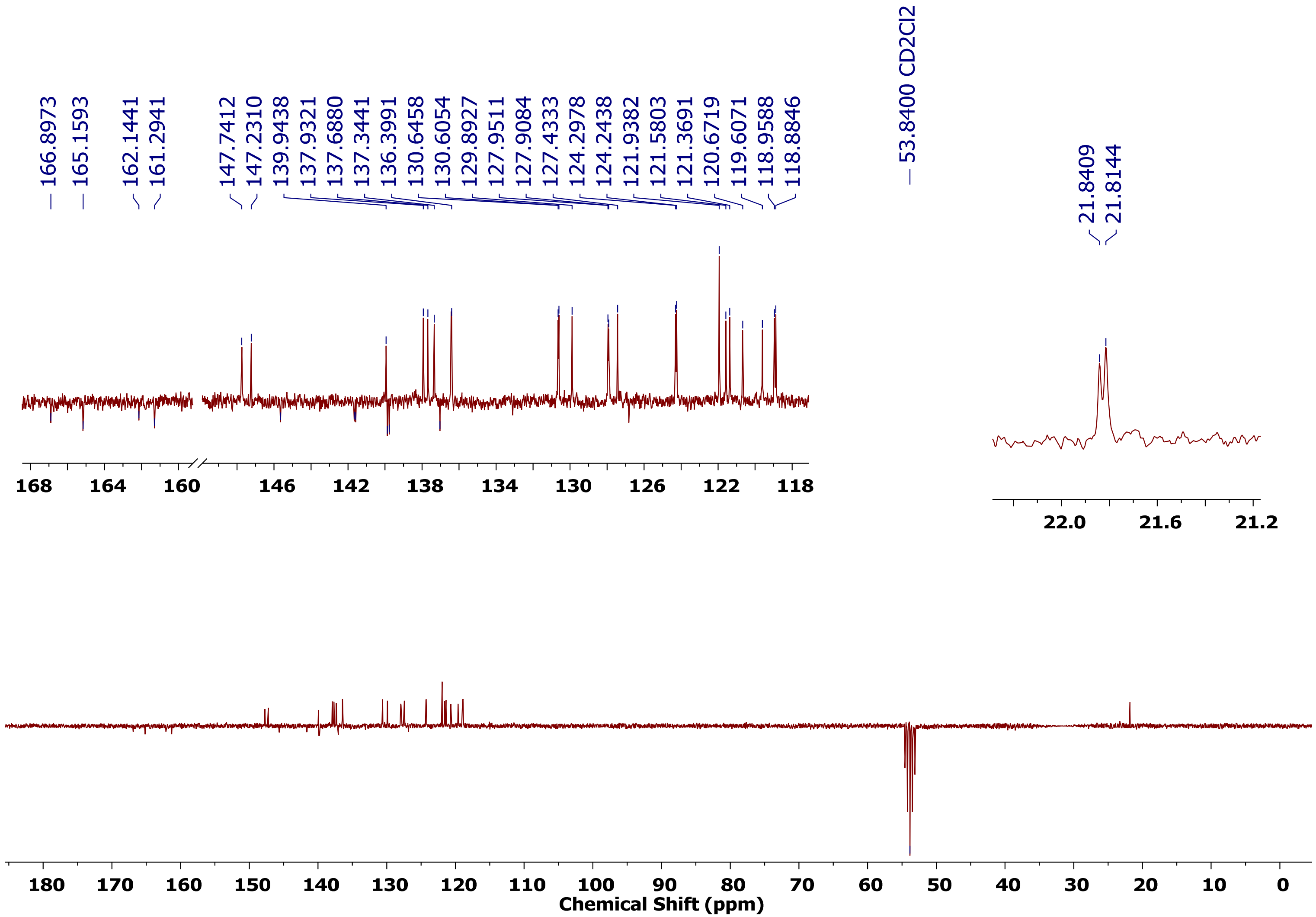

Figure S96. $^{13}C\{^1H\}$-apt NMR spectrum (75.45 MHz, CD_2Cl_2, 298 K) of *cis*-[Ir{κ^2-*C,N*-[C_6MeH_3-py]}$_2${κ^2-*C,N*-[C_6H_4-Isoqui]} (**9a**).

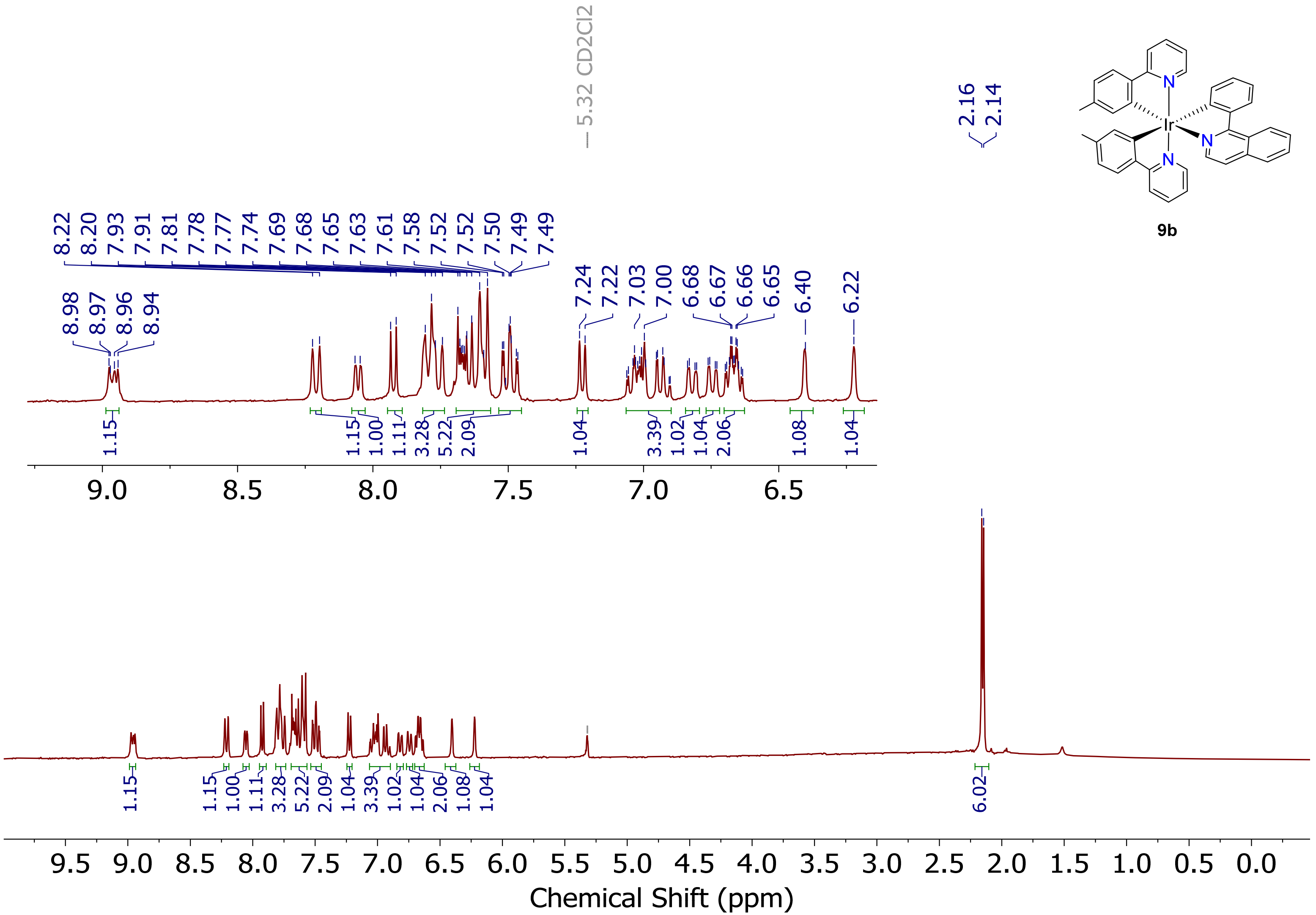

Figure S97. ^{1}H NMR spectrum (300 MHz, CD_2Cl_2, 298 K) of *trans*-[Ir{κ[2]-*C,N*-[C_6MeH_3-py]}$_2${κ[2]-*C,N*-[C_6H_4-Isoqui]} (**9b**).

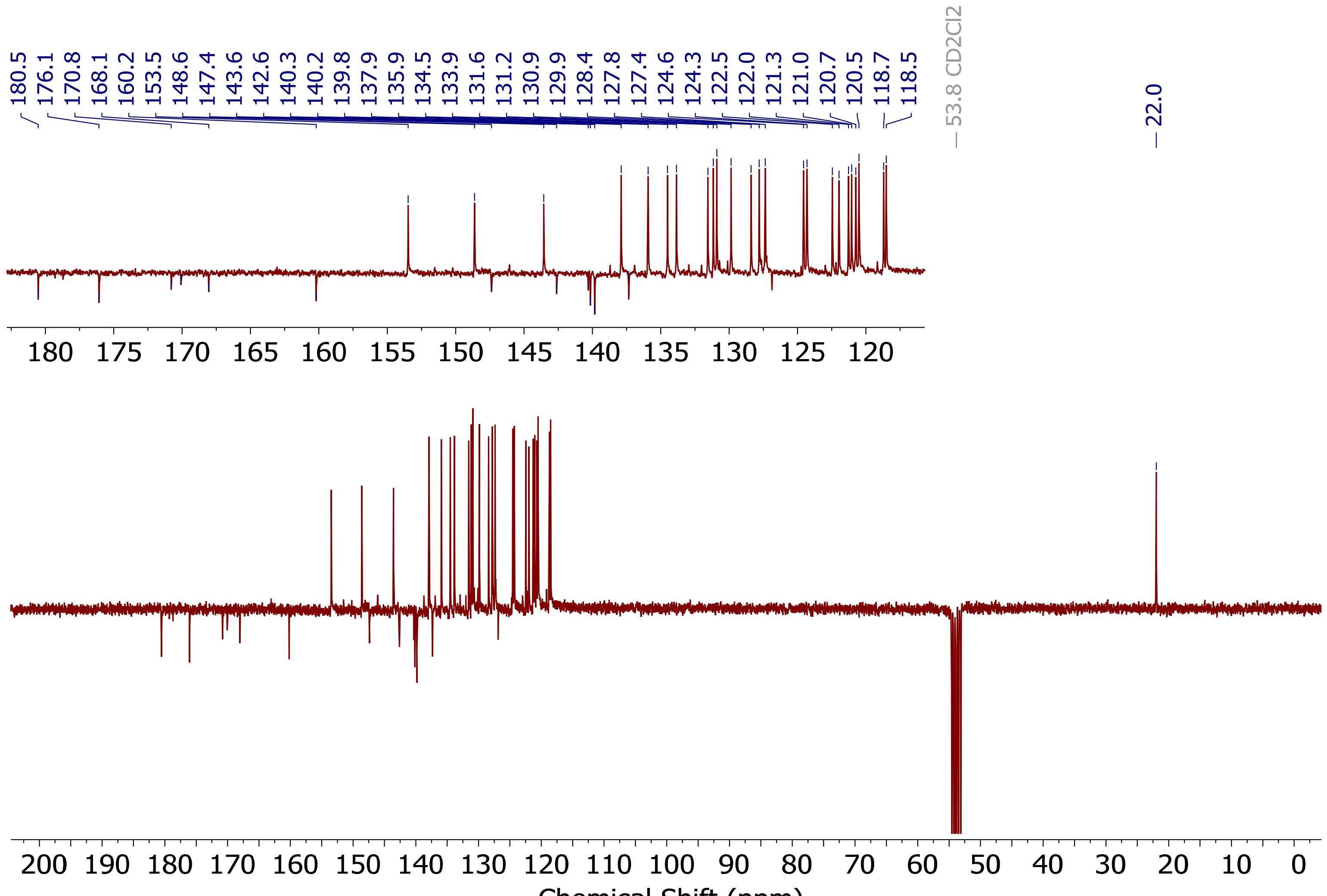

Figure S98. $^{13}C\{^1H\}$-apt NMR spectrum (75.45 MHz, CD_2Cl_2, 298 K) of *trans*-[Ir{κ^2-*C,N*-[C_6MeH_3-py]}$_2${κ^2-*C,N*-[C_6H_4-Isoqui]} (**9b**).

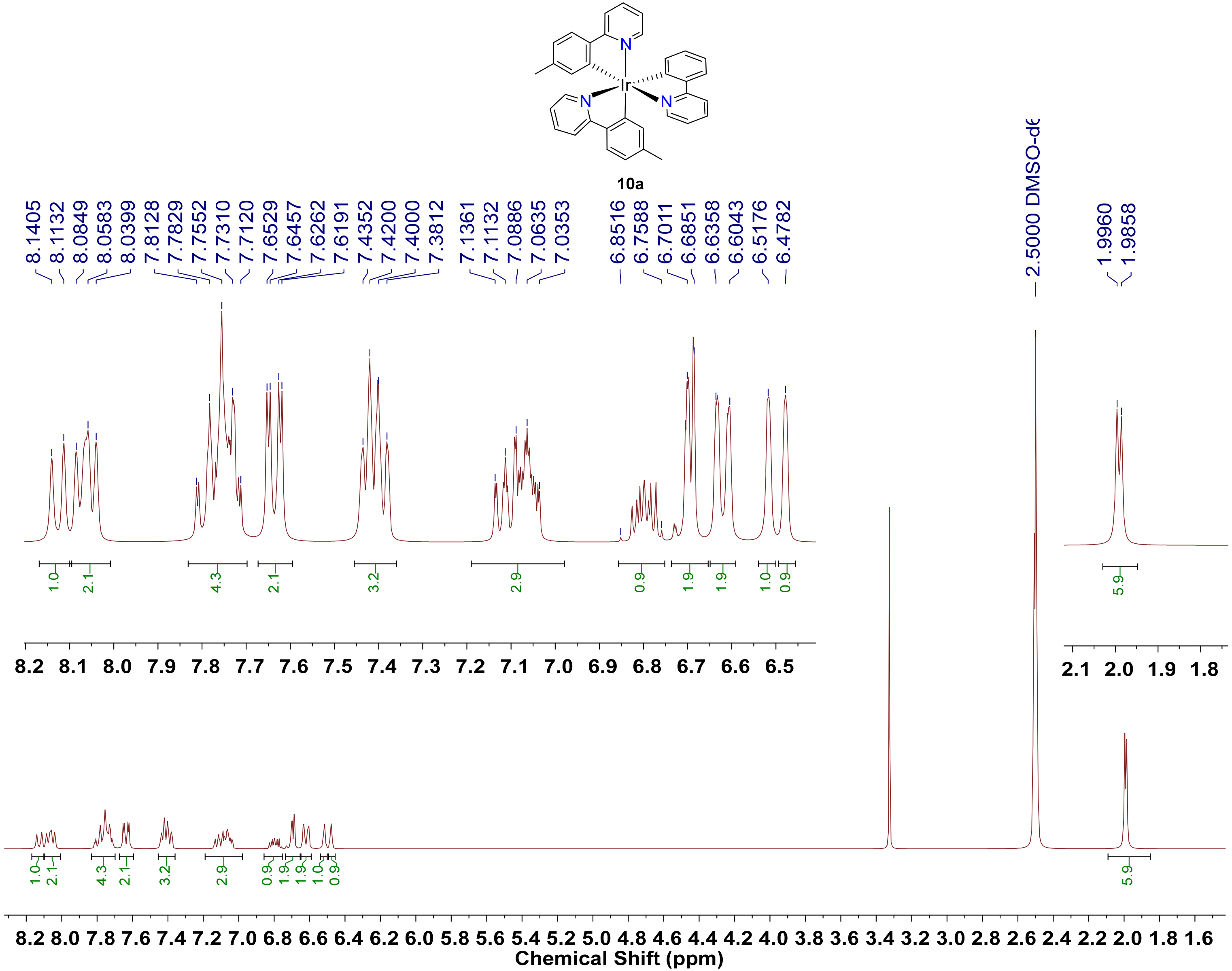

Figure S99. ^{1}H NMR spectrum (300 MHz, DMSO-d_6, 298 K) of *cis*-[Ir{κ^2-*C,N*-[C_6MeH_3-py]}$_2${κ^2-*C,N*-[C_6H_4-py]} (**10a**).

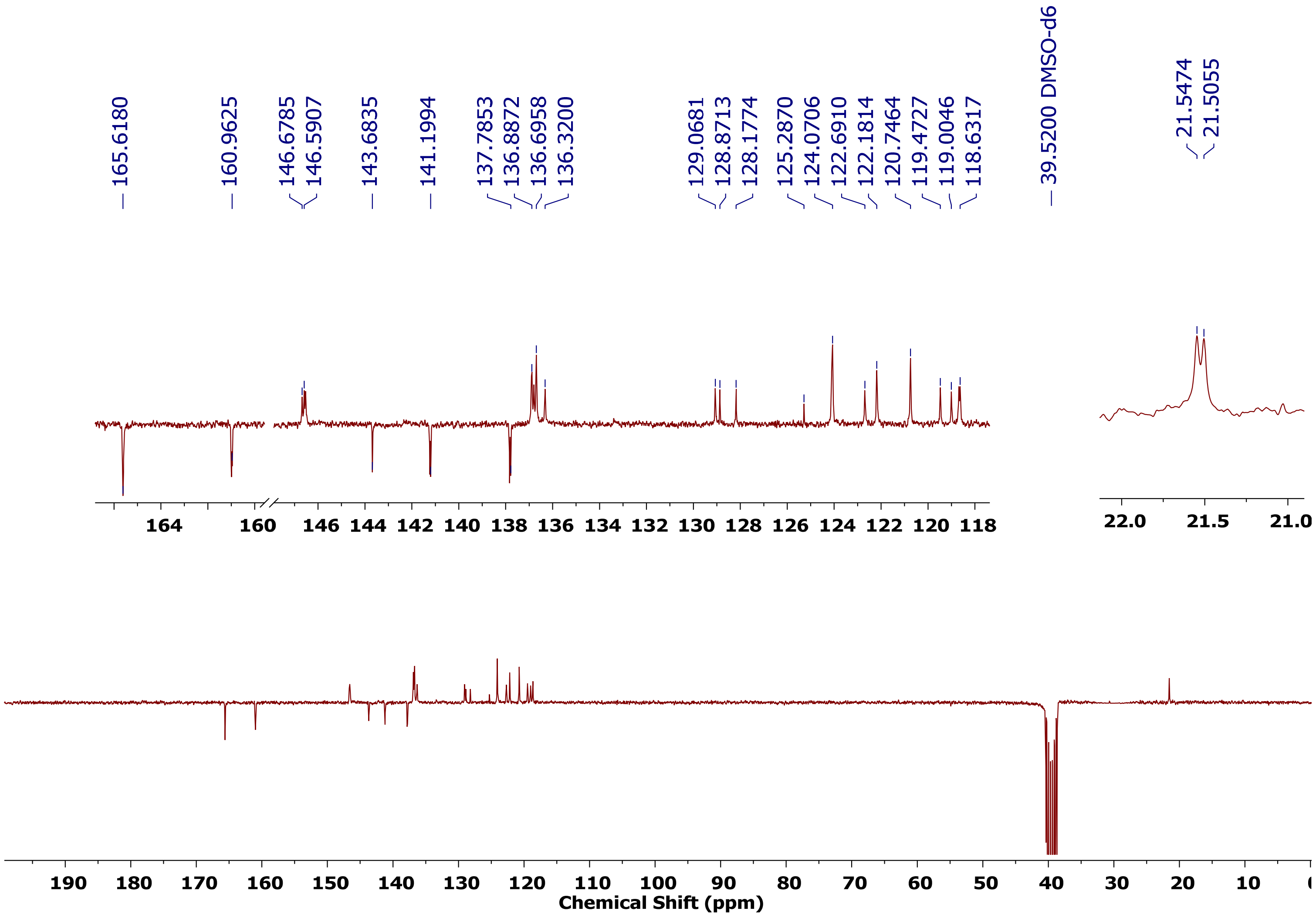

Figure S100. $^{13}C\{^1H\}$-apt NMR spectrum (75.45 MHz, DMSO-d_6, 298 K) of *cis*-[Ir{κ^2-*C,N*-[C_6MeH_3-py]}$_2${κ^2-*C,N*-[C_6H_4-py]} (**10a**).

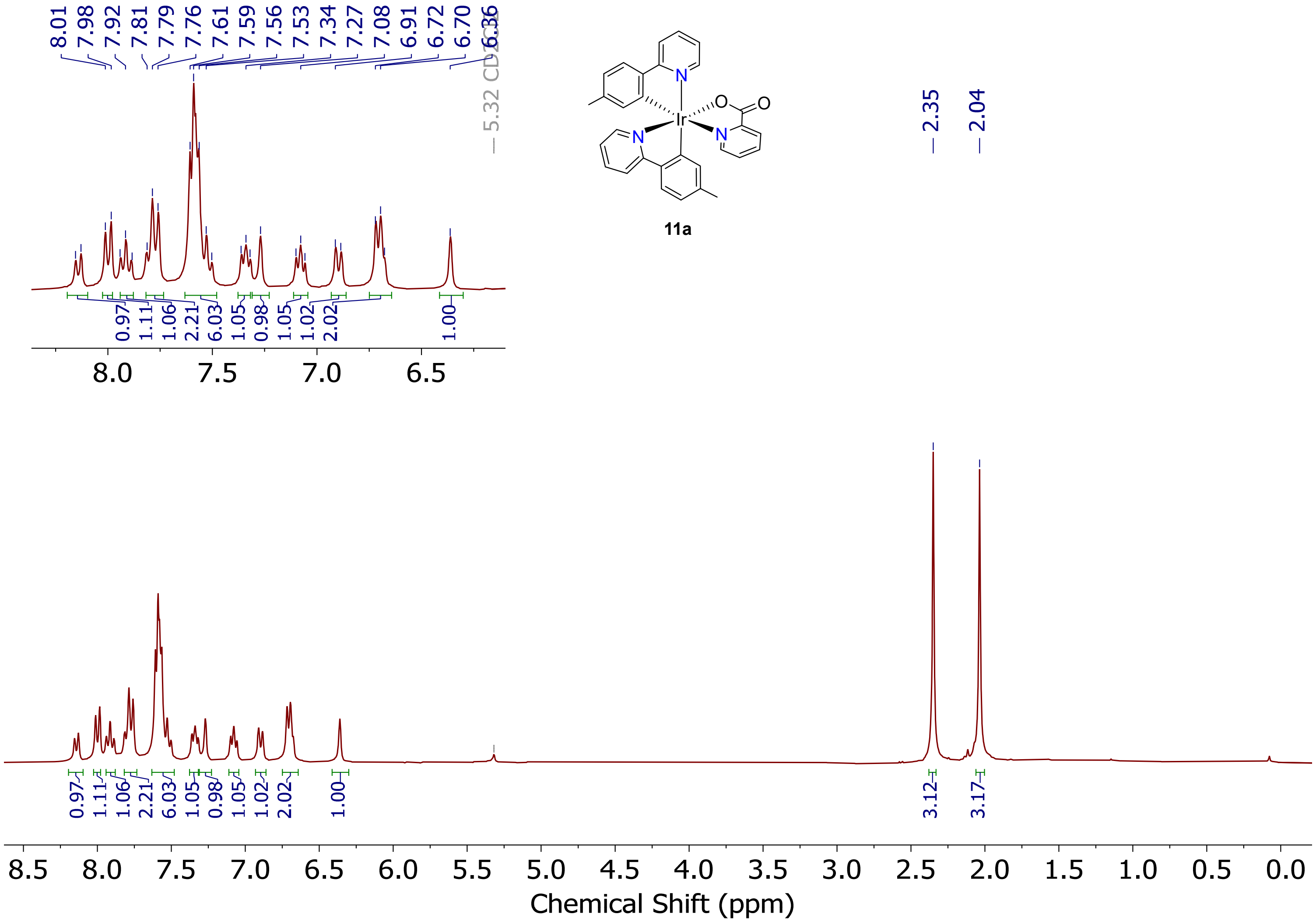

Figure S101. ^{1}H NMR spectrum (300 MHz, CD_2Cl_2, 298 K) of *cis*-Ir{κ^2-*C,N*-[C_6MeH_3-py]}$_2${κ^2-*O,N*-[OC(O)-py]} (**11a**).

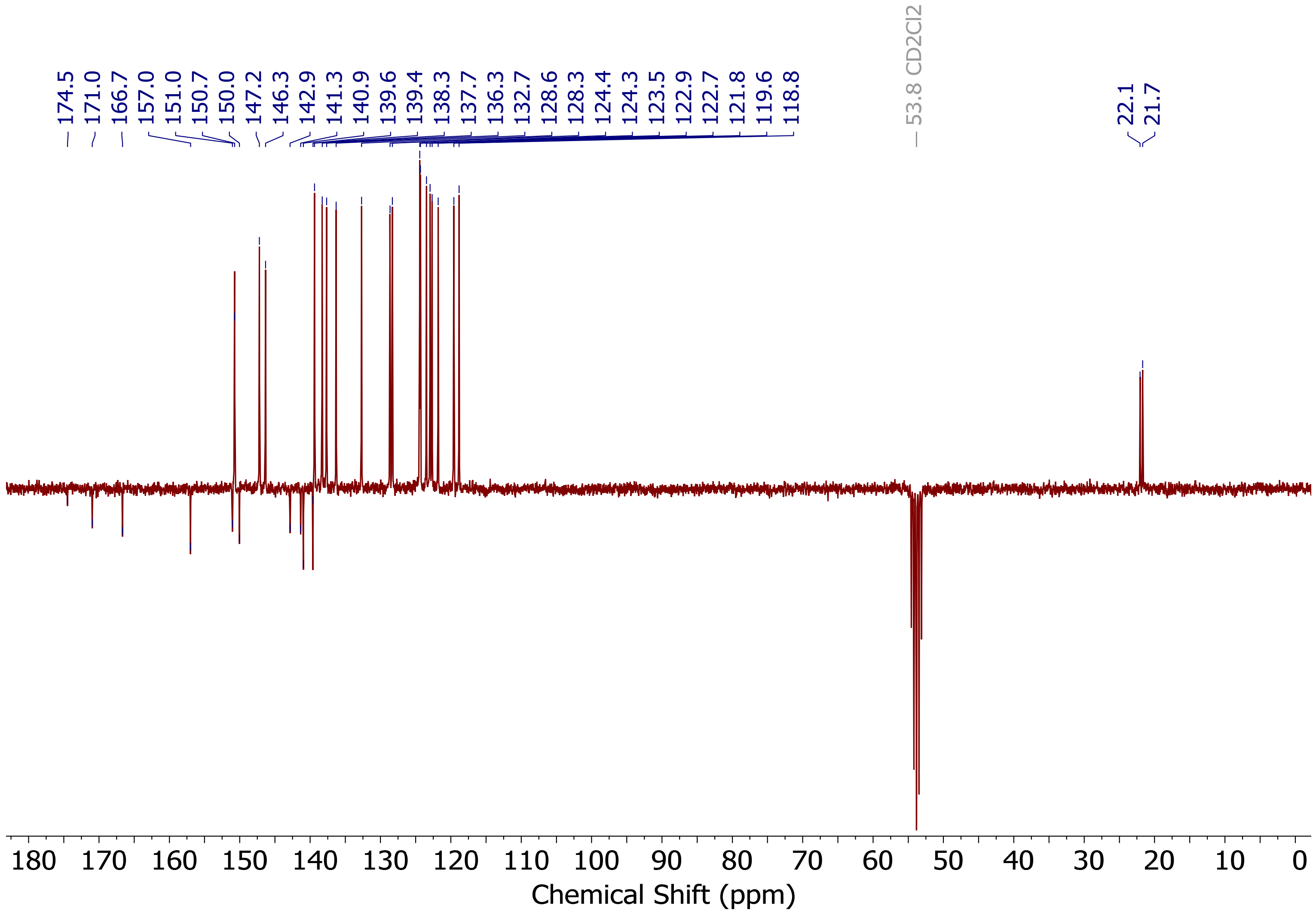

Figure S102. $^{13}C\{^1H\}$-apt NMR spectrum (75.45 MHz, CD_2Cl_2, 298 K) of *cis*-Ir{κ²-*C,N*-[C_6MeH_3-py]}$_2${κ²-*O,N*-[OC(O)-py]} (**11a**).

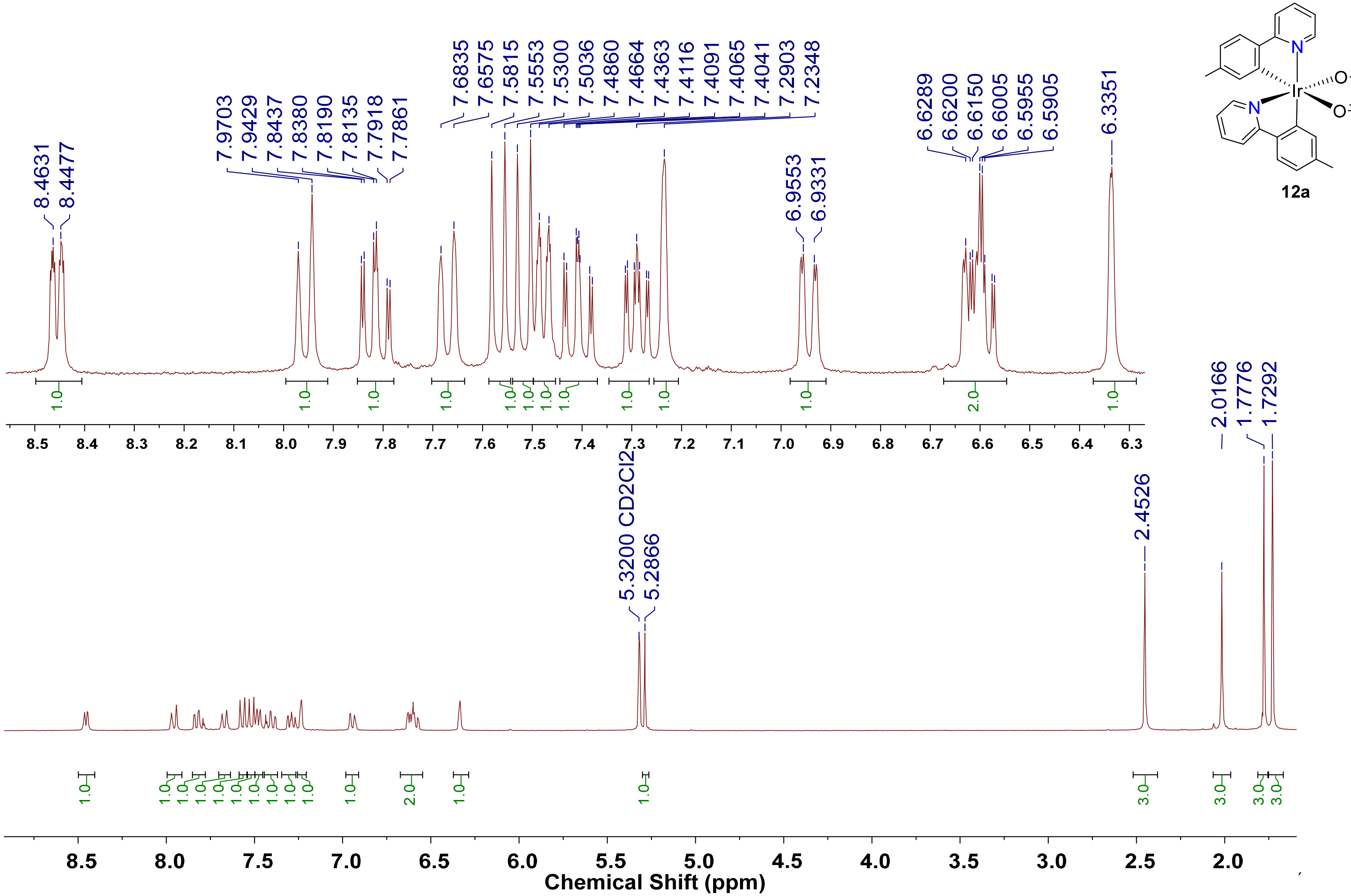

Figure S103. ^{1}H NMR spectrum (300 MHz, CD_2Cl_2, 298 K) of *cis*-Ir{κ[2]-*C,N*-[C_6MeH_3-py]}$_2${κ[2]-*O,O*-[acac]} (**12a**).

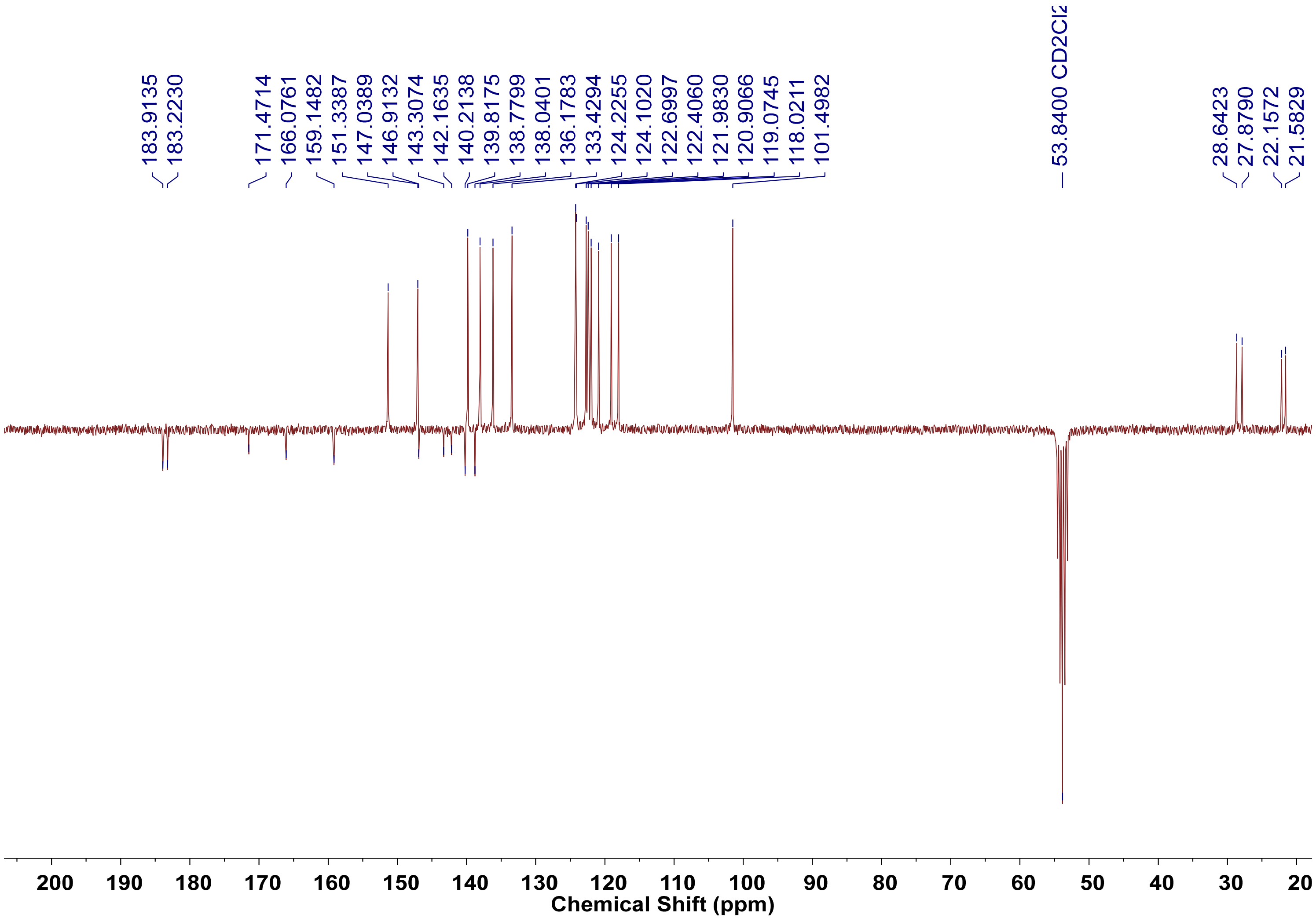

Figure S104. $^{13}C\{^1H\}$-apt NMR spectrum (75.45 MHz, CD_2Cl_2, 298 K) of *cis*-Ir{κ^2-*C,N*-[C_6MeH_3-py]}$_2${κ^2-*O,O*-[acac]} (**12a**).

• References

(1) Simpson, R. D.; Marshall, W. J.; Farischon, A. A.; Roe, D. C.; Grushin, V. V. Anionic Iridium Monohydrides. *Inorg. Chem.* **1999**, *38*, 4171-4173.

(2) SAINT+, version 6.01: Area-Detector Integration Software, Bruker AXS, Madison,WI, 2001

(3) Blessing, R. H. *Acta Crystallogr.* **1995**, *A51*, 33. SADABS: Area-detector absorption correction; Bruker- AXS, Madison, WI, 1996.

(4) SHELXL-2019/1. Sheldrick, G. M. *Acta Cryst.* **2008**, *A64*, 112-122.

(5) (a) Lee, C.; Yang, W.; Parr, R. G. Development of the Colle-Salvetti correlation energy formula into a functional of the electron density. *Phys. Rev. B* **1988**, *37*, 785-789. (b) Becke, A. D. Density-functional exchange-energy approximation with correct asymptotic behavior. *J. Chem. Phys.* **1993**, *98*, 5648-5652. (c) Stephens, P. J.; Devlin, F. J.; Chabalowski, C. F.; Frisch, M. J. Ab Initio Calculation of Vibrational Absorption and Circular Dichroism Spectra Using Density Functional Force Fields. *J. Phys. Chem.* **1994**, *98*, 11623-11627.

(6) Grimme, S.; Antony, J.; Ehrlich, S.; Krieg, H. A consistent and accurate ab initio parametrization of density functional dispersion correction (DFT-D) for the 94 elements H-Pu. *J. Chem. Phys.* **2010**, *132*, 154104.

(7) Gaussian 09, Revision D.01, Frisch, M. J.; Trucks, G. W.; Schlegel H. B.; Scuseria, G. E.; Robb, M. A.; Cheeseman, J. R.; Scalmani, G.; Barone, V.; Mennucci, B.; Petersson, G. A.; Nakatsuji, H.; Caricato, M.; Li, X.; Hratchian, H. P.; Izmaylov, A. F.; Bloino, J.; Zheng, G.; Sonnenberg, J. L.; Hada, M.; Ehara, M.; Toyota, K.; Fukuda, R.; Hasegawa, J.; Ishida, M.; Nakajima, T.; Honda, Y.; Kitao, O.; Nakai, H.; Vreven, T.; Montgomery, J. A.; Peralta, Jr., J. E.; Ogliaro, F.; Bearpark, M.; Heyd, J. J.; Brothers, E.; Kudin, K. N.; Staroverov, V. N.; Keith, T.; Kobayashi, R.; Normand, J.; Raghavachari, K.; Rendell, A.; Burant, J. C.; Iyengar, S. S.; Tomasi, J.; Cossi, M.; Rega, N.; S43 Millam, J. M.; Klene, M.; Knox, J. E.; Cross, J. B.; Bakken, V.; Adamo, C.; Jaramillo, J.; Gomperts, R.; Stratmann, R. E.; Yazyev, O.; Austin, A. J.; Cammi, R.; Pomelli, C.; Ochterski, J. W.; Martin, R. L.; Morokuma, K.; Zakrzewski, V. G.; Voth, G. A.; Salvador, P.; Dannenberg, J. J.; Dapprich, S.; Daniels, A. D.; Farkas, O.; Foresman, J. B.; Ortiz, J. V.; Cioslowski, J.; Fox, D. J. Gaussian, Inc., Wallingford CT, 2013.

(8) Andrea, D.; Häußermann, U. M.; Dolg, M.; Stoll, H.; Preuss, H. Energyadjusted ab initio pseudopotentials for the second and third row transition elements. *Theor. Chim. Acta* **1990**, *77*, 123–141.

(9) Ehlers, A. W.; Bohme, M.; Dapprich, S.; Gobbi, A.; Hollwarth, A.; Jonas, V.; Kohler, K. F.; Stegmann, R.; Veldkamp, A.; Frenking, G. A set of f-polarization

functions for pseudo-potential basis sets of the transition metals SC-Cu, Y-Ag and La-Au. *Chem. Phys. Lett.* **1993**, *208*, 111–114.

(10) (a) Hehre, W. J.; Ditchfield, R.; Pople, J. A. Self-Consistent Molecular Orbital Methods. XII. Further Extensions of Gaussian-Type Basis Sets for Use in Molecular Orbital Studies of Organic Molecules. *J. Chem. Phys.* **1972**, *56*, 2257–2261. (b) Francl, M. M.; Pietro, W. J.; Hehre, W. J.; Binkley, J. S.; Gordon, M. S.; DeFrees, D. J.; Pople, J. A. Self-consistent molecular orbital methods. XXIII. A polarization-type basis set for second-row elements. *J. Chem. Phys.* **1982**, *77*, 3654–3665.

(11) Marenich, A. V.; Cramer, C. J.; Truhlar, D. G. Universal Solvation Model Based on Solute Electron Density and on a Continuum Model of the Solvent Defined by the Bulk Dielectric Constant and Atomic Surface Tensions. *J. Phys. Chem. B* **2009**, *113*, 6378–6396.

(12) O'Boyle, N. M.; Tenderholt, A. L.; Langner, K. M. cclib: A Library for Package-Independent Computational Chemistry Algorithms. *J. Comput. Chem.* **2008**, *29*, 839−845.
